# Evidence for the role of transposons in the recruitment of *cis*-regulatory motifs during the evolution of C4 photosynthesis

**DOI:** 10.1186/s12864-016-2519-3

**Published:** 2016-03-08

**Authors:** Chensi Cao, Jiajia Xu, Guangyong Zheng, Xin-Guang Zhu

**Affiliations:** CAS Key Laboratory for Computational Biology, CAS-MPG Partner Institute for Computational Biology, Chinese Academy of Sciences, Room 102, Physiology Building, 320 Yueyang Road, Shanghai, 200031 China

**Keywords:** *cis*-regulatory elements, Motif recruitment, Transposons, Binding affinity

## Abstract

**Background:**

C_4_ photosynthesis evolved from C_3_ photosynthesis and has higher light, water, and nitrogen use efficiencies. Several C_4_ photosynthesis genes show cell-specific expression patterns, which are required for these high resource-use efficiencies. However, the mechanisms underlying the evolution of *cis-*regulatory elements that control these cell-specific expression patterns remain elusive.

**Results:**

In the present study, we tested the hypothesis that the *cis-*regulatory motifs related to C_4_ photosynthesis genes were recruited from non-photosynthetic genes and further examined potential mechanisms facilitating this recruitment. We examined 65 predicted bundle sheath cell-specific motifs, 17 experimentally validated cell-specific *cis-*regulatory elements, and 1,034 motifs derived from gene regulatory networks. Approximately 7, 5, and 1,000 of these three categories of motifs, respectively, were apparently recruited during the evolution of C_4_ photosynthesis. In addition, we checked 1) the distance between the acceptors and the donors of potentially recruited motifs in a chromosome, and 2) whether the potentially recruited motifs reside within the overlapping region of transposable elements and the promoter of donor genes. The results showed that 7, 4, and 658 of the potentially recruited motifs might have moved via the transposable elements. Furthermore, the potentially recruited motifs showed higher binding affinity to transcription factors compared to randomly generated sequences of the same length as the motifs.

**Conclusions:**

This study provides molecular evidence supporting the hypothesis that transposon-driven recruitment of pre-existing *cis-*regulatory elements from non-photosynthetic genes into photosynthetic genes plays an important role during C_4_ evolution. The findings of the present study coincide with the observed repetitive emergence of C_4_ during evolution.

**Electronic supplementary material:**

The online version of this article (doi:10.1186/s12864-016-2519-3) contains supplementary material, which is available to authorized users.

## Background

C_4_ photosynthesis differs from C_3_ photosynthesis by possessing a CO_2_ concentrating mechanism, which enables C_4_ plants to achieve higher light, water, and nitrogen use efficiencies [1, 2]. The higher photosynthetic efficiency in C_4_ plants is achieved by elevating the concentration of CO_2_ around ribulose-1,5-bisphosphate carboxylase/oxygenase (RuBisCO). Extensive efforts have been made to engineer a C_4_ photosynthetic machinery into C_3_ plants such as rice and wheat [3, 4]. Elucidation of the molecular mechanism underlying the evolution of the key components of the concentrating process in C_4_ plants and identifying its molecular regulators, either as *cis*-regulatory elements or *trans*-factors and controlling C_4_ photosynthetic features [5, 6] are necessary to successfully perform C_4_ photosynthesis [5, 7]. To date, despite the establishment of the biochemical and anatomical features of C_4_ photosynthesis, our understanding of the genetic control of various C_4_ properties such as the reduction in interveinal distance, increased number of chloroplasts within bundle sheath (BS) cells, extensive differentiation of M and BS chloroplast proteomes, and higher plasmodesmata abundance for transport between M and BS cells [3] is limited. More efforts to identify regulatory elements required for the establishment of cell-specific expression patterns of C_4_ photosynthesis-related genes are warranted. Various approaches, including both forward genetics and reverse genetics approaches, have been utilized to address this question [8].

To date, the *cis-*regulatory motifs controlling the cell-specific expression patterns of C_4_-related genes have been mainly discovered through experimental approaches such as deletion analysis [4, 5]. Recent technological advances in computational biology have facilitated the identification of motifs [9–11]. Such computational analyses usually start with clustering genes from a transcriptomic data set into different clusters, followed by prediction of motifs in genes from each cluster [9–11]. One underlying assumption of this approach is that genes within the same cluster are potentially regulated by common *cis-*regulatory elements. However, there are circumstances where this assumption is violated. For example, let us consider three genes, *A, B*, and C. Genes *A* and *B* are regulated by the same *cis-*regulatory elements, whereas *C* is regulated by *B* and hence shows the same expression pattern as that of *B*. If the expression pattern is used as the sole criterion in clustering these genes, then these three genes will be misclassified into the same gene cluster, which in turn can lead to the inaccurate detection of *cis*-regulatory elements. Gene regulatory networks constructed based on conditional mutual information can solve the issue of misclassifying genes into the same cluster because this algorithm only detects genes with direct regulatory relationships [12].

In the present study, we examined the potential mechanism related to the formation of new *cis-*regulatory elements in the promoter region of C_4_-related genes. To do this, we first identified the potentially recruited *cis-*regulatory elements using gene regulatory networks constructed based on conditional mutual information. Furthermore, we used three sets of motifs, i.e., network-derived motifs, experimentally identified *cis-*regulatory elements, and predicted bundle sheath specific motifs, to test whether these exist in the genes directly linked to the C_3_ ortholog of C_4_ genes in a C_3_ gene regulatory network. Lastly, to explore the potential mechanisms responsible for the recruitment of these motifs, we explored whether the potentially recruited motifs reside in the overlapping regions between transposable elements (TEs) and promoter regions of the C_4_ genes. We discussed all these results in light of the hypothesis that transposons play a role in the recruitment of these motifs during the emergence of C_4_ photosynthesis.

## Results

### Identification of *cis-*regulatory elements that were potentially recruited during C_4_ evolution

**Fig. 1:**
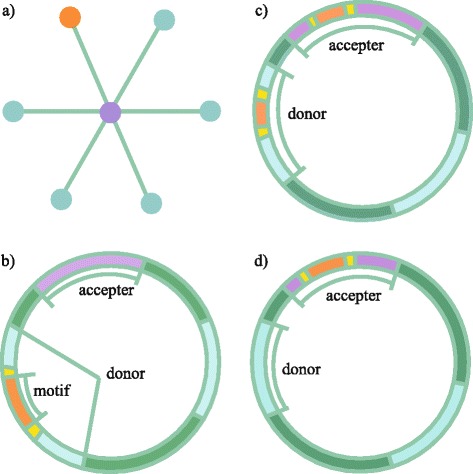
Distribution of motifs in genes before and after recruitment. **a** Before the recruitment, the donor (*orange dot*) is located in a neighboring gene of the acceptor (*purple dot*) in a gene regulatory network (GRN). **b** Before the recruitment, the donor contains the motif (*orange block*) and the acceptor (*purple block*) lacks the motif. **c** After a copy-and-paste recruitment, the acceptor (*purple block*) contains the motif (*orange block*, whereas the donor also maintains the motif. **d** After a cut-and-paste recruitment, the acceptor (*purple block*) recruits the motif (*orange block*), whereas the donor loses the motif

Based on the strategy shown in Fig. 1, 40 pairs of orthologs of the C_4_ genes in maize and rice, including its promoter sequences, were obtained. We checked the distribution of BS cell-specific motifs in these 40 pairs (see Methods). We identified seven motifs that might have been potentially recruited during C_4_ evolution based on the following criteria: a) these are differentially distributed in C_4_ and C_3_ orthologs; b) these existed in the neighboring genes of the C_3_ orthologs (Table 1).

**Table 1:**
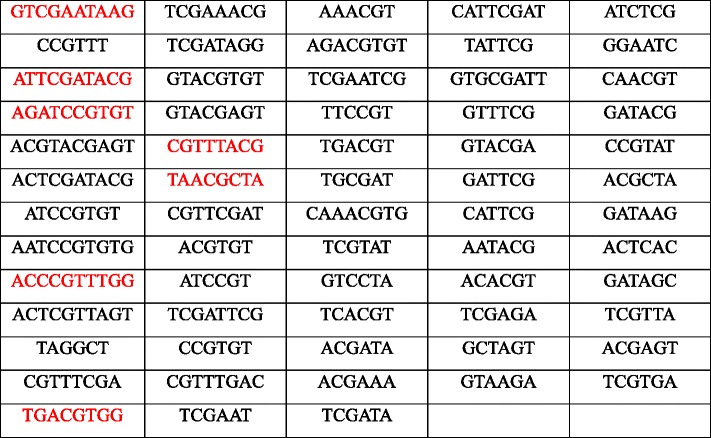
Predicted bundle sheath cells specific motifs. The potentially recruited motifs are shown in red color.

### Evidence for potential involvement of transposon in motif recruitment

**Fig. 2:**
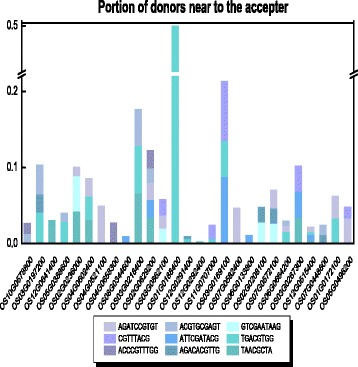
Proportion of donors that were proximal to the acceptor. For each acceptor (X axis) and each potentially recruited bundle sheath-specific motif (colored block), the proportion of donors (colored segments along the Y axis) that were near to the acceptor in the chromosome where the acceptor gene resides is indicated

We next tested whether transposons played a role during this motif movement. Considering that a small chromosomal distance facilitates transposition, we first determined the distance between the donor gene to the acceptor gene relative to the total length of the chromosome, and identified candidate donor genes that were located <1/10 of the total length of the chromosome from the acceptor genes (Fig. 2; Additional file 1: Table S3). Furthermore, when TE transferred a motif to another locus, the particular motif should be within the overlapping region of TEs and the promoters of the donor gene. We hence aligned the sequences of TEs with those of the promoters of donor genes by using BLAST [13] and identified the overlapping regions. All seven motifs that were differentially distributed in C_4_ and C_3_ orthologs were indeed present in the overlapping regions of TEs and candidate donors of motifs (Table 2). These two pieces of evidence suggest that transposons may have played a role in the recruitment of these BS cell-specific motifs.

**Table 2:**
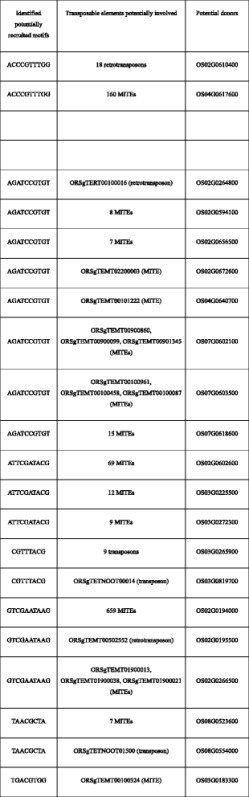
Predicted bundle sheath cell specific motifs that might have been recruited into C_4_ related enzymes through transposable elements

### Recruited motifs are possible binding sites for transcription factors

Earlier reports have shown that TEs contribute to the formation of new TF binding sites [14, 15] and evolution of new regulatory mechanisms. Here we checked whether the recruited motifs are potential binding sites of transcription factors (TFs). We tested 124 TFs, for which the position weight matrix information is available from TRANSFAC (Additional file 1: Table S4). For the potentially recruited motifs and TFs, we calculated their binding possibilities (see Methods). Seven of these potentially recruited motifs showed higher (more than two-fold) binding affinities with TFs compared to the calculated affinity for random elements of the same length as the motif (Table 3; see Methods). Several of the TFs binding to these motifs were earlier identified to be potential regulators of photosynthesis such as Opaque-2 and GBF (Table 4) [16].

**Table 3:**
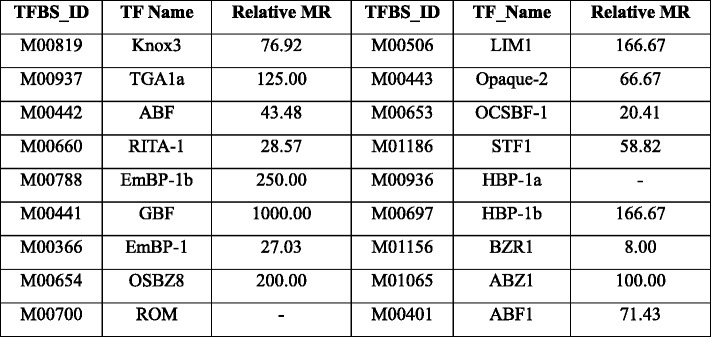
Potentially recruited bundle sheath cells specific motifs have higher binding affinity with TFs compared to randomly generated sequence of the same length

**Table 4:**
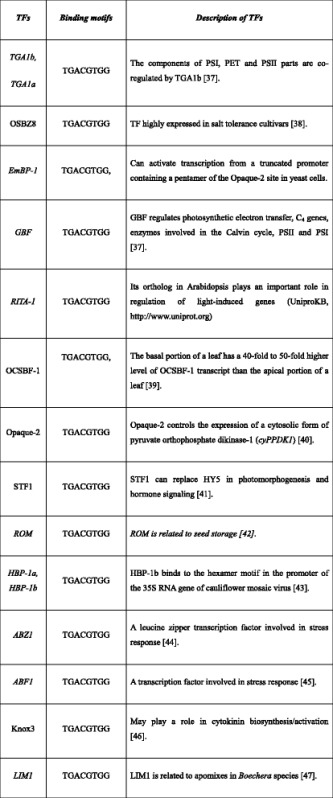
The identified transcription factors which showed high binding affinities to BS cell specific motifs and brief description of the TFs

### Occurrence of TE-driven motif recruitment in the experimentally validated motifs

To test whether TE-driven motif recruitment is a general phenomenon, we further examined whether TEs are involved in the recruitment of motifs that were experimentally identified to be related to their host gene’ cell-specific gene expression pattern [5] and also the predicted *cis-*regulatory motifs based on the genes in the same gene community in a gene regulatory network.

**Fig. 3:**
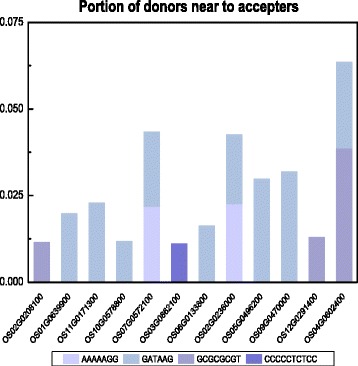
Proportion of donors that were proximal to the acceptor where experimentally validated *cis*-regulatory element (motif) resides. For each acceptor (X axis) and each potentially recruited motif (colored block), the proportion of donors (colored segments along Y axis) that were near to acceptor where the acceptor gene resides is indicated

Of the 17 experimentally validated motifs, five were identified as potentially recruited motifs (Additional file 1: Table S5). Of these five potentially recruited motifs, four resided in the overlapping region of TEs and their candidate donors (Table 5; Fig. 3). In addition, the donors of these four motifs were proximally located to the acceptors in their residing chromosome (Additional file 1: Table S6). Similar to the analysis of the BS cell-specific motifs, the five putative recruited and validated motifs showed higher binding affinity to TFs (Additional file 1: Table S7).

**Table 5:**
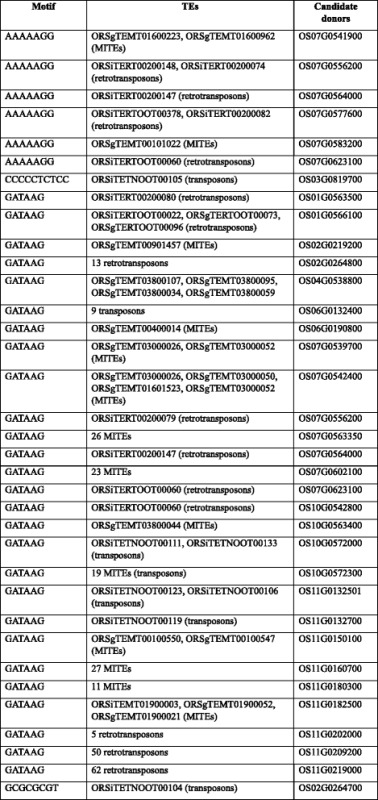
Transposable elements that may have played a crucial role in mediating transfer of experimentally validated motifs from the candidate donors to a C_4_ acceptor gene

We also obtained similar results in the analysis of network-derived motifs. There were 1,034 motifs differentially distributed in the C_4_ and C_3_ orthologs, and 1,000 of these were identified as potential recruited motifs (Additional file 2). A total of 658 of the 1,000 potentially recruited motifs were present in the overlapping region of TEs and candidate donors (Additional file 3), whereas the donors were situated proximal to the acceptors (Additional file 4). We also calculated the binding capacity of network-derived motifs (Additional file 5).

## Discussion

### Evidence supporting the recruitment of pre-existing *cis*-regulatory elements from non-photosynthetic genes into C_4_ genes

In the present study, we evaluated the possibility that a motif may have been recruited if it satisfies two criteria. First, this motif pre-exists in the neighboring genes surrounding an ortholog of a C_4_ photosynthetic gene in the rice genetic regulatory network; however, the orthologs of the C_4_ photosynthesis gene does not contain this motif. Second, this motif appears in the C_4_ ortholog of this photosynthesis gene. Based on these two criteria, we identified 7 out of the 65 bundle-sheath specific motifs to have been recruited during C_4_ emergence (Table 1). In addition to these predicted BS-specific motifs, we further examined the recruitment of *cis-*regulatory elements previously identified through experimental approaches to be associated with cell-specific expression [5], as well as motifs derived from gene regulatory networks. The results of these analyses also suggested a large-scale recruitment of pre-existing *cis-*regulatory elements from non-photosynthetic genes into C_4_ photosynthetic genes during C_4_ evolution (Additional file 1: Tables S5, S6 and S7; Additional files 2, 3, 4 and 5).

### Evidence for the potential role of transposable element in the recruitment of C_4_-specific motifs

TEs contribute to the interactions among various gene regulatory networks and the control the expression of genes [17–20] and lncRNA [21]. These can potentially contain binding sites for TFs [20]. Earlier work has suggested that about half of TF-binding sites are derived from TEs in human and mouse [14]. TEs may therefore contribute to the evolution of species-specific regulatory functions and phenotypes. In the present study, all seven putative recruited BSC-specific motifs were detected within the overlapping region of TEs and the promoter regions of candidate donor gene (Table 2). Furthermore, these donors are located near the acceptors in one chromosome, suggesting that TEs may have played an important role in the recruitment process. Similar results were obtained for the experimentally validated motifs and network-derived motifs (Additional file 1: Tables S5, S6 and S7; Additional files 2, 3, 4, 5 and 6). Similar to the function of TEs in human and mouse [14], the recruited motifs showed higher binding affinity to TFs. We hence propose that these putative recruited motifs might have contributed to the formation of new TF-binding sites and consequently modified the interactions among various gene regulatory networks in rice and maize. Not all of the putative recruited motifs were presented within the overlapping regions of TEs and the promoter regions of candidate donor genes (Table 1), thereby suggesting other mechanisms for the emergence of these motifs during C_4_ evolution.

### Implications of transposon-driven recruitment of *cis-*regulatory elements to the evolution of C_4_ photosynthesis

C_4_ photosynthesis differs from C_3_ photosynthesis in various aspects, including recruitment of new decarboxylation enzymes, re-adjustment of nitrogen metabolism, starch metabolism, and partitioning of the photosynthetic enzymes or proteins into bundle sheath and mesophyll cells [22]. Given these large number of differences between C_3_ and C_4_ photosynthesis, it is remarkable that C_4_ photosynthesis has independently emerged in more than 60 lineages [23]. Furthermore, the emergence of C_4_ photosynthesis occurred within a relatively short geological period. This is because 40 million years ago, the global atmospheric CO_2_ concentration dropped, and C_4_ photosynthesis started to show its competitive advantage over C_3_ photosynthesis [1], thereby eventually resulting in C_4_ photosynthesis 20 million years later [24]. How can such a complex trait have evolved in such a short timeframe? This study provides new sequence-based evidences that recruitment of pre-existing motifs might have been a mechanism for C_4_ evolution, which in turn may have contributed to the rapid evolution of C_4_ photosynthesis.

Furthermore, we showed that the new regulatory mechanism involving C_4_ photosynthesis might have been created through transposon-mediated motif movements. Genome duplication has been regarded as a major mechanism responsible for the creation of material for neofunctionalization or creation of new genes during C_4_ emergence [25]. However, several recent analyses have shown that the copy number of C_4_-related genes are not necessarily higher than those in C_3_ species [26]. Transposon-driven creation of new genes hence might have been used as an alternative mechanism for the creation of novel regulatory mechanisms for C_4_-related genes. Furthermore, considering that during C_4_ evolution, not only those motifs from the promoter regions, but also those in the coding sequences were potentially recruited [26–28]. Therefore, transposons were utilized as ideal mechanism for the recruitment of regulatory motifs because these can mobilize elements without particular location preferences [21]. Considering that there are relatively a lower number of whole genome duplication events during the evolution of land plants, this transposon-driven emergence of new genes might have been the predominant mechanism that has substantially contributed to the rapid evolution of new functions during the evolution of C_4_ photosynthesis of the low-CO_2_ oligocene period [24]. Additional experimental evidence is needed to test this potential mechanism.

## Conclusions

The present study has provided sequence-based evidence that suggests that transposon-mediated movement of motifs might have played a role in the formation of new *cis-*regulatory elements during the evolution of C_4_ photosynthesis. More experiments are needed to test this possibility. However, if this is true, then this may serve as a possible mechanism for the rapid emergence of C_4_ photosynthesis within a relatively short geological period during the Oligocene [24].

## Methods

**Fig. 4:**
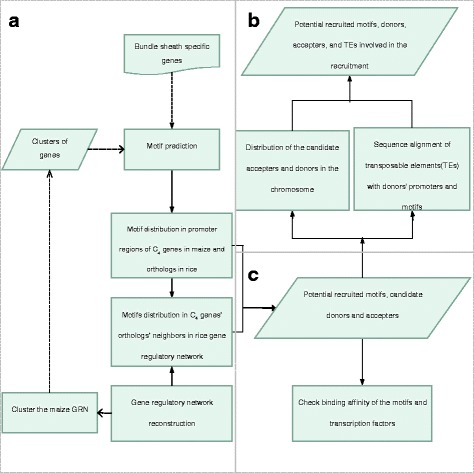
Pipeline for the analysis of motif recruitment. **a** analysis of the possibility of motif recruitment; **b** analysis to determine whether a transposable element is involved in the recruitment; **c** analysis to determine whether a recruited motif is a potential binding target of transcription factors

The whole analysis pipeline was composed of three sessions: 1) analysis of the possibility of motif recruitment; 2) analysis of whether TE-mediated motif movement served as the mechanism responsible for the observed motif recruitment; 3) analysis of whether the recruited motifs served as binding targets of TFs. The pipeline of the analysis is shown in Fig. 4, and the details are described in the following sections.

### Gene regulatory network reconstruction and motif prediction based on the network

The rice and maize GRNs were built using a PCA-CMI algorithm [12] using rice and maize transcriptomics data. With the constructed maize gene regulatory network, we classified the genes into communities with Markovian clustering algorithm [29] (MCL, http://micans.org/mcl/), and in each community of genes, we predicted motifs by using the Weeder2.0 software [30] (http://159.149.160.51/modtools/). We obtained a total of 54 communities and 1,649 motifs (hereby defined as network-derived motifs).

### *De novo* prediction of BS-cell specific motifs

We downloaded transcriptomics data for both the BS cells and mesophyll cells [31]. A total of 1,045 genes that showed relatively higher expression levels in BS cells were classified into clusters by K-mean clustering, with the number of clusters selected based on Figure of merits (FOM) using the R package clValid (http://cran.r-project.org/web/packages/clValid/index.html). The motifs of genes of each cluster were predicted by using Weeder2.0 using the sequence 3 kb upstream of the transcription start site (TSS). We obtained 65 motifs in BS-specific genes (Table 1). These motifs were annotated as BS cell-specific motifs.

### Distribution of motifs in promoter regions of C_4_-related genes of maize and its orthologs in rice

When a particular motif (orange block, Fig. 1) is recruited into an acceptor gene (purple block/dot, Fig. 1) from a neighboring gene (orange dot, Fig. 1) in the gene regulatory network (GRN, Fig. 1), it is necessary that the promoter region of the donor gene contains this motif and the acceptor lacks this motif prior to the recruitment event (Fig. 1). In the present study, we focused on motif recruitment into 78 C_4_ genes [31] (Additional file 1: Table S8).

As illustrated in Fig. 1, we first scanned the sequence 3 kb upstream of the TSS (downloaded from Ensembl Plant, http://plants.ensembl.org/index.html) in the C_4_ genes and their orthologous genes in rice to check whether a particular motif was present or not. For a *cis-*regulatory element validated experimentally to be involved in C_4_ photosynthesis, we aligned the element to the promoter sequences of C_4_ genes and their orthologous genes in rice. For predicted motifs based on gene regulatory network, we used MAST [32] (http://meme.nbcr.net/meme/tools/mast) to check its distribution in the promoters of genes in the genome.

We then examined whether the motifs differentially existed between C_4_ genes and their orthologous C_3_ genes. For those motifs differentially distributed between C_4_ and C_3_ orthologs, we examined its distribution in the rice gene regulatory network to determine whether there is a possibility that motifs in C_4_ orthologs were recruited from the neighboring genes.

### Distribution of the candidate acceptors and donors in the chromosome

To assess whether the donor genes were proximal to the acceptor gene, we identified the donors residing within a region around 1/10 of the length of chromosome surrounding the acceptor gene (i.e., *d*(*accepter*, *donor*) < 0.1). The length and number of genes in all 12 rice chromosomes were downloaded from NCBI (http://www.ncbi.nlm.nih.gov/assembly/GCF_000005425.2). The genes locus and description were downloaded from RAP-DB (http://rapdb.dna.affrc.go.jp/). The distance between acceptor and donor was calculated as follows:

$$ d\left( accepter,\kern0.4em  donor\right)=\frac{donor\kern0.4em  start\kern0.4em  site- accepter\kern0.4em  start\kern0.4em  site}{chromosome\kern0.4em  length} $$

### Sequence alignment of TEs with the promoters and motifs of donor genes

We further examined whether a motif resides within the overlapping region between TEs and the promoter region of the donor gene. To do this, we first checked the distribution of motifs in different categories of TEs, i.e., retrotransposons, class II transposons, including the miniature inverted-repeat transposable elements (MITEs), which have earlier been shown to be important in determining species diversity in *Oryza sativa* [33–35]. The sequences of these TEs were downloaded from Plant Repeat Databases [36] (http://plantrepeats.plantbiology.msu.edu/index.html). We aligned the sequences of TEs containing a particular motif with the promoter regions of candidate donors of this motif by using BLAST (http://blast.ncbi.nlm.nih.gov/Blast.cgi) to identify the overlapping region, and checked the distribution of motifs across the overlapping region.

### Enrichment of motifs in TF binding sites

To explore whether a recruited motif can potentially function as a binding site for TFs, we aligned motifs with the position weight matrix information for TFs (downloaded from TRANSFAC) (http://www.gene-regulation.com/pub/databases.html). To test whether the motif enrichment was statistically significant, for each motif, we aligned to the TF binding sites PWM, we randomly constructed 1,000 short sequences with the same length of the motif and aligned these to TF binding sites PWM. For both the potential recruited motifs and the randomly constructed sequences, we calculated the match ratio (MR), which is defined as $MR\left( elements,\kern0.4em TF\right)=\frac{\left|\kern0.1em  elements\kern0.4em  matched\kern0.4em to\kern0.4em  the\kern0.4em TF\kern0.4em  bingding\kern0.4em  site\right|}{\left| elements\right|}$; where |*S* | is the size of a set *S,* and *elements* can either be the potentially recruited motifs set or random sequences set of the same length as the potentially recruited motifs. The binding affinity of the motifs and the TFs were then assessed by the relative match ratio (relative MR) $relative\kern0.4em MR\left( motifs,\kern0.4em TF\right)=\frac{MR\left( motifs,\kern0.4em TF\right)}{MR\left( random\kern0.4em  elements,\kern0.4em TF\right)}$; where random elements are random sequences of the same length as potentially recruited motifs. Only elements with lengths > 5 were considered in this study.

## Availability of supporting data

The data sets used to construct GRN for rice and maize in the present study along with their NCBI accession numbers are listed in Additional file 1: Tables S1 and S2 (http://www.ncbi.nlm.nih.gov/sra/). The sequences of genes for both rice and maize were downloaded from Ensembl Plant (http://plants.ensembl.org/index.html). Location and description of rice genes were downloaded from (RAP-DB, http://rapdb.dna.affrc.go.jp/). Information on genomes of *Oryza sativa* was downloaded from NCBI (http://www.ncbi.nlm.nih.gov/assembly/GCF_000005425.2). Sequences of transposable elements in *Oryza sativa* were downloaded from a plant repeat database (http://plantrepeats.plantbiology.msu.edu/index.html). Sequences of TF binding sites were downloaded from TRANSFAC (Additional file 1: Table S5). The list of C_4_ genes is presented in Additional file 1: Table S8.

## Additional files

Additional file 1: Table S1. Basic information on the collected RNA-SEQ data on rice mature leaves that was used in the construction of the rice gene regulatory network. **Table S2.** Basic information of collected RNA-SEQ data on maize mature leaves that was used in the construction of the maize gene regulatory network. **Table S3.** Candidate acceptors and donors of potentially recruited BS cell-specific motifs. Those donors that were not farther than 10 genes along the same chromosome where the acceptor gene resides are listed. A brief description of the acceptor gene is also provided. **Table S4.** Brief description of TFs binding sites obtained from the TRANSFAC database. **Table S5.** Experimentally validated cell-specific motifs. Those potentially recruited motifs during C_4_ evolution are labeled as red. **Table S6** ***.*** Candidate acceptors and donors of potentially recruited C_4_ related motifs were experimentally validated. Donors not farther than 10 genes along the same chromosome where the acceptor gene resides are listed. A brief description of the acceptor gene is also provided. **Table S7.** The potentially recruited experimentally validated motifs show higher binding affinities with TFs compared to randomly generated sequences of the same length as the motif. **Table S8.** List of C_4_ genes used in the analysis of the present study. These genes were obtained from Li et al. 2010. (PDF 360 kb) Additional file 2: Potentially recruited network-derived motifs related to C4 photosynthesis. (XLSX 15 kb) Additional file 3: Potentially recruited network-derived motifs located within the overlapping region of transposable elements and the promoter region of the donor gene. (XLSX 1074 kb) Additional file 4: Candidate acceptors and donors of the recruited network-derived motifs, along with a brief description of each acceptors. Only those donor genes that were not farther than 10 genes from the acceptor gene are listed. (XLSX 110 kb) Additional file 5: Potentially recruited network-derived motifs detected within transcription factor binding sites (TFBS). (XLSX 27 kb) Additional file 6: Network-derived motifs with the probability of ‘A,’ ‘T,’ ‘C,’ ‘G’ in each site. (PDF 708 kb)
