# Supplementary material for: Evidence for the role of transposons in the recruitment of cis-regulatory motifs during the evolution of C4 photosynthesis
Source: BMC Genomics. 2016 Mar 8;17:201. doi: 10.1186/s12864-016-2519-3 (PMC4782515; doi:10.1186/s12864-016-2519-3)
Supplement: Additional file 1: Table S1. — Basic information on the collected RNA-SEQ data on rice mature leaves that was used in the construction of the rice gene regulatory network. Table S2. Basic information of collected RNA-SEQ data on maize mature leaves that was used in the construction of the maize gene regulatory network. Table S3. Candidate acceptors and donors of potentially recruited BS cell-specific motifs. Those donors that were not farther than 10 genes along the same chromosome where the acceptor gene resides are listed. A brief description of the acceptor gene is also provided. Table S4. Brief description of TFs binding sites obtained from the TRANSFAC database. Table S5. Experimentally validated cell-specific motifs. Those potentially recruited motifs during C4 evolution are labeled as red. Table S6 . Candidate acceptors and donors of potentially recruited C4 related motifs were experimentally validated. Donors not farther than 10 genes along the same chromosome where the acceptor gene resides are listed. A brief description of the acceptor gene is also provided. Table S7. The potentially recruited experimentally validated motifs show higher binding affinities with TFs compared to randomly generated sequences of the same length as the motif. Table S8. List of C4 genes used in the analysis of the present study. These genes were obtained from Li et al. 2010. (PDF 360 kb) [file 12864_2016_2519_MOESM1_ESM.pdf]

Additional file 1

Table S1. Basic information of collected experiments on rice mature leaves used to construct the rice gene regulatory network.

SRA	Platform	Layout	tissue	Submitted
SRR034580	Illumina Genome Analyzer	SINGLE	shoots from four-leaf stage	GEO
SRR034581	Illumina Genome Analyzer	SINGLE	shoots from four-leaf stage	GEO
SRR034582	Illumina Genome Analyzer	SINGLE	shoots from four-leaf stage	GEO
SRR034583	Illumina Genome Analyzer	SINGLE	shoots from four-leaf stage	GEO
SRR034584	Illumina Genome Analyzer	SINGLE	shoots from four-leaf stage	GEO
SRR034585	Illumina Genome Analyzer	SINGLE	shoots from four-leaf stage	GEO
SRR034586	Illumina Genome Analyzer	SINGLE	shoots from four-leaf stage	GEO
SRR034587	Illumina Genome Analyzer	SINGLE	shoots from four-leaf stage	GEO
SRR034588	Illumina Genome Analyzer	SINGLE	shoots from four-leaf stage	GEO
SRR034589	Illumina Genome Analyzer	SINGLE	shoots from four-leaf stage	GEO
SRR034590	Illumina Genome Analyzer	SINGLE	shoots from four-leaf stage	GEO
SRR034591	Illumina Genome Analyzer	SINGLE	shoots from four-leaf stage	GEO
SRR034592	Illumina Genome Analyzer	SINGLE	shoots from four-leaf stage	GEO
SRR034593	Illumina Genome Analyzer	SINGLE	shoots from four-leaf stage	GEO

SRR034594	Illumina Genome Analyzer	SINGLE	shoots from four-leaf stage	GEO
SRR034595	Illumina Genome Analyzer	SINGLE	shoots from four-leaf stage	GEO
SRR034596	Illumina Genome Analyzer	SINGLE	shoots from four-leaf stage	GEO
SRR034597	Illumina Genome Analyzer	SINGLE	shoots from four-leaf stage	GEO
SRR034598	Illumina Genome Analyzer	SINGLE	shoots from four-leaf stage	GEO
SRR034599	Illumina Genome Analyzer	SINGLE	shoots from four-leaf stage	GEO
SRR305463	Illumina Genome Analyzer IIx	SINGLE	Rice SS rep01	BTI,CU
SRR305476	Illumina Genome Analyzer IIx	SINGLE	Rice SS rep02	BTI,CU
SRR305477	Illumina Genome Analyzer IIx	SINGLE	Rice SS rep03	BTI,CU
SRR305891	Illumina Genome Analyzer IIx	SINGLE	Rice NSS rep02	BTI,CU
SRR305892	Illumina Genome Analyzer IIx	SINGLE	Rice NSS rep03	BTI,CU
SRR305893	Illumina Genome Analyzer IIx	SINGLE	Rice NSS rep01	BTI,CU
SRR504364	Illumina Genome Analyzer II	SINGLE	OSJG, leaf tissue	GEO
SRR504365	Illumina Genome Analyzer II	SINGLE	OSJG, leaf tissue	GEO
SRR711322	Illumina HiSeq 2000	SINGLE	OSJG, leaf tissue, wt	GEO
SRR711323	Illumina HiSeq 2001	SINGLE	OSJG, leaf tissue, wt	GEO
SRR711324	Illumina HiSeq 2002	SINGLE	OSJG, leaf tissue, PiZt 11	GEO
SRR711325	Illumina HiSeq 2003	SINGLE	OSJG, leaf tissue, PiZt 11	GEO
SRR711326	Illumina HiSeq 2004	SINGLE	OSJG, leaf tissue, PiZt 12	GEO
SRR711327	Illumina HiSeq 2005	SINGLE	OSJG, leaf tissue, PiZt 13	GEO
SRR711328	Illumina HiSeq 2006	SINGLE	OSJG, leaf tissue, PiZt 14	GEO

SRR711329	Illumina HiSeq 2007	SINGLE	OSJG, leaf tissue, Pi9	GEO
SRR711330	Illumina HiSeq 2008	SINGLE	OSJG, leaf tissue, Spin1 RNAi	GEO

OSJG: *Oryza sativa Japonica* Group; BTI,CU: Boyce Thompson Institute, Cornell University

Table S2. Basic information of collected experiments on maize mature leaves used to construct the rice gene regulatory network.

**SRA**	**Platform**	**Layout**	**tissue**	**Submitted by**
SRR189774	Illumina	SINGLE	ZB-20	Michigan State University
SRR404149	Illumina	SINGLE	Zea Mays, B73 V5_Tip of stage-2 Leaf_R1	MSU-BUELL
SRR404150	Illumina	SINGLE	Zea Mays, B73 V5_Tip of stage-2 Leaf_R1	MSU-BUELL
SRR404152	Illumina	SINGLE	Zea Mays, B73 V5_Tip of stage-2 Leaf_R2	MSU-BUELL
SRR404153	Illumina	SINGLE	Zea Mays, B73 V5_Tip of stage-2 Leaf_R3	MSU-BUELL
SRR404154	Illumina	SINGLE	Zea Mays, B73 V9_Eighth Leaf_R1	MSU-BUELL
SRR404155	Illumina	SINGLE	Zea Mays, B73 V9_Eighth Leaf_R2	MSU-BUELL
SRR404156	Illumina	SINGLE	Zea Mays, B73 V9_Eighth Leaf_R3	MSU-BUELL
SRR404158	Illumina	SINGLE	Zea Mays, B73 V9_Eleventh Leaf_R1	MSU-BUELL
SRR404159	Illumina	SINGLE	Zea Mays, B73 V9_Eleventh Leaf_R2	MSU-BUELL
SRR404160	Illumina	SINGLE	Zea Mays, B73 V9_Eleventh Leaf_R3	MSU-BUELL
SRR404161	Illumina	SINGLE	Zea Mays, B73 V9_Thirteenth Leaf_R1	MSU-BUELL
SRR404162	Illumina	SINGLE	Zea Mays, B73 V9_Thirteenth Leaf_R2	MSU-BUELL
SRR404163	Illumina	SINGLE	Zea Mays, B73 V9_Thirteenth Leaf_R3	MSU-BUELL
SRR404173	Illumina	SINGLE	Zea Mays, B73 VT_Thirteenth Leaf_R1	MSU-BUELL

SRR404174	Illumina	SINGLE	Zea Mays, B73 VT_Thirteenth Leaf_R2	MSU-BUELL
SRR404175	Illumina	SINGLE	Zea Mays, B73 VT_Thirteenth Leaf_R3	MSU-BUELL
SRR404176	Illumina	SINGLE	Zea Mays, B73 R2_Thirteenth Leaf_R1	MSU-BUELL
SRR404177	Illumina	SINGLE	Zea Mays, B73 R2_Thirteenth Leaf_R2	MSU-BUELL
SRR404178	Illumina	SINGLE	Zea Mays, B73 R2_Thirteenth Leaf_R3	MSU-BUELL
SRR501912	Illumina	PAIRED	B73 leaf rep1	Minnesota
SRR501913	Illumina	PAIRED	B73 leaf rep2	Minnesota
SRR502716	Illumina	PAIRED	B73 leaf rep3	Minnesota
SRR942916	Illumina	PAIRED	Zea Mays, FP5	Oxford
SRR942911	Illumina	PAIRED	Zea Mays, FI	Oxford
SRR942910	Illumina	PAIRED	Zea Mays, FE	Oxford
SRR531218	Illumina	SINGLE	Zea Mays, B73 V3 Topmost leaf Rep1	MSU-BUELL
SRR531219	Illumina	SINGLE	Zea Mays, B73 V3 Topmost leaf Rep2	MSU-BUELL
SRR531220	Illumina	SINGLE	Zea Mays, B73 V3 Topmost leaf Rep3	MSU-BUELL
SRR531869	Illumina	SINGLE	B73 V7 Tip of transition leaf Rep1	MSU-BUELL
SRR531870	Illumina	SINGLE	B73 V7 Tip of transition leaf Rep2	MSU-BUELL
SRR531871	Illumina	SINGLE	B73 V7 Tip of transition leaf Rep3	MSU-BUELL

ZB-20: Zea mays,B73 Leaves 20-dayold seedling-growth chamber RNA-Seq
Oxford: Oxford
Minnesota: Univeristy of Minnesota

Table S3. Bundle sheath cells specific motifs cells specific motifs. (Potential recruited motifs are colored in red)

GTCGAATAAG	TCGAAACG	AAACGT	CATTCGAT	ATCTCG
ACGTGCGAGT	TCGATAGG	AGACGTGT	TATTCG	GGAATC

ATTCGATACG	GTACGTGT	TCGAATCG	GTGCGATT	CAACGT
AGATCCGTGT	GTACGAGT	TTCCGT	GTTTCG	GATACG
ACGTACGAGT	CGTTTACG	TGACGT	GTACGA	CCGTAT
ACTCGATACG	TAACGCTA	TGCGAT	GATTCG	ACGCTA
ATCCGTGT	CGTTCGAT	CAAACGTG	CATTCG	GATAAG
AATCCGTGTG	ACGTGT	TCGTAT	AATACG	ACTCAC
ACCCGTTTGG	ATCCGT	GTCCTA	ACACGT	GATAGC
ACTCGTTAGT	TCGATTCG	TCACGT	TCGAGA	TCGTTA
AGACACGTTG	CCGTGT	ACGATA	GCTAGT	ACGAGT
CGTTTCGA	CGTTTGAC	ACGAAA	GTAAGA	TCGTGA
TGACGTGG	TCGAAT	TCGATA	TAGGCT	CCGTTT

Table S4. Candidate accepters and donors(not farther than 10 genes in the same chromosom) of the recruited motifs, along with brief description of accepters.

Accepter	Donors	Motif	Description of Accepter
OS04G065830<br>0	OS04G0658000<br>OS04G0658100<br>OS04G0659900	ACCCGTTTG<br>G	Similar to Ribulose bisphosphate carboxylase/oxygenase activase, chloroplast precursor (RuBisCO activase).
OS08G056210<br>0	OS08G0562700	TAACGCTA	Similar to *Sorghum* chloroplast CM3 malate dehydrogenase (NADP).
OS07G057210<br>0	OS07G0570500<br>OS07G0573800	GTCGAATAA<br>G	Similar to amine oxidase like protein (EC 1.4.3.6) (Copper amine oxidase).
OS07G057210<br>0	OS07G0570500<br>OS07G0571900<br>OS07G0572000<br>OS07G0573100<br>OS07G0571500<br>OS07G0573600	AGATCCGTG<br>T	Similar to amine oxidase like protein (EC 1.4.3.6) (Copper amine oxidase).
OS03G026730<br>0	OS03G0267700	TAACGCTA	Similar to chloroplast fructose-1, 6-bisphosphatase.
OS07G057210<br>0	OS07G0573100<br>OS07G0571900<br>OS07G0570500	AGACACGT<br>TG	Similar to amine oxidase like protein (EC 1.4.3.6) (Copper amine oxidase).

	OS07G0572000<br>OS07G0572300<br>OS07G0571500<br>OS07G0573900		
OS03G026730<br>0	OS03G0266700<br>OS03G0267700<br>OS03G0265200	ATTCGATAC<br>G	Similar to chloroplast fructose-1,<br>6-bisphosphatase.
OS03G086210<br>0	OS03G0860900<br>OS03G0861900<br>OS03G0859500	AGATCCGTG<br>T	Conserved hypothetical protein.
OS10G057880<br>0	OS10G0577800<br>OS10G0580200	ACGTGCGA<br>GT	LrgB-like protein family protein.
OS02G020810<br>0	OS02G0206700	GTCGAATAA<br>G	Similar to plastidic<br>ATP/ADP-transporter.
OS02G062920<br>0	OS02G0631200	TAACGCTA	Similar to HvPIP2%3B1 protein.
OS02G062920<br>0	OS02G0631601	ACGTGCGA<br>GT	Similar to HvPIP2%3B1 protein.
OS03G026730<br>0	OS03G0267700<br>OS03G0267800<br>OS03G0265900	CGTTTACG	Similar to chloroplast fructose-1,<br>6-bisphosphatase.
OS03G086210<br>0	OS03G0859500	CGTTTACG	Conserved hypothetical protein.
OS08G034460<br>0	OS08G0343300	ATTCGATAC<br>G	Similar to phospho*enol*pyruvate/<br>phosphate translocator.
OS02G023600<br>0	OS02G0236600	GTCGAATAA<br>G<br>TAACGCTA	Similar to aspartate aminotransferase.
OS08G056210<br>0	OS08G0560300	CGTTTACG	Similar to *Sorghum* chloroplast CM3<br>malate dehydrogenase (NADP).
OS10G057880<br>0	OS10G0577800<br>OS10G0580200<br>OS10G0580300	ACCCGTTTG<br>G	LrgB-like protein family protein.
OS06G013380<br>0	OS06G0136800	ATTCGATAC<br>G	Similar to transketolase, chloroplastic.
OS06G066420	OS06G0662000	TGACGTGG	Hypothetical gene.

0			
OS12G0515400	OS12G0516700	AGATCCGTGT	Similar to plastidic 2-oxoglutarate/malate transporter.
OS03G0862100	OS03G0860900<br>OS03G0861800<br>OS03G0859500<br>OS03G0859800	GTCGAATAAG	Conserved hypothetical protein.
OS03G0218400	OS03G0219200	TGACGTGG	Similar to hexose transporter.

Table S5. Brief descriptions of TFs binding sites.

TFBS_ID	TF_name	Description
M00010	Opaque-2	Control the expression of a cytosolic form of pyruvate orthophosphate dikinase-1 (*cyPPDK1*)[1].
M00089	Athb-1	The Athb-1 and -2 HD-Zip domains homodimerize forming complexes of different DNA binding specificities.
M00149	SBF-1	Silencer region of a chalcone synthase promoter contains multiple binding sites for a factor, SBF-1, closely related to GT-1.
M00151	AG	Isolation and characterization of the binding sequences for the product of the *Arabidopsis* floral homeotic gene AGAMOUS.
M00182	GBP	G-box binding proteins.
M00218	MYB.Ph3	Dual DNA binding specificity of a petal epidermis-specific MYB transcription factor (MYB.Ph3) from *Petunia hybrid.*
M00219	MYB.Ph3	Dual DNA binding specificity of a petal epidermis-specific MYB transcription factor (MYB.Ph3) from *Petunia hybrid.*
M00226	P	The myb-homologous P gene controls phlobaphene pigmentation in maize floral organs by directly activating a flavonoid biosynthetic gene subset.
M00343	RAV1	RAV1, a novel DNA-binding protein, binds to bipartite recognition sequence through two distinct DNA-binding domains uniquely found in higher plants.
M00344	RAV1	RAV1, a novel DNA-binding protein, binds to bipartite recognition sequence through two distinct DNA-binding domains uniquely found in higher plants.

M00345	GAMYB	Target genes and regulatory domains of the GAMYB transcriptional activator in cereal aleurone.
M00352	Dof1	Diversity and similarity among recognition sequences of Dof transcription factors.
M00353	Dof2	Diversity and similarity among recognition sequences of Dof transcription factors.
M00354	Dof3	Diversity and similarity among recognition sequences of Dof transcription factors.
M00355	PBF	Diversity and similarity among recognition sequences of Dof transcription factors.
M00356	bZIP910	Two bZIP proteins from Antirrhinum flowers preferentially bind a hybrid C-box/G-box motif and help to define a new sub-family of bZIP transcription factors.
M00357	bZIP910	Two bZIP proteins from Antirrhinum flowers preferentially bind a hybrid C-box/G-box motif and help to define a new sub-family of bZIP transcription factors.
M00358	bZIP911	Two bZIP proteins from Antirrhinum flowers preferentially bind a hybrid C-box/G-box motif and help to define a new sub-family of bZIP transcription factors.
M00359	bZIP911	Two bZIP proteins from Antirrhinum flowers preferentially bind a hybrid C-box/G-box motif and help to define a new sub-family of bZIP transcription factors.
M00361	CDC5	A cdc5+ homolog of a higher plant, *Arabidopsis* thaliana.
M00366	EmBP-1	Plant bZIP protein DNA binding specificity.
M00367	HBP-1a	Plant bZIP protein DNA binding specificity.
M00368	CPRF-1	Plant bZIP protein DNA binding specificity.
M00369	TAF-1	Plant bZIP protein DNA binding specificity.
M00370	CPRF-3	Binds to the chalcone synthase (CHS) gene promoter.
M00371	CPRF-2	Plant bZIP protein DNA binding specificity.
M00372	Opaque-2	Plant bZIP protein DNA binding specificity.
M00374	Opaque-2	Plant bZIP protein DNA binding specificity.
M00375	TGA1b	The components of PSI, PET and PSII parts are co-regulated by the TFs of TGA1b.
M00376	TGA1a	The components of PSI, PET and PSII parts are co-regulated by the TFs of TGA1b.

M00392	AGL3	The *Arabidopsis* MADS-box gene AGL3 is widely expressed and encodes a sequence-specific DNA-binding protein.
M00393	AGL3	The *Arabidopsis* MADS-box gene AGL3 is widely expressed and encodes a sequence-specific DNA-binding protein.
M00399	ABF1	DNA binding protein with possible chromatin-reorganizing activity; involved in transcriptional activation, gene silencing, and DNA replication and repair.
M00400	ABF1	ABFs, a family of ABA-responsive element binding factors.
M00401	ABF1	ABFs, a family of ABA-responsive element binding factors.
M00404	MADS-B	Binding matrices compiled for TRANSFAC.
M00408	MADS-A	Binding matrices compiled for TRANSFAC.
M00417	ATHB-9	The *Arabidopsis* Athb-8, -9 and -14 genes are members of a small gene family coding for highly related HD-ZIP proteins.
M00434	PIF3	Direct targeting of light signals to a promoter element-bound transcription factor.
M00435	PIF3	Direct targeting of light signals to a promoter element-bound transcription factor.
M00438	ARF	Binding matrices compiled for TRANSFAC.
M00439	C1	Binding matrices compiled for TRANSFAC.
M00440	CG1	Binding matrices compiled for TRANSFAC.
M00441	GBF	GBF familiy is an important TF regulating photosynthesis with various evidences.
M00442	ABF	Binding matrices compiled for TRANSFAC.
M00443	Opaque-2	Binding matrices compiled for TRANSFAC.
M00479	Alfin1	Alfin1, a novel zinc-finger protein in alfalfa roots that binds to promoter elements in the salt-inducible MsPRP2 gene.
M00501	ANT	DNA binding properties of the *Arabidopsis* floral development protein AINTEGUMENTA.
M00502	TEIL	Cloning and DNA-binding properties of a tobacco Ethylene-Insensitive3 (EIN3) homolog.
M00503	ATHB-5	DNA-binding and dimerization preferences of *Arabidopsis* homeodomain-leucine zipper transcription factors in vitro.
M00506	LIM1	Functional analysis of tobacco LIM protein Ntlim1 involved in lignin biosynthesis.
M00507	TRAB1	Interacts with VP1 and mediates abscisic acid-induced

		transcription[2].
M00635	GT-1	New TRANSFAC MATRIX entries.
M00653	OCSBF-1	OCSBF-1 mRNA with the basal portion of the leaves having 40-fold to 50-fold higher levels of OCSBF-1 transcripts than the apical portion of the leaves[3].
M00654	OSBZ8	TF highly expressed in salt tolerance cultivars[4].
M00660	RITA-1	Its ortholog in *Arabidopsis* plays an important role in regulation of light-induced genes. (UniproKB, http://www.uniprot.org)
M00681	WRKY	New TRANSFAC MATRIX entries.
M00697	HBP-1b	Leucine zipper-type transcription factors of wheat. HBP-1b binds to the hexamer motif in the promoter of the 35S RNA gene of cauliflower mosaic virus[5].
M00700	ROM	Related to seed storage[6].
M00702	SPF1	New TRANSFAC MATRIX entries.
M00735	ZAP1	Characterization of a zinc-dependent transcriptional activator from *Arabidopsis.*
M00788	EmBP-1b	Can activate transcription from a truncated promoter containing a pentamer of the Opaque-2 site in yeast cells.
M00798	MYBAS1	Novel anther-specific myb genes from tobacco as putative regulators of phenylalanine ammonia-lyase expression.
M00819	Knox3	May play a role in meristem formation and/or maintenance.
M00820	HAHB-4	A monomer-dimer equilibrium modulates the interaction of the sunflower homeodomain leucine-zipper protein Hahb-4 with DNA.
M00936	HBP-1a	Leucine zipper-type transcription factors of wheat.
M00937	TGA1a	New TRANSFAC MATRIX entries.
M00942	CPRF-1	*Member of G-box binding protein;* Regulate light-induced genes.
M00943	TAF-1	Homologs of *Arabidopsis* GBF3, binds to a G-box-related element, also binds to the HEX-motif of wheat histone H3 promoter.
M00944	CPRF-3	New TRANSFAC MATRIX entries.
M00945	CPRF-2	Member of G-box binding protein; Regulate light-induced genes.
M00946	TGA1b	New TRANSFAC MATRIX entries.
M00948	PCF2	DNA binding and dimerization specificity and potential targets for the TCP protein family.

M00949	AGL15	Binding site selection for the plant MADS domain protein AGL15: an in vitro and in vivo study.
M00950	AG	Binding site selection for the plant MADS domain protein AGL15: an in vitro and in vivo study.
M00952	PCF5	DNA binding and dimerization specificity and potential targets for the TCP protein family.
M00958	ABI4	Maize ABI4 binds coupling element1 in abscisic acid and sugar response genes.
M00968	AtMYB-77	More than 80R2R3-MYB regulatory genes in the genome of *Arabidopsis* thaliana.
M00969	AtMYB-15	More than 80R2R3-MYB regulatory genes in the genome of *Arabidopsis* thaliana.
M00970	AtMYB-84	More than 80R2R3-MYB regulatory genes in the genome of *Arabidopsis* thaliana.
M01006	AGP1	Transcriptional activation mediated by binding of a plant GATA-type zinc finger protein AGP1 to the AG-motif (AGATCCAA) of the wound-inducible Myb gene NtMyb2.
M01015	PEND	DNA-binding specificity and dimerization of the DNA-binding domain of the PEND protein in the chloroplast envelope membrane.
M01021	ID1	The maize ID1 flowering time regulator is a zinc finger protein with novel DNA binding properties.
M01050	ARR10	Functions as a response regulator involved in His-to-Asp phosphorelay signal transduction system.
M01052	MYB80	A CELD-fusion method for rapid determination of the DNA-binding sequence specificity of novel plant DNA-binding proteins.
M01054	bHLH66	A CELD-fusion method for rapid determination of the DNA-binding sequence specificity of novel plant DNA-binding proteins.
M01055	NAC69-1	A CELD-fusion method for rapid determination of the DNA-binding sequence specificity of novel plant DNA-binding proteins.
M01057	ERF2	A modified sensor chip for surface plasmon resonance enables a rapid determination of sequence specificity of DNA-binding proteins.
M01059	AGL1	DNA binding properties of two *Arabidopsis* MADS domain proteins: binding consensus and dimer formation.

M01060	AGL1	DNA binding properties of two *Arabidopsis* MADS domain proteins: binding consensus and dimer formation.
M01061	AGL2	DNA binding properties of two *Arabidopsis* MADS domain proteins: binding consensus and dimer formation.
M01062	AGL2	DNA binding properties of two *Arabidopsis* MADS domain proteins: binding consensus and dimer formation.
M01063	AG	DNA binding properties of two *Arabidopsis* MADS domain proteins: binding consensus and dimer formation.
M01064	AGL3	DNA binding properties of two *Arabidopsis* MADS domain proteins: binding consensus and dimer formation.
M01065	ABZ1	Para-aminobenzoate (PABA) synthase.
M01114	E2F	New matrix entries.
M01126	BPC1	New matrix entries.
M01128	SED	New matrix entries.
M01130	PBF	New matrix entries.
M01133	AG	New matrix entries.
M01135	GAMYB	New matrix entries.
M01136	Dof	New matrix entries.
M01156	BZR1	Plasma membrane transporter of the major facilitator superfamily.
M01161	ASR-1	The water- and salt-stress-regulated Asr1 (abscisic acid stress ripening) gene encodes a zinc-dependent DNA-binding protein.
M01164	SQUA	DNA binding and dimerisation determinants of Antirrhinum majus MADS-box transcription factors.
M01179	CBT	Isolation of a calmodulin-binding transcription factor from rice (*Oryza sativa* L.).
M01180	SPL14	New matrix entries.
M01186	STF1	STF1 Can Replace HY5 in Photomorp- hogenesis and Hormone Signaling[7].
M01187	STF1	New matrix entries.
M01188	CBNAC	Identification of a calmodulin-binding NAC protein as a transcriptional repressor in *Arabidopsis*.
M01189	CBNAC	Identification of a calmodulin-binding NAC protein as a transcriptional repressor in *Arabidopsis*.
M01191	HDG7	Characterization of the class IV homeodomain-Leucine Zipper gene family in *Arabidopsis*.

M01192	HDG9	Characterization of the class IV homeodomain-Leucine Zipper gene family in *Arabidopsis*.
M01193	ML1	Characterization of the class IV homeodomain-Leucine Zipper gene family in *Arabidopsis*.
M01194	PDF2	Characterization of the class IV homeodomain-Leucine Zipper gene family in *Arabidopsis*.
M01581	AGL15	Global Identification of Targets of the *Arabidopsis* MADS Domain Protein AGAMOUS-Like15.
M01582	AGL9	Target genes of the MADS transcription factor SEPALLATA3: integration of developmental and hormonal pathways in the *Arabidopsis* flower.
M01583	GL1	A systems approach reveals regulatory circuitry for *Arabidopsis* trichome initiation by the GL3 and GL1 selectors.
M01584	HY5	Analysis of transcription factor HY5 genomic binding sites revealed its hierarchical role in light regulation of development.
M01585	PIF1	Genome-wide analysis of genes targeted by phoytochrome interacting factor3-like5 during seed germination in *Arabidopsis*.
M01586	TGA2	Development of *Arabidopsis* whole-genome microarrays and their application to the discovery of binding sites for the TGA2 transcription factor in salicylic acid-treated plants.
M01700	DREB1B	Quadruple 9-mer-based protein binding microarray with DsRed fusion protein.
M01701	NAC6	Quadruple 9-mer-based protein binding microarray with DsRed fusion protein.
M01740	WRKY11	New matrix entries.

Table S6. Experimental validated cells specific motifs. (Potential recruited motifs are colored in red)

genes	regions	sequences	species
C4Pdk	5' flanking	TATAA	maize
C4Pdk	5' flanking	AGTGGAGTCGTGCCGCGTGT	maize
C4Pdk	5' UTR	CCCCCTCTCC	maize
C4Pdk	5' UTR	CACTCGCCACACACA	maize

C4Ppc	5' flanking	CCATCCCTATTT	maize
C4Ppc	5' flanking	CCCTCTCCACATCC	maize
C4Ppc	5' flanking	AAAAAGG	maize
RbcSZm1	5' flanking	CTATATATGCCGTCGGTG	maize
RbcSZm1	5' flanking	CCGGGTGCGGCCAC	maize
RbcSZm1	5' flanking	GCGCGCGT	maize
RbcSZm1	5' flanking	GATAAG	maize
RbcSZm3	5' flanking	CTATATATGCCGTCGGTG	maize
RbcSZm3	5' flanking	CCGGGTGCGGCCAC	maize
RbcSZm3	5' flanking	GATAAG	maize
Cab-m1	5' flanking	AATATTTTTTCT	maize
CabZm1	5' flanking	CGCGCCAAGTGTTCCAG	maize
CabZm5	5' flanking	TATTTA	maize
CabZm5	5' flanking	GATAAG	maize
CabZm5	5' flanking	CCAAT	maize
CabZm5	5' flanking	CACCTCCGGCGA	maize
CabZm5	5' flanking	ATCCGCCCACCT	maize

Table S7. Motif recruitment may be driven by transposable elements.

Motif	TEs	Candidate donors
AAAAAGG	ORSgTEMT01600223, ORSgTEMT01600962 (MITEs)	OS07G0541900
AAAAAGG	ORSiTERT00200148, ORSiTERT00200074 (retrotransposons)	OS07G0556200
AAAAAGG	ORSiTERT00200147 (retrotransposons)	OS07G0564000
AAAAAGG	ORSiTERTOOT00378, ORSiTERT00200082 (retrotransposons)	OS07G0577600
AAAAAGG	ORSgTEMT00101022 (MITEs)	OS07G0583200
AAAAAGG	ORSiTERTOOT00060 (retrotransposons)	OS07G0623100
CCCCCTCTCC	ORSiTETNOOT00105 (transposons)	OS03G0819700
GATAAG	ORSiTERT00200080 (retrotransposons)	OS01G0563500

GATAAG	ORSiTERTOOT00022, ORSgTERTOOT00073, ORSgTERTOOT00096 (retrotransposons)	OS01G0566100
GATAAG	ORSgTEMT00901457 (MITEs)	OS02G0219200
GATAAG	13 retrotransposons	OS02G0264800
GATAAG	ORSgTEMT03800107, ORSgTEMT03800095, ORSgTEMT03800034, ORSgTEMT03800059	OS04G0538800
GATAAG	9 transposons	OS06G0132400
GATAAG	ORSgTEMT00400014 (MITEs)	OS06G0190800
GATAAG	ORSgTEMT03000026, ORSgTEMT03000052 (MITEs)	OS07G0539700
GATAAG	ORSgTEMT03000026, ORSgTEMT03000050, ORSgTEMT01601523, ORSgTEMT03000052 (MITEs)	OS07G0542400
GATAAG	ORSiTERT00200079 (retrotransposons)	OS07G0556200
GATAAG	26 MITEs	OS07G0563350
GATAAG	ORSiTERT00200147 (retrotransposons)	OS07G0564000
GATAAG	23 MITEs	OS07G0602100
GATAAG	ORSiTERTOOT00060 (retrotransposons)	OS07G0623100
GATAAG	ORSiTERTOOT00060 (retrotransposons)	OS10G0542800
GATAAG	ORSgTEMT03800044 (MITEs)	OS10G0563400
GATAAG	ORSiTETNOOT00111, ORSiTETNOOT00133 (transposons)	OS10G0572000
GATAAG	19 MITEs (transposons)	OS10G0572300
GATAAG	ORSiTETNOOT00123, ORSiTETNOOT00106 (transposons)	OS11G0132501
GATAAG	ORSiTETNOOT00119 (transposons)	OS11G0132700
GATAAG	ORSgTEMT00100550, ORSgTEMT00100547 (MITEs)	OS11G0150100
GATAAG	27 MITEs	OS11G0160700
GATAAG	11 MITEs	OS11G0180300
GATAAG	ORSiTEMT01900003, ORSgTEMT01900052, ORSgTEMT01900021 (MITEs)	OS11G0182500
GATAAG	5 retrotransposons	OS11G0202000
GATAAG	50 retrotransposons	OS11G0209200

GATAAG	62 retrotransposons	OS11G0219000
GCGCGCGT	ORSiTETNOOT00104 (transposons)	OS02G0264700

Table S8. Candidate accepters and donors(not farther than 10 genes in the same chromosom) of the recruited motifs(experimental validated cells specific motifs), along with brief description of accepters.

Accepter	Donors	Motif	Description of Accepter
OS07G0572100	OS07G0570500, OS07G0572300, OS07G0573900, OS07G0573100	GTCGAATAAG	Similar to amine oxidase like protein (EC 1.4.3.6) (Copper amine oxidase).
OS07G0572100	OS07G0570500, OS07G0573800, OS07G0573900, OS07G0573100, OS07G0571500, OS07G0573600	GATAAG	Similar to amine oxidase like protein (EC 1.4.3.6) (Copper amine oxidase).
OS10G0578800	OS10G0577800, OS10G0580200, OS10G0577000	GATAAG	LrgB-like protein family protein.
OS02G0208100	OS02G0205400	GCGCGCGT	Similar to plastidic ATP/ADP-transporter.
OS02G0236000	OS02G0236600	AAAAAGG	Similar to Aspartate aminotransferase
OS11G0171300	OS11G0169900, OS11G0170400, OS11G0168600, OS11G0171800, OS11G0172150, OS11G0171500, OS11G0173500	GATAAG	Fructose-bisphosphate aldolase, chloroplast precursor (EC 4.1.2.13) (ALDP).
OS06G0133800	OS06G0136800, OS06G0135000, OS06G0132400	GATAAG	Similar to transketolase, chloroplastic.
OS01G0639900	OS01G0641700	GATAAG	Non-protein coding transcript.

Table S9. Potential recruited cells specific motifs (experimental validated) have higher binding affinity with TFs compared to random binding.

TFBS_ID	TFname	Relative MR	TFBS_ID	TF_Name	Relative MR
M01136	Dof	50	M00404	MADS-B	38.46154
M01130	PBF	20	M01164	SQUA	19.60784
M01189	CBNAC	7.194245	M01161	ASR-1	2.061856
M00354	Dof3	19.60784	M01133	AG	18.18182

Table S10. List of C_4 genes.

ID	Category
GRMZM2G109708	C4.ME.Carbonic Anhydrase
GRMZM2G348512	C4.ME.Carbonic Anhydrase
GRMZM2G094165	C4.ME.Carbonic Anhydrase
GRMZM2G414528	C4.ME.Carbonic Anhydrase
GRMZM2G121878	C4.ME.Carbonic Anhydrase
GRMZM2G305851	C4.ME.Envelope protein
GRMZM2G174107	C4.ME. phosphate/phospho*enol*pyruvate translocator (PPT)
GRMZM2G127591	C4.ME. glucose-6-phosphate/phosphate translocator (GPT)
GRMZM2G092678	C4.ME. triose phosphate/phosphate translocator (TPT)
AC208897.3_FG004	C4. ME. Sugar transporter
GRMZM2G004694	C4. ME. Sugar transporter
GRMZM2G012923	C4. ME. Sugar transporter
GRMZM2G442523	C4. ME. Sugar transporter
GRMZM2G081192	C4.ME.PIP
GRMZM2G154628	C4.ME.PIP
GRMZM2G174807	C4.ME.PIP
GRMZM2G083841	C4.ME.Phosphoenopyruvate Carboxylase
GRMZM2G469150	C4.ME.Aspartate Aminotransferase
GRMZM2G359038	C4.ME.ATP/ADP translocator

GRMZM2G011507	C4.ME.Pyruvate Orthophosphodikinase
GRMZM2G002807	C4.ME.Triosephosphate isomerase
GRMZM2G007263	C4.ME.Glyceraldehyde-3-phosphate dehydrogenase (GAPB)
GRMZM2G089136	C4.ME.Phosphoglycerate kinase (PGK)
GRMZM2G040933	C4.ME.dicarboxylate/tricarboxylate carrier (DCT1)
GRMZM2G383088	C4.ME.dicarboxylate/tricarboxylate carrier (OMT)
GRMZM2G129513	C4.ME. NADP-MDH
GRMZM2G001696	C4.BS.Phosphoenolpyruvate Carboxykinase
GRMZM2G013900	C4.BS.Phosphoenolpyruvate Carboxykinase
GRMZM2G039723	C4.BS.Glyceraldehyde-3-phosphate dehydrogenase (GAPA)
GRMZM2G337113	C4.BS.Glyceraldehyde-3-phosphate dehydrogenase (GAPA)
GRMZM2G018177	C4.BS.TPI
GRMZM2G030784	C4.BS.TPI
GRMZM2G083016	C4.BS.Phosphoglycerate kinase (PGK)
GRMZM2G156356	C4.BS.Maltose exporter (MEX1)
GRMZM2G071423	C4. BS. Envelope protein (MEP1)
GRMZM2G077222	C4. BS. Envelope protein (MEP2)
GRMZM2G138258	C4. BS. Envelope protein (MEP3)
GRMZM2G478212	C4. BS. Envelope protein
GRMZM2G009223	C4.BS. glucose-6-phosphate/phosphate translocator (GPT)
GRMZM2G125850	C4.BS. glucose-6-phosphate/phosphate translocator (GPT)
GRMZM2G042146	C4.BS. dicarboxylate/tricarboxylate carrier (DTC)
GRMZM2G051630	C4.BS. dicarboxylate/tricarboxylate carrier (DTC)
GRMZM2G086258	C4.BS. dicarboxylate/tricarboxylate carrier (DTC)
GRMZM2G134544	C4.BS. dicarboxylate/tricarboxylate carrier (DCT2)
GRMZM2G034302	C4. BS. sucrose transporter
GRMZM2G096683	C4. BS. sucrose transporter
GRMZM2G307561	C4. BS. sucrose transporter
GRMZM2G088196	C4.BS. Sugar transporter
GRMZM2G153920	C4.BS. Sugar transporter
GRMZM2G160069	C4.BS. Sugar transporter

GRMZM2G374812	C4.BS. Sugar transporter
GRMZM2G066413	C4.BS. triose phosphate/phosphate translocator (TPT)
GRMZM2G026807	C4.BS.RPE
GRMZM2G178960	C4.BS.RPE
GRMZM2G306732	C4..BS. FBP
GRMZM2G095287	C4..BS. FBP
GRMZM2G081843	C4.BS.PIP
GRMZM2G392975	C4.BS.PIP
GRMZM2G047368	C4.BS.PIP
GRMZM2G092125	C4.BS.PIP
GRMZM2G178693	C4.BS.PIP
GRMZM2G085019	C4.BS.NADP-malic enzyme
GRMZM2G122479	C4.BS chl cytosol.NADP-malic enzyme
GRMZM2G039345	C4.BS.Rubisco activase
GRMZM2G162200	C4.BS.Rubisco activase
GRMZM2G162282	C4.BS.Rubisco activase
GRMZM2G098520	C4.BS.Rubisco small subunit-4 (RBCS)
GRMZM2G113033	C4.BS.Rubisco small subunit-4 (RBCS)
GRMZM2G026024	C4.BS.Phosphoribulokinase(PRK)
GRMZM2G162529	C4.BS.Phosphoribulokinase(PRK)
GRMZM2G166424	C4.BS.Ribose-5-phosphate isomerase
GRMZM2G104070	C4.BS.Ribose-5-phosphate isomerase
GRMZM2G033208	C4.BS.Transketolase (TKL)
AC147602.5_FG003	C4.BS.Seduheptulose bisphosphatase
GRMZM2G046284	C4.BS.Fructose-bisphosphate aldolase
GRMZM2G155253	C4.BS.Fructose-bisphosphate aldolase
GRMZM2G146677	aspartate aminotransferase, mitochondrial precursor
GRMZM2G463280	C4. ME. PRK

[1] Maddaloni, M., Donini, G., Balconi, C., Rizzi, E., Gallusci, P., Forlani, F., Lohmer, S., Thompson, R., Salamini, F., and Motto, M. The transcriptional activator Opaque-2

controls the expression of a cytosolic form of pyruvate orthophosphate dikinase-1 in maize endosperms. Molecular & general genetics 1996, MGG 250: 647-654.

[2] Hobo T., Kowyama Y., Hattori T. A bZIP factor, TRAB1, interacts with VP1 and mediates abscisic acid-induced transcription. Proc. Natl. Acad. Sci. U.S.A. 1999, 96:15348-15353.

[3] Singh, Karambir, et al. OCSBF-1, a maize ocs enhancer binding factor: isolation and expression during development. The Plant Cell 1990, 29: 891-903.

[4] Mukherjee K, Choudhury A R, Gupta B, et al. An ABRE-binding factor, OSBZ8, is highly expressed in salt tolerant cultivars than in salt sensitive cultivars of indica rice. BMC plant biology 2006, 6(1): 18.

[5] Tabata T, Nakayama T, Mikami K, Iwabuchi M. HBP-1a and HBP-1b: leucine zipper-type transcription factors of wheat. EMBO J. 1991, 10:1459–1467.

[6] Verdier, J, & Thompson, RD. Transcriptional regulation of storage protein synthesis during dicotyledon seed filling. Plant Cell Physiol 2008, 49(9):1263-1271.

[7] Song, YH, Yoo, CM, Hong, AP, Kim, SH, Jeong, HJ, Shin, SY, & Hong, JC. DNA-binding study identifies C-box and hybrid C/G-box or C/A-box moti fs as high-affinity binding sites for STF1 and LONG HYPOCOTYL5 proteins. Plant physiology 2008, 146(4):1862-1877.
