# Supplementary material for: Evidence for the role of transposons in the recruitment of cis-regulatory motifs during the evolution of C4 photosynthesis
Source: BMC Genomics. 2016 Mar 8;17:201. doi: 10.1186/s12864-016-2519-3 (PMC4782515; doi:10.1186/s12864-016-2519-3)
Supplement: Additional file 6: — Network-derived motifs with the probability of ‘A,’ ‘T,’ ‘C,’ ‘G’ in each site. (PDF 708 kb) [file 12864_2016_2519_MOESM6_ESM.pdf]

Additional file 6: Network derived motifs with probabilities of 'A', 'C', 'G', 'T' in each site

MOTIF ACGTACGGTA

0.7503	0.0878	0.07855	0.08335
0.09483	0.6982	0.08994	0.1171
0.101	0.07443	0.7498	0.07485
0.08912	0.09534	0.1	0.7155
0.7749	0.07155	0.0654	0.08811
0.08125	0.7417	0.08353	0.09356
0.108	0.08182	0.7115	0.09873
0.08638	0.08177	0.7425	0.08937
0.1	0.1022	0.08777	0.7101
0.68	0.116	0.1067	0.0973

MOTIF AACGCACGGG

0.7056	0.1134	0.1067	0.07429
0.7562	0.08399	0.1015	0.05827
0.1086	0.6995	0.1035	0.08838
0.09396	0.08665	0.7561	0.06332
0.08911	0.7496	0.1004	0.06091
0.8024	0.05454	0.08105	0.06203
0.09447	0.7497	0.07458	0.08128
0.09268	0.09314	0.7445	0.06964
0.09971	0.09742	0.7253	0.07755
0.1039	0.1002	0.7182	0.07772

MOTIF CGTACT

0.01891	0.8662	0.04421	0.07071
0.05308	0.05187	0.8526	0.04248
0.04045	0.03497	0.05215	0.8724
0.8507	0.04408	0.06476	0.0405
0.02757	0.9007	0.03927	0.03242
0.04648	0.04988	0.01957	0.8841

MOTIF TACCGTAC

0.08527	0.1414	0.06752	0.7058
0.8017	0.06452	0.07367	0.06015
0.06115	0.8145	0.05654	0.06776
0.08939	0.7566	0.06542	0.08855
0.08128	0.06558	0.7866	0.0665
0.06889	0.05308	0.1102	0.7678
0.8013	0.06639	0.07381	0.05847
0.05645	0.8251	0.05536	0.06306

MOTIF AACGCACG

0.7602	0.1069	0.06956	0.06336
0.8067	0.077	0.06686	0.04948
0.08297	0.7618	0.08368	0.0716

0.09222	0.06289	0.7896	0.05533
0.07637	0.7793	0.07672	0.06759
0.8433	0.04314	0.05652	0.05701
0.07557	0.7971	0.05566	0.07172
0.08394	0.05822	0.8038	0.05405

MOTIF AACGCT

0.8303	0.06536	0.03597	0.06833
0.9251	0.04577	0.009617	0.01949
0.02844	0.8792	0.06284	0.02957
0.03501	0.05341	0.8961	0.01551
0.03998	0.8395	0.101	0.01953
0.1028	0.04452	0.04512	0.8076

MOTIF TACGCT

0.03921	0.04837	0.02728	0.8851
0.8692	0.05032	0.05207	0.0284
0.02907	0.8857	0.04497	0.04025
0.03922	0.0598	0.8661	0.0349
0.05169	0.8258	0.06747	0.05503
0.05298	0.04821	0.04693	0.8519

MOTIF CGTATG

0.06109	0.8036	0.05919	0.07616
0.02015	0.04024	0.8967	0.04289
0.02584	0.0572	0.03733	0.8796
0.846	0.03397	0.06347	0.05651
0.02786	0.02412	0.04041	0.9076
0.0382	0.03212	0.8924	0.03731

MOTIF TACGGA

0.06284	0.05544	0.05587	0.8258
0.9071	0.01388	0.03907	0.03994
0.04384	0.8568	0.04811	0.05125
0.03754	0.05269	0.8727	0.0371
0.05337	0.04583	0.8761	0.02467
0.8582	0.04638	0.04403	0.05139

MOTIF CGTTAG

0.05184	0.8716	0.04322	0.03337
0.02608	0.05103	0.904	0.01888
0.02327	0.04323	0.06247	0.871
0.04005	0.05157	0.05426	0.8541
0.7615	0.07601	0.0814	0.08108
0.02522	0.04838	0.8963	0.03013

MOTIF GCGGTA

0.06556	0.05662	0.8155	0.06237
0.0553	0.8407	0.03765	0.0664
0.07434	0.06081	0.8148	0.05003

0.01772	0.03142	0.9205	0.03035
0.03225	0.04567	0.0375	0.8846
0.866	0.03517	0.05835	0.04048

MOTIF TAGTACGC

0.07966	0.08289	0.06028	0.7772
0.7578	0.0757	0.08537	0.08116
0.04891	0.07352	0.8363	0.04132
0.0464	0.08475	0.06233	0.8065
0.8315	0.04724	0.06568	0.0556
0.06635	0.7816	0.0661	0.08598
0.1036	0.06872	0.7511	0.07653
0.08034	0.7131	0.08315	0.1234

MOTIF CGCAAT

0.0506	0.8556	0.03065	0.06316
0.03069	0.01727	0.9439	0.008137
0.04857	0.7976	0.05723	0.09659
0.873	0.04086	0.03542	0.05071
0.7625	0.1162	0.06261	0.05866
0.04965	0.08488	0.1244	0.741

MOTIF TACGTC

0.03089	0.0516	0.01084	0.9067
0.8261	0.06222	0.04979	0.06188
0.02752	0.9088	0.03234	0.03136
0.04247	0.04874	0.8575	0.05125
0.05335	0.04026	0.05102	0.8554
0.02652	0.8489	0.07514	0.04949

MOTIF GTCGTA

0.04432	0.05739	0.8415	0.05683
0.04196	0.05156	0.04089	0.8656
0.1205	0.7705	0.05623	0.05277
0.02168	0.04717	0.9106	0.02057
0.01705	0.02922	0.04307	0.9107
0.869	0.03895	0.05476	0.03726

MOTIF CGCATA

0.05194	0.8243	0.02825	0.09555
0.0813	0.04894	0.8329	0.03685
0.02194	0.8642	0.04122	0.07268
0.881	0.03916	0.06054	0.01931
0.03169	0.09644	0.03529	0.8366
0.8159	0.0387	0.1072	0.03814

MOTIF CTATCG

0.0446	0.8405	0.07616	0.03873
0.0193	0.0427	0.02978	0.9082
0.8415	0.03304	0.04037	0.0851

0.0963 0.1211 0.08453 0.6981
0.008178 0.8908 0.07481 0.02625
0.02476 0.03488 0.9009 0.03947

MOTIF CGTGTA
0.03986 0.8485 0.03932 0.07227
0.08416 0.04701 0.8167 0.05209
0.03434 0.04788 0.05835 0.8594
0.05754 0.02002 0.896 0.02648
0.0216 0.09407 0.03535 0.849
0.8191 0.04496 0.0879 0.04809

MOTIF ATTACG
0.8618 0.04097 0.04597 0.05128
0.05949 0.07222 0.08251 0.7858
0.03368 0.02987 0.02217 0.9143
0.9172 0.0319 0.02459 0.02629
0.02757 0.8875 0.0447 0.0402
0.05373 0.04956 0.8386 0.0581

MOTIF GCTGTA
0.08066 0.05263 0.8082 0.05852
0.05812 0.8226 0.04524 0.074
0.08374 0.06842 0.05487 0.793
0.027 0.02911 0.9148 0.02908
0.01801 0.04697 0.02719 0.9078
0.9035 0.02051 0.05241 0.02362

MOTIF GACGTT
0.01812 0.07622 0.8261 0.07955
0.8012 0.0895 0.04759 0.06174
0.03157 0.9262 0.02601 0.01618
0.01188 0.04147 0.9298 0.01682
0.0331 0.03829 0.1061 0.8225
0.07015 0.01647 0.08591 0.8275

MOTIF CAGTACGT
0.078 0.7491 0.08063 0.09223
0.7766 0.07618 0.06545 0.08177
0.0527 0.06514 0.8248 0.05739
0.05593 0.07533 0.05707 0.8117
0.7668 0.08062 0.07708 0.07554
0.05907 0.797 0.06838 0.07553
0.08993 0.06417 0.7855 0.06036
0.07271 0.06018 0.07254 0.7946

MOTIF ACGTACAG
0.8059 0.07695 0.05384 0.06329
0.08187 0.7975 0.05878 0.06185
0.08015 0.05753 0.8118 0.05054

0.07463	0.09199	0.08955	0.7438
0.8467	0.04935	0.064	0.03991
0.05461	0.8461	0.04632	0.05294
0.7905	0.06394	0.07175	0.0738
0.09176	0.08134	0.7385	0.0884

MOTIF GATCCCGGAT

0.06757	0.1036	0.7647	0.06416
0.7463	0.09639	0.09139	0.06589
0.0858	0.06027	0.1249	0.729
0.07467	0.7062	0.09477	0.1244
0.09293	0.6976	0.08439	0.1251
0.1215	0.6511	0.09642	0.1309
0.1383	0.08772	0.6672	0.1068
0.1003	0.08491	0.7552	0.05965
0.7668	0.07993	0.0876	0.06568
0.0848	0.09622	0.107	0.712

MOTIF GTGCGTTACT

0.09861	0.07705	0.7372	0.0871
0.06126	0.06639	0.05897	0.8134
0.07573	0.08361	0.7464	0.09423
0.0987	0.6678	0.08226	0.1512
0.1277	0.1008	0.6297	0.1418
0.06691	0.08927	0.09628	0.7475
0.08015	0.09861	0.1018	0.7194
0.6417	0.1013	0.1269	0.13
0.09192	0.7216	0.0971	0.08933
0.1059	0.07867	0.07384	0.7416

MOTIF TAGTACGCAT

0.08862	0.09365	0.1068	0.711
0.7432	0.06958	0.07626	0.111
0.09082	0.08293	0.7372	0.08908
0.109	0.1018	0.07199	0.7172
0.7433	0.07446	0.09406	0.08817
0.1087	0.6238	0.1163	0.1511
0.1435	0.1043	0.628	0.1242
0.08983	0.7378	0.07469	0.09766
0.7806	0.06612	0.08047	0.07276
0.06602	0.08044	0.0576	0.7959

MOTIF ACGACTGGAC

0.6912	0.1027	0.1	0.1061
0.1131	0.6936	0.1174	0.07588
0.1125	0.1134	0.6887	0.0853
0.7117	0.0961	0.09402	0.09822
0.1084	0.7147	0.09886	0.078
0.09637	0.09678	0.08871	0.7181
0.07525	0.0997	0.7395	0.08558

0.09795 0.1076 0.7243 0.0701
0.7746 0.071 0.08241 0.07201
0.09794 0.6731 0.1251 0.1039

MOTIF GTATACGC
0.08548 0.0502 0.7744 0.08995
0.06623 0.07218 0.07894 0.7826
0.7601 0.07718 0.08552 0.07724
0.05822 0.1049 0.08542 0.7515
0.7198 0.08735 0.1187 0.07413
0.07098 0.7331 0.08828 0.1077
0.08956 0.0655 0.7701 0.07487
0.07672 0.7596 0.06913 0.09457

MOTIF CCTGTACG
0.08409 0.7891 0.06536 0.06147
0.09334 0.7615 0.05257 0.09261
0.06209 0.05966 0.05623 0.822
0.0376 0.07553 0.8268 0.06007
0.06137 0.09621 0.08555 0.7569
0.7226 0.1129 0.08728 0.07727
0.07384 0.7636 0.06886 0.09372
0.112 0.06367 0.7454 0.07893

MOTIF AGACTGGA
0.7476 0.09116 0.09448 0.06679
0.09989 0.07739 0.7438 0.07894
0.7585 0.08451 0.08271 0.07431
0.09433 0.7579 0.08055 0.06726
0.07213 0.07606 0.07583 0.776
0.06433 0.05408 0.8212 0.06038
0.08141 0.08408 0.7812 0.05328
0.8151 0.06171 0.05973 0.06349

MOTIF ACGCAT
0.859 0.04763 0.05214 0.04122
0.08844 0.8087 0.06603 0.03684
0.09967 0.05735 0.7823 0.06064
0.04361 0.871 0.04977 0.03558
0.9018 0.04733 0.01453 0.03637
0.04381 0.0636 0.03995 0.8526

MOTIF TAACGCAC
0.08619 0.1012 0.09216 0.7205
0.7325 0.1142 0.08765 0.06571
0.7748 0.08444 0.08611 0.05461
0.1067 0.7026 0.08027 0.1104
0.1205 0.07163 0.7256 0.08232
0.0654 0.8299 0.05357 0.05112
0.8522 0.04472 0.05358 0.04954

0.06477	0.7957	0.04878	0.09077

MOTIF CGACTGGA

0.1159	0.7089	0.1027	0.07248
0.0886	0.08723	0.7431	0.08106
0.7506	0.08366	0.08648	0.07928
0.08825	0.7579	0.08452	0.06937
0.07607	0.08273	0.09198	0.7492
0.06224	0.07818	0.7984	0.06118
0.06631	0.07924	0.8075	0.047
0.8059	0.05614	0.07275	0.06523

MOTIF ACGTCT

0.8151	0.06276	0.05797	0.06421
0.05318	0.8387	0.03942	0.06871
0.06365	0.06733	0.8175	0.05152
0.06444	0.06633	0.0562	0.813
0.05338	0.8361	0.07268	0.03781
0.04902	0.04806	0.05217	0.8508

MOTIF ACGCTA

0.8516	0.05033	0.06107	0.03703
0.04762	0.8195	0.05745	0.07541
0.08439	0.06025	0.7913	0.06401
0.03358	0.8787	0.05092	0.03681
0.061	0.05957	0.06191	0.8175
0.826	0.05841	0.06227	0.05332

MOTIF CCGCATAC

0.1073	0.7233	0.09163	0.07776
0.08439	0.7483	0.05643	0.1108
0.1027	0.07729	0.7497	0.07029
0.06044	0.8078	0.07511	0.05666
0.7822	0.09186	0.07559	0.05038
0.07324	0.1177	0.08751	0.7215
0.7105	0.09091	0.1108	0.08778
0.06171	0.8186	0.05216	0.0675

MOTIF GTAGGC

0.05067	0.04663	0.8528	0.04987
0.07743	0.06929	0.07632	0.777
0.8204	0.05628	0.05941	0.06391
0.04138	0.04494	0.8542	0.05952
0.04972	0.0508	0.8579	0.04157
0.04784	0.8593	0.04779	0.04504

MOTIF ATCCCG

0.8099	0.0801	0.05001	0.06003
0.04048	0.03994	0.05646	0.8631
0.04557	0.8441	0.05626	0.05403

0.04772	0.8518	0.04257	0.0579
0.07174	0.7987	0.06549	0.06402
0.07368	0.06139	0.8002	0.06478

MOTIF TAACGC

0.05016	0.07159	0.06281	0.8154
0.7924	0.08529	0.06111	0.06123
0.8307	0.0642	0.06675	0.03833
0.07068	0.7786	0.06977	0.08091
0.07009	0.057	0.8184	0.05452
0.04172	0.8721	0.04416	0.04202

MOTIF GAACGCTT

0.05547	0.07462	0.8035	0.06636
0.6874	0.1192	0.09924	0.09415
0.7949	0.06586	0.08756	0.05168
0.06131	0.7087	0.09955	0.1304
0.09845	0.0642	0.7653	0.072
0.05417	0.8545	0.05574	0.03557
0.1264	0.07219	0.1038	0.6976
0.06494	0.05827	0.07458	0.8022

MOTIF CATTCG

0.03967	0.8697	0.04462	0.04604
0.8737	0.05475	0.037	0.03458
0.0276	0.069	0.06019	0.8432
0.03021	0.05474	0.06759	0.8474
0.05335	0.8213	0.04735	0.07798
0.08572	0.09727	0.7442	0.07276

MOTIF CCGGAATC

0.07102	0.7935	0.06149	0.07396
0.09326	0.7345	0.07145	0.1008
0.09556	0.08042	0.7269	0.09708
0.07718	0.0814	0.7794	0.06197
0.7601	0.08711	0.08952	0.06331
0.7235	0.1243	0.07813	0.07412
0.06731	0.06873	0.08873	0.7752
0.06915	0.7661	0.0974	0.0674

MOTIF ATACCG

0.8517	0.05785	0.05324	0.03726
0.05683	0.07208	0.06688	0.8042
0.7906	0.07622	0.06785	0.06534
0.03295	0.8739	0.05717	0.03601
0.07597	0.7892	0.04996	0.08486
0.06856	0.07362	0.8056	0.05224

MOTIF GACTGG

0.05678	0.04918	0.8502	0.04382

0.8164	0.05769	0.06422	0.06172
0.07077	0.8208	0.05914	0.04926
0.05576	0.04599	0.05831	0.8399
0.0346	0.0561	0.8553	0.05404
0.04969	0.05545	0.8422	0.05267

MOTIF CGATAG

0.05295	0.7979	0.09747	0.05165
0.06734	0.07347	0.8309	0.02831
0.7919	0.06954	0.07778	0.06076
0.06451	0.1131	0.1085	0.7139
0.8671	0.01755	0.05842	0.05696
0.0342	0.05368	0.8593	0.05279

MOTIF GTAGAC

0.03441	0.06185	0.8759	0.02785
0.07558	0.07706	0.08871	0.7587
0.8798	0.01768	0.0693	0.03322
0.04884	0.06948	0.8625	0.01922
0.82	0.05947	0.07079	0.04971
0.06623	0.8051	0.07015	0.0585

MOTIF TTACGG

0.05367	0.05671	0.06432	0.8253
0.04414	0.06731	0.06115	0.8274
0.7808	0.07662	0.06778	0.07479
0.05886	0.8216	0.07328	0.04624
0.07222	0.04179	0.8119	0.07409
0.0475	0.0627	0.8417	0.04808

MOTIF TGCGGT

0.04591	0.04463	0.04243	0.867
0.04043	0.1258	0.778	0.05576
0.04446	0.823	0.06152	0.07105
0.08738	0.0492	0.7924	0.07097
0.03822	0.05031	0.8514	0.0601
0.05084	0.06335	0.05691	0.8289

MOTIF CCCTGA

0.05256	0.8272	0.0575	0.06274
0.05495	0.8408	0.05407	0.0502
0.04841	0.8466	0.05066	0.05435
0.06404	0.04785	0.0566	0.8315
0.05222	0.0556	0.8459	0.04632
0.8144	0.06187	0.04887	0.0749

MOTIF CTACGT

0.0577	0.8039	0.09227	0.04609
0.03787	0.09111	0.05733	0.8137
0.806	0.06393	0.06453	0.06558

0.04322	0.8447	0.05535	0.05668
0.09556	0.05074	0.8038	0.04989
0.05612	0.05647	0.05545	0.832

MOTIF ACCGCTAAGG

0.7231	0.074	0.1077	0.09519
0.1247	0.7058	0.08633	0.08316
0.1032	0.675	0.1009	0.121
0.1077	0.1017	0.6586	0.1321
0.0894	0.7363	0.1033	0.07098
0.129	0.09994	0.1012	0.6699
0.6993	0.1039	0.1157	0.08106
0.7635	0.08575	0.06604	0.0847
0.1039	0.06478	0.7389	0.09232
0.0916	0.09035	0.7285	0.08951

MOTIF TGTCGACTGG

0.08283	0.0973	0.08528	0.7346
0.06924	0.08563	0.7665	0.07858
0.0684	0.08099	0.0854	0.7652
0.09343	0.7004	0.1063	0.09988
0.09056	0.1104	0.7045	0.0945
0.7486	0.07881	0.08618	0.08642
0.07786	0.667	0.1187	0.1365
0.08407	0.09111	0.07716	0.7477
0.08722	0.0679	0.7162	0.1287
0.0891	0.1168	0.6981	0.09598

MOTIF ATTCCGGCTA

0.7408	0.0797	0.08735	0.09216
0.09576	0.09859	0.08084	0.7248
0.0774	0.05548	0.06263	0.8045
0.09655	0.6956	0.1004	0.1075
0.1159	0.6595	0.1128	0.1118
0.1384	0.09843	0.6329	0.1303
0.1197	0.09337	0.7094	0.07748
0.0772	0.7487	0.07761	0.09646
0.09526	0.1011	0.07331	0.7303
0.6938	0.1135	0.101	0.09165

MOTIF TTTCTACCTT

0.0789	0.09059	0.06657	0.7639
0.07191	0.09339	0.07088	0.7638
0.07115	0.06931	0.07193	0.7876
0.1028	0.6415	0.1131	0.1427
0.09799	0.09774	0.06244	0.7418
0.6737	0.1128	0.07288	0.1406
0.1272	0.6224	0.08942	0.161
0.1255	0.6602	0.05886	0.1555
0.0804	0.07258	0.05353	0.7935

0.0812	0.08215	0.07601	0.7606

MOTIF CCGGAATC

0.06884	0.7795	0.06645	0.08518
0.08606	0.7571	0.06499	0.09188
0.1087	0.09191	0.702	0.09742
0.1075	0.08885	0.7529	0.05075
0.8019	0.08326	0.05491	0.05991
0.7566	0.08496	0.08851	0.06996
0.07509	0.05875	0.06399	0.8022
0.06628	0.7917	0.0782	0.06381

MOTIF AGTCTACG

0.8035	0.05768	0.06309	0.07578
0.07072	0.06416	0.7899	0.07526
0.0739	0.05684	0.06817	0.8011
0.04295	0.8177	0.06335	0.07601
0.06915	0.08824	0.1035	0.7392
0.7525	0.08361	0.05624	0.1076
0.09459	0.7198	0.08092	0.1047
0.11	0.08128	0.7076	0.1011

MOTIF TGAACG

0.04933	0.03703	0.04118	0.8725
0.04875	0.05453	0.853	0.04367
0.8514	0.04138	0.06655	0.04068
0.8506	0.05384	0.03944	0.05613
0.07774	0.7683	0.06917	0.08477
0.06518	0.07812	0.7691	0.08764

MOTIF ATTGCG

0.866	0.04329	0.04557	0.04518
0.04539	0.04877	0.03652	0.8693
0.03724	0.03835	0.0738	0.8506
0.04611	0.06409	0.8209	0.06889
0.07922	0.7636	0.07373	0.08341
0.08404	0.06692	0.7625	0.08654

MOTIF GACGTT

0.01133	0.04226	0.9084	0.03802
0.8151	0.06358	0.04011	0.08121
0.09342	0.7446	0.08749	0.07449
0.0568	0.06574	0.7834	0.09401
0.07677	0.05539	0.04369	0.8241
0.04283	0.02115	0.06109	0.8749

MOTIF CGGAAT

0.07541	0.7728	0.062	0.08982
0.07059	0.05476	0.8082	0.0664
0.06353	0.04718	0.8474	0.04185

0.8699	0.05055	0.03972	0.03983
0.8029	0.07292	0.06393	0.06028
0.04896	0.03774	0.0514	0.8619

MOTIF GCCGTTAA

0.07886	0.07644	0.7706	0.07407
0.07247	0.7748	0.0607	0.09201
0.1062	0.7157	0.07772	0.1004
0.1202	0.08715	0.6847	0.1079
0.07059	0.08397	0.07452	0.7709
0.08388	0.09338	0.0952	0.7275
0.7259	0.09336	0.1023	0.07847
0.8051	0.06563	0.06392	0.06535

MOTIF ATTGCGGC

0.7736	0.07161	0.07765	0.0771
0.07651	0.07896	0.0698	0.7747
0.05451	0.06566	0.08657	0.7933
0.05377	0.08096	0.7723	0.09299
0.09808	0.7225	0.07257	0.1068
0.1006	0.06475	0.7152	0.1195
0.06973	0.05845	0.8016	0.07022
0.08174	0.7796	0.05219	0.08648

MOTIF GTCGTAAC

0.07344	0.07077	0.7658	0.09003
0.05197	0.06799	0.05253	0.8275
0.08995	0.7244	0.09018	0.09545
0.07569	0.0656	0.7664	0.09231
0.1035	0.05602	0.06678	0.7737
0.7527	0.1088	0.06188	0.07657
0.7532	0.07579	0.09314	0.07792
0.07957	0.7677	0.07155	0.08115

MOTIF GGTATC

0.06549	0.04445	0.8124	0.07769
0.01625	0.0473	0.8942	0.0423
0.07807	0.06337	0.05497	0.8036
0.7551	0.06749	0.07245	0.1049
0.05328	0.02004	0.04412	0.8826
0.06612	0.8184	0.05659	0.05885

MOTIF CGCTTA

0.08294	0.7662	0.06996	0.08089
0.07969	0.07342	0.7726	0.0743
0.06077	0.812	0.06952	0.05772
0.03078	0.05475	0.03778	0.8767
0.0513	0.04642	0.05331	0.849
0.807	0.07484	0.06133	0.05688

MOTIF CGTAGA

0.09492	0.7469	0.07491	0.08331
0.05343	0.05936	0.813	0.07424
0.07438	0.03854	0.05536	0.8317
0.8077	0.08068	0.06221	0.04938
0.03871	0.03662	0.8848	0.0399
0.8928	0.04163	0.02912	0.03648

MOTIF ATACCGCT

0.763	0.08364	0.07389	0.07943
0.09974	0.09501	0.07996	0.7253
0.7236	0.07336	0.0914	0.1117
0.08459	0.7588	0.07368	0.08297
0.09728	0.7271	0.0635	0.1122
0.08779	0.09893	0.6956	0.1176
0.08946	0.7615	0.07893	0.07009
0.06241	0.06866	0.0428	0.8261

MOTIF CGCATT

0.0891	0.7476	0.07178	0.09157
0.07669	0.09361	0.7297	0.1
0.07124	0.8079	0.07874	0.04212
0.8444	0.06569	0.03369	0.05621
0.06151	0.04309	0.05717	0.8382
0.03141	0.04452	0.03799	0.8861

MOTIF CAGGTACT

0.0936	0.7261	0.08444	0.09587
0.8321	0.03727	0.05909	0.0715
0.08432	0.0571	0.7464	0.1122
0.08068	0.08304	0.7419	0.09435
0.08634	0.08199	0.08354	0.7481
0.7319	0.08152	0.09381	0.09276
0.07232	0.7942	0.06425	0.06927
0.1146	0.07507	0.06943	0.7409

MOTIF GCGGTTAA

0.07846	0.08049	0.7273	0.1138
0.1006	0.7009	0.09732	0.1011
0.1097	0.08194	0.7063	0.102
0.08395	0.07413	0.7565	0.08545
0.06952	0.09182	0.06058	0.7781
0.08539	0.08012	0.09746	0.737
0.7483	0.07278	0.09588	0.08302
0.7786	0.07631	0.06906	0.076

MOTIF CTTACCGG

0.05954	0.7852	0.06818	0.08709
0.07415	0.05362	0.07093	0.8013
0.07544	0.1145	0.09517	0.7149

0.7402	0.08445	0.07158	0.1037
0.06755	0.775	0.08503	0.07242
0.07511	0.7865	0.05281	0.08553
0.1124	0.06225	0.7354	0.08994
0.07679	0.06227	0.7962	0.06477

MOTIF CGTAAT

0.07018	0.7854	0.05693	0.08753
0.07636	0.08688	0.7414	0.0954
0.07614	0.05653	0.071	0.7963
0.8532	0.05834	0.04994	0.03854
0.8777	0.03819	0.04401	0.04005
0.04534	0.04279	0.02974	0.8821

MOTIF GCGAAT

0.05557	0.06424	0.8035	0.07669
0.1075	0.7344	0.07308	0.08504
0.08075	0.06753	0.7783	0.07339
0.8797	0.03283	0.05254	0.0349
0.8455	0.05186	0.0439	0.0587
0.05301	0.04844	0.06803	0.8305

MOTIF ATTACGGC

0.769	0.06703	0.07896	0.08496
0.08169	0.07187	0.06914	0.7773
0.06502	0.07464	0.1053	0.7551
0.6754	0.1028	0.1042	0.1176
0.1307	0.6754	0.08817	0.1057
0.1072	0.05673	0.7368	0.09921
0.0957	0.07356	0.7517	0.07906
0.07924	0.7676	0.06879	0.08433

MOTIF GATACG

0.04461	0.02748	0.8761	0.05179
0.8492	0.05414	0.05065	0.04598
0.07356	0.06289	0.07992	0.7836
0.8093	0.05534	0.04293	0.09239
0.06925	0.819	0.05749	0.0543
0.09157	0.06617	0.7638	0.07851

MOTIF GACGTTCT

0.0643	0.08171	0.7733	0.08067
0.7462	0.07746	0.07119	0.1051
0.118	0.6667	0.124	0.09128
0.1064	0.1002	0.6543	0.1391
0.09521	0.07754	0.07907	0.7482
0.08259	0.08877	0.06463	0.764
0.06307	0.7681	0.08363	0.08519
0.07687	0.05675	0.0765	0.7899

MOTIF GTCGTA

0.03944	0.05453	0.8596	0.04641
0.03715	0.04276	0.04931	0.8708
0.07078	0.7937	0.05438	0.08111
0.05899	0.04819	0.8204	0.07244
0.05907	0.04578	0.06434	0.8308
0.7546	0.1067	0.05849	0.08016

MOTIF CGTGAA

0.08256	0.7716	0.07853	0.06736
0.08354	0.07083	0.7572	0.08844
0.07037	0.04639	0.0512	0.832
0.0431	0.06327	0.8487	0.04498
0.8414	0.04227	0.07041	0.04589
0.8707	0.04544	0.03733	0.04657

MOTIF GCGATT

0.05554	0.05671	0.8081	0.0796
0.1067	0.7331	0.07365	0.08655
0.09218	0.07947	0.7704	0.05792
0.8579	0.04666	0.04958	0.04582
0.04533	0.04871	0.0412	0.8648
0.03175	0.04864	0.057	0.8626

MOTIF TACGAG

0.05878	0.0438	0.07971	0.8177
0.8377	0.05608	0.03571	0.07047
0.07094	0.8075	0.06859	0.05298
0.09237	0.07788	0.7432	0.08659
0.8706	0.03799	0.04558	0.04581
0.04982	0.05988	0.8469	0.04343

MOTIF CGTTAA

0.09604	0.7623	0.07124	0.07038
0.08065	0.08944	0.7582	0.0717
0.0411	0.04841	0.05528	0.8552
0.04691	0.06948	0.07014	0.8135
0.8196	0.06341	0.0619	0.05508
0.8765	0.04209	0.04156	0.03982

MOTIF CCTGTA

0.05906	0.8259	0.05389	0.06114
0.08239	0.796	0.04313	0.07846
0.04911	0.03767	0.04148	0.8717
0.05041	0.08409	0.7971	0.06845
0.06666	0.05525	0.05173	0.8264
0.7999	0.06064	0.06443	0.07502

MOTIF GGTAGA

0.0792	0.04765	0.8056	0.06753

0.0514	0.05624	0.8466	0.04572
0.07962	0.04704	0.05116	0.8222
0.7943	0.06082	0.07861	0.0663
0.06174	0.05078	0.8361	0.05134
0.8901	0.03183	0.03911	0.03894

MOTIF GGCGACATTC

0.08405	0.08268	0.7254	0.1079
0.09614	0.08125	0.7385	0.08415
0.1146	0.6754	0.09983	0.1102
0.1043	0.1064	0.7184	0.0709
0.739	0.09167	0.08292	0.08638
0.08556	0.7481	0.09544	0.07089
0.722	0.1112	0.07969	0.08714
0.1004	0.08388	0.07218	0.7436
0.06443	0.1117	0.104	0.7198
0.083	0.722	0.1064	0.08866

MOTIF CGTCGCATTA

0.1183	0.6881	0.06734	0.1263
0.1456	0.09928	0.6319	0.1232
0.08743	0.07274	0.06703	0.7728
0.08803	0.7059	0.09685	0.1092
0.1206	0.08344	0.6835	0.1124
0.0883	0.7587	0.07542	0.0776
0.7686	0.09999	0.05631	0.07505
0.08039	0.08892	0.09701	0.7337
0.08188	0.1143	0.1052	0.6986
0.6627	0.1224	0.09984	0.115

MOTIF ATAGCG

0.8418	0.05873	0.05234	0.04717
0.05297	0.04817	0.04516	0.8537
0.813	0.05082	0.08531	0.05088
0.04019	0.03917	0.8658	0.05488
0.07641	0.7943	0.05527	0.07397
0.08967	0.06086	0.7866	0.06283

MOTIF TAACGC

0.04214	0.06168	0.03327	0.8629
0.8011	0.0736	0.08043	0.04487
0.8634	0.04094	0.04954	0.04614
0.07717	0.7675	0.05971	0.09565
0.09248	0.08469	0.7459	0.07695
0.04521	0.8751	0.0376	0.04207

MOTIF ATTGCG

0.8449	0.0487	0.05951	0.04687
0.06142	0.04365	0.03626	0.8587
0.05323	0.05164	0.0854	0.8097

0.0344	0.03999	0.8756	0.05002
0.05317	0.8093	0.06301	0.07453
0.1123	0.0743	0.7347	0.07863

MOTIF TCGCATTA

0.06294	0.05056	0.0441	0.8424
0.09911	0.7009	0.08416	0.1158
0.1162	0.07307	0.7321	0.07862
0.06821	0.8238	0.05451	0.05351
0.8384	0.05564	0.0411	0.06483
0.0639	0.05234	0.05317	0.8306
0.06629	0.09405	0.05875	0.7809
0.7502	0.08022	0.07858	0.09103

MOTIF GTTACGCA

0.06819	0.06285	0.7919	0.07703
0.07382	0.08691	0.05343	0.7858
0.08142	0.08484	0.118	0.7157
0.7457	0.06472	0.1031	0.08653
0.1067	0.6753	0.07695	0.141
0.1176	0.09587	0.6926	0.09386
0.06459	0.8093	0.0588	0.06734
0.8019	0.06237	0.05497	0.08081

MOTIF AATGCG

0.8534	0.04895	0.05549	0.0422
0.874	0.04197	0.03489	0.04917
0.04989	0.03168	0.05067	0.8678
0.03417	0.04593	0.8561	0.06375
0.06815	0.7686	0.07117	0.09209
0.1065	0.05893	0.7537	0.08089

MOTIF TACTCG

0.08033	0.07291	0.07994	0.7668
0.8293	0.06446	0.04518	0.06109
0.0534	0.8271	0.05989	0.0596
0.0363	0.07732	0.03481	0.8516
0.04892	0.845	0.04472	0.06136
0.05996	0.06877	0.789	0.08231

MOTIF TCCGCTAT

0.06162	0.05401	0.07276	0.8116
0.07666	0.7658	0.07133	0.08617
0.09268	0.7234	0.068	0.1159
0.111	0.0907	0.6759	0.1224
0.07445	0.8066	0.05837	0.06055
0.06404	0.1137	0.0538	0.7684
0.7519	0.08265	0.0714	0.09404
0.05731	0.08839	0.06622	0.7881

MOTIF AGACGC

0.8273	0.05935	0.05024	0.06313
0.0331	0.03859	0.8977	0.03066
0.8244	0.06042	0.05259	0.06263
0.09074	0.7708	0.06918	0.06926
0.07398	0.04109	0.8276	0.05731
0.06725	0.8568	0.03995	0.03602

MOTIF TGCGAA

0.05638	0.03868	0.07624	0.8287
0.03501	0.05162	0.8978	0.01557
0.07536	0.7661	0.06316	0.09538
0.1002	0.08226	0.7499	0.06758
0.9057	0.01534	0.0399	0.03902
0.8274	0.0598	0.05243	0.06033

MOTIF CGCATA

0.09721	0.7397	0.07725	0.08582
0.08092	0.06028	0.7932	0.06556
0.05393	0.8671	0.04672	0.03226
0.8808	0.0537	0.03216	0.03333
0.05562	0.05628	0.04925	0.8389
0.8002	0.06779	0.05965	0.07235

MOTIF GGCGATTC

0.07562	0.06549	0.776	0.08287
0.06265	0.06081	0.8134	0.06309
0.078	0.7364	0.0772	0.1084
0.08066	0.06938	0.7842	0.06574
0.7893	0.07857	0.063	0.06912
0.07538	0.08181	0.07535	0.7675
0.0617	0.06793	0.1176	0.7528
0.07026	0.7612	0.07331	0.09527

MOTIF TCTACG

0.05266	0.03747	0.06615	0.8437
0.03947	0.8736	0.03456	0.0524
0.04878	0.05782	0.07708	0.8163
0.7772	0.07993	0.05743	0.08542
0.065	0.7959	0.07127	0.06781
0.06007	0.08215	0.7698	0.08794

MOTIF GTAGCG

0.05386	0.05874	0.8395	0.04786
0.06741	0.06339	0.06433	0.8049
0.7757	0.0672	0.0978	0.05934
0.04017	0.035	0.8926	0.03219
0.06169	0.8329	0.04305	0.06239
0.08754	0.06422	0.7832	0.06505

MOTIF TACGCA

0.05845	0.06581	0.09055	0.7852
0.8528	0.05534	0.02115	0.07068
0.0919	0.735	0.06736	0.1057
0.07017	0.0977	0.7693	0.06286
0.04687	0.9065	0.03527	0.01134
0.851	0.05181	0.03701	0.06014

MOTIF CGTTCT

0.04078	0.8296	0.07474	0.05487
0.08656	0.103	0.7059	0.1045
0.0801	0.07619	0.05609	0.7876
0.04425	0.07514	0.05362	0.827
0.06605	0.7831	0.07622	0.07462
0.0394	0.05547	0.04159	0.8635

MOTIF ACGCTACT

0.7678	0.08665	0.06997	0.07561
0.1033	0.6743	0.08878	0.1336
0.08883	0.06807	0.7583	0.0848
0.06304	0.8122	0.05558	0.06917
0.06774	0.09313	0.07484	0.7643
0.7911	0.05584	0.07537	0.07764
0.07434	0.7766	0.06521	0.08381
0.0907	0.08902	0.05671	0.7636

MOTIF GCCGTA

0.04917	0.03909	0.8775	0.03423
0.03806	0.8458	0.05236	0.06379
0.07944	0.8206	0.04939	0.05053
0.1037	0.05601	0.7688	0.07149
0.07978	0.09509	0.04875	0.7764
0.804	0.07969	0.04965	0.0667

MOTIF ATATCG

0.9021	0.02856	0.03098	0.03839
0.06036	0.05598	0.04433	0.8393
0.8048	0.07624	0.0717	0.04731
0.0383	0.03759	0.05115	0.873
0.06811	0.7822	0.07177	0.07791
0.09192	0.07694	0.7806	0.05057

MOTIF AGCGAT

0.8035	0.05922	0.0712	0.06608
0.05087	0.04382	0.8907	0.01457
0.09278	0.7655	0.06563	0.07606
0.09274	0.07581	0.758	0.07342
0.9109	0.01387	0.03576	0.03948
0.06316	0.07468	0.05804	0.8041

MOTIF CATATCGG

0.07992	0.7644	0.08217	0.07356
0.8641	0.03302	0.04785	0.055
0.08353	0.1176	0.05566	0.7432
0.7715	0.07921	0.08153	0.0678
0.06148	0.04895	0.08746	0.8021
0.06098	0.7421	0.1012	0.09567
0.1128	0.07684	0.746	0.06439
0.09537	0.1176	0.6784	0.1087

MOTIF ATAGCGGT

0.7943	0.06351	0.09113	0.05105
0.06756	0.05505	0.05613	0.8213
0.7777	0.04515	0.1059	0.07126
0.07232	0.06094	0.7805	0.08624
0.1032	0.6871	0.1015	0.1082
0.1218	0.08333	0.7271	0.0678
0.079	0.08629	0.7363	0.09845
0.1249	0.1	0.07482	0.7002

MOTIF CTGGTAACCC

0.08235	0.7488	0.07362	0.09527
0.09544	0.07754	0.05954	0.7675
0.06643	0.07365	0.739	0.121
0.07686	0.1273	0.724	0.07184
0.1075	0.1112	0.07566	0.7056
0.6901	0.1184	0.1084	0.08309
0.682	0.1026	0.1193	0.09609
0.09418	0.7298	0.09251	0.08349
0.07828	0.7637	0.06699	0.09105
0.1143	0.6872	0.08267	0.1158

MOTIF ACTGCAAGCG

0.7041	0.09808	0.1039	0.0939
0.09892	0.6739	0.1051	0.1221
0.08582	0.08991	0.07371	0.7506
0.07949	0.0747	0.7586	0.08719
0.06534	0.7937	0.09537	0.04556
0.7178	0.112	0.08354	0.08665
0.7424	0.08011	0.0963	0.08116
0.08895	0.09886	0.7373	0.07488
0.1077	0.685	0.09737	0.1099
0.1434	0.09036	0.6648	0.1015

MOTIF ATTGCGCGCG

0.6688	0.1095	0.1257	0.09599
0.06446	0.1246	0.12	0.6909
0.04978	0.1033	0.1235	0.7235
0.05549	0.1016	0.7763	0.06669
0.08594	0.7524	0.08492	0.07673

0.09129	0.1045	0.7312	0.07299
0.09151	0.7348	0.08974	0.08395
0.07225	0.07412	0.7786	0.07501
0.07389	0.7269	0.08927	0.1099
0.1041	0.08388	0.7438	0.06825

MOTIF GTTACCAG

0.07797	0.07418	0.7533	0.0946
0.06715	0.1105	0.0801	0.7422
0.06107	0.09143	0.07985	0.7677
0.7561	0.06344	0.08545	0.09498
0.06017	0.7667	0.1071	0.06601
0.09295	0.7953	0.04988	0.06188
0.7997	0.05691	0.05304	0.09038
0.08859	0.06591	0.7596	0.08595

MOTIF CGCGCAAT

0.05571	0.8022	0.06448	0.07757
0.07937	0.05819	0.7999	0.06252
0.07252	0.7759	0.07547	0.07612
0.05896	0.05757	0.8185	0.06501
0.05709	0.8479	0.05635	0.0387
0.8121	0.08834	0.06597	0.03355
0.7806	0.08754	0.07852	0.05334
0.07737	0.09815	0.08959	0.7349

MOTIF TTTCAGGC

0.06615	0.07934	0.06338	0.7911
0.05782	0.07321	0.08056	0.7884
0.06815	0.05388	0.1151	0.7629
0.06639	0.7984	0.0733	0.06186
0.8075	0.05905	0.06569	0.06775
0.09999	0.05892	0.7195	0.1216
0.06993	0.07767	0.7622	0.09019
0.08457	0.7602	0.06217	0.0931

MOTIF GTGCGGAA

0.05608	0.07761	0.794	0.0723
0.0715	0.1217	0.06112	0.7457
0.04315	0.04036	0.8541	0.06235
0.09076	0.7252	0.07698	0.1071
0.07664	0.0698	0.7554	0.09812
0.08374	0.11	0.736	0.07018
0.7237	0.135	0.08302	0.05828
0.7907	0.05468	0.09121	0.06341

MOTIF GGAAGCGT

0.07252	0.06318	0.7934	0.07089
0.05212	0.0881	0.8077	0.05203
0.7751	0.1022	0.06943	0.05331

0.7579	0.07845	0.09772	0.06591
0.06485	0.06147	0.8289	0.04476
0.06609	0.7941	0.07742	0.06238
0.0961	0.07484	0.7338	0.09528
0.1093	0.09114	0.07408	0.7255

MOTIF AGACGT

0.8076	0.06063	0.06614	0.06567
0.04849	0.06268	0.8417	0.04711
0.8035	0.06515	0.07045	0.06091
0.06398	0.8068	0.06087	0.06833
0.05282	0.03396	0.8729	0.0403
0.06731	0.0809	0.0752	0.7766

MOTIF TAACGC

0.06085	0.07278	0.06426	0.8021
0.7778	0.09161	0.07516	0.05543
0.8545	0.05201	0.05225	0.04123
0.06318	0.7912	0.07264	0.07296
0.0827	0.08139	0.7849	0.05101
0.03611	0.8718	0.04643	0.04569

MOTIF GATACG

0.02697	0.03975	0.8958	0.03744
0.8347	0.062	0.05272	0.05054
0.0669	0.07141	0.08069	0.781
0.8148	0.05684	0.07754	0.05078
0.06037	0.8063	0.05529	0.07803
0.06948	0.05268	0.8213	0.0565

MOTIF AGTCGCAC

0.7198	0.1027	0.08123	0.09622
0.06669	0.08855	0.7821	0.06264
0.07056	0.09079	0.09283	0.7458
0.09204	0.6949	0.08822	0.1249
0.08769	0.06615	0.7653	0.08083
0.0617	0.8232	0.05694	0.05816
0.7814	0.07007	0.07218	0.07636
0.05491	0.802	0.08407	0.05897

MOTIF TCGCAC

0.05354	0.05851	0.07734	0.8106
0.06022	0.7915	0.07624	0.07208
0.06581	0.05883	0.8113	0.06406
0.04016	0.8862	0.03847	0.03516
0.8167	0.05876	0.06436	0.06021
0.03204	0.8709	0.05112	0.04594

MOTIF CTGGTA

0.05156	0.8563	0.05022	0.0419

0.05144	0.03479	0.03929	0.8745
0.03834	0.03909	0.8461	0.07642
0.05751	0.06938	0.8284	0.04476
0.06343	0.08224	0.05364	0.8007
0.8325	0.04138	0.0728	0.05335

MOTIF GTACGCTC

0.058	0.0743	0.7884	0.07935
0.06384	0.1796	0.0854	0.6712
0.7348	0.08507	0.1021	0.07805
0.1038	0.6967	0.08162	0.1179
0.07463	0.09537	0.7632	0.06683
0.06155	0.8214	0.07155	0.0455
0.075	0.0596	0.09229	0.7731
0.0566	0.7977	0.07566	0.07004

MOTIF GAACGCTC

0.06631	0.08767	0.7818	0.06419
0.6272	0.1874	0.09798	0.08739
0.7857	0.09181	0.02543	0.09709
0.1091	0.6689	0.1062	0.1158
0.08669	0.08803	0.7491	0.0762
0.04741	0.8701	0.06208	0.02045
0.09243	0.05944	0.08782	0.7603
0.0707	0.769	0.09948	0.06082

MOTIF GGTAAC

0.05548	0.03808	0.8381	0.06836
0.03628	0.06641	0.8856	0.01176
0.07211	0.08205	0.05141	0.7944
0.7788	0.06861	0.08712	0.06548
0.8776	0.01849	0.06136	0.04254
0.06544	0.8372	0.05381	0.04355

MOTIF AAGCGT

0.8185	0.07094	0.07101	0.0396
0.8612	0.01747	0.06181	0.05954
0.03983	0.04939	0.8742	0.03662
0.05047	0.8271	0.05556	0.06692
0.1012	0.06566	0.8105	0.02263
0.07378	0.07543	0.04714	0.8036

MOTIF ATTCGC

0.7876	0.08102	0.0679	0.0635
0.05727	0.06762	0.07697	0.7981
0.04577	0.0784	0.07514	0.8007
0.06168	0.8018	0.04523	0.09129
0.06463	0.06301	0.8085	0.0639
0.0505	0.8648	0.03734	0.04736

MOTIF CTCACATC

0.08076	0.7813	0.0671	0.07088
0.1148	0.072	0.07753	0.7357
0.07092	0.7706	0.08916	0.06932
0.739	0.09624	0.09295	0.07181
0.09432	0.7641	0.06998	0.07161
0.8016	0.08758	0.04894	0.06185
0.07849	0.09751	0.07828	0.7457
0.08273	0.7631	0.08983	0.06431

MOTIF ATGCGAGG

0.7222	0.0984	0.1051	0.07433
0.07034	0.06123	0.1017	0.7668
0.0489	0.06343	0.8197	0.06793
0.07205	0.7569	0.07651	0.09455
0.165	0.09001	0.6713	0.0737
0.7698	0.06766	0.05965	0.1029
0.07019	0.07267	0.7733	0.08379
0.07557	0.08881	0.7637	0.0719

MOTIF ACGCTC

0.7758	0.07703	0.07381	0.0734
0.0634	0.777	0.07187	0.08771
0.05839	0.05202	0.8501	0.03952
0.03991	0.8822	0.04353	0.03436
0.0649	0.04118	0.0685	0.8254
0.03295	0.8684	0.05546	0.04321

MOTIF CCGTAA

0.04312	0.8566	0.05369	0.04657
0.06915	0.7761	0.06501	0.08972
0.05631	0.0666	0.7916	0.08551
0.07127	0.07046	0.09118	0.7671
0.8241	0.06643	0.06737	0.0421
0.8628	0.0512	0.04007	0.04592

MOTIF GTTCCG

0.04087	0.07007	0.8379	0.05111
0.05343	0.08778	0.05429	0.8045
0.04958	0.04359	0.08446	0.8224
0.04637	0.8083	0.08686	0.05843
0.07015	0.8397	0.04354	0.04662
0.08594	0.06627	0.7837	0.06413

MOTIF TACCGG

0.04164	0.1457	0.06054	0.7521
0.7748	0.0526	0.08961	0.08297
0.04061	0.8731	0.05346	0.03281
0.0824	0.8356	0.03458	0.04739
0.1182	0.06133	0.7376	0.08281

0.04422	0.04644	0.8666	0.0427

MOTIF CGGCAT

0.06295	0.7659	0.07604	0.09511
0.0739	0.05374	0.7505	0.1219
0.04095	0.08128	0.8413	0.03644
0.04003	0.8889	0.04165	0.02946
0.8248	0.04591	0.07578	0.05351
0.07262	0.08151	0.0643	0.7816

MOTIF GCGTTC

0.03879	0.04169	0.88	0.03954
0.06088	0.8251	0.05216	0.06186
0.06634	0.08165	0.7759	0.0761
0.05701	0.08037	0.06521	0.7974
0.04457	0.08396	0.09859	0.7729
0.0399	0.8597	0.0545	0.0459

MOTIF GGACGT

0.05362	0.0564	0.829	0.06095
0.04466	0.08852	0.8038	0.06303
0.7994	0.0862	0.05554	0.05887
0.05828	0.7909	0.06927	0.08155
0.04935	0.04612	0.8663	0.03818
0.06324	0.1029	0.05753	0.7764

MOTIF TTGCGT

0.04536	0.06111	0.06372	0.8298
0.03301	0.03418	0.0558	0.877
0.03082	0.04253	0.8883	0.03839
0.04817	0.8629	0.04007	0.04889
0.1439	0.08267	0.6579	0.1155
0.03976	0.07458	0.0501	0.8356

MOTIF TGACGT

0.05264	0.05708	0.06138	0.8289
0.04002	0.04995	0.8686	0.04143
0.8011	0.07473	0.07561	0.04854
0.092	0.7484	0.08326	0.07634
0.08223	0.06624	0.7894	0.06217
0.05399	0.06775	0.06241	0.8159

MOTIF ACGCAT

0.8322	0.04619	0.05968	0.06197
0.0863	0.802	0.06563	0.04605
0.1057	0.06471	0.7765	0.05306
0.03649	0.8904	0.04011	0.03296
0.8848	0.05142	0.01591	0.04789
0.05221	0.07093	0.06745	0.8094

MOTIF TGTGCTACCT

0.07548	0.05764	0.1239	0.743
0.07045	0.1219	0.6877	0.12
0.08613	0.07541	0.07371	0.7647
0.09205	0.08276	0.7199	0.1053
0.08921	0.7408	0.08193	0.08808
0.07455	0.1011	0.05531	0.7691
0.6856	0.09181	0.09906	0.1235
0.101	0.6767	0.1057	0.1166
0.1064	0.717	0.06109	0.1155
0.1069	0.07132	0.08373	0.738

MOTIF AGTACCGCCG

0.6643	0.1114	0.1212	0.1031
0.08008	0.09943	0.7433	0.07718
0.07985	0.1711	0.09536	0.6537
0.7218	0.07215	0.1291	0.07699
0.087	0.7468	0.08947	0.07675
0.07019	0.7758	0.05843	0.0956
0.1474	0.06275	0.7102	0.07969
0.1018	0.7517	0.07481	0.07172
0.09366	0.7754	0.06217	0.06876
0.0968	0.09182	0.7354	0.07598

MOTIF GGTATCGC

0.06975	0.04105	0.7932	0.09596
0.06639	0.08225	0.7765	0.07488
0.06373	0.09366	0.07165	0.771
0.7146	0.07112	0.1284	0.08589
0.06941	0.06964	0.09512	0.7658
0.072	0.7644	0.08309	0.08048
0.09241	0.06095	0.7479	0.09872
0.06655	0.8	0.07863	0.05484

MOTIF TCAGACGTAG

0.08941	0.09441	0.07399	0.7422
0.06965	0.7332	0.08691	0.1103
0.7639	0.06624	0.08344	0.08641
0.1048	0.09178	0.7128	0.09056
0.7505	0.0911	0.08362	0.0748
0.1113	0.6193	0.1218	0.1476
0.1239	0.08299	0.6808	0.1123
0.1313	0.08379	0.09431	0.6906
0.7248	0.09931	0.09257	0.0833
0.08203	0.08467	0.7661	0.06722

MOTIF TATCGC

0.04707	0.07447	0.05845	0.82
0.8376	0.05897	0.06399	0.0394
0.03127	0.04988	0.03782	0.881

0.07161	0.8001	0.05629	0.07202
0.0839	0.05113	0.7896	0.07535
0.04184	0.8826	0.04111	0.03441

MOTIF CGTTGCGA

0.07839	0.7462	0.07202	0.1034
0.08693	0.098	0.7402	0.07485
0.06275	0.07641	0.1201	0.7408
0.07403	0.1025	0.086	0.7375
0.05154	0.06228	0.8352	0.05097
0.04874	0.7845	0.08593	0.08087
0.1049	0.1079	0.6897	0.09745
0.7658	0.08743	0.08679	0.05996

MOTIF TGTATCGC

0.06881	0.04164	0.08851	0.801
0.0858	0.07459	0.7519	0.08768
0.04727	0.0878	0.07276	0.7922
0.764	0.08266	0.07756	0.07583
0.05298	0.07202	0.0841	0.7909
0.08168	0.7499	0.06632	0.1021
0.1005	0.06483	0.7278	0.107
0.05755	0.8202	0.06179	0.06048

MOTIF ATACGCCG

0.6848	0.09997	0.1265	0.08875
0.09087	0.08129	0.1115	0.7163
0.7301	0.09876	0.08034	0.09079
0.03785	0.8122	0.0639	0.08605
0.1055	0.1067	0.7047	0.08308
0.1045	0.7593	0.07997	0.05626
0.05273	0.822	0.06621	0.05906
0.07346	0.05751	0.8198	0.04927

MOTIF GCGATAAC

0.06541	0.09067	0.7733	0.0706
0.08665	0.7562	0.07381	0.08332
0.09406	0.0871	0.7287	0.09016
0.8332	0.07073	0.06226	0.03384
0.08435	0.1021	0.08621	0.7273
0.7612	0.06903	0.1085	0.06125
0.7503	0.09641	0.08339	0.06985
0.1028	0.7659	0.05073	0.08053

MOTIF GTTATCGG

0.0707	0.04213	0.7897	0.09747
0.06862	0.06827	0.08618	0.7769
0.06212	0.0974	0.06969	0.7708
0.7238	0.0827	0.09698	0.09654
0.04686	0.07384	0.07502	0.8043

0.11	0.7254	0.07467	0.08991
0.08821	0.0556	0.7579	0.09832
0.07572	0.08716	0.7675	0.06964

MOTIF AGTCGTAC

0.7255	0.0992	0.08106	0.0942
0.05928	0.05436	0.8249	0.06147
0.0692	0.07477	0.06313	0.7929
0.1281	0.6512	0.1112	0.1094
0.0612	0.09207	0.7822	0.06448
0.08103	0.08277	0.07475	0.7615
0.7648	0.06688	0.09514	0.07316
0.05767	0.7771	0.1009	0.0643

MOTIF TAGACG

0.05652	0.06357	0.06118	0.8187
0.7941	0.09083	0.07333	0.04175
0.03596	0.04461	0.8811	0.03835
0.8303	0.06227	0.05159	0.05581
0.09553	0.7518	0.08561	0.06705
0.05146	0.05466	0.8563	0.0376

MOTIF GGCAGTAC

0.09432	0.05775	0.7584	0.08958
0.0732	0.0618	0.8074	0.05756
0.06675	0.7892	0.07549	0.06855
0.7877	0.06146	0.08495	0.06587
0.06677	0.07692	0.799	0.05726
0.08388	0.1249	0.08622	0.705
0.7528	0.09051	0.086	0.0707
0.07208	0.76	0.1053	0.06262

MOTIF GCGGTA

0.03861	0.05074	0.8461	0.06455
0.07411	0.7885	0.04589	0.09147
0.08732	0.04723	0.8102	0.05522
0.0579	0.04036	0.8683	0.03341
0.04116	0.07983	0.06265	0.8164
0.7998	0.05679	0.08602	0.05738

MOTIF TTGCGA

0.03695	0.05338	0.05616	0.8535
0.04663	0.01926	0.06144	0.8727
0.03155	0.05569	0.8698	0.04293
0.06882	0.7627	0.09481	0.0737
0.0273	0.09026	0.7872	0.09526
0.8467	0.05748	0.05713	0.03868

MOTIF TAGCGA

0.0453	0.08044	0.07152	0.8027

0.8558	0.02094	0.06847	0.05476
0.04201	0.07081	0.839	0.04822
0.07965	0.7895	0.0628	0.06803
0.09986	0.0464	0.8262	0.02758
0.8636	0.04405	0.05726	0.03512

MOTIF GCGAAT

0.05071	0.05366	0.8419	0.05371
0.05182	0.8105	0.09369	0.04395
0.06816	0.05991	0.8181	0.0538
0.8346	0.05933	0.06942	0.03661
0.7852	0.06715	0.06806	0.0796
0.0667	0.06252	0.09516	0.7756

MOTIF AATCGC

0.7598	0.0958	0.07471	0.06972
0.8491	0.05395	0.05367	0.04324
0.03985	0.07113	0.04596	0.8431
0.06845	0.7954	0.0744	0.06176
0.06503	0.0579	0.8219	0.05518
0.0525	0.866	0.04257	0.0389

MOTIF TTCACG

0.06434	0.05443	0.06359	0.8176
0.04791	0.08649	0.07063	0.795
0.04204	0.8725	0.04789	0.03757
0.7714	0.06972	0.06627	0.09259
0.05172	0.8397	0.06147	0.04713
0.05741	0.07331	0.8218	0.04747

MOTIF GGCAGT

0.07229	0.04033	0.8215	0.06589
0.05484	0.04225	0.8588	0.04406
0.05775	0.8374	0.05398	0.05083
0.8331	0.04211	0.07358	0.0512
0.05447	0.05878	0.8411	0.04567
0.05703	0.08137	0.04792	0.8137

MOTIF GCTACC

0.05259	0.03563	0.8618	0.05002
0.04655	0.8909	0.01634	0.04621
0.05354	0.0935	0.05001	0.803
0.7634	0.0919	0.08115	0.06353
0.05113	0.8685	0.0282	0.05215
0.07458	0.8177	0.04336	0.06438

MOTIF CTCGTA

0.04987	0.8223	0.07614	0.05165
0.04945	0.05007	0.04443	0.856
0.0731	0.7827	0.08197	0.0622

0.04294 0.08296 0.8378 0.03629
0.05354 0.069 0.0844 0.7931
0.7803 0.0985 0.05658 0.06462

MOTIF GTCGTA
0.03735 0.04381 0.8795 0.03939
0.03146 0.05113 0.04924 0.8682
0.09383 0.7162 0.0954 0.09459
0.05829 0.0509 0.856 0.03476
0.04513 0.0546 0.05816 0.8421
0.7814 0.08591 0.07897 0.05371

MOTIF AGTACG
0.8768 0.02081 0.06123 0.04119
0.03793 0.05752 0.87 0.03452
0.04507 0.07785 0.07093 0.8062
0.807 0.0828 0.05459 0.05563
0.06198 0.8087 0.07305 0.0563
0.09627 0.08685 0.7945 0.02243

MOTIF ACGTTC
0.8017 0.0619 0.05066 0.08577
0.07399 0.7998 0.06197 0.06428
0.04002 0.1058 0.8039 0.05032
0.07151 0.05918 0.1084 0.7609
0.09529 0.08653 0.07484 0.7433
0.04268 0.8332 0.07098 0.0531

MOTIF ACGCTT
0.7411 0.07914 0.0863 0.09347
0.02007 0.8479 0.06021 0.0718
0.07505 0.05917 0.7965 0.06926
0.07087 0.7658 0.0997 0.06358
0.04522 0.08306 0.02336 0.8484
0.06999 0.07244 0.09321 0.7644

MOTIF CCCAGT
0.04603 0.8237 0.07175 0.05854
0.07045 0.8119 0.06573 0.05189
0.05977 0.7927 0.09705 0.05048
0.8368 0.05793 0.04851 0.05675
0.07 0.07431 0.8005 0.0552
0.05279 0.07207 0.05114 0.824

MOTIF TTACGC
0.0459 0.06985 0.07725 0.807
0.06739 0.08802 0.07806 0.7665
0.7267 0.08269 0.1061 0.08452
0.03151 0.8663 0.04462 0.05762
0.07075 0.07867 0.7444 0.1062

0.0619	0.8331	0.06096	0.04408

MOTIF GCGTAT

0.05484	0.06154	0.7963	0.08733
0.08292	0.7617	0.0911	0.06431
0.05772	0.06461	0.84	0.03766
0.07212	0.07627	0.08951	0.7621
0.7954	0.07711	0.06014	0.06731
0.0502	0.0791	0.07107	0.7996

MOTIF AACTCACGAC

0.7587	0.1081	0.05856	0.07462
0.7667	0.07669	0.08668	0.06994
0.0902	0.7502	0.07677	0.08279
0.1132	0.08135	0.0691	0.7363
0.08498	0.7698	0.09291	0.0523
0.7626	0.09006	0.06878	0.07854
0.101	0.6878	0.07861	0.1326
0.1579	0.1405	0.593	0.1087
0.7594	0.08732	0.07767	0.07565
0.1174	0.7016	0.09481	0.08621

MOTIF GGTGAACGCT

0.06709	0.05772	0.794	0.0812
0.08968	0.07477	0.756	0.07954
0.06646	0.06923	0.08214	0.7822
0.0842	0.09069	0.7664	0.05866
0.7271	0.1126	0.0869	0.07343
0.6792	0.1001	0.1266	0.09412
0.09698	0.6636	0.1149	0.1245
0.1213	0.1034	0.6789	0.09628
0.09238	0.7452	0.08329	0.07915
0.1206	0.1213	0.08251	0.6755

MOTIF AGGCTTATCG

0.7799	0.06527	0.06742	0.08738
0.1079	0.05919	0.6762	0.1567
0.09095	0.07581	0.7556	0.07766
0.09528	0.6905	0.09761	0.1166
0.0851	0.06611	0.07334	0.7754
0.07878	0.08759	0.1062	0.7275
0.7175	0.09321	0.07472	0.1146
0.07497	0.07793	0.08365	0.7634
0.101	0.6405	0.1339	0.1245
0.116	0.121	0.6422	0.1207

MOTIF ACACCCACCT

0.7169	0.09497	0.1013	0.08686
0.09758	0.7395	0.08095	0.08196
0.7564	0.1031	0.06494	0.0755

0.1113	0.7058	0.08989	0.09309
0.1271	0.7328	0.05633	0.08378
0.1238	0.7312	0.06544	0.07956
0.72	0.08568	0.07875	0.1156
0.09415	0.7238	0.08339	0.09868
0.123	0.7293	0.07168	0.07598
0.1232	0.1115	0.06902	0.6963

MOTIF ATACTCAGCT

0.7002	0.09459	0.1041	0.1011
0.1162	0.07716	0.07419	0.7324
0.7049	0.09072	0.107	0.09738
0.1135	0.7024	0.06696	0.1172
0.1012	0.08759	0.05238	0.7588
0.1067	0.7275	0.06551	0.1003
0.7594	0.08522	0.0538	0.1016
0.1044	0.0925	0.6983	0.1048
0.1067	0.7349	0.07307	0.08531
0.1252	0.06184	0.06039	0.7526

MOTIF AACTCACG

0.782	0.08803	0.05321	0.07682
0.7798	0.06685	0.09585	0.05752
0.07941	0.7816	0.0669	0.0721
0.08997	0.07281	0.05997	0.7772
0.07523	0.8115	0.06962	0.04365
0.8102	0.07397	0.06014	0.05564
0.09373	0.7216	0.08551	0.09916
0.127	0.1258	0.6461	0.1012

MOTIF ACTTACCC

0.8006	0.06623	0.06479	0.06839
0.09906	0.7633	0.05433	0.08332
0.1045	0.05946	0.04441	0.7917
0.07877	0.1062	0.06258	0.7524
0.7577	0.07754	0.0919	0.07283
0.0856	0.7589	0.07923	0.07629
0.07007	0.8077	0.05013	0.0721
0.09812	0.7815	0.04129	0.07913

MOTIF GCTTATCG

0.07586	0.05522	0.8068	0.06212
0.07969	0.7657	0.06894	0.08566
0.05685	0.0609	0.05728	0.825
0.06952	0.08862	0.09988	0.742
0.7379	0.09366	0.07439	0.09403
0.05136	0.06543	0.06975	0.8135
0.06442	0.7652	0.07835	0.09202
0.0976	0.08701	0.7042	0.1112

MOTIF TACTCAGC

0.08898	0.06857	0.065	0.7775
0.73	0.0922	0.09238	0.08541
0.08972	0.7674	0.06246	0.08045
0.07587	0.06137	0.04503	0.8177
0.07013	0.8024	0.05078	0.07666
0.7968	0.07039	0.05301	0.07984
0.08444	0.08134	0.7454	0.08884
0.07368	0.7931	0.06939	0.06379

MOTIF TGCGAA

0.06266	0.04415	0.0883	0.8049
0.05423	0.06351	0.8663	0.01592
0.1077	0.7404	0.05147	0.1004
0.06956	0.05473	0.7731	0.1027
0.8751	0.01988	0.06553	0.03946
0.8538	0.04188	0.04805	0.05629

MOTIF CACTCACC

0.09996	0.7644	0.06561	0.07006
0.7705	0.07451	0.07767	0.07736
0.09392	0.765	0.06776	0.07328
0.1111	0.1038	0.06412	0.721
0.07169	0.8151	0.06072	0.05245
0.7867	0.07437	0.07611	0.06283
0.08078	0.7839	0.05994	0.07536
0.1046	0.7639	0.06201	0.06955

MOTIF GGCGTT

0.03992	0.03546	0.869	0.05562
0.03242	0.05094	0.8581	0.05852
0.0889	0.7529	0.06975	0.08848
0.04585	0.05664	0.8388	0.05867
0.06274	0.0437	0.05217	0.8414
0.0418	0.05216	0.0728	0.8332

MOTIF GTGAACGC

0.08057	0.06461	0.7833	0.07149
0.06975	0.06327	0.05496	0.812
0.06361	0.07327	0.8212	0.04189
0.7736	0.1037	0.0722	0.05048
0.7604	0.07675	0.1055	0.05726
0.07705	0.7281	0.08795	0.1069
0.09572	0.08584	0.7176	0.1008
0.07832	0.7772	0.07285	0.07166

MOTIF CGCGATTA

0.119	0.6977	0.09822	0.08512
0.1355	0.08666	0.6658	0.112
0.09566	0.7173	0.07889	0.1081

0.09136	0.08741	0.7483	0.07292
0.7982	0.06737	0.07656	0.05789
0.06467	0.08171	0.09479	0.7588
0.06275	0.07629	0.08359	0.7774
0.7577	0.08858	0.07546	0.07823

MOTIF ATTGCG

0.8234	0.05335	0.07092	0.05237
0.04134	0.05935	0.05689	0.8424
0.05125	0.05636	0.0887	0.8037
0.04556	0.0479	0.8453	0.0612
0.0915	0.7647	0.05135	0.09246
0.07266	0.06592	0.752	0.1094

MOTIF CCGACTTA

0.08227	0.7364	0.07839	0.103
0.1158	0.6888	0.08528	0.1101
0.09148	0.09824	0.7097	0.1005
0.8238	0.05192	0.0652	0.0591
0.07143	0.7597	0.08941	0.07943
0.0668	0.07451	0.0903	0.7684
0.104	0.1011	0.06974	0.7251
0.7105	0.1193	0.07453	0.0957

MOTIF CGTTCA

0.08374	0.756	0.07621	0.08402
0.07565	0.06631	0.7987	0.0593
0.04923	0.05572	0.06976	0.8253
0.04165	0.04716	0.05967	0.8515
0.03734	0.8549	0.06062	0.0471
0.8692	0.0447	0.04101	0.04505

MOTIF TTATCG

0.03755	0.04247	0.03709	0.8829
0.0647	0.07341	0.05308	0.8088
0.806	0.0677	0.06887	0.05739
0.03885	0.04451	0.05022	0.8664
0.04667	0.8417	0.05641	0.05526
0.05558	0.06972	0.7955	0.07917

MOTIF TAATCG

0.06106	0.03488	0.0505	0.8536
0.8144	0.07322	0.04837	0.06399
0.8436	0.06274	0.05528	0.03834
0.04919	0.0504	0.03819	0.8622
0.04192	0.8487	0.05651	0.05291
0.1	0.07069	0.7642	0.06515

MOTIF AACGCA

0.868	0.06198	0.03868	0.03131

0.8637 0.03726 0.05368 0.04538
0.07542 0.7864 0.06998 0.06819
0.1148 0.08559 0.6962 0.1035
0.04458 0.8651 0.0516 0.03869
0.8618 0.06381 0.03556 0.03881

MOTIF CGTGAG
0.06547 0.7597 0.09357 0.08131
0.07379 0.0713 0.7925 0.06241
0.04638 0.04212 0.04937 0.8621
0.03126 0.06511 0.8624 0.04128
0.8343 0.05123 0.05315 0.06129
0.05278 0.04498 0.8613 0.04093

MOTIF CTATCG
0.04704 0.8245 0.04564 0.08284
0.04812 0.06936 0.02456 0.858
0.783 0.07325 0.08275 0.061
0.0636 0.04826 0.05947 0.8287
0.02169 0.8642 0.06919 0.04497
0.06337 0.06007 0.7764 0.1002

MOTIF GGTATC
0.06597 0.04856 0.7986 0.08682
0.01334 0.04559 0.8736 0.06751
0.05146 0.04763 0.06876 0.8322
0.7699 0.06585 0.08027 0.08396
0.0654 0.02468 0.06156 0.8484
0.06354 0.7636 0.09355 0.07936

MOTIF TACGTT
0.01537 0.04663 0.04576 0.8922
0.8015 0.06679 0.07294 0.05878
0.09453 0.7408 0.07867 0.08604
0.06852 0.1148 0.7371 0.07959
0.04074 0.05706 0.04523 0.857
0.01698 0.04001 0.05328 0.8897

MOTIF TACGCT
0.04821 0.05503 0.06259 0.8342
0.7919 0.07829 0.0673 0.06247
0.07742 0.788 0.05823 0.07633
0.0679 0.08428 0.7807 0.06714
0.04264 0.8632 0.04559 0.04857
0.03713 0.05199 0.03831 0.8726

MOTIF CGATTG
0.07344 0.755 0.07855 0.09303
0.0195 0.05566 0.8783 0.04652
0.7814 0.07033 0.0809 0.06732

0.04655	0.05845	0.09415	0.8008
0.05882	0.0245	0.07067	0.846
0.08266	0.06468	0.7725	0.08018

MOTIF AGCGAT

0.8304	0.04212	0.07531	0.0522
0.04152	0.05526	0.8766	0.02663
0.08003	0.7598	0.08081	0.07932
0.08659	0.06461	0.7752	0.0736
0.8978	0.01617	0.03561	0.0504
0.05958	0.06089	0.06602	0.8135

MOTIF ACCCTG

0.837	0.04682	0.05703	0.0592
0.05848	0.8241	0.05748	0.05998
0.05779	0.8254	0.05391	0.06292
0.07269	0.8148	0.04699	0.06556
0.05533	0.05546	0.04667	0.8425
0.06871	0.061	0.7986	0.07173

MOTIF TGCGGA

0.07244	0.05539	0.08065	0.7915
0.04052	0.01374	0.8858	0.05992
0.09328	0.7444	0.07652	0.08581
0.08666	0.06002	0.7335	0.1199
0.04586	0.01914	0.8962	0.0388
0.882	0.03917	0.03663	0.04218

MOTIF TACAGC

0.08005	0.08246	0.06498	0.7725
0.7592	0.06267	0.08879	0.08934
0.0495	0.8512	0.04468	0.05459
0.8788	0.03197	0.03625	0.05296
0.07176	0.09546	0.772	0.06076
0.06113	0.8366	0.05382	0.04844

MOTIF CGAGTA

0.06084	0.7669	0.06937	0.1029
0.07574	0.04174	0.8325	0.05005
0.8553	0.0344	0.05507	0.05528
0.03805	0.05353	0.8469	0.06149
0.06478	0.06506	0.07063	0.7995
0.8088	0.06126	0.06594	0.06404

MOTIF AAGTCG

0.8452	0.04612	0.05247	0.05623
0.8151	0.07002	0.06042	0.05443
0.06072	0.06762	0.8302	0.04145
0.07194	0.05234	0.05412	0.8216
0.06919	0.7946	0.06566	0.07058

0.08159	0.05767	0.7874	0.07336

MOTIF TGACCGCGAC

0.08657	0.1699	0.1036	0.6399
0.05956	0.1077	0.7805	0.0522
0.674	0.09869	0.1336	0.09374
0.07958	0.7547	0.1041	0.06162
0.09924	0.6495	0.155	0.09632
0.114	0.0987	0.6659	0.1213
0.08104	0.7872	0.06031	0.07148
0.08004	0.1214	0.7445	0.05408
0.7042	0.1085	0.1196	0.06764
0.07385	0.7643	0.0985	0.06334

MOTIF TCGGCGTTTC

0.08382	0.1055	0.08509	0.7255
0.07864	0.6946	0.09017	0.1366
0.09567	0.06626	0.7329	0.1052
0.09567	0.1356	0.6437	0.1251
0.09324	0.7068	0.09695	0.103
0.08193	0.1138	0.6857	0.1186
0.07689	0.1135	0.09786	0.7118
0.07324	0.1443	0.09424	0.6882
0.05079	0.08394	0.1528	0.7125
0.08223	0.7252	0.1028	0.08979

MOTIF TCAACGCCCA

0.09452	0.1246	0.1053	0.6756
0.0579	0.834	0.05775	0.05032
0.6939	0.1382	0.1045	0.0633
0.7372	0.08699	0.07413	0.1017
0.102	0.712	0.1078	0.07822
0.09936	0.1344	0.6544	0.1118
0.1255	0.6738	0.1162	0.08448
0.06695	0.8056	0.0576	0.06988
0.1268	0.6902	0.107	0.07598
0.7384	0.08329	0.09342	0.08491

MOTIF GCGGTAAT

0.06963	0.06005	0.8002	0.07015
0.08937	0.7223	0.07283	0.1155
0.0648	0.05079	0.8105	0.07388
0.07295	0.06094	0.8088	0.05732
0.08207	0.09515	0.09197	0.7308
0.7303	0.09191	0.09244	0.08538
0.7945	0.06283	0.0748	0.06793
0.06305	0.07256	0.06376	0.8006

MOTIF GCGTTTCG

0.04517	0.08175	0.7833	0.08981

0.08678	0.7433	0.08342	0.08654
0.06259	0.08589	0.7784	0.07312
0.05766	0.1192	0.08942	0.7337
0.03554	0.08358	0.09786	0.783
0.03916	0.07058	0.1004	0.7898
0.06044	0.7618	0.1011	0.07665
0.07273	0.08299	0.7637	0.08056

MOTIF CTCAGTAG

0.08318	0.6902	0.1425	0.08404
0.06126	0.09251	0.08301	0.7632
0.03424	0.8504	0.04932	0.066
0.7712	0.06748	0.09588	0.06548
0.04711	0.05864	0.8551	0.03912
0.09756	0.1155	0.1034	0.6835
0.7545	0.1036	0.08589	0.05601
0.05349	0.05979	0.839	0.04774

MOTIF CCGACTTA

0.0773	0.7657	0.08531	0.07169
0.05762	0.8336	0.06402	0.04478
0.06766	0.08092	0.7619	0.08952
0.7334	0.1071	0.08016	0.07943
0.04142	0.8579	0.06487	0.03577
0.08344	0.1054	0.09421	0.717
0.09422	0.1168	0.1238	0.6652
0.6217	0.1553	0.1195	0.1035

MOTIF GCGGTA

0.03993	0.04578	0.8607	0.05363
0.06509	0.8011	0.06948	0.06429
0.0543	0.04567	0.8496	0.05044
0.04032	0.04762	0.8847	0.02731
0.0658	0.09276	0.08937	0.7521
0.7491	0.06877	0.09965	0.08247

MOTIF CCCGGTAA

0.07663	0.7562	0.1096	0.05761
0.08482	0.6972	0.1012	0.1168
0.06075	0.8065	0.06156	0.07121
0.06165	0.06823	0.8207	0.04939
0.05386	0.06891	0.8302	0.04706
0.09358	0.1508	0.08877	0.6668
0.7251	0.11	0.1114	0.05354
0.7831	0.07917	0.0822	0.05549

MOTIF CCGCAT

0.03827	0.854	0.06631	0.0414
0.06905	0.8142	0.06783	0.04896
0.07144	0.06625	0.8119	0.05039

0.03582	0.8875	0.04111	0.03553
0.7501	0.1237	0.07011	0.05608
0.05585	0.06374	0.06795	0.8125

MOTIF ACGCTT

0.7947	0.05545	0.08475	0.06508
0.02101	0.8351	0.08006	0.0638
0.05246	0.08196	0.8083	0.05724
0.04671	0.8599	0.04945	0.04393
0.03843	0.07085	0.01592	0.8748
0.04141	0.05083	0.06756	0.8402

MOTIF TCGGCGTT

0.05885	0.08753	0.103	0.7506
0.05916	0.7482	0.09563	0.09701
0.07009	0.04679	0.8278	0.05529
0.07374	0.096	0.7182	0.1121
0.07672	0.762	0.08096	0.08037
0.06569	0.1087	0.7372	0.08842
0.07461	0.1077	0.07474	0.743
0.05971	0.1581	0.1025	0.6797

MOTIF AACGTCCA

0.7292	0.09946	0.0972	0.07411
0.7712	0.08888	0.07812	0.06184
0.0835	0.7482	0.1038	0.06455
0.07503	0.1519	0.687	0.08604
0.1067	0.09125	0.1068	0.6952
0.03951	0.8651	0.05291	0.04252
0.07632	0.8103	0.07797	0.03544
0.798	0.05916	0.06409	0.07874

MOTIF GCGATAGG

0.05391	0.05083	0.8214	0.0739
0.06638	0.806	0.0694	0.05824
0.08861	0.1001	0.7748	0.03644
0.6776	0.1222	0.1405	0.05967
0.09785	0.1364	0.1913	0.5745
0.6821	0.09723	0.1214	0.09931
0.0683	0.07701	0.7987	0.05603
0.05906	0.07979	0.778	0.08321

MOTIF CTACGA

0.02974	0.9054	0.03415	0.0307
0.08059	0.08083	0.1423	0.6963
0.7748	0.09569	0.06042	0.06906
0.0382	0.8681	0.06267	0.03102
0.05325	0.06026	0.8319	0.05455
0.8504	0.05305	0.04492	0.05165

MOTIF GGTAAG

0.0325	0.04425	0.887	0.0362
0.03358	0.05848	0.873	0.0349
0.07034	0.09138	0.1052	0.7331
0.7916	0.08744	0.07767	0.04332
0.8199	0.05569	0.07477	0.04963
0.06001	0.06168	0.7759	0.1024

MOTIF GGACGT

0.03586	0.07047	0.8426	0.05106
0.03029	0.04615	0.8814	0.04217
0.8056	0.06685	0.0623	0.06521
0.06303	0.7944	0.1003	0.04226
0.0549	0.05547	0.842	0.04764
0.07713	0.07855	0.07832	0.766

MOTIF TTCCGC

0.04911	0.1116	0.0605	0.7788
0.04715	0.07746	0.08034	0.7951
0.02891	0.8776	0.05315	0.04031
0.05954	0.8312	0.0621	0.04713
0.07114	0.07637	0.7936	0.05889
0.05328	0.8468	0.05295	0.04696

MOTIF CGATAG

0.04812	0.8617	0.04688	0.04336
0.06433	0.06368	0.8594	0.01255
0.8053	0.08747	0.06934	0.03785
0.06223	0.1581	0.1339	0.6457
0.8147	0.02894	0.1009	0.05546
0.05654	0.0518	0.8504	0.04128

MOTIF AAGTCG

0.8184	0.0656	0.06462	0.05138
0.7657	0.08526	0.08615	0.0629
0.04047	0.04377	0.8882	0.02758
0.07171	0.06947	0.08076	0.7781
0.04073	0.8365	0.06975	0.05303
0.04468	0.06	0.8515	0.04383

MOTIF TTGACG

0.06161	0.05063	0.06268	0.8251
0.03168	0.09663	0.08541	0.7863
0.03021	0.04424	0.9028	0.02276
0.7368	0.08941	0.0859	0.08792
0.0521	0.836	0.05016	0.06174
0.06924	0.05665	0.8275	0.04662

MOTIF GCGCTT

0.04509	0.04412	0.8608	0.05001

0.05773	0.8234	0.06141	0.05744
0.04939	0.07462	0.8092	0.06675
0.04168	0.8411	0.06874	0.04849
0.04898	0.06088	0.06836	0.8218
0.03857	0.06707	0.07178	0.8226

MOTIF TCGCAG

0.0533	0.05924	0.0746	0.8129
0.04592	0.8116	0.08632	0.05616
0.04025	0.0484	0.8662	0.04511
0.06759	0.7753	0.09369	0.06339
0.7549	0.1044	0.06826	0.07243
0.03561	0.03976	0.887	0.03764

MOTIF GCGTTT

0.03666	0.05771	0.8495	0.05612
0.05143	0.8009	0.0696	0.07809
0.05759	0.09772	0.775	0.06967
0.04529	0.06927	0.07524	0.8102
0.02844	0.04477	0.06536	0.8614
0.02463	0.04643	0.06766	0.8613

MOTIF CCGTAT

0.0357	0.8533	0.05818	0.0528
0.04907	0.8407	0.07184	0.03837
0.04767	0.06983	0.8388	0.04368
0.05799	0.1111	0.06562	0.7653
0.6635	0.1348	0.09167	0.11
0.06038	0.06009	0.07871	0.8008

MOTIF TAGGCC

0.11	0.08303	0.1139	0.6931
0.725	0.09467	0.09441	0.08588
0.04315	0.05125	0.8699	0.03568
0.03089	0.06356	0.872	0.03352
0.03163	0.8669	0.06056	0.04091
0.0436	0.842	0.05281	0.06158

MOTIF GCGTAT

0.0334	0.05703	0.8595	0.05007
0.04589	0.8342	0.07614	0.04375
0.04571	0.06857	0.8324	0.05335
0.05571	0.1032	0.09614	0.745
0.7008	0.07937	0.1209	0.09886
0.05336	0.05705	0.08798	0.8016

MOTIF CCCAGT

0.0469	0.8147	0.09502	0.04343
0.03923	0.8657	0.05238	0.0427
0.05734	0.7761	0.1203	0.04622

0.8023	0.06758	0.06097	0.06916
0.04058	0.08433	0.8124	0.06271
0.0538	0.0867	0.07267	0.7868

MOTIF ACCTAACCCT

0.678	0.1272	0.07777	0.1171
0.074	0.7784	0.0651	0.08248
0.07463	0.799	0.04642	0.07994
0.134	0.1061	0.07012	0.6897
0.7206	0.108	0.08625	0.08512
0.7619	0.08728	0.08313	0.06766
0.1148	0.731	0.07167	0.08253
0.1352	0.7165	0.04627	0.102
0.1017	0.7093	0.09693	0.09203
0.09783	0.07965	0.05844	0.7641

MOTIF AGTGTTAGGG

0.7269	0.05651	0.1081	0.1086
0.08491	0.06483	0.7586	0.09168
0.1156	0.04729	0.1344	0.7027
0.08071	0.07232	0.7465	0.1004
0.08543	0.08766	0.09797	0.7289
0.09155	0.08941	0.0814	0.7376
0.6735	0.09373	0.08728	0.1455
0.08193	0.0455	0.7609	0.1117
0.09977	0.07089	0.7303	0.09903
0.126	0.06563	0.6724	0.1359

MOTIF CCCTTACACT

0.1008	0.7017	0.07586	0.1217
0.0894	0.7642	0.0656	0.08075
0.08934	0.7794	0.04223	0.089
0.122	0.0845	0.06826	0.7252
0.1506	0.09092	0.09925	0.6592
0.6367	0.1402	0.1091	0.114
0.07534	0.7579	0.0846	0.08217
0.7082	0.1189	0.06017	0.1128
0.09091	0.7427	0.0665	0.09991
0.11	0.1055	0.06187	0.7226

MOTIF ACTGAGCCGT

0.7114	0.0964	0.09444	0.09772
0.07641	0.7452	0.1021	0.07625
0.07818	0.0929	0.06572	0.7632
0.07697	0.0872	0.7314	0.1045
0.7114	0.05629	0.07519	0.1571
0.08219	0.1359	0.7318	0.05007
0.07358	0.7724	0.07852	0.07554
0.09315	0.6945	0.08994	0.1224
0.1007	0.1264	0.6571	0.1158

0.1012 0.09945 0.08095 0.7184

MOTIF CCTGTAATCG
0.08223 0.724 0.09515 0.09862
0.08436 0.7338 0.06348 0.1183
0.05375 0.07567 0.0621 0.8085
0.06167 0.1431 0.6735 0.1217
0.1067 0.1156 0.06861 0.7091
0.6814 0.1148 0.121 0.08287
0.7205 0.09526 0.1094 0.07482
0.07074 0.1061 0.07572 0.7474
0.1175 0.6702 0.08497 0.1274
0.1229 0.1099 0.639 0.1283

MOTIF CGCTAACCCG
0.1065 0.7166 0.09991 0.07691
0.08568 0.1334 0.6674 0.1135
0.06909 0.797 0.06466 0.06929
0.1117 0.1409 0.09575 0.6517
0.6895 0.153 0.1163 0.04118
0.6555 0.1162 0.1431 0.08515
0.06685 0.7587 0.09376 0.08069
0.1017 0.7366 0.06511 0.09653
0.09542 0.7125 0.08032 0.1118
0.1079 0.1319 0.634 0.1262

MOTIF TAAGTCGC
0.07974 0.092 0.07294 0.7553
0.7082 0.1119 0.06505 0.1149
0.7956 0.06071 0.07817 0.06551
0.06428 0.08132 0.7937 0.06072
0.05652 0.0834 0.06426 0.7958
0.09936 0.7248 0.08566 0.09022
0.08589 0.06918 0.7475 0.09745
0.0893 0.7378 0.08358 0.08933

MOTIF CTAGCGAC
0.07859 0.7988 0.04384 0.07878
0.07846 0.09113 0.1007 0.7297
0.7927 0.06073 0.08063 0.06599
0.0611 0.07177 0.8032 0.06389
0.09463 0.7549 0.07042 0.08006
0.1066 0.1059 0.7022 0.08528
0.7582 0.07295 0.09763 0.07123
0.07738 0.7624 0.07864 0.08159

MOTIF TAGCGA
0.04617 0.06015 0.07698 0.8167
0.8762 0.01378 0.06184 0.04818
0.03338 0.04096 0.8942 0.03145

0.08917	0.7707	0.06824	0.0719
0.07593	0.08342	0.8122	0.02846
0.8485	0.04347	0.05549	0.05251

MOTIF CTTGCGGA

0.06434	0.77	0.07644	0.08919
0.05557	0.05929	0.07079	0.8144
0.0758	0.05382	0.08265	0.7877
0.05623	0.08071	0.792	0.07104
0.1047	0.7038	0.1064	0.08513
0.07677	0.09332	0.7254	0.1046
0.08254	0.08228	0.7761	0.05912
0.7641	0.08938	0.06888	0.07761

MOTIF CGGCTGTA

0.08602	0.7672	0.0667	0.08004
0.103	0.05375	0.7496	0.09367
0.06205	0.05794	0.8119	0.06813
0.05228	0.8226	0.06424	0.06089
0.05773	0.06213	0.05122	0.8289
0.07148	0.07525	0.7768	0.0765
0.09277	0.1045	0.08948	0.7132
0.7225	0.09487	0.1054	0.07727

MOTIF TCGCGTAG

0.07624	0.06642	0.09001	0.7673
0.08865	0.6981	0.1287	0.08463
0.05917	0.07525	0.7904	0.07515
0.1361	0.7156	0.07571	0.07254
0.04058	0.126	0.7407	0.09274
0.08165	0.0961	0.09679	0.7255
0.7622	0.1072	0.06565	0.0649
0.05171	0.07485	0.8173	0.05612

MOTIF ATTGCG

0.7844	0.08466	0.06456	0.06641
0.04637	0.06274	0.05962	0.8313
0.07056	0.06071	0.09328	0.7754
0.04138	0.04612	0.8703	0.04223
0.07949	0.7843	0.06788	0.06831
0.05909	0.04314	0.8198	0.07795

MOTIF GTGTAAGG

0.08459	0.05552	0.7709	0.08895
0.09741	0.05551	0.09017	0.7569
0.06023	0.07158	0.7991	0.06904
0.09813	0.08219	0.1126	0.7071
0.6932	0.08611	0.09194	0.1287
0.7612	0.07168	0.07506	0.0921
0.06787	0.03493	0.817	0.08021

0.0652	0.06716	0.7915	0.07613

MOTIF GCGTAG

0.04579	0.05327	0.8482	0.05278
0.0847	0.7991	0.05623	0.06002
0.03717	0.08452	0.7995	0.07876
0.06833	0.08279	0.09939	0.7495
0.828	0.07405	0.05347	0.04443
0.0394	0.05641	0.8669	0.0373

MOTIF GGGTCA

0.0539	0.06587	0.8275	0.05274
0.06508	0.04476	0.8168	0.07338
0.04811	0.0812	0.8173	0.0534
0.04398	0.05254	0.06638	0.8371
0.04202	0.8073	0.07778	0.07292
0.8275	0.04788	0.07828	0.04637

MOTIF TGAGCG

0.05778	0.06676	0.06362	0.8118
0.03584	0.04736	0.8623	0.05453
0.8456	0.05192	0.05405	0.04845
0.03866	0.059	0.8629	0.03941
0.06983	0.7951	0.06596	0.06912
0.07526	0.07187	0.7692	0.08362

MOTIF TTGACCCG

0.06992	0.1113	0.07365	0.7451
0.0725	0.07973	0.06817	0.7796
0.09807	0.1156	0.7288	0.05754
0.8032	0.07713	0.06956	0.05009
0.06176	0.7274	0.1308	0.08006
0.07574	0.7735	0.06262	0.08817
0.07801	0.7496	0.1077	0.06472
0.09185	0.08469	0.7109	0.1125

MOTIF AGTCGC

0.8112	0.07897	0.05812	0.05167
0.0497	0.04708	0.8673	0.03589
0.04392	0.08474	0.05642	0.8149
0.06896	0.7888	0.08207	0.06014
0.05347	0.05263	0.8361	0.0578
0.05991	0.8093	0.06772	0.06312

MOTIF GCTGTA

0.0509	0.05327	0.8438	0.05207
0.04583	0.837	0.05648	0.06068
0.05343	0.0424	0.03096	0.8732
0.05299	0.07285	0.8198	0.0544
0.0683	0.07278	0.0693	0.7896

0.8175	0.06478	0.06434	0.05338

MOTIF GCGATT

0.04367	0.06214	0.8319	0.06231
0.07757	0.7932	0.05266	0.07661
0.06876	0.06896	0.7994	0.06286
0.8323	0.05942	0.06411	0.0442
0.04613	0.08371	0.06348	0.8067
0.03822	0.05854	0.06021	0.843

MOTIF ACTGAG

0.8104	0.0619	0.07401	0.05369
0.04221	0.8385	0.06789	0.05137
0.06302	0.05056	0.05339	0.833
0.04966	0.05895	0.8123	0.07908
0.8641	0.04144	0.03977	0.05465
0.06023	0.06016	0.813	0.0666

MOTIF GCTTAC

0.05527	0.07426	0.8278	0.04264
0.05124	0.8637	0.04708	0.03797
0.03957	0.05356	0.05273	0.8541
0.07253	0.08829	0.0906	0.7486
0.7802	0.07883	0.08013	0.06086
0.05962	0.8168	0.07317	0.05043

MOTIF CCTAAC

0.06292	0.823	0.05706	0.05706
0.04626	0.8858	0.0289	0.03901
0.1095	0.07409	0.04592	0.7705
0.8304	0.06208	0.05423	0.0533
0.8682	0.0439	0.05431	0.03362
0.07189	0.8164	0.05565	0.05608

MOTIF CGCATT

0.08938	0.7857	0.05849	0.06646
0.07824	0.07215	0.7698	0.07982
0.07551	0.7953	0.08332	0.04586
0.8105	0.08764	0.04428	0.0576
0.06623	0.07107	0.07464	0.7881
0.04177	0.06202	0.06857	0.8276

MOTIF GGCAGT

0.07551	0.04449	0.8193	0.06069
0.08789	0.0528	0.7948	0.06447
0.07337	0.8345	0.0479	0.0442
0.804	0.05192	0.06975	0.07437
0.03019	0.05018	0.8858	0.03381
0.06162	0.07434	0.0719	0.7921

MOTIF CGGAAT

0.08268	0.7991	0.05702	0.06117
0.06298	0.0731	0.8042	0.05976
0.05201	0.07015	0.8286	0.04922
0.833	0.05864	0.06913	0.03923
0.7013	0.1263	0.09006	0.08229
0.05477	0.04801	0.1073	0.79

MOTIF TAACGC

0.04912	0.05435	0.05254	0.844
0.821	0.07225	0.05872	0.04803
0.8483	0.04663	0.06422	0.04086
0.08481	0.7543	0.09547	0.06542
0.125	0.09645	0.6842	0.09436
0.0478	0.8545	0.05351	0.04417

MOTIF TACGAG

0.05977	0.04375	0.09226	0.8042
0.7972	0.08484	0.0781	0.03987
0.06301	0.7654	0.1111	0.06044
0.08589	0.05905	0.7742	0.08089
0.8402	0.05925	0.05422	0.0463
0.05129	0.0674	0.8406	0.04069

MOTIF GACGTA

0.03382	0.05029	0.897	0.01887
0.8091	0.0634	0.06408	0.06344
0.06162	0.789	0.07392	0.07548
0.05929	0.0829	0.7431	0.1147
0.0967	0.06155	0.09911	0.7426
0.8641	0.02264	0.06562	0.0476

MOTIF TTGACGCCTT

0.07156	0.07874	0.05972	0.79
0.06463	0.09825	0.07325	0.7639
0.06281	0.08181	0.7783	0.07711
0.6854	0.1024	0.09802	0.1142
0.1354	0.6304	0.114	0.1202
0.1258	0.09048	0.6587	0.125
0.06663	0.765	0.07524	0.09313
0.08893	0.7468	0.06293	0.1013
0.07379	0.07802	0.06988	0.7783
0.09268	0.06801	0.07962	0.7597

MOTIF GCGAACTGAG

0.1094	0.09235	0.6918	0.1065
0.1417	0.6772	0.08496	0.09611
0.1087	0.0852	0.7212	0.08494
0.7638	0.08332	0.09225	0.06068
0.6953	0.09441	0.1185	0.0918

0.0841	0.6956	0.1224	0.09787
0.08294	0.1082	0.08941	0.7194
0.08181	0.0946	0.7493	0.07429
0.7521	0.07318	0.0871	0.08764
0.1036	0.07711	0.7403	0.07895

MOTIF CTTAGGGCGC

0.07861	0.7673	0.0528	0.1013
0.08767	0.1348	0.1007	0.6768
0.1052	0.1192	0.1215	0.6541
0.6982	0.1074	0.1042	0.0902
0.0997	0.0555	0.7601	0.0847
0.1053	0.08236	0.7409	0.0714
0.0931	0.09016	0.723	0.09374
0.09202	0.7332	0.08416	0.09063
0.1201	0.09077	0.6342	0.1549
0.09093	0.7138	0.1041	0.09114

MOTIF GGGACCTTAA

0.1282	0.06453	0.6787	0.1285
0.1245	0.08185	0.6895	0.1042
0.1271	0.09692	0.6641	0.1118
0.7346	0.09194	0.0857	0.08774
0.09749	0.665	0.09151	0.146
0.1139	0.744	0.05372	0.08833
0.1071	0.09303	0.07617	0.7237
0.1505	0.07833	0.08391	0.6872
0.7139	0.09028	0.1041	0.09172
0.7495	0.07394	0.08698	0.08954

MOTIF TGGCAGTT

0.05836	0.04818	0.05629	0.8372
0.08541	0.05613	0.7386	0.1199
0.076	0.05948	0.778	0.08654
0.08042	0.7571	0.06803	0.09445
0.7823	0.06514	0.08135	0.07123
0.05856	0.0792	0.7693	0.09298
0.07118	0.08349	0.05866	0.7867
0.07111	0.06412	0.08683	0.7779

MOTIF GTAGCGTA

0.08416	0.0697	0.7687	0.07741
0.06339	0.08188	0.07051	0.7842
0.7927	0.05615	0.07532	0.07587
0.04707	0.07181	0.8057	0.07547
0.1054	0.6916	0.08547	0.1175
0.1439	0.08289	0.6791	0.09412
0.07053	0.07337	0.08159	0.7745
0.8048	0.0625	0.05789	0.07477

MOTIF GACCGTAT

0.07576	0.08477	0.7586	0.08082
0.8241	0.06904	0.06073	0.04613
0.04375	0.8183	0.06666	0.07125
0.1068	0.7535	0.06805	0.07163
0.107	0.1093	0.6405	0.1432
0.08386	0.07027	0.03827	0.8076
0.6808	0.107	0.1164	0.09576
0.09303	0.08329	0.1213	0.7024

MOTIF AGCGTA

0.8726	0.04472	0.04287	0.03979
0.03119	0.06122	0.8494	0.05818
0.06667	0.7758	0.06256	0.09493
0.1094	0.07874	0.746	0.06587
0.05052	0.06274	0.05879	0.8279
0.8635	0.053	0.04736	0.03613

MOTIF ATCGCGTA

0.7776	0.06208	0.09052	0.0698
0.06324	0.07048	0.06791	0.7984
0.1285	0.6371	0.102	0.1323
0.08435	0.06139	0.7332	0.1211
0.07004	0.7714	0.06338	0.09519
0.1127	0.08921	0.7239	0.07415
0.07412	0.07957	0.08561	0.7607
0.766	0.08426	0.08045	0.06929

MOTIF TGCCTTAG

0.0856	0.04691	0.0904	0.7771
0.08049	0.08026	0.7388	0.1005
0.06601	0.8042	0.06547	0.06432
0.07609	0.7548	0.04357	0.1256
0.08381	0.08843	0.064	0.7638
0.08573	0.06542	0.0672	0.7817
0.7339	0.08133	0.07933	0.1055
0.1003	0.0611	0.7589	0.07969

MOTIF CTCGTAGT

0.07087	0.765	0.07879	0.08534
0.05518	0.0661	0.04187	0.8369
0.09238	0.6838	0.07351	0.1503
0.1045	0.08501	0.715	0.09549
0.09267	0.06974	0.09226	0.7453
0.7347	0.09636	0.07744	0.09148
0.0792	0.08976	0.7658	0.06526
0.06335	0.0762	0.07143	0.789

MOTIF ATTGCG

0.8468	0.05248	0.04815	0.0526

0.05227	0.06147	0.03934	0.8469
0.0438	0.04356	0.09134	0.8213
0.04955	0.03387	0.8621	0.05443
0.07918	0.7892	0.05477	0.07683
0.1025	0.06547	0.7635	0.06853

MOTIF TCAGTATG

0.09462	0.05929	0.08843	0.7577
0.07985	0.7536	0.0806	0.08598
0.8359	0.04789	0.05811	0.05814
0.0835	0.09157	0.7342	0.0907
0.05977	0.07485	0.0647	0.8007
0.7148	0.0843	0.09093	0.11
0.07203	0.04848	0.08924	0.7903
0.09232	0.05289	0.7546	0.1002

MOTIF ACCGTA

0.8858	0.0372	0.04446	0.03251
0.03008	0.8567	0.05856	0.05465
0.08393	0.814	0.04032	0.06175
0.09268	0.1033	0.7059	0.09817
0.07243	0.05294	0.04956	0.8251
0.8162	0.07453	0.06176	0.04753

MOTIF TTAGGCGT

0.06181	0.06408	0.07191	0.8022
0.0644	0.07035	0.07315	0.7921
0.7654	0.06979	0.0628	0.102
0.06224	0.06561	0.8109	0.06125
0.05702	0.06779	0.7694	0.1058
0.115	0.6642	0.1178	0.103
0.09401	0.1024	0.6322	0.1713
0.06053	0.05744	0.05831	0.8237

MOTIF ATCGTACC

0.7879	0.07321	0.06223	0.07662
0.05669	0.08062	0.07594	0.7868
0.07823	0.7273	0.104	0.09051
0.1078	0.07986	0.7342	0.07815
0.09632	0.07073	0.07673	0.7562
0.7656	0.09086	0.07313	0.07039
0.0707	0.7511	0.1115	0.06673
0.07786	0.7615	0.0721	0.0885

MOTIF CACGGTTA

0.06576	0.7697	0.07758	0.08694
0.8138	0.05026	0.0634	0.07258
0.1369	0.656	0.1052	0.1019
0.1046	0.05866	0.7032	0.1335
0.09443	0.06663	0.7624	0.07655

0.06158	0.1147	0.05296	0.7708
0.07448	0.1056	0.08472	0.7352
0.7081	0.0841	0.1003	0.1075

MOTIF TTACGCGT

0.05538	0.05414	0.0672	0.8233
0.07069	0.06831	0.08525	0.7757
0.7941	0.05638	0.0687	0.08078
0.09641	0.653	0.1542	0.09634
0.08175	0.05189	0.7763	0.09005
0.1134	0.7277	0.07824	0.08064
0.08851	0.1016	0.6734	0.1365
0.05891	0.06914	0.05659	0.8154

MOTIF GACGCCTT

0.04955	0.06802	0.8255	0.05695
0.7144	0.11	0.07952	0.0961
0.1055	0.7309	0.07083	0.09277
0.1199	0.08657	0.6923	0.1013
0.07009	0.7965	0.06345	0.07001
0.07688	0.8153	0.03971	0.06807
0.0841	0.06401	0.07158	0.7803
0.08481	0.08638	0.08203	0.7468

MOTIF CTAAGGTC

0.08392	0.7513	0.06079	0.104
0.07846	0.1105	0.0715	0.7395
0.7571	0.07271	0.08195	0.08827
0.7782	0.06935	0.08534	0.0671
0.07891	0.04981	0.7936	0.07771
0.07507	0.08179	0.7632	0.07997
0.06502	0.08122	0.08642	0.7673
0.0797	0.7339	0.07891	0.1075

MOTIF ATAGGGTC

0.6842	0.1135	0.07662	0.1257
0.1066	0.08959	0.05553	0.7483
0.7837	0.05247	0.08615	0.07769
0.1148	0.06529	0.7336	0.08627
0.08359	0.06004	0.7503	0.1061
0.08025	0.07019	0.7853	0.06429
0.073	0.07135	0.07041	0.7852
0.0782	0.7009	0.0882	0.1327

MOTIF ATTACG

0.8775	0.0358	0.0426	0.04411
0.03968	0.05225	0.0456	0.8625
0.03623	0.06662	0.06334	0.8338
0.8308	0.03842	0.06844	0.06229
0.1066	0.678	0.1074	0.1079

0.0682	0.04752	0.8338	0.05044

MOTIF CAATCG

0.06403	0.8338	0.04665	0.05548
0.8524	0.08657	0.0191	0.04196
0.8353	0.05376	0.05871	0.05226
0.07478	0.05952	0.06353	0.8022
0.07671	0.8031	0.09552	0.02462
0.07693	0.05562	0.7988	0.0686

MOTIF ATCGTA

0.8593	0.05847	0.04118	0.04102
0.02813	0.05799	0.05762	0.8563
0.08143	0.7482	0.09177	0.07858
0.09203	0.08205	0.748	0.07794
0.05172	0.05336	0.04793	0.847
0.8481	0.05973	0.0534	0.0388

MOTIF GACGTT

0.01301	0.05002	0.9007	0.03627
0.8224	0.05695	0.05681	0.06385
0.0902	0.7678	0.07896	0.06301
0.09248	0.1281	0.7176	0.06191
0.06861	0.0576	0.08716	0.7866
0.04243	0.01886	0.04402	0.8947

MOTIF ATAGCG

0.8398	0.05134	0.0536	0.05526
0.05272	0.05906	0.04874	0.8395
0.7696	0.05092	0.1083	0.07127
0.03815	0.04685	0.858	0.05697
0.0792	0.7499	0.08191	0.08898
0.1006	0.06621	0.7613	0.0719

MOTIF CGCATA

0.1395	0.6712	0.07508	0.1142
0.07308	0.07883	0.7631	0.08499
0.06295	0.8592	0.03716	0.04069
0.8278	0.07079	0.05667	0.04472
0.07084	0.05464	0.06768	0.8068
0.8036	0.0568	0.06805	0.07158

MOTIF CTCAGT

0.06619	0.7976	0.0613	0.07491
0.05999	0.03945	0.0537	0.8469
0.06612	0.81	0.06108	0.06276
0.8666	0.04524	0.0446	0.04357
0.06889	0.06185	0.8076	0.06165
0.05557	0.06744	0.06013	0.8169

MOTIF ACCGCA

0.8487	0.05607	0.05423	0.04105
0.04722	0.8294	0.0649	0.05851
0.07045	0.76	0.05425	0.1153
0.08154	0.07066	0.7746	0.07325
0.08003	0.7993	0.0545	0.06618
0.8379	0.06682	0.05143	0.04381

MOTIF AGCGAT

0.7963	0.05257	0.08892	0.06222
0.05033	0.05884	0.8722	0.01864
0.07661	0.744	0.07319	0.1062
0.08175	0.09489	0.7516	0.07174
0.8863	0.01737	0.05204	0.04427
0.05933	0.06088	0.0668	0.813

MOTIF ATACTG

0.8158	0.07005	0.05676	0.05739
0.07336	0.0766	0.05699	0.7931
0.8416	0.04813	0.05935	0.05093
0.08186	0.7958	0.06763	0.05468
0.03445	0.03639	0.03321	0.8959
0.07187	0.05047	0.821	0.05662

MOTIF CGTGAT

0.09325	0.7406	0.06828	0.0979
0.09163	0.06538	0.7478	0.09518
0.05361	0.04931	0.0353	0.8618
0.05479	0.06163	0.8484	0.0352
0.8237	0.07499	0.05589	0.04543
0.05072	0.0526	0.05291	0.8438

MOTIF GCCTTA

0.07931	0.06088	0.7941	0.06573
0.04301	0.8724	0.04612	0.03848
0.06253	0.8401	0.02729	0.07011
0.05998	0.07131	0.0346	0.8341
0.06869	0.06282	0.06844	0.8
0.7707	0.07553	0.06676	0.08698

MOTIF ATATCG

0.8576	0.04484	0.04425	0.05327
0.06897	0.05329	0.06219	0.8156
0.7931	0.06319	0.07094	0.07278
0.04147	0.05128	0.05806	0.8492
0.06552	0.7571	0.09445	0.08294
0.08974	0.05951	0.8194	0.03134

MOTIF GGCGAT

0.04338	0.04442	0.8591	0.05306

0.06068	0.0415	0.8393	0.05855
0.06934	0.8174	0.05009	0.06321
0.06444	0.07341	0.8076	0.05459
0.844	0.04576	0.05955	0.05068
0.07679	0.06465	0.07041	0.7882

MOTIF CGTTCA

0.1095	0.7413	0.08017	0.06907
0.06416	0.09795	0.7597	0.07823
0.06531	0.0524	0.07299	0.8093
0.04495	0.06707	0.06362	0.8244
0.04891	0.8412	0.05722	0.05271
0.8291	0.05386	0.05626	0.06076

MOTIF ACTGCG

0.8335	0.05522	0.06211	0.04916
0.05128	0.8513	0.03857	0.05888
0.04849	0.04464	0.06061	0.8463
0.04439	0.06557	0.8159	0.07412
0.07823	0.8094	0.04414	0.06823
0.09889	0.07214	0.743	0.08601

MOTIF ATCGGT

0.7445	0.07165	0.08553	0.09833
0.06896	0.01087	0.05582	0.8643
0.0723	0.7769	0.07442	0.07643
0.1109	0.05098	0.7799	0.05818
0.02066	0.06282	0.8632	0.05335
0.06315	0.05385	0.0547	0.8283

MOTIF CGTAGA

0.09516	0.7828	0.06204	0.06004
0.08304	0.06834	0.7525	0.0961
0.05925	0.06054	0.06053	0.8197
0.8232	0.07671	0.04584	0.05426
0.04663	0.04402	0.8737	0.0357
0.8337	0.06639	0.04693	0.05298

MOTIF TTCGCA

0.06178	0.05794	0.05436	0.8259
0.04802	0.0481	0.02524	0.8786
0.09843	0.7101	0.09746	0.09401
0.07112	0.05175	0.7994	0.07777
0.02086	0.8717	0.0563	0.05114
0.7979	0.09175	0.05284	0.05754

MOTIF TGACCCGATC

0.07953	0.1017	0.06335	0.7554
0.05351	0.08141	0.7957	0.06941
0.7472	0.07778	0.079	0.096

0.08965	0.7187	0.09055	0.1011
0.1032	0.7302	0.07015	0.09638
0.09288	0.7459	0.08345	0.07775
0.08948	0.06616	0.7633	0.08104
0.7643	0.08403	0.08146	0.07023
0.07821	0.07616	0.08199	0.7636
0.06064	0.7927	0.07333	0.07335

MOTIF TGTCACGATC

0.09767	0.1118	0.0503	0.7402
0.05815	0.07215	0.7639	0.1058
0.1083	0.08021	0.0868	0.7246
0.07328	0.7692	0.07871	0.07885
0.7287	0.1009	0.06446	0.106
0.1029	0.6899	0.1092	0.09801
0.09054	0.1457	0.674	0.08972
0.733	0.07993	0.07573	0.1114
0.07451	0.07661	0.1212	0.7277
0.09651	0.7484	0.08477	0.07034

MOTIF TGCGAGTTAA

0.0871	0.06	0.105	0.7479
0.1105	0.07358	0.7133	0.1027
0.1129	0.6741	0.1093	0.1037
0.1019	0.07292	0.7148	0.1103
0.7613	0.04275	0.1075	0.0884
0.1007	0.06691	0.7355	0.09687
0.08	0.06863	0.09623	0.7551
0.0566	0.1094	0.08017	0.7538
0.7045	0.09582	0.0922	0.1074
0.7136	0.07752	0.09646	0.1124

MOTIF TTAACCCGCA

0.0877	0.1012	0.09673	0.7143
0.1205	0.1205	0.1075	0.6515
0.6791	0.1122	0.1173	0.09141
0.8198	0.05859	0.05202	0.06961
0.1046	0.7206	0.07939	0.0955
0.1106	0.6556	0.05339	0.1804
0.1342	0.6714	0.07995	0.1145
0.1093	0.1116	0.6929	0.0862
0.114	0.7008	0.0648	0.1203
0.7141	0.1071	0.08521	0.09356

MOTIF CCAATCCG

0.06688	0.8054	0.04809	0.07965
0.09811	0.7954	0.05204	0.05448
0.7828	0.09387	0.07854	0.04479
0.7834	0.07651	0.07942	0.06066
0.0829	0.09903	0.07355	0.7445

0.04167	0.8329	0.07087	0.05456
0.1086	0.7739	0.04423	0.07325
0.08325	0.08743	0.7473	0.08202

MOTIF CGAGTCAC

0.08902	0.756	0.06597	0.08905
0.07636	0.076	0.7973	0.05033
0.7451	0.07082	0.09244	0.09161
0.093	0.07094	0.7751	0.061
0.07654	0.1089	0.09298	0.7216
0.06663	0.7491	0.1013	0.08293
0.7995	0.05373	0.09288	0.05393
0.09391	0.7347	0.08958	0.08181

MOTIF TCGTTCCA

0.07523	0.09088	0.07646	0.7574
0.08716	0.7522	0.06334	0.09728
0.09176	0.07585	0.7479	0.0845
0.07508	0.0733	0.08941	0.7622
0.03527	0.09755	0.1086	0.7586
0.07589	0.7099	0.1079	0.1062
0.07146	0.8193	0.0509	0.05833
0.7392	0.0993	0.06983	0.09168

MOTIF CAACCCGC

0.1112	0.7171	0.09428	0.07742
0.7637	0.1022	0.06723	0.06691
0.7615	0.06857	0.1113	0.05855
0.07606	0.8023	0.05621	0.06539
0.06166	0.7854	0.06678	0.08615
0.09354	0.7278	0.08318	0.09553
0.09154	0.09484	0.7559	0.05769
0.09887	0.7626	0.06073	0.07779

MOTIF TCGGGTCA

0.08786	0.07931	0.09072	0.7421
0.05619	0.8029	0.05207	0.08881
0.05453	0.07046	0.7763	0.09869
0.1034	0.06913	0.7211	0.1063
0.0888	0.0904	0.7043	0.1165
0.06402	0.08054	0.06484	0.7906
0.06998	0.7718	0.0689	0.08931
0.7719	0.07382	0.09112	0.06315

MOTIF GCTACTGG

0.06415	0.06066	0.8014	0.07379
0.07706	0.7875	0.07672	0.05868
0.08456	0.08757	0.09548	0.7324
0.6987	0.08257	0.1199	0.09887
0.06246	0.7343	0.1203	0.08297

0.05979	0.06177	0.06336	0.8151
0.0899	0.09956	0.7332	0.07735
0.09938	0.09717	0.6986	0.1048

MOTIF TTGATCGG

0.07975	0.08226	0.06872	0.7693
0.06219	0.07328	0.08871	0.7758
0.06986	0.08341	0.7862	0.06056
0.7388	0.1058	0.08397	0.07139
0.06774	0.05806	0.06061	0.8136
0.08101	0.698	0.1237	0.09722
0.09201	0.1208	0.7023	0.0849
0.05858	0.06366	0.803	0.07479

MOTIF CTGTCACG

0.09065	0.7275	0.09978	0.08202
0.06835	0.08226	0.07695	0.7724
0.06459	0.05586	0.7993	0.08021
0.08464	0.1022	0.09526	0.7178
0.04616	0.8251	0.05602	0.07277
0.7849	0.07013	0.07371	0.07124
0.08208	0.7339	0.1108	0.07325
0.07889	0.09782	0.7539	0.06939

MOTIF ATCCGT

0.805	0.06524	0.05017	0.07958
0.07028	0.05868	0.04619	0.8248
0.03632	0.8336	0.04931	0.08079
0.06665	0.8193	0.05106	0.06298
0.05683	0.09267	0.7691	0.08138
0.05852	0.04942	0.08335	0.8087

MOTIF TTCCGT

0.08366	0.07119	0.05379	0.7914
0.06837	0.05352	0.04369	0.8344
0.04474	0.8425	0.04613	0.06667
0.07815	0.7998	0.06301	0.059
0.06544	0.09148	0.7482	0.09484
0.06061	0.04211	0.07158	0.8257

MOTIF TATACG

0.06333	0.05761	0.08169	0.7974
0.8162	0.06394	0.05507	0.0648
0.07652	0.1008	0.08241	0.7403
0.7923	0.06916	0.05347	0.08506
0.06161	0.8056	0.07823	0.05456
0.051	0.09631	0.7709	0.08177

MOTIF CGTGAC

0.05324	0.8164	0.07442	0.05591

0.06817	0.07401	0.7944	0.06338
0.0476	0.05883	0.04771	0.8459
0.05522	0.05655	0.8441	0.0441
0.7331	0.08736	0.08651	0.09301
0.07384	0.8211	0.05248	0.05255

MOTIF CGTAAC

0.06876	0.7585	0.09133	0.08141
0.07397	0.06873	0.7936	0.06372
0.07055	0.05769	0.09302	0.7787
0.769	0.06948	0.1083	0.05328
0.8291	0.05666	0.05773	0.05654
0.04421	0.8679	0.03241	0.05549

MOTIF ACGGTA

0.852	0.04106	0.05298	0.05392
0.1012	0.7403	0.08471	0.07381
0.1212	0.04731	0.7629	0.06857
0.04838	0.05664	0.8396	0.05535
0.09091	0.04679	0.05669	0.8056
0.7883	0.06363	0.06756	0.08051

MOTIF TACACG

0.07922	0.06813	0.1268	0.7258
0.754	0.06855	0.07665	0.1008
0.05876	0.8185	0.06213	0.0606
0.8024	0.07429	0.05158	0.07172
0.0705	0.8089	0.06498	0.05565
0.06204	0.05151	0.799	0.08741

MOTIF GGAACG

0.05336	0.04481	0.85	0.05184
0.07676	0.06785	0.8092	0.04616
0.7978	0.09266	0.06435	0.04516
0.8116	0.06277	0.0613	0.06432
0.08443	0.771	0.07665	0.06794
0.08138	0.07275	0.7817	0.0642

MOTIF GTTACC

0.03991	0.05382	0.8143	0.092
0.05021	0.05886	0.02869	0.8622
0.06175	0.08042	0.05809	0.7997
0.7415	0.06358	0.08421	0.1107
0.02438	0.8484	0.06367	0.06356
0.06556	0.8119	0.06163	0.06094

MOTIF CCGCTT

0.05373	0.8236	0.05263	0.07006
0.09948	0.7816	0.06094	0.05803
0.07168	0.08423	0.783	0.06109

0.05652	0.7881	0.07582	0.0796
0.06083	0.05735	0.07114	0.8107
0.0577	0.07129	0.06242	0.8086

MOTIF AAGCGT

0.8231	0.06209	0.05534	0.05947
0.8523	0.02562	0.05796	0.0641
0.0405	0.08734	0.8189	0.05323
0.08253	0.73	0.08636	0.1011
0.09344	0.07972	0.791	0.03579
0.07962	0.05948	0.08459	0.7763

MOTIF CAGGGT

0.07667	0.7967	0.06272	0.06389
0.7932	0.05665	0.08101	0.06919
0.05921	0.06064	0.8123	0.06786
0.05762	0.05985	0.8117	0.07082
0.07317	0.07933	0.7723	0.07521
0.08238	0.04001	0.07082	0.8068

MOTIF CGATCA

0.06731	0.8126	0.08466	0.03541
0.08888	0.08494	0.7645	0.06166
0.838	0.0578	0.0525	0.05167
0.06398	0.06645	0.08812	0.7815
0.03706	0.8526	0.055	0.05532
0.8304	0.06424	0.02054	0.08482

MOTIF CATTCG

0.07527	0.7936	0.07518	0.05595
0.8038	0.08957	0.05445	0.05221
0.04431	0.09094	0.08032	0.7844
0.03823	0.09538	0.07782	0.7886
0.06404	0.7941	0.06865	0.07323
0.0752	0.08204	0.7833	0.05948

MOTIF TAACTC

0.08403	0.06954	0.04087	0.8056
0.8012	0.06994	0.08315	0.04569
0.8296	0.05384	0.06066	0.05585
0.0556	0.8216	0.06324	0.05957
0.0715	0.05949	0.04236	0.8267
0.04493	0.8318	0.05379	0.0695

MOTIF TGACTC

0.05224	0.0664	0.03341	0.8479
0.09499	0.07656	0.7782	0.05022
0.7743	0.07151	0.07595	0.07826
0.05629	0.8121	0.0666	0.06497
0.05816	0.06416	0.05419	0.8235

0.0325	0.8114	0.06598	0.09014

MOTIF TGGCGT

0.06252	0.07575	0.05809	0.8036
0.06659	0.07009	0.8051	0.0582
0.05952	0.0576	0.8237	0.05915
0.1016	0.7703	0.05881	0.06928
0.05634	0.05205	0.7984	0.09323
0.08177	0.09313	0.0742	0.7509

MOTIF ACTGGG

0.8029	0.05435	0.0929	0.04985
0.07534	0.7396	0.08987	0.0952
0.07687	0.05147	0.06486	0.8068
0.05528	0.08599	0.7807	0.07802
0.07643	0.05687	0.8025	0.06417
0.08812	0.07661	0.7577	0.07757

MOTIF AGTAGC

0.8589	0.04566	0.04941	0.04605
0.04723	0.06933	0.824	0.05939
0.08842	0.09905	0.07309	0.7394
0.7963	0.06171	0.07276	0.06921
0.0531	0.04438	0.8503	0.05221
0.07769	0.816	0.05512	0.05115

MOTIF GTACGTTACG

0.09107	0.06891	0.743	0.09703
0.08569	0.09405	0.09196	0.7283
0.7194	0.1058	0.08195	0.09288
0.08545	0.7182	0.08704	0.1094
0.103	0.08545	0.7121	0.09941
0.08117	0.07134	0.1459	0.7016
0.07135	0.08134	0.101	0.7463
0.6744	0.1102	0.1164	0.09894
0.08222	0.7194	0.09765	0.1007
0.132	0.07655	0.693	0.09849

MOTIF TCTAACGCTA

0.07246	0.07244	0.06744	0.7877
0.08854	0.7301	0.07429	0.1071
0.09805	0.08706	0.0572	0.7577
0.7817	0.08303	0.05756	0.07776
0.7512	0.06989	0.08807	0.09089
0.1435	0.6486	0.0885	0.1194
0.1457	0.1081	0.6034	0.1428
0.09352	0.7333	0.08126	0.09193
0.0849	0.0709	0.04998	0.7942
0.777	0.07222	0.06075	0.09

MOTIF CGTAACGT

0.06993	0.7551	0.07065	0.1043
0.09383	0.0736	0.7595	0.07305
0.08403	0.07831	0.08141	0.7562
0.7459	0.09532	0.0929	0.06587
0.7547	0.1039	0.07405	0.06737
0.07826	0.7642	0.07361	0.08393
0.09337	0.07241	0.7521	0.08212
0.07227	0.05933	0.07079	0.7976

MOTIF AGCGTA

0.906	0.03257	0.03292	0.02851
0.04671	0.05	0.8604	0.04286
0.05646	0.8216	0.05638	0.06557
0.0604	0.04889	0.8327	0.05802
0.03965	0.03863	0.05556	0.8662
0.8666	0.04066	0.05161	0.04116

MOTIF GCCCGTTACA

0.1102	0.07255	0.7214	0.0959
0.1016	0.696	0.08369	0.1186
0.1134	0.7283	0.06269	0.09564
0.08055	0.7606	0.07207	0.08679
0.09367	0.08047	0.7169	0.109
0.09673	0.08555	0.0995	0.7182
0.07325	0.1024	0.09632	0.7281
0.6637	0.1148	0.1229	0.09863
0.09094	0.7606	0.08545	0.06303
0.7675	0.0817	0.08604	0.06473

MOTIF TTACGG

0.04185	0.03682	0.04587	0.8755
0.04482	0.0506	0.04759	0.857
0.8597	0.04192	0.04051	0.05782
0.06348	0.7863	0.07848	0.07169
0.057	0.04761	0.8482	0.04715
0.04241	0.03962	0.8717	0.04629

MOTIF TAACGG

0.07471	0.07463	0.05166	0.799
0.8615	0.05091	0.0476	0.03998
0.8846	0.03978	0.04134	0.03426
0.05941	0.8304	0.05713	0.05308
0.0469	0.04671	0.8657	0.04066
0.04843	0.03235	0.8742	0.04506

MOTIF GATACG

0.04461	0.03897	0.8699	0.04654
0.8379	0.05816	0.0556	0.04836
0.08874	0.07842	0.06103	0.7718

0.8972 0.03899 0.03287 0.03099
0.04193 0.8896 0.03258 0.03593
0.0485 0.02899 0.8923 0.03026

MOTIF ATTGCG
0.7627 0.07571 0.088 0.07355
0.03928 0.02124 0.03316 0.9063
0.06525 0.03902 0.05336 0.8424
0.07178 0.05556 0.8027 0.06996
0.02467 0.8895 0.03652 0.04935
0.04828 0.02856 0.8842 0.03892

MOTIF TAAGCG
0.04208 0.05161 0.05664 0.8497
0.8629 0.03863 0.03845 0.06003
0.8911 0.04233 0.03486 0.03174
0.0599 0.06035 0.8429 0.03687
0.05236 0.8153 0.07744 0.05487
0.05656 0.04664 0.8219 0.07486

MOTIF TATCGG
0.0379 0.03029 0.0467 0.8851
0.8317 0.03006 0.05421 0.08399
0.06138 0.05498 0.05827 0.8254
0.07073 0.832 0.06075 0.03653
0.032 0.05733 0.8809 0.02977
0.04809 0.03797 0.8409 0.073

MOTIF TGTACG
0.03567 0.01882 0.05594 0.8896
0.04396 0.03509 0.8616 0.05932
0.03741 0.03812 0.03191 0.8926
0.8456 0.07522 0.03062 0.04857
0.08284 0.8124 0.04123 0.06356
0.01643 0.05945 0.8733 0.05086

MOTIF CGTATT
0.04261 0.8519 0.04836 0.05718
0.04564 0.03628 0.8702 0.04789
0.02804 0.0319 0.03654 0.9035
0.8165 0.03019 0.06595 0.08737
0.05591 0.04185 0.04352 0.8587
0.05144 0.0584 0.04349 0.8467

MOTIF CGAAACGC
0.06536 0.7995 0.06775 0.06735
0.07594 0.09197 0.7635 0.06855
0.7845 0.0926 0.06862 0.05428
0.806 0.07932 0.06594 0.04878
0.8214 0.07751 0.05886 0.04226

0.06478	0.822	0.06209	0.05111
0.09012	0.07871	0.7771	0.05403
0.1109	0.7052	0.1049	0.07897

MOTIF CGGTTA
0.06386	0.8243	0.05374	0.0581
0.04441	0.04856	0.8566	0.0504
0.02969	0.03132	0.8898	0.04921
0.04021	0.04158	0.05781	0.8604
0.04923	0.05014	0.0521	0.8485
0.824	0.06158	0.05155	0.06289

MOTIF GTTAGCGT
0.06048	0.05336	0.7929	0.09323
0.05767	0.06893	0.05617	0.8172
0.05852	0.07062	0.07388	0.797
0.7742	0.06071	0.08484	0.08021
0.05967	0.07083	0.7984	0.07114
0.09294	0.7105	0.0999	0.09664
0.09081	0.07526	0.7304	0.1036
0.0643	0.05587	0.05149	0.8283

MOTIF TTAGCG
0.03752	0.03998	0.04244	0.8801
0.03155	0.03078	0.03058	0.9071
0.8826	0.03298	0.04166	0.0428
0.04394	0.04826	0.865	0.04282
0.08999	0.7701	0.08301	0.05688
0.06489	0.04881	0.8089	0.07742

MOTIF CGGTAA
0.05791	0.826	0.05499	0.06113
0.05408	0.04692	0.8341	0.06495
0.04637	0.05158	0.8533	0.04871
0.04814	0.0372	0.03929	0.8754
0.8356	0.05444	0.05645	0.05354
0.8654	0.05009	0.03987	0.04462

MOTIF CGTTTA
0.07614	0.8238	0.05199	0.04808
0.03871	0.06834	0.8366	0.05634
0.03046	0.03208	0.05028	0.8872
0.04101	0.02498	0.04241	0.8916
0.04717	0.03291	0.03802	0.8819
0.7953	0.07568	0.06377	0.06526

MOTIF GACCGTAT
0.09332	0.08536	0.7334	0.08794
0.8458	0.0513	0.04846	0.05439
0.06631	0.802	0.06176	0.06993

0.0806	0.7677	0.07709	0.07465
0.1044	0.06756	0.7459	0.08221
0.06131	0.04847	0.06277	0.8274
0.7259	0.08064	0.08261	0.1109
0.07439	0.08734	0.07564	0.7626

MOTIF CAGCGTAT

0.1215	0.6453	0.1025	0.1307
0.8545	0.05278	0.05373	0.03898
0.1036	0.1479	0.6662	0.0823
0.04981	0.8123	0.06723	0.07068
0.07737	0.05288	0.79	0.07978
0.03792	0.04316	0.06346	0.8555
0.764	0.05217	0.08471	0.09915
0.1158	0.09481	0.07787	0.7116

MOTIF TTACGGTG

0.08077	0.05809	0.1455	0.7156
0.06119	0.05861	0.08025	0.8
0.7451	0.1089	0.06968	0.07635
0.07663	0.7278	0.09227	0.1033
0.07936	0.07805	0.7701	0.07254
0.05619	0.06301	0.8035	0.07734
0.0496	0.04476	0.04821	0.8574
0.07769	0.06712	0.7537	0.1015

MOTIF GCCACTCCAG

0.07988	0.1039	0.7085	0.1077
0.0588	0.741	0.1258	0.07442
0.07725	0.7699	0.06801	0.08488
0.7134	0.09626	0.09962	0.09077
0.09494	0.7296	0.1008	0.07464
0.08279	0.07705	0.101	0.7392
0.06452	0.7741	0.09307	0.06832
0.06609	0.7697	0.07976	0.08449
0.7284	0.09317	0.1103	0.0682
0.0824	0.1132	0.7253	0.0791

MOTIF TTGCAGTCCC

0.09935	0.1031	0.09856	0.699
0.05618	0.0587	0.08146	0.8037
0.06506	0.1073	0.7193	0.1083
0.0692	0.7566	0.08764	0.08658
0.7344	0.1053	0.07732	0.08299
0.08943	0.1369	0.7021	0.07158
0.1236	0.08575	0.08588	0.7048
0.07393	0.6936	0.129	0.1034
0.0852	0.6706	0.1096	0.1346
0.1052	0.7006	0.08862	0.1056

MOTIF TCAATCGGGA

0.1294	0.07682	0.08191	0.7119
0.07648	0.71	0.1239	0.08961
0.7018	0.123	0.1168	0.05842
0.7402	0.1029	0.07236	0.08446
0.1165	0.1013	0.05968	0.7225
0.07283	0.7063	0.1029	0.1179
0.096	0.06958	0.7689	0.06554
0.0859	0.08972	0.7401	0.08425
0.1008	0.1447	0.6107	0.1438
0.7821	0.07224	0.06476	0.08086

MOTIF GTCTACGA

0.07885	0.09913	0.7621	0.05987
0.08583	0.07189	0.07613	0.7662
0.08524	0.7941	0.07374	0.04694
0.08092	0.1014	0.1364	0.6812
0.7067	0.1128	0.06927	0.1112
0.04853	0.823	0.07084	0.0576
0.08074	0.06313	0.7534	0.1028
0.7659	0.07941	0.08406	0.0706

MOTIF CGGGATTA

0.1151	0.6981	0.08156	0.1053
0.08513	0.09579	0.7264	0.09269
0.04681	0.0444	0.853	0.05584
0.06608	0.0739	0.783	0.077
0.7986	0.06242	0.06034	0.07865
0.06438	0.06456	0.0907	0.7804
0.07173	0.09693	0.08768	0.7437
0.7135	0.07191	0.116	0.09863

MOTIF ACTGGATC

0.7807	0.08189	0.0806	0.05684
0.06904	0.7623	0.0859	0.08273
0.08947	0.07584	0.0657	0.769
0.06071	0.07875	0.7619	0.09862
0.0707	0.08744	0.7986	0.04322
0.7917	0.06084	0.07428	0.0732
0.08025	0.08164	0.07191	0.7662
0.06557	0.7644	0.1019	0.0681

MOTIF TAGCATCG

0.06865	0.1096	0.08117	0.7405
0.6294	0.1038	0.1485	0.1183
0.04778	0.08092	0.8117	0.05961
0.06541	0.7662	0.09512	0.07331
0.7911	0.06244	0.07915	0.06733
0.05503	0.06685	0.07811	0.8
0.06545	0.72	0.1141	0.1004

0.07723	0.08254	0.7853	0.05491

MOTIF ATAGGGTC

0.761	0.07888	0.06973	0.0904
0.05423	0.08607	0.1012	0.7585
0.698	0.09597	0.1003	0.1057
0.0645	0.07955	0.7453	0.1106
0.0856	0.09776	0.7325	0.08413
0.1113	0.06027	0.7329	0.09553
0.08337	0.064	0.06119	0.7914
0.06723	0.7818	0.06787	0.08314

MOTIF TATGCCGA

0.08364	0.1069	0.1152	0.6943
0.7102	0.06418	0.09037	0.1352
0.05967	0.07098	0.06877	0.8006
0.07864	0.1111	0.7326	0.07767
0.04838	0.7983	0.08385	0.06947
0.06607	0.807	0.06841	0.05853
0.08426	0.05472	0.8133	0.04774
0.7744	0.08792	0.06911	0.06857

MOTIF TCCCGA

0.08895	0.08534	0.1059	0.7199
0.05986	0.7574	0.12	0.06269
0.04218	0.8812	0.01951	0.05708
0.05087	0.8882	0.0206	0.04036
0.07999	0.08174	0.784	0.05422
0.8225	0.0677	0.04989	0.05988

MOTIF CGCTAT

0.06462	0.768	0.0728	0.09459
0.05776	0.08011	0.774	0.08813
0.07842	0.8105	0.07183	0.03924
0.08022	0.1313	0.05285	0.7357
0.7712	0.08947	0.1002	0.0392
0.04511	0.05907	0.04248	0.8533

MOTIF CGGTAC

0.06523	0.7911	0.07213	0.07157
0.07397	0.1001	0.7405	0.08547
0.05153	0.05031	0.8664	0.03172
0.06943	0.09503	0.06656	0.769
0.7207	0.08267	0.1269	0.06971
0.03893	0.8445	0.05513	0.06146

MOTIF ACGTAG

0.7703	0.07268	0.06108	0.09593
0.0832	0.7766	0.08373	0.05651
0.06471	0.06558	0.7988	0.07091

0.05898	0.08002	0.08195	0.779
0.7523	0.09398	0.09315	0.06056
0.03032	0.07937	0.8495	0.04076

MOTIF ATTCCG

0.808	0.07057	0.0667	0.05475
0.08219	0.1108	0.1037	0.7033
0.05602	0.09075	0.08875	0.7645
0.03973	0.8414	0.06777	0.05115
0.06438	0.8212	0.07431	0.04009
0.08573	0.0723	0.7887	0.05326

MOTIF ATTGCG

0.8071	0.08322	0.05312	0.05653
0.04479	0.06641	0.06055	0.8283
0.06535	0.04664	0.1126	0.7754
0.04072	0.07545	0.8321	0.05168
0.05923	0.7703	0.09599	0.07446
0.1065	0.09126	0.7225	0.07968

MOTIF GGGTCA

0.06351	0.07582	0.7688	0.09184
0.05944	0.05807	0.8194	0.06306
0.08098	0.06116	0.7906	0.06725
0.05546	0.06315	0.06338	0.818
0.04876	0.8186	0.06421	0.06841
0.7773	0.05947	0.112	0.0512

MOTIF GTCTAC

0.06214	0.09079	0.8074	0.03971
0.0836	0.06517	0.05918	0.7921
0.02769	0.8827	0.06047	0.02916
0.07795	0.1048	0.0311	0.7861
0.6874	0.1096	0.09896	0.1041
0.05602	0.8179	0.05835	0.06769

MOTIF GACCAG

0.05248	0.07217	0.82	0.05538
0.7795	0.06844	0.07904	0.07298
0.04743	0.8201	0.07385	0.05864
0.04701	0.8316	0.08515	0.03626
0.8047	0.06066	0.08626	0.04833
0.05584	0.06416	0.8098	0.07015

MOTIF CCGTAC

0.06088	0.8293	0.05564	0.05415
0.06607	0.7863	0.06953	0.07807
0.06032	0.06416	0.8335	0.04199
0.08377	0.07635	0.1065	0.7333
0.7655	0.09933	0.06804	0.0671

0.03005	0.8696	0.05873	0.04161

MOTIF GAACGC

0.04213	0.08051	0.8447	0.03271
0.7747	0.09009	0.06974	0.06548
0.798	0.07584	0.06738	0.05875
0.05844	0.7712	0.1093	0.06107
0.06604	0.0596	0.7874	0.08695
0.06473	0.8029	0.07641	0.05599

MOTIF ATGCCG

0.7646	0.06227	0.07664	0.09651
0.0631	0.07952	0.05678	0.8006
0.0462	0.07402	0.8222	0.05759
0.04786	0.8048	0.08853	0.05878
0.07986	0.8211	0.04988	0.04915
0.07613	0.0446	0.8323	0.047

MOTIF GTACTT

0.03628	0.0651	0.8702	0.02841
0.0644	0.06036	0.07575	0.7995
0.8007	0.06956	0.06439	0.0653
0.04708	0.8242	0.07412	0.05458
0.06511	0.05719	0.06084	0.8169
0.07395	0.07497	0.06539	0.7857

MOTIF GCGAAT

0.04166	0.08802	0.7878	0.08251
0.05574	0.7811	0.08984	0.07334
0.07911	0.0826	0.772	0.06625
0.7633	0.1022	0.09075	0.04375
0.7907	0.07848	0.06362	0.06716
0.05706	0.08047	0.1123	0.7502

MOTIF GTACAG

0.05235	0.05967	0.8416	0.04638
0.06106	0.06657	0.09406	0.7783
0.778	0.09938	0.07221	0.05042
0.04731	0.8296	0.0756	0.04753
0.7593	0.0733	0.09703	0.07039
0.05506	0.04648	0.821	0.07745

MOTIF GTTCCG

0.07791	0.07652	0.7873	0.05827
0.08771	0.1199	0.06752	0.7249
0.07878	0.06818	0.09816	0.7549
0.0257	0.8594	0.05569	0.0592
0.05304	0.78	0.09909	0.06788
0.07543	0.05335	0.7768	0.09444

MOTIF ACTGGA

0.827	0.05782	0.07632	0.03888
0.08119	0.7704	0.08374	0.06465
0.06746	0.08931	0.05481	0.7884
0.04548	0.0853	0.8109	0.05834
0.05262	0.09083	0.8101	0.04646
0.8425	0.04772	0.05151	0.05827

MOTIF TTTGCG

0.0826	0.09604	0.06096	0.7604
0.05701	0.08323	0.07668	0.7831
0.04538	0.05845	0.07688	0.8193
0.02686	0.06954	0.8444	0.05925
0.06789	0.7461	0.1131	0.07288
0.114	0.1196	0.6982	0.06813

MOTIF CCGATT

0.05492	0.8286	0.05562	0.06083
0.04843	0.8018	0.08638	0.06341
0.0763	0.078	0.7699	0.07575
0.825	0.04104	0.06399	0.06994
0.06738	0.08764	0.1521	0.6928
0.04382	0.08443	0.1053	0.7665

MOTIF CGGATT

0.05564	0.7426	0.1033	0.09846
0.04739	0.06486	0.8262	0.06153
0.03969	0.04352	0.8802	0.03655
0.7845	0.07348	0.07118	0.07079
0.06741	0.09908	0.1055	0.7281
0.06003	0.07817	0.06285	0.799

MOTIF GCGATA

0.05664	0.06857	0.7632	0.1115
0.06303	0.7752	0.09451	0.06728
0.08565	0.116	0.72	0.07843
0.8301	0.06546	0.05901	0.0454
0.06955	0.118	0.1008	0.7117
0.7372	0.07988	0.1004	0.08248

MOTIF TGCGACTCGG

0.06066	0.0806	0.1057	0.753
0.05097	0.172	0.701	0.07606
0.09911	0.7272	0.1123	0.06133
0.05293	0.07032	0.8057	0.07108
0.7314	0.1223	0.09402	0.05225
0.07661	0.7457	0.09484	0.08284
0.08051	0.0663	0.09633	0.7569
0.04224	0.8229	0.05277	0.08212
0.06187	0.07708	0.7992	0.0618

0.113	0.08797	0.7475	0.05148

MOTIF TCGCACGTAG

0.1098	0.06632	0.07323	0.7507
0.0792	0.7559	0.07934	0.08553
0.1137	0.08924	0.7496	0.04751
0.04338	0.7158	0.1734	0.06738
0.7026	0.118	0.1015	0.07787
0.06172	0.8092	0.06893	0.06014
0.08626	0.1083	0.7403	0.06517
0.09717	0.0817	0.08714	0.734
0.644	0.1217	0.1569	0.07739
0.08345	0.145	0.6858	0.08575

MOTIF TGGACACGGT

0.09423	0.1078	0.0855	0.7124
0.06302	0.07388	0.7855	0.07761
0.08759	0.09076	0.761	0.06067
0.7156	0.1334	0.06148	0.08949
0.09596	0.7393	0.1174	0.04731
0.7235	0.07022	0.09394	0.1123
0.1007	0.6914	0.09156	0.1163
0.07377	0.0907	0.7675	0.06804
0.1141	0.08491	0.7107	0.09026
0.09865	0.09915	0.1122	0.69

MOTIF ACGTGCGA

0.7365	0.06362	0.09497	0.105
0.06351	0.7585	0.1079	0.07012
0.05129	0.08584	0.8133	0.04956
0.08682	0.06943	0.1238	0.7199
0.04546	0.0997	0.81	0.04486
0.04297	0.8148	0.07725	0.06503
0.08272	0.09715	0.7484	0.07168
0.7994	0.06667	0.07659	0.05736

MOTIF GAATCGGC

0.05461	0.05234	0.8552	0.03785
0.7239	0.1229	0.0925	0.0607
0.5936	0.2061	0.1341	0.06617
0.07464	0.0924	0.1406	0.6924
0.04169	0.8434	0.06512	0.04974
0.04646	0.06428	0.8508	0.03849
0.08048	0.08419	0.784	0.05128
0.06877	0.7945	0.07776	0.05892

MOTIF TACTGCCG

0.09419	0.1374	0.143	0.6254
0.6827	0.1232	0.113	0.08106
0.05431	0.7735	0.1311	0.04107

0.04917	0.1033	0.09619	0.7513
0.03842	0.0565	0.8459	0.05914
0.06999	0.7726	0.04975	0.1076
0.05481	0.8333	0.05076	0.06114
0.06626	0.05315	0.8114	0.06923

MOTIF AAGTGCCG

0.6724	0.1366	0.09745	0.09358
0.7514	0.09633	0.08764	0.06467
0.06654	0.09938	0.7806	0.05348
0.07134	0.08109	0.1107	0.7369
0.03983	0.08232	0.827	0.05085
0.07917	0.7668	0.07438	0.07963
0.07025	0.8104	0.05007	0.06932
0.07508	0.07417	0.7708	0.07992

MOTIF CACACCGA

0.07429	0.785	0.08132	0.05941
0.7307	0.09083	0.09794	0.08051
0.06449	0.7885	0.08929	0.05769
0.7544	0.07691	0.1056	0.06308
0.08255	0.7838	0.07332	0.06038
0.06575	0.7797	0.08211	0.07245
0.08023	0.1069	0.7359	0.07698
0.7651	0.06463	0.1027	0.06755

MOTIF TGCGACTC

0.06255	0.0746	0.0933	0.7695
0.03919	0.1697	0.7185	0.07265
0.06216	0.7478	0.1041	0.08593
0.08063	0.07939	0.7592	0.08082
0.736	0.1306	0.0807	0.05271
0.05553	0.8028	0.06042	0.08122
0.09516	0.09828	0.08186	0.7247
0.05215	0.8047	0.08266	0.06047

MOTIF CCGTCGTC

0.08341	0.7138	0.08572	0.1171
0.04016	0.7836	0.1064	0.06989
0.06931	0.06275	0.8119	0.05602
0.1138	0.1548	0.08501	0.6464
0.04907	0.8141	0.08211	0.05473
0.06844	0.09463	0.7597	0.07724
0.06345	0.116	0.1454	0.6752
0.04348	0.8037	0.08815	0.06468

MOTIF ACCGCCGT

0.6208	0.06895	0.1997	0.1105
0.05658	0.771	0.1038	0.06861
0.03867	0.8252	0.03261	0.1035

0.09485	0.07458	0.7534	0.07716
0.08427	0.7143	0.07793	0.1235
0.04978	0.8808	0.02179	0.04766
0.0741	0.06039	0.7683	0.09723
0.08922	0.1733	0.1307	0.6067

MOTIF AGCTCCGC

0.6982	0.09803	0.1052	0.09856
0.06256	0.1008	0.7699	0.06673
0.0566	0.7989	0.09642	0.04808
0.07323	0.07694	0.1181	0.7318
0.05491	0.8042	0.08812	0.05272
0.07152	0.7815	0.07661	0.07032
0.08178	0.04238	0.7587	0.1171
0.08879	0.79	0.06751	0.05374

MOTIF CGACCT

0.02374	0.871	0.05176	0.05348
0.06999	0.07466	0.8129	0.04247
0.8142	0.04179	0.08288	0.06118
0.04856	0.8616	0.04258	0.04724
0.04635	0.8635	0.05638	0.03373
0.04958	0.09976	0.06049	0.7902

MOTIF GTTCCG

0.04918	0.0964	0.8085	0.04588
0.04377	0.1228	0.1358	0.6976
0.04304	0.07136	0.1077	0.7779
0.03698	0.8569	0.04687	0.0593
0.03768	0.8408	0.07384	0.04773
0.06123	0.06215	0.8009	0.07572

MOTIF ACGTCC

0.7287	0.1159	0.06078	0.09469
0.0558	0.7783	0.07011	0.09582
0.04226	0.08335	0.8198	0.05457
0.1072	0.04241	0.08966	0.7607
0.04638	0.8431	0.06944	0.04109
0.06261	0.8186	0.07465	0.0441

MOTIF GTCGGA

0.0355	0.0986	0.8319	0.03403
0.05572	0.04916	0.09907	0.796
0.03015	0.8723	0.04567	0.05189
0.0539	0.1044	0.7983	0.04342
0.02602	0.05357	0.8875	0.03291
0.7343	0.08	0.1027	0.08298

MOTIF ATACCG

0.7715	0.08891	0.07336	0.06623

0.07132	0.1795	0.1364	0.6128
0.7436	0.07405	0.1033	0.079
0.05695	0.8635	0.0361	0.04341
0.03447	0.8463	0.06891	0.05032
0.05956	0.05517	0.7835	0.1017

MOTIF AAGCGG

0.7952	0.09563	0.06884	0.04036
0.6676	0.0837	0.192	0.05667
0.06633	0.05139	0.8149	0.06739
0.05615	0.7825	0.07201	0.08937
0.07671	0.0797	0.7823	0.06128
0.04243	0.05281	0.8686	0.03618

MOTIF ACACCG

0.7204	0.1179	0.1072	0.05451
0.04239	0.8305	0.07782	0.04933
0.7567	0.06994	0.09305	0.08028
0.06532	0.8375	0.05643	0.04077
0.03912	0.8398	0.07441	0.04669
0.0763	0.05889	0.7924	0.07241

MOTIF GGACTC

0.04898	0.07679	0.8201	0.05412
0.0635	0.04571	0.8555	0.03532
0.7411	0.1011	0.1039	0.05391
0.03674	0.8615	0.08023	0.02157
0.1051	0.05632	0.06387	0.7747
0.04985	0.8173	0.07389	0.05893

MOTIF CGATTC

0.03825	0.8602	0.04167	0.0599
0.03321	0.07472	0.8567	0.03538
0.7014	0.0935	0.09578	0.1093
0.06414	0.1146	0.1537	0.6676
0.05894	0.07593	0.0949	0.7702
0.04484	0.8501	0.04141	0.06367

MOTIF ACGTAG

0.7906	0.07895	0.06018	0.07029
0.05842	0.7919	0.09191	0.05775
0.04031	0.08272	0.8219	0.05505
0.0878	0.06904	0.08166	0.7615
0.6716	0.1121	0.1555	0.06071
0.04703	0.07502	0.8382	0.03975

MOTIF TGCGAC

0.07709	0.06541	0.09693	0.7606
0.0443	0.1304	0.7905	0.03481
0.0448	0.7987	0.07306	0.08342

0.06624	0.1108	0.757	0.06597
0.8134	0.07037	0.06274	0.05347
0.03309	0.8599	0.06019	0.04685

MOTIF GCCTAT

0.06767	0.06028	0.8208	0.05123
0.06233	0.8229	0.0701	0.04467
0.063	0.8223	0.05576	0.05898
0.07739	0.1322	0.1515	0.6389
0.6358	0.1267	0.1523	0.08521
0.06363	0.08055	0.1388	0.717

MOTIF CAGCGG

0.0615	0.8574	0.05079	0.03034
0.7272	0.02706	0.1887	0.057
0.1032	0.1465	0.6861	0.06426
0.05417	0.8328	0.04777	0.06526
0.06357	0.05657	0.853	0.02688
0.06011	0.07058	0.83	0.03926

MOTIF TGACGG

0.06995	0.1446	0.1105	0.675
0.03766	0.05393	0.8765	0.03195
0.7681	0.0733	0.06963	0.08899
0.04951	0.8213	0.05872	0.07051
0.0771	0.06121	0.7994	0.0623
0.05813	0.05907	0.8201	0.06266

MOTIF GTGTCC

0.09185	0.0765	0.7735	0.05811
0.09827	0.0584	0.0537	0.7896
0.02859	0.0673	0.869	0.03517
0.05977	0.07263	0.08637	0.7812
0.03408	0.8512	0.05821	0.05648
0.08823	0.778	0.07161	0.06217

MOTIF GTAATG

0.0529	0.05349	0.8535	0.04015
0.07114	0.08731	0.06053	0.781
0.721	0.1106	0.1085	0.0599
0.8018	0.07502	0.06619	0.05703
0.04509	0.05501	0.04372	0.8562
0.03812	0.0683	0.8417	0.05185

MOTIF CGACTC

0.04717	0.8021	0.1	0.05073
0.05539	0.06477	0.8445	0.03535
0.7284	0.1294	0.07476	0.06743
0.04248	0.8098	0.09383	0.05389
0.07011	0.1227	0.09732	0.7099

0.04327	0.8464	0.06452	0.04579

MOTIF CCACGT

0.05108	0.8402	0.06958	0.03916
0.04064	0.8325	0.07398	0.05289
0.7996	0.05825	0.06606	0.07605
0.05475	0.818	0.06987	0.05734
0.06182	0.09999	0.7746	0.06361
0.08881	0.06619	0.06501	0.78

MOTIF GACCTATCGG

0.1065	0.09934	0.6838	0.1104
0.7823	0.07075	0.06699	0.07999
0.08033	0.7361	0.08961	0.09392
0.1174	0.7534	0.06231	0.06686
0.0949	0.1228	0.1371	0.6452
0.7241	0.08594	0.09429	0.0957
0.09207	0.06409	0.05954	0.7843
0.09096	0.6911	0.08477	0.1332
0.09178	0.1543	0.6572	0.09669
0.1256	0.07273	0.7317	0.07001

MOTIF AAGCCATTGA

0.7503	0.05871	0.0906	0.1003
0.7235	0.05303	0.1278	0.09563
0.1094	0.06668	0.6923	0.1316
0.125	0.6725	0.09924	0.1032
0.1355	0.7024	0.06099	0.1011
0.7618	0.08377	0.06053	0.09396
0.1158	0.09284	0.07719	0.7142
0.1049	0.08885	0.09121	0.7151
0.09012	0.0842	0.6849	0.1408
0.7389	0.06859	0.0855	0.107

MOTIF GAACAAAGGT

0.1122	0.09912	0.7064	0.08231
0.7816	0.0609	0.08236	0.0751
0.745	0.06367	0.07718	0.1141
0.1484	0.6536	0.09392	0.1041
0.7665	0.0702	0.06782	0.09551
0.7975	0.06029	0.07419	0.06803
0.7713	0.05703	0.0991	0.07257
0.09903	0.05693	0.719	0.1251
0.119	0.09215	0.6869	0.102
0.1085	0.0948	0.09584	0.7008

MOTIF GCTTTAGTGA

0.1343	0.07859	0.6975	0.08962
0.1287	0.6802	0.07043	0.1208
0.09179	0.09877	0.05426	0.7552

0.06339	0.05842	0.0544	0.8238
0.09427	0.06342	0.0659	0.7764
0.662	0.1095	0.09948	0.1289
0.09431	0.07962	0.7158	0.1103
0.09116	0.0512	0.07375	0.7839
0.11	0.1053	0.6578	0.1268
0.7374	0.06858	0.1054	0.08859

MOTIF GCTTCGAGTC

0.09287	0.1073	0.6779	0.1219
0.1454	0.6685	0.09395	0.09216
0.08207	0.04625	0.05695	0.8147
0.09378	0.1357	0.06212	0.7084
0.07558	0.6825	0.1427	0.09915
0.08451	0.08838	0.7341	0.09301
0.7703	0.08047	0.0739	0.07537
0.06208	0.1491	0.6511	0.1377
0.08358	0.08801	0.04801	0.7804
0.05896	0.6432	0.1273	0.1705

MOTIF AGTTCAACCC

0.7534	0.07055	0.08037	0.09568
0.103	0.07365	0.7379	0.08546
0.1151	0.0749	0.05895	0.751
0.07638	0.0995	0.05264	0.7715
0.08008	0.7121	0.1062	0.1017
0.7864	0.07808	0.0605	0.07501
0.7675	0.08055	0.05296	0.09902
0.08485	0.7573	0.07487	0.08298
0.09184	0.6879	0.09402	0.1262
0.09221	0.7095	0.05933	0.1389

MOTIF CGCCAGTA

0.07593	0.737	0.1155	0.07157
0.1233	0.05548	0.737	0.08427
0.08356	0.7712	0.07174	0.07346
0.0748	0.8086	0.06777	0.04887
0.7944	0.06378	0.0621	0.07975
0.07817	0.09127	0.7533	0.07721
0.1079	0.08199	0.08391	0.7262
0.7158	0.1195	0.06889	0.09585

MOTIF TGATTACA

0.105	0.08253	0.09687	0.7156
0.07017	0.06852	0.7763	0.08504
0.7587	0.09195	0.05946	0.08994
0.08055	0.06766	0.06464	0.7871
0.07594	0.09908	0.0923	0.7327
0.7256	0.1056	0.08081	0.08794
0.1046	0.7077	0.1065	0.08114

0.8325	0.05358	0.04487	0.06901

MOTIF GATAGGTC

0.1199	0.0602	0.7618	0.05808
0.7308	0.07209	0.07713	0.12
0.08391	0.03513	0.1006	0.7803
0.7573	0.06889	0.09532	0.07848
0.07382	0.04145	0.7347	0.15
0.06493	0.07772	0.7852	0.07218
0.0769	0.06891	0.07772	0.7765
0.08733	0.7111	0.09534	0.1062

MOTIF GCGTGAGA

0.1151	0.07486	0.7178	0.09227
0.1554	0.6383	0.105	0.1013
0.1101	0.08356	0.7215	0.08481
0.1873	0.06854	0.1086	0.6356
0.08281	0.08515	0.7565	0.07552
0.7569	0.07347	0.09217	0.07742
0.1032	0.0669	0.7734	0.05648
0.7851	0.07407	0.0798	0.06102

MOTIF TCTAAGCG

0.04808	0.09651	0.06015	0.7953
0.07585	0.7768	0.08858	0.05873
0.05761	0.08599	0.09038	0.766
0.8076	0.04693	0.07064	0.07484
0.7961	0.0546	0.07168	0.07758
0.08984	0.09615	0.7048	0.1092
0.1259	0.6346	0.1108	0.1287
0.1156	0.1041	0.6567	0.1236

MOTIF AGCCATTT

0.7672	0.04708	0.08801	0.09774
0.1073	0.08123	0.7074	0.104
0.1208	0.7028	0.07371	0.1026
0.1067	0.7331	0.0635	0.09671
0.7849	0.07712	0.04412	0.09382
0.08003	0.08912	0.0523	0.7786
0.07049	0.1044	0.07634	0.7488
0.05983	0.06494	0.08599	0.7892

MOTIF GGTATTCG

0.04753	0.0563	0.7944	0.1018
0.1185	0.09361	0.7156	0.07238
0.08389	0.06189	0.08291	0.7713
0.6978	0.09008	0.1114	0.1007
0.06676	0.06178	0.06903	0.8024
0.03901	0.08457	0.04674	0.8297
0.09214	0.6436	0.1532	0.1111

0.1022	0.07204	0.7291	0.09663

MOTIF GCTTTAGT

0.1074	0.06384	0.7321	0.09665
0.1047	0.6952	0.07595	0.1242
0.07536	0.09112	0.04809	0.7854
0.04881	0.05594	0.06108	0.8342
0.07629	0.06325	0.04901	0.8115
0.716	0.06521	0.08779	0.131
0.1041	0.08668	0.7043	0.1048
0.06781	0.05409	0.06105	0.8171

MOTIF TGTATTCC

0.04416	0.05076	0.06323	0.8419
0.08568	0.1027	0.6649	0.1467
0.08529	0.06624	0.08466	0.7638
0.6857	0.1232	0.09244	0.09861
0.1112	0.06899	0.07836	0.7414
0.04519	0.05649	0.08609	0.8122
0.07869	0.7213	0.07381	0.1262
0.101	0.7137	0.07335	0.1119

MOTIF CGATTACA

0.1302	0.5995	0.1166	0.1536
0.111	0.1177	0.6607	0.1106
0.7759	0.08017	0.08776	0.05616
0.09491	0.1019	0.05175	0.7514
0.04893	0.09009	0.05506	0.8059
0.7459	0.09051	0.08276	0.08078
0.09762	0.725	0.101	0.07642
0.8394	0.06364	0.05018	0.04682

MOTIF CAGTTACG

0.08336	0.7617	0.07096	0.08396
0.8235	0.04202	0.05738	0.07707
0.08771	0.08013	0.729	0.1032
0.08589	0.0724	0.07382	0.7679
0.05734	0.05965	0.1179	0.7651
0.7106	0.09028	0.06983	0.1293
0.1205	0.6524	0.1139	0.1133
0.1407	0.1111	0.6565	0.09172

MOTIF GCTAATCT

0.1113	0.08308	0.6527	0.1529
0.1189	0.709	0.06466	0.1074
0.1061	0.07817	0.07693	0.7388
0.755	0.06691	0.069	0.1091
0.7466	0.0829	0.05466	0.1158
0.08402	0.08037	0.06467	0.7709
0.1042	0.7173	0.06625	0.1123

0.08997	0.06432	0.05191	0.7938

MOTIF TCCTAACC

0.07605	0.1086	0.05744	0.7579
0.05802	0.7605	0.0704	0.1111
0.07284	0.7953	0.04436	0.08754
0.1287	0.08904	0.07866	0.7036
0.7102	0.1358	0.06969	0.08433
0.7111	0.09379	0.09339	0.1017
0.07381	0.7629	0.06991	0.09337
0.09111	0.7171	0.0511	0.1407

MOTIF TTCGAG

0.06648	0.02182	0.06887	0.8428
0.05556	0.09221	0.04577	0.8065
0.102	0.6645	0.1153	0.1182
0.06688	0.04904	0.7722	0.1119
0.8203	0.05419	0.05739	0.06814
0.0275	0.05638	0.8554	0.0607

MOTIF TAAGCG

0.0591	0.06424	0.07548	0.8012
0.806	0.05268	0.06887	0.07249
0.8545	0.04	0.05971	0.04575
0.07676	0.08082	0.766	0.07639
0.1337	0.6165	0.1325	0.1172
0.07547	0.0864	0.7466	0.09153

MOTIF TTACGG

0.06005	0.05926	0.06541	0.8153
0.05457	0.04651	0.08292	0.816
0.7885	0.0806	0.06475	0.06615
0.0844	0.6465	0.137	0.132
0.08669	0.04689	0.7596	0.1068
0.04335	0.0524	0.8366	0.06763

MOTIF CCTACC

0.05603	0.834	0.03927	0.07073
0.06374	0.8478	0.0365	0.05191
0.09412	0.0831	0.05732	0.7655
0.7181	0.1117	0.06359	0.1067
0.09066	0.767	0.05354	0.08881
0.08292	0.7774	0.04608	0.09359

MOTIF CGAATC

0.1002	0.7485	0.07866	0.07264
0.1162	0.07447	0.7149	0.09445
0.8657	0.04673	0.04696	0.04065
0.7643	0.08491	0.08014	0.07066
0.05727	0.06686	0.05891	0.817

0.06913 0.8192 0.04582 0.06589

MOTIF GGTTCA
0.1071 0.04119 0.7638 0.0879
0.07048 0.06628 0.7937 0.06955
0.05434 0.05799 0.06452 0.8231
0.05153 0.06944 0.09076 0.7883
0.07609 0.7784 0.08038 0.06517
0.8538 0.0345 0.06508 0.04666

MOTIF CCTATC
0.04243 0.8276 0.0538 0.07614
0.0832 0.7958 0.03751 0.08354
0.07014 0.06775 0.06242 0.7997
0.7173 0.1167 0.06431 0.1016
0.08381 0.07561 0.04974 0.7909
0.04771 0.8169 0.04212 0.0933

MOTIF CTCAGT
0.1097 0.7709 0.06153 0.05783
0.07237 0.07013 0.04784 0.8097
0.07417 0.7812 0.06042 0.08424
0.8096 0.06088 0.05264 0.0769
0.0827 0.07786 0.71 0.1294
0.07853 0.04192 0.05186 0.8277

MOTIF ATCTCG
0.786 0.09047 0.05548 0.06806
0.07772 0.05619 0.05193 0.8142
0.05218 0.8176 0.05763 0.07257
0.05668 0.05447 0.04066 0.8482
0.0787 0.7424 0.08539 0.09347
0.1034 0.1014 0.6577 0.1375

MOTIF TAACGT
0.06212 0.05163 0.0432 0.843
0.7931 0.07687 0.06005 0.06993
0.8201 0.07675 0.05118 0.05196
0.1438 0.6456 0.1002 0.1105
0.1454 0.08358 0.6373 0.1338
0.07868 0.0526 0.05651 0.8122

MOTIF ATCGGA
0.7961 0.08884 0.07298 0.04204
0.05619 0.08383 0.06715 0.7928
0.1036 0.6909 0.08065 0.1249
0.1186 0.06247 0.7116 0.1073
0.058 0.05787 0.8436 0.04058
0.8477 0.06089 0.03845 0.05293

MOTIF CTTGCG

0.05429	0.8329	0.05757	0.05525
0.04619	0.06059	0.0551	0.8381
0.05747	0.05042	0.08935	0.8028
0.0506	0.07521	0.8007	0.0735
0.07595	0.7308	0.09833	0.0949
0.09949	0.1119	0.6621	0.1265

MOTIF GCCAGT

0.06239	0.07122	0.7893	0.07708
0.07292	0.7849	0.07904	0.06309
0.08526	0.8175	0.04975	0.04753
0.8362	0.04081	0.05675	0.06621
0.06664	0.1062	0.7136	0.1136
0.07205	0.06751	0.07755	0.7829

MOTIF ACGGGT

0.7842	0.07719	0.05943	0.0792
0.1195	0.6384	0.1341	0.108
0.08679	0.02601	0.8125	0.07476
0.09773	0.0167	0.8315	0.05409
0.06783	0.05904	0.7942	0.0789
0.07336	0.04689	0.06317	0.8166

MOTIF CAGTTA

0.08512	0.7699	0.07081	0.07412
0.8532	0.03744	0.05382	0.05556
0.07066	0.07786	0.7374	0.1141
0.09285	0.04448	0.04681	0.8159
0.07068	0.05883	0.07355	0.7969
0.7767	0.0754	0.05374	0.09416

MOTIF CCCAGT

0.06435	0.7658	0.08916	0.08073
0.07744	0.7773	0.05089	0.09439
0.09438	0.7635	0.07447	0.06769
0.8352	0.05537	0.03853	0.07086
0.1057	0.07898	0.7333	0.08198
0.06159	0.07937	0.07245	0.7866

MOTIF GATACC

0.1265	0.09048	0.703	0.08005
0.843	0.05313	0.029	0.07484
0.1337	0.07087	0.1011	0.6943
0.7307	0.07453	0.08909	0.1057
0.06908	0.7995	0.0891	0.04231
0.06367	0.8262	0.05352	0.05659

MOTIF GACCTA

0.07146	0.07005	0.8035	0.05497

0.7857	0.08299	0.06629	0.065
0.118	0.7366	0.06328	0.08218
0.1098	0.7982	0.03249	0.05951
0.09186	0.07765	0.05954	0.7709
0.8096	0.05865	0.06133	0.07043

MOTIF TGCGAA

0.06582	0.06289	0.09164	0.7797
0.06792	0.05644	0.842	0.03365
0.177	0.6154	0.0832	0.1244
0.1509	0.106	0.6514	0.09166
0.8778	0.01806	0.06129	0.04288
0.8218	0.05447	0.05512	0.06862

MOTIF CATCCTTAGT

0.1101	0.7264	0.08696	0.07659
0.8101	0.05329	0.05401	0.08259
0.1132	0.04043	0.04666	0.7997
0.1083	0.7244	0.07849	0.08884
0.08184	0.7666	0.0297	0.1219
0.05661	0.05536	0.02717	0.8609
0.09973	0.05357	0.04926	0.7974
0.793	0.07908	0.06359	0.0643
0.08704	0.09629	0.7296	0.08702
0.0798	0.07467	0.04061	0.8049

MOTIF GGATACCCAT

0.1116	0.1254	0.6213	0.1417
0.08744	0.0967	0.7344	0.08147
0.7474	0.08442	0.09784	0.0703
0.1206	0.06342	0.08103	0.735
0.7953	0.06981	0.07349	0.06145
0.0846	0.769	0.07793	0.06846
0.09984	0.7867	0.05759	0.05585
0.08543	0.7842	0.04737	0.083
0.7777	0.1021	0.04832	0.07187
0.08932	0.08438	0.06109	0.7652

MOTIF AGCAAGTCGT

0.7498	0.1002	0.07023	0.07983
0.1408	0.1005	0.6677	0.09095
0.1135	0.7272	0.08999	0.06935
0.8116	0.06761	0.05553	0.06525
0.7736	0.07057	0.07838	0.07747
0.07081	0.0495	0.8222	0.05744
0.1	0.05752	0.1037	0.7387
0.08522	0.7538	0.07488	0.08609
0.1084	0.1194	0.6779	0.0942
0.1251	0.07669	0.06394	0.7342

MOTIF ATGCTCGA

0.7766	0.05061	0.08807	0.08473
0.08828	0.06167	0.06505	0.785
0.05524	0.07667	0.8017	0.06642
0.07766	0.7663	0.06943	0.08659
0.07198	0.06483	0.04694	0.8162
0.09784	0.713	0.06879	0.1204
0.09927	0.0864	0.7473	0.06699
0.7786	0.06897	0.07642	0.07597

MOTIF CTAAGGAT

0.09035	0.725	0.09072	0.09389
0.05674	0.06473	0.08897	0.7896
0.7957	0.055	0.05242	0.09687
0.8083	0.03786	0.09583	0.05796
0.1291	0.03931	0.7606	0.071
0.09295	0.0907	0.6983	0.118
0.7583	0.06277	0.05349	0.1254
0.07988	0.06702	0.0472	0.8059

MOTIF CAAGTCGT

0.07558	0.7811	0.06701	0.0763
0.8099	0.04568	0.07189	0.07256
0.803	0.06916	0.05352	0.07433
0.06166	0.05118	0.8138	0.07336
0.07765	0.05667	0.08534	0.7803
0.06066	0.7541	0.09037	0.09491
0.1157	0.09195	0.7098	0.08251
0.1008	0.04842	0.06307	0.7877

MOTIF TCTTGCGT

0.08609	0.09146	0.07249	0.75
0.06229	0.7765	0.08827	0.07291
0.06762	0.06961	0.0693	0.7935
0.05934	0.05591	0.05568	0.8291
0.08735	0.08353	0.7372	0.09188
0.0811	0.6919	0.1213	0.1057
0.1272	0.06939	0.6996	0.1037
0.08715	0.06146	0.06712	0.7843

MOTIF GGTACCCA

0.06101	0.08716	0.7827	0.06914
0.1616	0.09547	0.6698	0.07313
0.08778	0.09003	0.06209	0.7601
0.7716	0.05956	0.08415	0.08468
0.09578	0.722	0.07907	0.1032
0.05664	0.8446	0.04189	0.05683
0.08492	0.7756	0.08845	0.05101
0.8096	0.07981	0.05068	0.05986

MOTIF CATTCGGG

0.0712	0.7309	0.09608	0.1018
0.7447	0.08841	0.07072	0.09619
0.07468	0.077	0.09813	0.7502
0.04115	0.0783	0.08211	0.7984
0.06421	0.6932	0.1182	0.1243
0.0483	0.04188	0.8328	0.07699
0.07773	0.0554	0.7884	0.07845
0.08985	0.0916	0.7368	0.08177

MOTIF ACTTAAGA

0.8513	0.05543	0.05567	0.03758
0.1036	0.6896	0.06127	0.1456
0.09781	0.06197	0.07452	0.7657
0.1317	0.07026	0.07364	0.7244
0.7992	0.05979	0.06402	0.07701
0.7578	0.03892	0.1202	0.0831
0.112	0.1038	0.6842	0.09999
0.8584	0.06209	0.04813	0.03134

MOTIF GTTTCGGG

0.08964	0.08831	0.7038	0.1182
0.08803	0.1088	0.1094	0.6937
0.07724	0.08072	0.0859	0.7561
0.06214	0.05083	0.07554	0.8115
0.1072	0.6479	0.1109	0.134
0.07651	0.07224	0.7732	0.07801
0.09121	0.05358	0.7841	0.07114
0.06561	0.1055	0.7314	0.09748

MOTIF GGGTAT

0.05982	0.05563	0.8257	0.05885
0.05441	0.03547	0.841	0.06912
0.05014	0.05021	0.8376	0.06207
0.04524	0.05226	0.05791	0.8446
0.7579	0.06235	0.06211	0.1177
0.06732	0.07394	0.05937	0.7994

MOTIF CCGGTT

0.08937	0.7065	0.09648	0.1077
0.05941	0.7926	0.09721	0.05076
0.06175	0.05022	0.8379	0.05017
0.08354	0.05125	0.7819	0.08327
0.05882	0.04365	0.05058	0.8469
0.06027	0.06153	0.05438	0.8238

MOTIF AAAGCG

0.8213	0.07926	0.05729	0.04217
0.8649	0.03804	0.05114	0.04592
0.8297	0.04249	0.07616	0.0517

0.07663	0.1571	0.6845	0.08184
0.08421	0.765	0.07645	0.07434
0.1099	0.07396	0.7248	0.09131

MOTIF CGTAAG
0.09004	0.7097	0.09449	0.1058
0.09847	0.09666	0.7636	0.04129
0.08121	0.1186	0.09001	0.7102
0.8143	0.0649	0.06412	0.05664
0.8775	0.02363	0.05446	0.04438
0.05773	0.0542	0.8303	0.05777

MOTIF TTGCGG
0.0623	0.06051	0.04983	0.8273
0.06543	0.03658	0.07502	0.823
0.03985	0.05577	0.7794	0.125
0.07962	0.721	0.1144	0.08501
0.04845	0.08083	0.7852	0.08551
0.04726	0.05757	0.8004	0.09473

MOTIF TACCGA
0.1048	0.06219	0.08614	0.7469
0.7843	0.06223	0.06798	0.08553
0.03624	0.8837	0.02931	0.05075
0.05135	0.843	0.04835	0.05725
0.08717	0.1708	0.6791	0.06286
0.8314	0.05534	0.05482	0.05843

MOTIF TTCCGA
0.05563	0.06786	0.07594	0.8006
0.09001	0.05899	0.06734	0.7837
0.0596	0.8276	0.03578	0.07703
0.09544	0.7746	0.05708	0.07291
0.07207	0.07119	0.745	0.1118
0.8386	0.05107	0.04773	0.06258

MOTIF ATGCGT
0.8075	0.04607	0.05793	0.08853
0.05265	0.01696	0.06137	0.869
0.05027	0.04914	0.833	0.06755
0.09816	0.6139	0.1696	0.1183
0.03446	0.06946	0.755	0.1411
0.04506	0.04423	0.05885	0.8519

MOTIF GCACTA
0.09527	0.1016	0.721	0.08212
0.07668	0.7655	0.09851	0.05929
0.784	0.08157	0.0564	0.07802
0.08284	0.7832	0.0553	0.07869
0.06584	0.07359	0.09682	0.7637

0.8076	0.06636	0.06099	0.06505

MOTIF AATCGG

0.8218	0.06208	0.06836	0.04771
0.7604	0.08205	0.08616	0.07142
0.07406	0.09553	0.04854	0.7819
0.0974	0.7056	0.08816	0.1088
0.05094	0.05617	0.8469	0.04599
0.0759	0.05994	0.8083	0.05584

MOTIF AGCGTA

0.7989	0.05537	0.06597	0.07975
0.07276	0.07793	0.7695	0.07978
0.08426	0.689	0.1266	0.1002
0.1091	0.08125	0.6977	0.1119
0.0715	0.06424	0.05562	0.8086
0.8029	0.06349	0.06634	0.06724

MOTIF GACGTT

0.02387	0.05438	0.8506	0.07115
0.7611	0.06452	0.05463	0.1198
0.09013	0.7233	0.09056	0.09606
0.06044	0.1125	0.7499	0.07716
0.07163	0.0578	0.0827	0.7879
0.08044	0.01922	0.07881	0.8215

MOTIF GTTAAG

0.07397	0.03164	0.768	0.1264
0.04295	0.06588	0.077	0.8142
0.05928	0.05006	0.09164	0.799
0.7533	0.05596	0.1054	0.08542
0.8353	0.04463	0.05373	0.06636
0.06035	0.0157	0.8522	0.07176

MOTIF ACTAAG

0.8373	0.05781	0.05084	0.0541
0.07317	0.8162	0.05484	0.05581
0.0687	0.06271	0.06811	0.8005
0.8258	0.03952	0.04465	0.09007
0.8535	0.0341	0.06665	0.0457
0.1292	0.04599	0.7628	0.06205

MOTIF ATTCCTGCCT

0.7264	0.08706	0.0792	0.1073
0.07341	0.08814	0.07761	0.7608
0.04383	0.04571	0.07367	0.8368
0.04961	0.7549	0.06859	0.1269
0.09122	0.7112	0.08859	0.109
0.06731	0.07214	0.0277	0.8328
0.1218	0.1003	0.6288	0.1492

0.05555	0.764	0.07848	0.1019
0.1009	0.7146	0.08965	0.09482
0.09193	0.04264	0.04716	0.8183

MOTIF ATTTGGCCAT

0.7153	0.09348	0.09834	0.09283
0.1154	0.1005	0.07593	0.7082
0.09407	0.06399	0.08687	0.7551
0.0553	0.05138	0.07516	0.8182
0.07131	0.05344	0.7447	0.1305
0.1053	0.06851	0.6894	0.1368
0.08662	0.6842	0.1078	0.1214
0.1194	0.6976	0.04453	0.1384
0.7797	0.07978	0.07472	0.06585
0.09048	0.06072	0.06026	0.7885

MOTIF AGTGCCAAGC

0.7628	0.076	0.05388	0.1073
0.09835	0.09595	0.683	0.1227
0.1172	0.07791	0.09741	0.7075
0.1016	0.09884	0.734	0.06553
0.07037	0.7501	0.1083	0.0713
0.1149	0.7238	0.06594	0.09535
0.7612	0.06156	0.0869	0.0903
0.7098	0.05624	0.1143	0.1197
0.1356	0.07955	0.7	0.08483
0.1324	0.701	0.09024	0.07641

MOTIF ATTGGCAATT

0.6917	0.09302	0.08874	0.1265
0.05929	0.04855	0.05304	0.8391
0.08108	0.07378	0.08225	0.7629
0.1143	0.06937	0.7126	0.1037
0.1163	0.1277	0.6568	0.09921
0.1155	0.689	0.07684	0.1186
0.8066	0.07409	0.04476	0.07457
0.7518	0.0949	0.05471	0.09859
0.07865	0.08399	0.07621	0.7611
0.111	0.08924	0.0966	0.7032

MOTIF AGTGCAGTAT

0.7223	0.07977	0.09091	0.107
0.1295	0.08964	0.6495	0.1314
0.1223	0.04612	0.05716	0.7745
0.1434	0.05991	0.693	0.1037
0.1276	0.6517	0.1238	0.09683
0.7856	0.0541	0.06633	0.09395
0.1289	0.05933	0.7001	0.1117
0.1036	0.1193	0.08852	0.6885
0.7557	0.07244	0.09043	0.08148

0.08743	0.07973	0.08383	0.749

MOTIF TTTGCGCA

0.06006	0.09988	0.06446	0.7756
0.04944	0.05464	0.05903	0.8369
0.04947	0.03424	0.09108	0.8252
0.0558	0.03989	0.8084	0.09593
0.1062	0.6564	0.104	0.1333
0.0842	0.09412	0.7281	0.0936
0.08482	0.7919	0.06513	0.05817
0.7872	0.0755	0.05185	0.08542

MOTIF ACAGTATA

0.7448	0.08325	0.1123	0.05961
0.1429	0.6518	0.08515	0.1202
0.8389	0.03652	0.03976	0.08477
0.1069	0.087	0.7224	0.0837
0.1176	0.07378	0.0906	0.7181
0.7906	0.06705	0.07718	0.06518
0.08501	0.06872	0.07298	0.7733
0.7265	0.07827	0.0787	0.1166

MOTIF TTGGCAAT

0.05977	0.04877	0.06465	0.8268
0.07005	0.0752	0.05934	0.7954
0.0892	0.06141	0.7588	0.09059
0.08972	0.1088	0.7288	0.07276
0.1012	0.7308	0.08461	0.08344
0.7728	0.07587	0.04399	0.1073
0.7511	0.1091	0.06289	0.0769
0.07935	0.06258	0.07979	0.7783

MOTIF CCCTACGC

0.08818	0.7706	0.06448	0.07677
0.06369	0.8083	0.06397	0.06404
0.06642	0.8194	0.04418	0.07001
0.0647	0.1068	0.08665	0.7419
0.6598	0.1158	0.1198	0.1046
0.06736	0.7558	0.07612	0.1007
0.08966	0.07914	0.7226	0.1086
0.07325	0.8066	0.06191	0.05827

MOTIF GAATGCTC

0.1016	0.08155	0.7016	0.1153
0.7579	0.1112	0.07628	0.05461
0.8001	0.06604	0.05768	0.07617
0.06498	0.02734	0.08316	0.8245
0.06572	0.07894	0.7669	0.08846
0.08048	0.7767	0.06735	0.07547
0.08361	0.1006	0.07467	0.7412

0.1158	0.6508	0.1197	0.1137

MOTIF ACGCAGGA

0.7677	0.08214	0.07442	0.07573
0.1009	0.6292	0.137	0.1329
0.1004	0.09151	0.7376	0.07052
0.1193	0.7329	0.09564	0.0522
0.8383	0.06356	0.04676	0.05139
0.0666	0.0641	0.7634	0.1059
0.08201	0.09895	0.7645	0.05457
0.8099	0.07178	0.07017	0.04813

MOTIF TCAATGTG

0.07662	0.06126	0.118	0.7441
0.1163	0.6617	0.1161	0.1059
0.7532	0.108	0.05297	0.08582
0.7885	0.05156	0.06428	0.09571
0.1178	0.07625	0.09295	0.713
0.07851	0.06873	0.7496	0.1032
0.06882	0.06935	0.06535	0.7965
0.08206	0.07695	0.7352	0.1058

MOTIF TAAGGCGT

0.1231	0.09816	0.1395	0.6392
0.7594	0.07455	0.08783	0.07819
0.8115	0.05913	0.05642	0.07298
0.05327	0.04268	0.8063	0.09772
0.04384	0.0731	0.7777	0.1054
0.1261	0.7302	0.06088	0.08284
0.1302	0.08639	0.6617	0.1217
0.07557	0.06384	0.06968	0.7909

MOTIF GTGCAGTA

0.1109	0.09114	0.6935	0.1044
0.1115	0.07612	0.06346	0.749
0.06867	0.04751	0.8142	0.06959
0.09475	0.7188	0.1024	0.08409
0.7793	0.06518	0.07704	0.07852
0.1054	0.09099	0.7207	0.08286
0.1146	0.1333	0.07686	0.6752
0.7632	0.05177	0.09941	0.08561

MOTIF CGCAAA

0.1132	0.6619	0.09102	0.1339
0.1163	0.08694	0.7054	0.0914
0.04753	0.8789	0.0384	0.03518
0.8456	0.07164	0.04344	0.03933
0.8392	0.06087	0.06237	0.03759
0.8113	0.05911	0.07945	0.05012

MOTIF TGTACG

0.05653	0.0177	0.05231	0.8735
0.05238	0.08385	0.8149	0.04891
0.05517	0.1058	0.07413	0.7649
0.76	0.07738	0.08658	0.07606
0.08574	0.6999	0.07451	0.1399
0.04253	0.102	0.7417	0.1138

MOTIF TGCGAA

0.06467	0.04623	0.06549	0.8236
0.05953	0.05878	0.854	0.02772
0.07282	0.7538	0.07443	0.09891
0.148	0.1544	0.6086	0.08903
0.869	0.0337	0.04226	0.05506
0.8092	0.04933	0.06606	0.07545

MOTIF CCTGTA

0.06316	0.8035	0.07654	0.05677
0.1105	0.724	0.06173	0.1038
0.07045	0.04022	0.03838	0.8509
0.08284	0.0699	0.7584	0.0889
0.08305	0.08356	0.08359	0.7498
0.8091	0.05699	0.06289	0.07106

MOTIF CCTACG

0.04801	0.7958	0.07578	0.08044
0.04959	0.8995	0.01714	0.03377
0.07697	0.07302	0.05564	0.7944
0.6757	0.0986	0.1037	0.1219
0.0783	0.8023	0.02825	0.09114
0.06491	0.06153	0.7829	0.09061

MOTIF CAGTAT

0.0983	0.7325	0.07529	0.09388
0.8602	0.03511	0.05104	0.05365
0.08931	0.0555	0.7713	0.08388
0.09243	0.07304	0.08294	0.7516
0.8246	0.05661	0.04258	0.07621
0.06155	0.06065	0.05591	0.8219

MOTIF ACGTTC

0.8217	0.05679	0.05299	0.0685
0.1234	0.63	0.1247	0.1219
0.1074	0.1229	0.6765	0.09318
0.05705	0.05736	0.04902	0.8366
0.05323	0.04982	0.06306	0.8339
0.04258	0.8307	0.06336	0.06335

MOTIF GGCATA

0.07921	0.03835	0.7881	0.0943

0.06349	0.06877	0.795	0.07276
0.09944	0.7967	0.05514	0.04872
0.8434	0.05168	0.0549	0.05006
0.09532	0.07967	0.0774	0.7476
0.7594	0.05899	0.09078	0.09087

MOTIF CCGTTA

0.06164	0.7864	0.07398	0.07794
0.08319	0.7387	0.04964	0.1285
0.1404	0.1169	0.5923	0.1504
0.05715	0.06473	0.06411	0.814
0.05482	0.07535	0.08447	0.7854
0.6677	0.08349	0.1674	0.08143

MOTIF AATGCG

0.8174	0.07677	0.06141	0.04442
0.8284	0.05783	0.05337	0.06041
0.04273	0.03738	0.09968	0.8202
0.06582	0.06654	0.8108	0.05687
0.08869	0.7084	0.07664	0.1263
0.1417	0.1045	0.6227	0.1311

MOTIF TACAGC

0.05926	0.08744	0.05204	0.8013
0.7415	0.07203	0.1054	0.0811
0.07533	0.7549	0.08109	0.08872
0.7797	0.05469	0.07136	0.09428
0.1009	0.07633	0.7674	0.0553
0.05633	0.7916	0.09068	0.06142

MOTIF ACTGGG

0.78	0.0696	0.08796	0.06245
0.08484	0.7555	0.07944	0.08023
0.07367	0.04399	0.04755	0.8348
0.06925	0.07414	0.7294	0.1272
0.08457	0.0627	0.7847	0.068
0.08897	0.0776	0.7695	0.06389

MOTIF AAGGCG

0.7954	0.05863	0.06745	0.07849
0.8042	0.07765	0.0577	0.06044
0.03824	0.03438	0.8606	0.06674
0.04176	0.05201	0.8459	0.06036
0.1133	0.7158	0.0659	0.105
0.123	0.08254	0.7039	0.09061

MOTIF ATAGCC

0.7906	0.08147	0.0656	0.06231
0.0845	0.05943	0.07322	0.7829
0.7338	0.05293	0.1134	0.09991

0.06154	0.06277	0.8149	0.06079
0.05004	0.8076	0.07534	0.06705
0.1155	0.7844	0.0352	0.06492

MOTIF GGCAAT

0.09247	0.04348	0.7648	0.09921
0.05555	0.07058	0.8183	0.05556
0.06825	0.7968	0.07729	0.05768
0.788	0.09108	0.04314	0.07774
0.7903	0.07022	0.06173	0.07771
0.08067	0.07811	0.07924	0.762

MOTIF CGAATA

0.1087	0.6872	0.1084	0.09569
0.1635	0.1009	0.6721	0.06353
0.871	0.04183	0.04485	0.04227
0.8215	0.06202	0.06619	0.05026
0.05229	0.05564	0.05468	0.8374
0.82	0.06284	0.06586	0.0513

MOTIF GGTAAG

0.09136	0.02956	0.8088	0.07033
0.06836	0.06751	0.7996	0.06457
0.09607	0.1164	0.0893	0.6983
0.7932	0.06001	0.05966	0.08719
0.8028	0.04368	0.06089	0.09264
0.07553	0.06857	0.7711	0.08479

MOTIF CGCAGA

0.1218	0.6411	0.09086	0.1462
0.06472	0.05766	0.8307	0.04693
0.05974	0.8226	0.04505	0.07261
0.8002	0.08687	0.07367	0.03927
0.1019	0.07223	0.7804	0.04538
0.7562	0.09339	0.09219	0.05819

MOTIF ACTACC

0.8264	0.05573	0.0673	0.05058
0.0852	0.7776	0.06331	0.07394
0.08361	0.0849	0.05983	0.7717
0.784	0.07304	0.07787	0.06508
0.07666	0.777	0.07267	0.07364
0.0787	0.7828	0.04315	0.09533

MOTIF AGCCGT

0.7144	0.09991	0.08512	0.1006
0.04533	0.04012	0.8613	0.0533
0.03769	0.7636	0.1082	0.09052
0.09102	0.7713	0.04104	0.09667
0.1105	0.07172	0.6932	0.1246

0.06324	0.04833	0.0448	0.8436

MOTIF TACCTC

0.06853	0.06035	0.04288	0.8282
0.6735	0.1115	0.09906	0.1159
0.06184	0.8289	0.04981	0.05947
0.05338	0.8419	0.03067	0.07406
0.07849	0.05748	0.05165	0.8124
0.08266	0.7382	0.07682	0.1023

MOTIF TTCAGT

0.03906	0.06166	0.04893	0.8504
0.0492	0.06139	0.08196	0.8074
0.07705	0.7805	0.07461	0.0678
0.8354	0.0476	0.04769	0.06936
0.09692	0.09107	0.6975	0.1145
0.06114	0.06595	0.06663	0.8063

MOTIF CCACTGCACC

0.06888	0.785	0.07134	0.07474
0.1164	0.7687	0.05175	0.06316
0.6618	0.109	0.1251	0.1041
0.0435	0.8121	0.1016	0.04274
0.06652	0.08318	0.09475	0.7555
0.04242	0.07226	0.7668	0.1186
0.08051	0.8098	0.07509	0.03465
0.7107	0.1103	0.1118	0.0672
0.07646	0.7328	0.144	0.04667
0.06372	0.7949	0.07423	0.06717

MOTIF GAGGCCTGAA

0.08575	0.1071	0.7447	0.06246
0.7139	0.0927	0.1057	0.08769
0.1087	0.05861	0.7671	0.06559
0.0783	0.1275	0.747	0.04717
0.07393	0.7572	0.1049	0.06391
0.07345	0.7903	0.05504	0.08121
0.0934	0.06545	0.1238	0.7174
0.1026	0.09423	0.7416	0.06154
0.7454	0.09465	0.1076	0.05241
0.7306	0.0623	0.1461	0.06101

MOTIF ATCTGCCG

0.7057	0.1138	0.09978	0.0807
0.07551	0.1259	0.137	0.6617
0.06471	0.803	0.0803	0.05197
0.07341	0.05883	0.1018	0.7659
0.03166	0.06071	0.8417	0.06593
0.08317	0.773	0.08681	0.05701
0.09848	0.7338	0.06903	0.09867

0.06355	0.05324	0.8004	0.08279

MOTIF CCTGCGCC

0.06813	0.7609	0.09403	0.07698
0.05604	0.7847	0.1031	0.05621
0.06899	0.1089	0.1107	0.7114
0.04294	0.1384	0.7384	0.08024
0.09639	0.7343	0.07569	0.09359
0.08578	0.07736	0.7425	0.09436
0.05373	0.7807	0.09922	0.06638
0.08552	0.7348	0.1089	0.07073

MOTIF CAGGGCGA

0.05779	0.7827	0.1059	0.05362
0.7537	0.1075	0.06029	0.07849
0.07687	0.07353	0.7859	0.06372
0.06828	0.09543	0.7956	0.04066
0.05068	0.1121	0.7552	0.08198
0.07144	0.8051	0.07604	0.04746
0.07775	0.0811	0.7715	0.06965
0.7153	0.13	0.09292	0.06176

MOTIF TCACCGGG

0.09026	0.1277	0.1879	0.5941
0.06647	0.7916	0.08933	0.05262
0.7937	0.05039	0.0903	0.06558
0.05093	0.8194	0.07911	0.05056
0.07592	0.8261	0.04517	0.05283
0.09843	0.0609	0.7658	0.07488
0.06515	0.08119	0.7841	0.06957
0.1613	0.1235	0.6467	0.06851

MOTIF CCAGACGG

0.05902	0.7888	0.09329	0.05885
0.04164	0.8466	0.06759	0.04413
0.6752	0.08962	0.1326	0.1026
0.05524	0.07507	0.8199	0.04984
0.7051	0.09117	0.1283	0.0755
0.0863	0.7771	0.09367	0.04291
0.105	0.09461	0.7183	0.08207
0.1092	0.1102	0.7169	0.06371

MOTIF TCGGTAGC

0.06943	0.09577	0.09832	0.7365
0.05984	0.7922	0.06456	0.08344
0.08934	0.08752	0.7607	0.06244
0.05586	0.1008	0.8011	0.04226
0.06815	0.1643	0.07279	0.6947
0.6448	0.1061	0.1713	0.07781
0.05364	0.05786	0.848	0.04053

0.05186	0.8282	0.05806	0.06193

MOTIF CAGGCCGA

0.05366	0.7894	0.1185	0.03846
0.6964	0.0915	0.1416	0.07056
0.07034	0.09173	0.7814	0.05655
0.05309	0.1093	0.7633	0.07426
0.04885	0.7583	0.1098	0.08301
0.09231	0.7618	0.08499	0.06091
0.09268	0.1058	0.7532	0.04829
0.6687	0.1201	0.1388	0.07242

MOTIF GCGCGCAT

0.03588	0.06751	0.835	0.06166
0.06713	0.8345	0.05202	0.0463
0.06819	0.1244	0.7318	0.0756
0.09054	0.7432	0.08323	0.08307
0.05758	0.09404	0.8072	0.04121
0.0629	0.8181	0.08517	0.03385
0.6272	0.159	0.1135	0.1003
0.06589	0.1112	0.1818	0.6412

MOTIF TCGACCTG

0.08445	0.1076	0.148	0.66
0.03886	0.7887	0.08992	0.08253
0.05922	0.1329	0.7475	0.0604
0.6249	0.1523	0.1542	0.06859
0.05079	0.793	0.1044	0.05179
0.04329	0.8229	0.06109	0.07272
0.07408	0.06436	0.1143	0.7472
0.05064	0.0857	0.8265	0.03718

MOTIF CCTGACGA

0.07766	0.704	0.1126	0.1057
0.04827	0.8065	0.09502	0.05022
0.1348	0.06604	0.09886	0.7003
0.03633	0.0815	0.8495	0.03272
0.6884	0.1178	0.1225	0.07135
0.1252	0.6903	0.09581	0.08875
0.06478	0.1032	0.777	0.05499
0.694	0.09293	0.1258	0.08735

MOTIF AAGCGG

0.7877	0.08264	0.07363	0.056
0.788	0.07019	0.0762	0.06557
0.0541	0.06384	0.8504	0.03167
0.07905	0.789	0.06404	0.0679
0.08134	0.05471	0.7715	0.09243
0.05627	0.04724	0.8711	0.0254

MOTIF CTGGGT

0.03595	0.8452	0.07616	0.0427
0.08266	0.06186	0.06765	0.7878
0.0489	0.07874	0.8078	0.06459
0.0558	0.08905	0.7978	0.0573
0.07375	0.1189	0.7437	0.06358
0.05065	0.1264	0.08793	0.735

MOTIF GTACGC

0.04308	0.06544	0.8454	0.04613
0.05956	0.1308	0.1089	0.7008
0.6206	0.1426	0.1521	0.08465
0.0445	0.8567	0.05682	0.04196
0.0389	0.06747	0.8502	0.04341
0.03443	0.8201	0.07345	0.07203

MOTIF CTGACG

0.03444	0.8501	0.06725	0.04824
0.06861	0.06937	0.06331	0.7987
0.0255	0.05665	0.8936	0.02423
0.6789	0.1086	0.1515	0.06094
0.1016	0.7499	0.07335	0.07512
0.04763	0.06859	0.8221	0.06165

MOTIF GGGCGT

0.03388	0.06609	0.8184	0.0816
0.05277	0.07218	0.8156	0.05942
0.04934	0.08573	0.813	0.05192
0.05525	0.8217	0.07082	0.05226
0.0622	0.07404	0.8054	0.05837
0.09806	0.07869	0.09573	0.7275

MOTIF CGGCAG

0.07378	0.7876	0.06091	0.0777
0.1231	0.0494	0.7993	0.02825
0.0482	0.1186	0.7678	0.06538
0.04352	0.8768	0.04061	0.03907
0.8284	0.02564	0.08573	0.0602
0.03766	0.08237	0.8402	0.03982

MOTIF GCAGGG

0.0458	0.05184	0.8449	0.05748
0.03616	0.8682	0.06744	0.02817
0.796	0.07517	0.05575	0.07313
0.04969	0.07589	0.8132	0.06121
0.06426	0.08134	0.7897	0.0647
0.04773	0.07319	0.7975	0.08162

MOTIF CGGTAG

0.07176	0.7929	0.05962	0.0757

0.07316	0.06452	0.8052	0.05709
0.03109	0.09069	0.8441	0.03409
0.0837	0.155	0.0776	0.6837
0.6757	0.09411	0.1391	0.091
0.05829	0.05362	0.8505	0.03756

MOTIF CACGGA

0.04962	0.8506	0.05479	0.04501
0.7395	0.05455	0.1109	0.09509
0.05333	0.7767	0.1132	0.05679
0.0586	0.07477	0.7955	0.07111
0.05823	0.09255	0.7937	0.05548
0.7622	0.09773	0.06311	0.07696

MOTIF GCCGTT

0.04084	0.05729	0.851	0.05085
0.02092	0.8771	0.06567	0.03635
0.0884	0.7664	0.06077	0.08443
0.05737	0.08451	0.7878	0.0703
0.06042	0.1044	0.08955	0.7457
0.06003	0.08122	0.0955	0.7633

MOTIF CCCGGT

0.01685	0.7348	0.08221	0.1662
0.07021	0.8121	0.05186	0.06584
0.07184	0.8076	0.04915	0.07139
0.04443	0.05807	0.8336	0.06393
0.03733	0.09739	0.8195	0.04577
0.04212	0.1166	0.01662	0.8247

MOTIF ACAGGT

0.6813	0.09398	0.1603	0.06448
0.04744	0.8444	0.06957	0.03857
0.8544	0.02741	0.04256	0.07568
0.05823	0.03391	0.8898	0.01806
0.05705	0.09134	0.8005	0.05114
0.09537	0.09454	0.08654	0.7236

MOTIF ATAGCG

0.7247	0.1467	0.06937	0.05924
0.1285	0.1218	0.1593	0.5904
0.7186	0.07505	0.1504	0.05595
0.02348	0.06779	0.8856	0.02311
0.04344	0.8226	0.08404	0.04988
0.09936	0.08804	0.7169	0.09567

MOTIF AAACGC

0.7603	0.1117	0.08159	0.04641
0.7295	0.09668	0.1199	0.05393
0.7608	0.1059	0.08534	0.04795

0.0921	0.7016	0.1262	0.08001
0.05657	0.06878	0.8114	0.06325
0.03782	0.829	0.106	0.02718

MOTIF GCCGAA

0.05347	0.05227	0.8584	0.03586
0.05264	0.8494	0.06629	0.03162
0.07833	0.7954	0.05224	0.07403
0.06197	0.06617	0.8116	0.06031
0.7881	0.07133	0.09229	0.04832
0.7993	0.0713	0.07875	0.05068

MOTIF GCGTCT

0.03604	0.06312	0.8383	0.06252
0.04049	0.8164	0.07175	0.07136
0.05578	0.1257	0.7166	0.102
0.07999	0.09926	0.1009	0.7199
0.03885	0.8351	0.06602	0.06001
0.07338	0.09392	0.09289	0.7398

MOTIF CCAGAC

0.0568	0.8175	0.07404	0.05166
0.03073	0.8775	0.04881	0.043
0.6859	0.06891	0.1721	0.07314
0.05585	0.05856	0.8506	0.03497
0.6846	0.1459	0.1017	0.06775
0.06559	0.8135	0.06529	0.05561

MOTIF TTGCGC

0.07765	0.1348	0.1224	0.6651
0.0507	0.08035	0.1327	0.7363
0.03618	0.06702	0.8526	0.04422
0.06642	0.7923	0.08456	0.05673
0.0527	0.05547	0.785	0.1069
0.03067	0.8665	0.06258	0.04024

MOTIF CCAGGC

0.05502	0.7951	0.07727	0.07266
0.05684	0.8377	0.05808	0.04734
0.7402	0.07625	0.1091	0.07439
0.04642	0.05397	0.8329	0.06671
0.06483	0.1234	0.7555	0.05625
0.04453	0.8473	0.05523	0.05297

MOTIF CTGATC

0.03334	0.88	0.04295	0.04369
0.0868	0.05763	0.05681	0.7988
0.03848	0.06373	0.872	0.0258
0.6787	0.154	0.09105	0.07626
0.1028	0.08816	0.09869	0.7103

0.06303	0.7848	0.1039	0.0483

MOTIF CCAGCAGAAT

0.06829	0.7596	0.08718	0.08493
0.08324	0.7511	0.05761	0.108
0.8508	0.03157	0.05264	0.06496
0.08028	0.09762	0.7481	0.07401
0.06681	0.8332	0.05415	0.04584
0.8619	0.04085	0.0447	0.05252
0.1039	0.06733	0.7722	0.0566
0.6949	0.08089	0.1132	0.111
0.7442	0.1046	0.1038	0.04736
0.0758	0.1126	0.1095	0.7021

MOTIF TGCAAGCGTG

0.07515	0.1125	0.09972	0.7126
0.06364	0.08832	0.7635	0.08455
0.1277	0.7207	0.07954	0.07211
0.7582	0.1062	0.08165	0.05392
0.705	0.1004	0.1034	0.09122
0.1077	0.098	0.721	0.07336
0.111	0.7172	0.08648	0.08538
0.0994	0.1331	0.6386	0.129
0.08493	0.09026	0.08466	0.7401
0.06027	0.0821	0.8016	0.05599

MOTIF CAGGCGCT

0.08976	0.786	0.08467	0.03955
0.6697	0.1261	0.1089	0.09536
0.07721	0.05665	0.8012	0.06494
0.04998	0.1036	0.7769	0.06958
0.1089	0.706	0.1089	0.07608
0.07251	0.0595	0.8013	0.06669
0.04951	0.8356	0.06511	0.04974
0.1135	0.1125	0.1165	0.6576

MOTIF ACGACTGT

0.6862	0.1199	0.1123	0.08161
0.0974	0.7301	0.07293	0.09954
0.1092	0.09835	0.695	0.09738
0.8022	0.09512	0.0672	0.03546
0.04363	0.8561	0.05924	0.04103
0.06449	0.0521	0.07203	0.8114
0.04568	0.06814	0.8408	0.04542
0.08017	0.09193	0.08054	0.7474

MOTIF CTGACCGG

0.0359	0.8003	0.1085	0.05535
0.1076	0.0514	0.09822	0.7428
0.02469	0.1162	0.8157	0.04344

0.6109	0.09597	0.1029	0.1901
0.115	0.7546	0.09689	0.03353
0.08947	0.7633	0.07504	0.07216
0.06163	0.04869	0.7996	0.09012
0.04463	0.0886	0.7785	0.08823

MOTIF GCTACAGT

0.06788	0.08649	0.7942	0.05141
0.07337	0.7939	0.07218	0.06055
0.1012	0.1069	0.1004	0.6916
0.6998	0.09986	0.09884	0.1015
0.05242	0.8228	0.06567	0.05913
0.7371	0.07709	0.05094	0.1349
0.06571	0.07974	0.7739	0.08063
0.08238	0.1005	0.06991	0.7472

MOTIF CTGGCTGT

0.08079	0.7181	0.1169	0.08419
0.07156	0.07811	0.08096	0.7694
0.05044	0.06446	0.7992	0.08589
0.1229	0.09665	0.708	0.07248
0.07035	0.7688	0.08503	0.07587
0.0788	0.08453	0.06238	0.7743
0.06601	0.07587	0.8052	0.05292
0.06548	0.1342	0.08267	0.7176

MOTIF CTGAACGG

0.03429	0.836	0.0878	0.0419
0.08577	0.04341	0.07426	0.7966
0.03498	0.09313	0.8337	0.03814
0.6844	0.1079	0.1185	0.0892
0.668	0.1877	0.1086	0.03572
0.07232	0.7559	0.1008	0.07098
0.0593	0.04203	0.8008	0.09792
0.05462	0.05756	0.8344	0.05339

MOTIF TTAACGCA

0.1011	0.1377	0.1139	0.6473
0.1111	0.1943	0.1555	0.5391
0.6827	0.1116	0.157	0.04865
0.6456	0.1052	0.1645	0.08474
0.1173	0.7066	0.08167	0.09449
0.1069	0.07015	0.762	0.06086
0.03637	0.8569	0.06702	0.0397
0.667	0.1463	0.09774	0.08899

MOTIF CAAGCGTG

0.0839	0.7759	0.09149	0.04868
0.7619	0.09342	0.09849	0.04618
0.7947	0.03142	0.1043	0.0696

0.04301	0.1012	0.8012	0.0545
0.1137	0.6529	0.1055	0.1279
0.0857	0.1079	0.7783	0.02809
0.09302	0.1185	0.09468	0.6938
0.07382	0.0902	0.7991	0.03683

MOTIF CTGGCCGG

0.0416	0.7377	0.1331	0.08755
0.1054	0.03083	0.0668	0.797
0.04504	0.1356	0.753	0.06638
0.09315	0.09415	0.6279	0.1848
0.09822	0.7637	0.06607	0.07204
0.09099	0.7582	0.0636	0.08716
0.02286	0.06818	0.8369	0.07205
0.06412	0.1008	0.7588	0.07625

MOTIF GTTACGCG

0.06638	0.1036	0.7579	0.07209
0.05834	0.1488	0.1392	0.6536
0.08704	0.1925	0.2489	0.4716
0.641	0.1159	0.1734	0.06967
0.06924	0.7694	0.09934	0.06205
0.05944	0.09451	0.7671	0.07891
0.07111	0.7578	0.08994	0.08119
0.07221	0.06712	0.7631	0.09753

MOTIF ACAGCGGC

0.6547	0.1082	0.1762	0.06098
0.05764	0.8131	0.0882	0.04109
0.7055	0.06395	0.153	0.07759
0.08594	0.1042	0.7567	0.05317
0.07601	0.6958	0.09278	0.1354
0.1097	0.09772	0.7331	0.05944
0.08508	0.1029	0.7135	0.09859
0.06425	0.8515	0.04763	0.03666

MOTIF CTGGCGCA

0.09301	0.7367	0.09249	0.07781
0.1388	0.1512	0.09053	0.6194
0.07279	0.08023	0.7956	0.0514
0.05839	0.1021	0.7501	0.08943
0.1049	0.7038	0.06645	0.1248
0.0794	0.05359	0.8118	0.05523
0.04112	0.8279	0.09498	0.03598
0.6634	0.1358	0.08956	0.1112

MOTIF CCAGCAGA

0.05425	0.7434	0.1113	0.09109
0.08873	0.785	0.06633	0.05998
0.8265	0.03493	0.08354	0.05505

0.08932	0.1029	0.7438	0.06403
0.06466	0.8238	0.05534	0.05616
0.8065	0.06031	0.08663	0.04652
0.08125	0.09454	0.7713	0.05287
0.7545	0.09952	0.08268	0.06332

MOTIF GCAGCGCA

0.07712	0.09635	0.7748	0.05169
0.06316	0.8191	0.07146	0.04625
0.7434	0.07584	0.09597	0.08477
0.05388	0.1127	0.7876	0.0458
0.08538	0.7609	0.0726	0.08107
0.09223	0.07969	0.757	0.07109
0.05708	0.746	0.1497	0.04722
0.6615	0.1412	0.1057	0.09153

MOTIF TGCGAC

0.06641	0.06973	0.07961	0.7842
0.03754	0.08768	0.8476	0.02719
0.03633	0.8387	0.06897	0.05598
0.1057	0.1041	0.6985	0.09169
0.6711	0.1466	0.1035	0.07878
0.02715	0.8692	0.04938	0.05427

MOTIF ACTGGC

0.756	0.07713	0.104	0.06284
0.05005	0.8216	0.08455	0.04382
0.07448	0.06167	0.06818	0.7957
0.03482	0.03619	0.8808	0.04816
0.07545	0.06338	0.7251	0.1361
0.06005	0.8367	0.04792	0.05535

MOTIF ACGCTT

0.7461	0.07682	0.1143	0.06279
0.0267	0.7839	0.07676	0.1126
0.1096	0.07248	0.7504	0.06749
0.03723	0.8561	0.06951	0.03712
0.05622	0.07339	0.03347	0.8369
0.04439	0.08477	0.1027	0.7682

MOTIF GCCTAC

0.0467	0.05187	0.8443	0.05708
0.05712	0.8046	0.0986	0.03973
0.06309	0.8409	0.04548	0.05053
0.1056	0.08788	0.1157	0.6908
0.6906	0.08985	0.116	0.1036
0.04348	0.8457	0.0707	0.04015

MOTIF CAGTCG

0.04185	0.8619	0.05349	0.04281

0.771	0.07666	0.0799	0.07242
0.02002	0.06077	0.8939	0.02528
0.05451	0.09656	0.1125	0.7364
0.08764	0.7386	0.1018	0.07195
0.08139	0.08404	0.7625	0.07202

MOTIF AGCGCC

0.7661	0.09616	0.03126	0.1065
0.03648	0.06497	0.8608	0.03776
0.04448	0.8512	0.04862	0.0557
0.05704	0.08215	0.7722	0.08863
0.04773	0.7979	0.1032	0.05119
0.07147	0.8572	0.05405	0.01733

MOTIF ACGGAA

0.7497	0.09967	0.09896	0.05167
0.08025	0.7838	0.08313	0.05281
0.1125	0.06284	0.7119	0.1128
0.04212	0.09182	0.8396	0.02649
0.7685	0.1106	0.06624	0.05466
0.717	0.08897	0.1391	0.05495

MOTIF TGCGCC

0.09686	0.08457	0.03012	0.7884
0.02651	0.08424	0.8399	0.04932
0.0415	0.864	0.03923	0.05526
0.131	0.06104	0.7233	0.08472
0.05454	0.82	0.06895	0.05653
0.01507	0.8534	0.06795	0.06359

MOTIF GTCAGT

0.06207	0.05707	0.8111	0.06972
0.1515	0.1025	0.07164	0.6744
0.0295	0.8898	0.05162	0.02911
0.827	0.05748	0.05618	0.05934
0.04181	0.1041	0.8045	0.04957
0.07125	0.1033	0.1071	0.7184

MOTIF ACTCTG

0.7376	0.09426	0.08533	0.08277
0.04197	0.7986	0.0876	0.07186
0.1123	0.06683	0.06846	0.7525
0.03749	0.8126	0.09542	0.05454
0.05941	0.1093	0.05385	0.7775
0.03869	0.06863	0.8462	0.04652

MOTIF GCACCT

0.06597	0.06091	0.82	0.05308
0.05112	0.8693	0.03931	0.04025
0.7107	0.08266	0.09037	0.1163

0.04335	0.8018	0.1025	0.05228
0.05075	0.8564	0.04057	0.0523
0.08029	0.08101	0.1477	0.691

MOTIF GTAGCC

0.06594	0.05268	0.8331	0.04827
0.08202	0.1453	0.08849	0.6842
0.7584	0.08093	0.09227	0.06841
0.05333	0.05453	0.8578	0.03435
0.04499	0.821	0.07715	0.05685
0.09223	0.7716	0.06807	0.06813

MOTIF AGCGGC

0.7136	0.1046	0.1114	0.07042
0.05869	0.05311	0.8628	0.02544
0.04357	0.8113	0.07733	0.06779
0.0978	0.07081	0.744	0.08738
0.04478	0.09183	0.8168	0.04658
0.0674	0.8383	0.04818	0.04615

MOTIF GTGTAC

0.05804	0.0772	0.8005	0.06431
0.05093	0.05237	0.06948	0.8272
0.02048	0.06628	0.885	0.02829
0.07758	0.09114	0.062	0.7693
0.5997	0.1078	0.1815	0.111
0.04628	0.8144	0.08779	0.05155

MOTIF GCGGCATGGA

0.1064	0.1003	0.6959	0.09741
0.1099	0.6647	0.1135	0.1119
0.1125	0.1155	0.6911	0.08092
0.07011	0.1019	0.7503	0.07765
0.0585	0.8083	0.09219	0.04105
0.6915	0.09774	0.1246	0.0862
0.07968	0.09045	0.1022	0.7277
0.06474	0.09201	0.7615	0.08172
0.1047	0.06973	0.7687	0.0569
0.7438	0.09835	0.06927	0.08853

MOTIF CCGTTCTGAG

0.1024	0.7287	0.107	0.06192
0.131	0.7077	0.06544	0.09587
0.09715	0.08038	0.7507	0.07177
0.09846	0.09321	0.09162	0.7167
0.05902	0.1093	0.07514	0.7566
0.07324	0.7435	0.09848	0.08481
0.08246	0.1246	0.09755	0.6954
0.09289	0.08783	0.7532	0.06606
0.6318	0.07504	0.1267	0.1665

0.06502	0.06362	0.7563	0.1151

MOTIF CCCTACTATG

0.1216	0.6825	0.132	0.06394
0.06721	0.6921	0.1263	0.1144
0.1095	0.7656	0.06299	0.06189
0.06106	0.1099	0.05799	0.7711
0.7283	0.08836	0.08975	0.09359
0.06959	0.7626	0.076	0.09183
0.07311	0.1246	0.0676	0.7347
0.6569	0.1579	0.06131	0.1239
0.07704	0.0918	0.07475	0.7564
0.08069	0.07618	0.7416	0.1015

MOTIF ACACTGAG

0.7406	0.08735	0.1	0.07205
0.0896	0.7826	0.06513	0.06269
0.8145	0.06594	0.05476	0.06478
0.08942	0.7435	0.08427	0.08284
0.09734	0.05327	0.1013	0.7481
0.06143	0.05688	0.8306	0.05112
0.7656	0.06257	0.07892	0.09294
0.1088	0.08955	0.7313	0.07044

MOTIF GCGAGCGA

0.05758	0.04378	0.8668	0.03184
0.1142	0.7092	0.1179	0.05877
0.0842	0.08347	0.7662	0.06616
0.6666	0.08405	0.151	0.09839
0.06861	0.05109	0.8213	0.05895
0.1072	0.7062	0.1262	0.06041
0.06529	0.07949	0.8055	0.04968
0.7356	0.1062	0.0852	0.07294

MOTIF TGTACGGC

0.07953	0.08274	0.08147	0.7563
0.04681	0.09553	0.8028	0.05482
0.09394	0.1098	0.1312	0.6651
0.6797	0.09441	0.1262	0.09975
0.1005	0.7422	0.08596	0.0713
0.06912	0.06476	0.759	0.1071
0.06962	0.06198	0.7856	0.08279
0.06488	0.7864	0.05578	0.09294

MOTIF CCTACGCC

0.05863	0.8139	0.05877	0.06868
0.03816	0.8434	0.06413	0.05429
0.1147	0.1019	0.1024	0.681
0.5652	0.1764	0.1232	0.1352
0.08486	0.8118	0.05786	0.04547

0.07419	0.08212	0.7408	0.1029
0.08146	0.7834	0.07887	0.05627
0.05857	0.8326	0.05574	0.05307

MOTIF CCTCCGTG

0.0585	0.7171	0.08159	0.1428
0.06548	0.7765	0.06879	0.08923
0.08742	0.0445	0.1412	0.7269
0.05123	0.8051	0.06728	0.07641
0.1197	0.7219	0.07176	0.08669
0.1139	0.1024	0.6974	0.08627
0.06561	0.1365	0.03221	0.7656
0.05821	0.1255	0.7163	0.1

MOTIF AGTCCTCC

0.6887	0.136	0.07583	0.09949
0.07294	0.1674	0.6912	0.06842
0.06772	0.09418	0.07552	0.7626
0.0557	0.804	0.05388	0.08645
0.108	0.7105	0.1203	0.06122
0.07207	0.08086	0.06781	0.7793
0.06446	0.7874	0.07917	0.06898
0.1103	0.7419	0.08093	0.06688

MOTIF GCCTTCCA

0.09078	0.1114	0.6954	0.1024
0.07646	0.7424	0.0879	0.09326
0.07419	0.796	0.07039	0.05945
0.09402	0.1071	0.09562	0.7033
0.08913	0.09157	0.09408	0.7252
0.07532	0.7578	0.09276	0.07414
0.06166	0.7816	0.08137	0.07542
0.732	0.08089	0.08683	0.1003

MOTIF CGCGGAAC

0.08609	0.7664	0.07227	0.07524
0.1242	0.08934	0.7129	0.0735
0.07632	0.7157	0.07793	0.1301
0.1038	0.0932	0.716	0.08698
0.0557	0.06127	0.8395	0.04357
0.7371	0.09373	0.09006	0.07907
0.6311	0.119	0.1038	0.1462
0.06394	0.7517	0.1351	0.04927

MOTIF CCGTTCTG

0.0902	0.7481	0.08603	0.07566
0.07667	0.7278	0.1012	0.09441
0.08315	0.1145	0.7239	0.07848
0.05206	0.06872	0.07481	0.8044
0.05919	0.1037	0.1226	0.7145

0.04975	0.7901	0.0874	0.07279
0.07688	0.106	0.05574	0.7614
0.05869	0.08838	0.7752	0.07775

MOTIF CCGTACCA

0.05728	0.7794	0.06322	0.1001
0.106	0.7484	0.08271	0.06291
0.1063	0.1394	0.6754	0.07887
0.07762	0.1164	0.09763	0.7083
0.5864	0.1536	0.1235	0.1365
0.07622	0.7613	0.1054	0.0571
0.08379	0.7965	0.05444	0.06524
0.7413	0.08568	0.09606	0.07696

MOTIF TCCGTG

0.06435	0.03681	0.06641	0.8324
0.03573	0.8488	0.06425	0.05124
0.112	0.7361	0.06792	0.08395
0.1166	0.07979	0.724	0.07959
0.04369	0.1165	0.03477	0.8051
0.04401	0.04746	0.8577	0.05078

MOTIF CGTTCC

0.06086	0.8205	0.05668	0.06202
0.06839	0.1227	0.7528	0.05614
0.04333	0.09648	0.05694	0.8033
0.04984	0.1111	0.07049	0.7686
0.04166	0.8222	0.06695	0.06917
0.05834	0.8083	0.04783	0.0855

MOTIF TACGGC

0.1015	0.0786	0.1461	0.6738
0.7078	0.08723	0.1332	0.07177
0.05827	0.8158	0.06748	0.0585
0.06024	0.05495	0.8068	0.07804
0.06297	0.06224	0.8316	0.0432
0.05101	0.8422	0.04665	0.06011

MOTIF ATGCCG

0.751	0.09058	0.07884	0.07961
0.08047	0.07908	0.04882	0.7916
0.04228	0.05213	0.8716	0.03402
0.07044	0.736	0.1173	0.07627
0.06975	0.793	0.06904	0.06823
0.0866	0.07027	0.77	0.07313

MOTIF TCAGTG

0.08745	0.05615	0.09083	0.7656
0.02925	0.8726	0.05495	0.04315
0.7569	0.09403	0.06627	0.08283

0.06918	0.07935	0.7762	0.0753
0.05985	0.06314	0.04913	0.8279
0.04671	0.05142	0.8423	0.05958

MOTIF AGTGCG

0.7484	0.09222	0.08713	0.07223
0.04708	0.07389	0.8134	0.06565
0.05894	0.06437	0.09145	0.7852
0.04252	0.05323	0.8501	0.05413
0.07239	0.7551	0.09381	0.0787
0.08744	0.09319	0.7349	0.08452

MOTIF GGTAGG

0.07537	0.06286	0.7962	0.06558
0.04147	0.06909	0.8448	0.0446
0.1211	0.1018	0.1197	0.6575
0.7459	0.07703	0.09758	0.07947
0.04511	0.0568	0.8317	0.06637
0.06256	0.04633	0.8426	0.0485

MOTIF CGTAGT

0.08526	0.7788	0.05847	0.07746
0.07655	0.09539	0.768	0.06003
0.06018	0.07887	0.05659	0.8044
0.703	0.1414	0.09206	0.06347
0.04788	0.04699	0.8647	0.04048
0.07766	0.08041	0.07461	0.7673

MOTIF CTAGCG

0.05007	0.8342	0.05997	0.05579
0.06471	0.114	0.08228	0.739
0.699	0.1028	0.1179	0.0803
0.03935	0.06919	0.8508	0.0407
0.0776	0.784	0.05729	0.08109
0.09044	0.07256	0.7562	0.0808

MOTIF AATCGC

0.7265	0.1203	0.08912	0.06414
0.7483	0.08495	0.09755	0.06923
0.06575	0.1039	0.1066	0.7238
0.0608	0.7288	0.1519	0.05846
0.07407	0.08943	0.7624	0.07407
0.06165	0.8356	0.05705	0.04572

MOTIF AACGGC

0.7306	0.071	0.1337	0.06464
0.8127	0.04054	0.0901	0.05665
0.07312	0.7584	0.08273	0.08577
0.0873	0.06345	0.7953	0.05391
0.05574	0.05969	0.8406	0.04398

0.0578	0.8078	0.07386	0.0605

MOTIF TACTGG

0.05239	0.07254	0.08535	0.7897
0.6849	0.1001	0.1278	0.08727
0.06837	0.8044	0.08258	0.04461
0.06854	0.05131	0.04501	0.8351
0.0744	0.06932	0.8014	0.05493
0.05443	0.0758	0.7908	0.079

MOTIF TACGAG

0.08912	0.05768	0.09756	0.7556
0.7682	0.06448	0.09795	0.06934
0.06646	0.7509	0.1157	0.06698
0.07512	0.08928	0.7454	0.0902
0.8223	0.07038	0.05544	0.05186
0.03815	0.07437	0.8326	0.05488

MOTIF CTTACG

0.06226	0.822	0.07286	0.04289
0.07901	0.1081	0.02944	0.7834
0.05235	0.1067	0.08016	0.7607
0.6524	0.1193	0.1203	0.1079
0.03708	0.804	0.09827	0.06068
0.09029	0.07945	0.7375	0.09277

MOTIF CGTACC

0.07342	0.8386	0.02442	0.06352
0.0893	0.1216	0.7301	0.05894
0.07118	0.1036	0.07736	0.7478
0.6244	0.1544	0.09692	0.1242
0.05719	0.8076	0.0855	0.04968
0.07493	0.8433	0.02523	0.05652

MOTIF CCGCGCATGA

0.07141	0.7348	0.1011	0.09265
0.06352	0.8135	0.07427	0.0487
0.04225	0.07177	0.781	0.105
0.1086	0.7512	0.07371	0.06647
0.0515	0.08876	0.7951	0.0646
0.04189	0.8105	0.09937	0.04826
0.6737	0.1377	0.07537	0.1133
0.09727	0.1598	0.1135	0.6294
0.03067	0.06817	0.8449	0.05623
0.7061	0.1418	0.07565	0.07651

MOTIF GGGTGTTCGT

0.07714	0.1078	0.7263	0.08878
0.03957	0.07534	0.8245	0.06061
0.05822	0.06076	0.8249	0.05613

0.1273	0.06858	0.05472	0.7494
0.03119	0.06067	0.8247	0.08342
0.04282	0.08932	0.0862	0.7817
0.03459	0.07196	0.09576	0.7977
0.03371	0.7999	0.0851	0.08132
0.06236	0.08857	0.7631	0.08595
0.03705	0.114	0.1288	0.7202

MOTIF AGCGGCGA

0.6595	0.1398	0.1334	0.06728
0.0841	0.0779	0.7717	0.06632
0.07246	0.7951	0.08894	0.04346
0.07775	0.08834	0.7805	0.05344
0.07705	0.1152	0.7575	0.05027
0.09871	0.7895	0.07228	0.03953
0.06345	0.07683	0.8148	0.04491
0.6271	0.1294	0.156	0.08741

MOTIF GCAAGCGC

0.07291	0.06598	0.7689	0.09223
0.05315	0.8309	0.07829	0.03767
0.7089	0.115	0.1027	0.07333
0.656	0.1232	0.1176	0.1032
0.06109	0.09237	0.8126	0.03394
0.0672	0.7762	0.1213	0.0353
0.1142	0.1234	0.6992	0.06312
0.06617	0.7593	0.1011	0.07343

MOTIF GGGACCCT

0.06465	0.1073	0.7533	0.07473
0.1172	0.05496	0.7419	0.08592
0.07771	0.07242	0.7711	0.07873
0.7087	0.09992	0.08858	0.1028
0.05774	0.8082	0.09704	0.03702
0.05493	0.8022	0.07829	0.06455
0.05029	0.7649	0.1122	0.07265
0.05718	0.1016	0.1097	0.7316

MOTIF GCCCCGAA

0.07351	0.07013	0.8126	0.04373
0.07263	0.7806	0.09202	0.05475
0.05305	0.7703	0.1157	0.06094
0.06967	0.7688	0.112	0.04953
0.05289	0.8057	0.07651	0.06494
0.04232	0.07482	0.8206	0.06224
0.6045	0.1338	0.1606	0.101
0.6352	0.153	0.1312	0.08058

MOTIF GGTCCGAG

0.1093	0.1216	0.6942	0.07481

0.07084	0.07748	0.8041	0.04758
0.08835	0.1428	0.1786	0.5903
0.04114	0.8244	0.07736	0.05705
0.05414	0.7586	0.1079	0.07935
0.05489	0.0824	0.7951	0.06765
0.6738	0.1265	0.1263	0.07339
0.04236	0.0831	0.8195	0.05509

MOTIF GCCGGTCT

0.05065	0.08424	0.8075	0.05763
0.1009	0.7298	0.09662	0.07268
0.06742	0.7638	0.09695	0.07184
0.04702	0.0825	0.7967	0.07375
0.07765	0.09	0.7561	0.07625
0.09652	0.1531	0.1409	0.6094
0.02879	0.7959	0.09411	0.08117
0.09669	0.1607	0.1241	0.6185

MOTIF TGACGGTA

0.06853	0.1731	0.1379	0.6205
0.0265	0.0913	0.836	0.04619
0.7826	0.04697	0.1123	0.05818
0.05086	0.8049	0.09834	0.04586
0.0482	0.1272	0.7832	0.04136
0.04443	0.0688	0.8548	0.03197
0.1184	0.1262	0.1025	0.6529
0.5264	0.1889	0.2225	0.06216

MOTIF GGCGGTGC

0.05485	0.08334	0.8038	0.05799
0.08411	0.06884	0.7549	0.09215
0.0551	0.7971	0.1002	0.04759
0.0556	0.09832	0.7763	0.06977
0.07815	0.08841	0.7624	0.07098
0.124	0.1624	0.1483	0.5653
0.03408	0.1047	0.8066	0.0546
0.06725	0.7204	0.12	0.09234

MOTIF GGAAACCC

0.08314	0.08534	0.7585	0.07299
0.09437	0.1156	0.7295	0.06049
0.7083	0.1178	0.07985	0.09397
0.7635	0.1109	0.08196	0.04356
0.7083	0.1353	0.111	0.0454
0.06336	0.7999	0.07367	0.06309
0.04282	0.8094	0.09418	0.0536
0.08555	0.7556	0.08516	0.07369

MOTIF GCTACGGC

0.07425	0.1438	0.7342	0.04766

0.02772	0.8496	0.07368	0.04899
0.09352	0.1104	0.1694	0.6266
0.6411	0.1476	0.1363	0.07503
0.07285	0.7704	0.1177	0.03903
0.02724	0.07688	0.8239	0.07197
0.07388	0.08121	0.778	0.0669
0.04322	0.8187	0.06778	0.07029

MOTIF GCCCCG

0.05213	0.05118	0.8473	0.04942
0.05182	0.828	0.06754	0.0526
0.04948	0.8043	0.08587	0.06032
0.05374	0.7614	0.09784	0.08705
0.05468	0.8077	0.07687	0.06077
0.03694	0.05488	0.8486	0.05958

MOTIF CCTACG

0.06121	0.8245	0.07414	0.04016
0.04124	0.8935	0.02767	0.03758
0.06295	0.07518	0.1335	0.7283
0.6027	0.1575	0.1389	0.1008
0.04913	0.8719	0.03528	0.04374
0.05802	0.06093	0.8407	0.04039

MOTIF GCCGTA

0.06021	0.05311	0.8656	0.02111
0.04711	0.8421	0.05694	0.05387
0.05728	0.851	0.0579	0.03383
0.0636	0.0823	0.8032	0.05094
0.06345	0.09841	0.122	0.7161
0.644	0.1505	0.1281	0.07743

MOTIF ACGCCC

0.7327	0.108	0.0648	0.09456
0.07183	0.8066	0.08516	0.03636
0.06377	0.08481	0.8073	0.04411
0.05927	0.7928	0.09452	0.05343
0.05473	0.8046	0.07735	0.06329
0.04556	0.8196	0.07952	0.05532

MOTIF CTTACG

0.0407	0.8266	0.07784	0.05481
0.07854	0.1731	0.05022	0.6982
0.03712	0.1378	0.1576	0.6675
0.5691	0.262	0.09814	0.07072
0.0128	0.8636	0.09342	0.03018
0.04633	0.1023	0.7929	0.05845

MOTIF AATCGC

0.7232	0.1259	0.1038	0.04703

0.7188	0.0963	0.1104	0.07457
0.07739	0.1199	0.1248	0.6779
0.04296	0.8493	0.08862	0.01911
0.05668	0.07556	0.8305	0.03722
0.04291	0.8038	0.1002	0.05308

MOTIF ACGCTC

0.6725	0.1372	0.08404	0.1063
0.04566	0.8229	0.08085	0.05062
0.03681	0.08907	0.8294	0.04472
0.03809	0.8311	0.09337	0.03745
0.0742	0.108	0.1065	0.7113
0.04325	0.8713	0.04619	0.03924

MOTIF CGGGTC

0.0581	0.8093	0.08727	0.04537
0.05366	0.1004	0.795	0.05087
0.08433	0.06691	0.7894	0.0594
0.05562	0.07946	0.8169	0.04806
0.07664	0.1006	0.1502	0.6726
0.04587	0.8267	0.06397	0.06343

MOTIF TATCGT

0.09104	0.208	0.1084	0.5925
0.5175	0.159	0.2168	0.1066
0.05069	0.1311	0.1184	0.6998
0.02744	0.8562	0.09186	0.02448
0.0386	0.09824	0.8227	0.04049
0.04113	0.09712	0.1095	0.7522

MOTIF ATTCGC

0.6603	0.1798	0.07963	0.08022
0.09881	0.1026	0.1166	0.682
0.07519	0.08345	0.1226	0.7188
0.05373	0.8128	0.09746	0.03598
0.03769	0.09709	0.8293	0.03588
0.03398	0.8461	0.07003	0.04993

MOTIF CTTCCG

0.03853	0.8538	0.06278	0.04489
0.05603	0.1479	0.1135	0.6825
0.05971	0.09558	0.07201	0.7727
0.03019	0.8794	0.05626	0.03412
0.03537	0.8441	0.07887	0.0417
0.04762	0.09404	0.7565	0.1018

MOTIF ATACGC

0.7246	0.1175	0.09517	0.06272
0.1002	0.1583	0.09333	0.6482
0.6654	0.09553	0.1448	0.09422

0.04316 0.8509 0.07485 0.0311
0.05999 0.1366 0.7613 0.04213
0.05234 0.8446 0.06733 0.03574

MOTIF CTCAGC
0.04367 0.8265 0.07746 0.05242
0.08191 0.08325 0.08631 0.7485
0.03629 0.8383 0.0849 0.04052
0.6651 0.1542 0.117 0.06368
0.04735 0.05648 0.843 0.05317
0.04251 0.8494 0.07349 0.03461

MOTIF ACGTCT
0.7144 0.1156 0.06839 0.1016
0.03573 0.847 0.07 0.04724
0.03153 0.09456 0.844 0.02987
0.06257 0.1186 0.1157 0.7031
0.02892 0.8844 0.05997 0.02671
0.1145 0.1074 0.1399 0.6382

MOTIF AGCGGA
0.7519 0.08839 0.1182 0.04146
0.04985 0.04121 0.8659 0.04306
0.07934 0.8159 0.0692 0.03557
0.05264 0.1002 0.7732 0.07396
0.05893 0.04765 0.8481 0.04535
0.7681 0.1087 0.0792 0.04407

MOTIF AGGTCG
0.7652 0.06719 0.09438 0.07318
0.03739 0.09011 0.8128 0.05972
0.03404 0.07569 0.8277 0.06259
0.08126 0.1075 0.0883 0.7229
0.0471 0.8338 0.06668 0.05243
0.04422 0.07146 0.8462 0.03816

MOTIF CGGGGT
0.05854 0.7921 0.08501 0.06438
0.04916 0.06808 0.8391 0.04363
0.07926 0.08041 0.7737 0.06663
0.05988 0.08127 0.8164 0.04245
0.0465 0.1171 0.7768 0.05957
0.06946 0.148 0.07348 0.709

MOTIF ATCTCGTGAA
0.8144 0.03543 0.05687 0.09332
0.09423 0.05292 0.09479 0.7581
0.07598 0.6652 0.1187 0.1401
0.07268 0.05496 0.06876 0.8036
0.09888 0.6802 0.06996 0.151

0.1226	0.1115	0.6492	0.1167
0.05919	0.04661	0.04955	0.8446
0.07523	0.08576	0.7787	0.06028
0.7953	0.06945	0.06967	0.0656
0.8052	0.07129	0.06566	0.05786

MOTIF AACTACGAGC

0.7614	0.06889	0.08625	0.08348
0.763	0.04568	0.1327	0.05856
0.07868	0.7417	0.0866	0.09304
0.1075	0.07858	0.09075	0.7232
0.7194	0.06726	0.133	0.08035
0.1227	0.6634	0.1109	0.103
0.1023	0.09923	0.7117	0.08683
0.827	0.06176	0.04072	0.07057
0.09138	0.08405	0.7541	0.07051
0.09209	0.6739	0.1181	0.116

MOTIF CCATTGCTCG

0.06659	0.7831	0.06459	0.0857
0.05984	0.799	0.07	0.07117
0.6875	0.1379	0.0779	0.09664
0.04109	0.04289	0.04928	0.8667
0.05604	0.0878	0.07084	0.7853
0.05576	0.07324	0.7647	0.1063
0.04559	0.8372	0.05534	0.06182
0.05501	0.05229	0.05034	0.8424
0.07116	0.7302	0.0605	0.1381
0.05919	0.1043	0.7341	0.1023

MOTIF CGTGTGACCC

0.07943	0.7946	0.05217	0.07377
0.1071	0.06256	0.6928	0.1376
0.1583	0.0884	0.1053	0.648
0.1025	0.12	0.6907	0.08672
0.05708	0.08008	0.05488	0.808
0.05647	0.08242	0.7926	0.06853
0.7858	0.07816	0.06318	0.07289
0.06799	0.7274	0.1148	0.0898
0.1132	0.6452	0.07442	0.1672
0.09391	0.7082	0.08855	0.1094

MOTIF CACGAAATGG

0.1431	0.6431	0.1359	0.07798
0.784	0.0803	0.05155	0.08413
0.1884	0.5282	0.1188	0.1646
0.1058	0.1136	0.6804	0.1003
0.7855	0.09011	0.06339	0.06102
0.7312	0.1032	0.1058	0.05979
0.8195	0.05639	0.07308	0.05102

0.09228	0.08316	0.06919	0.7554
0.1207	0.07925	0.699	0.1011
0.1004	0.1062	0.6544	0.139

MOTIF CCAGGCAAGG

0.08485	0.7426	0.08901	0.08355
0.1188	0.6913	0.136	0.05384
0.8279	0.05635	0.05007	0.06565
0.1532	0.06895	0.7305	0.04728
0.08632	0.07633	0.7497	0.08762
0.08154	0.776	0.0866	0.05583
0.7949	0.059	0.07563	0.07046
0.8212	0.05799	0.06323	0.05754
0.09902	0.0543	0.752	0.09472
0.1068	0.148	0.6767	0.06852

MOTIF ACTGCGAGCT

0.7412	0.06413	0.08216	0.1125
0.07192	0.7551	0.08501	0.08798
0.06447	0.0618	0.06521	0.8085
0.1008	0.06298	0.7672	0.06895
0.1023	0.7437	0.06422	0.08981
0.0804	0.07527	0.7498	0.09455
0.8277	0.05481	0.03385	0.08369
0.06018	0.06069	0.8304	0.04871
0.05972	0.7587	0.07722	0.1043
0.1028	0.05384	0.07725	0.7661

MOTIF TCGTGTGA

0.06899	0.06524	0.07898	0.7868
0.06849	0.7581	0.06828	0.1051
0.09227	0.05045	0.7275	0.1298
0.09526	0.07171	0.04619	0.7868
0.04886	0.07867	0.752	0.1205
0.0803	0.04814	0.05567	0.8159
0.06595	0.05337	0.7903	0.09042
0.7644	0.08177	0.06902	0.08481

MOTIF CACGAAAT

0.09638	0.7245	0.1034	0.07581
0.8394	0.05716	0.03695	0.0665
0.1518	0.6503	0.07721	0.1207
0.1279	0.08487	0.7023	0.08489
0.8226	0.07359	0.05152	0.05225
0.7747	0.09417	0.0758	0.05536
0.7923	0.08446	0.05609	0.06712
0.1056	0.1114	0.06199	0.721

MOTIF GGTAAGTC

0.07098	0.06553	0.7463	0.1172

0.08154	0.05015	0.7769	0.09137
0.05918	0.08722	0.0886	0.765
0.7931	0.08899	0.06186	0.05602
0.7498	0.05109	0.06648	0.1326
0.06721	0.09542	0.7386	0.09873
0.06812	0.07454	0.09465	0.7627
0.1265	0.6887	0.07038	0.1144

MOTIF ACTGCGAG

0.7472	0.05517	0.09442	0.1032
0.08072	0.7523	0.09149	0.0755
0.07935	0.06519	0.0794	0.7761
0.1616	0.09534	0.6519	0.09116
0.08299	0.746	0.06813	0.1029
0.09186	0.08772	0.7116	0.1088
0.8197	0.06198	0.03721	0.08113
0.08876	0.06778	0.7778	0.06567

MOTIF TCGTAAGT

0.04626	0.0633	0.0494	0.841
0.1396	0.677	0.09378	0.08958
0.1328	0.05964	0.7154	0.09216
0.0796	0.07339	0.08082	0.7662
0.7845	0.06207	0.08577	0.06764
0.8224	0.03566	0.06926	0.07263
0.0902	0.05698	0.7646	0.0882
0.04915	0.07389	0.04984	0.8271

MOTIF GAAAGCTC

0.1027	0.1061	0.6715	0.1197
0.7171	0.101	0.1048	0.07716
0.7768	0.05296	0.0659	0.1043
0.8141	0.03593	0.08804	0.06192
0.1187	0.06835	0.7591	0.05382
0.08136	0.7062	0.09911	0.1133
0.09525	0.0593	0.07244	0.773
0.1257	0.6916	0.08487	0.09789

MOTIF ACCGTTCT

0.7301	0.09458	0.09469	0.08059
0.1325	0.6994	0.07231	0.09581
0.1254	0.7223	0.06894	0.08336
0.1021	0.07046	0.6184	0.209
0.06294	0.05416	0.07188	0.811
0.05034	0.07788	0.07833	0.7935
0.06631	0.6378	0.08835	0.2075
0.09768	0.1205	0.07565	0.7062

MOTIF GCATCGAT

0.0903	0.0865	0.7407	0.0825

0.148	0.728	0.06719	0.05676
0.764	0.05859	0.06681	0.1106
0.09541	0.08137	0.07755	0.7457
0.08539	0.7545	0.0726	0.08752
0.09575	0.08517	0.6982	0.1209
0.7763	0.0779	0.06566	0.08015
0.06254	0.08987	0.08586	0.7617

MOTIF TACCGT

0.06452	0.09755	0.07088	0.7671
0.7839	0.0606	0.07015	0.08533
0.05527	0.7843	0.06875	0.09163
0.06375	0.7869	0.05884	0.09052
0.1442	0.104	0.6075	0.1443
0.06555	0.03833	0.0676	0.8285

MOTIF AGCGAA

0.8501	0.04773	0.05298	0.04918
0.1048	0.06342	0.7747	0.05702
0.08405	0.7312	0.08459	0.1001
0.1078	0.07629	0.7135	0.1024
0.8752	0.04783	0.03859	0.03837
0.8106	0.06429	0.06788	0.05725

MOTIF AACGCG

0.7962	0.07239	0.07293	0.05843
0.8035	0.06296	0.05821	0.07529
0.1038	0.7292	0.08226	0.08479
0.1107	0.06688	0.7235	0.09885
0.09937	0.7702	0.06836	0.06212
0.07995	0.04437	0.822	0.05367

MOTIF CGCTTT

0.1141	0.7132	0.07263	0.1001
0.08273	0.08748	0.7462	0.08355
0.08484	0.7565	0.06867	0.08994
0.06537	0.06221	0.06719	0.8052
0.0426	0.0608	0.05667	0.8399
0.04887	0.1018	0.0646	0.7848

MOTIF CTCGTA

0.07047	0.8141	0.05937	0.0561
0.03391	0.04287	0.04706	0.8762
0.076	0.7446	0.07911	0.1003
0.08433	0.06989	0.7594	0.08641
0.0728	0.09259	0.05994	0.7747
0.777	0.07235	0.07329	0.0774

MOTIF GGTAAG

0.08498	0.05176	0.7998	0.06347

0.0639	0.06548	0.813	0.05761
0.07778	0.1059	0.07874	0.7375
0.7781	0.08915	0.07661	0.05615
0.8304	0.04303	0.04492	0.0816
0.07749	0.04466	0.801	0.07683

MOTIF AATGCG

0.8395	0.05133	0.0557	0.05352
0.8109	0.06167	0.07494	0.05249
0.08676	0.07531	0.07021	0.7677
0.06159	0.0561	0.7901	0.09219
0.09622	0.6745	0.1332	0.09611
0.1133	0.08957	0.6895	0.1076

MOTIF TTCACG

0.06703	0.06082	0.06141	0.8107
0.0493	0.05166	0.0546	0.8444
0.06145	0.8154	0.0664	0.05679
0.8361	0.05583	0.0487	0.05937
0.1048	0.7079	0.06839	0.1189
0.0911	0.08353	0.7201	0.1053

MOTIF CGTAAG

0.1201	0.6642	0.1274	0.08831
0.1077	0.0752	0.7848	0.03232
0.12	0.0627	0.06165	0.7557
0.7747	0.07872	0.08357	0.06296
0.8467	0.01877	0.07447	0.06008
0.07731	0.06024	0.8009	0.06157

MOTIF GTCATG

0.08797	0.06695	0.7571	0.08795
0.05851	0.07277	0.06044	0.8083
0.06727	0.7956	0.06434	0.07278
0.8053	0.05316	0.04868	0.09288
0.06521	0.04588	0.06092	0.828
0.05634	0.05705	0.8302	0.05639

MOTIF CGAAAG

0.1278	0.7089	0.07643	0.08693
0.1142	0.06715	0.7482	0.07048
0.8493	0.04956	0.04807	0.05309
0.8404	0.04711	0.06801	0.04444
0.8394	0.04958	0.0685	0.04255
0.09106	0.06946	0.7427	0.09678

MOTIF TACGAT

0.08398	0.07011	0.05673	0.7892
0.8274	0.04344	0.0574	0.07174
0.09769	0.6716	0.08111	0.1496

0.07652	0.1318	0.6884	0.1032
0.8583	0.04971	0.05805	0.03393
0.06159	0.06768	0.1117	0.759

MOTIF AGGGTC

0.7889	0.06768	0.05951	0.08387
0.1093	0.0583	0.7355	0.09695
0.09536	0.0705	0.758	0.07609
0.07582	0.09152	0.7365	0.09615
0.05447	0.04951	0.04976	0.8463
0.09533	0.786	0.05313	0.06555

MOTIF CGTTCT

0.09041	0.7146	0.07761	0.1174
0.08139	0.07852	0.6788	0.1612
0.05601	0.06846	0.05803	0.8175
0.05064	0.06363	0.0707	0.815
0.05385	0.7713	0.07601	0.09883
0.06655	0.06803	0.05829	0.8071

MOTIF CTTACT

0.05746	0.8255	0.04086	0.07614
0.06572	0.05436	0.03943	0.8405
0.09503	0.06089	0.05892	0.7852
0.6372	0.13	0.1236	0.1091
0.05667	0.809	0.05636	0.07799
0.05573	0.07811	0.04666	0.8195

MOTIF AGAGCG

0.7785	0.1003	0.0632	0.05794
0.1073	0.07494	0.7636	0.05425
0.7921	0.05567	0.0593	0.09297
0.09975	0.06897	0.7659	0.06533
0.1177	0.721	0.08592	0.07534
0.08708	0.0778	0.7274	0.1078

MOTIF TGATAG

0.05225	0.04678	0.05254	0.8484
0.05725	0.05916	0.8312	0.05238
0.7718	0.09695	0.08056	0.05072
0.08829	0.08936	0.07519	0.7472
0.7971	0.03958	0.07723	0.08605
0.09087	0.05781	0.7591	0.09224

MOTIF CGCACT

0.09053	0.7236	0.07708	0.1088
0.08561	0.07904	0.7233	0.1121
0.08892	0.8039	0.0504	0.05676
0.8022	0.05884	0.05534	0.08364
0.0602	0.7817	0.07145	0.08669

0.07988	0.05687	0.07448	0.7888

MOTIF AGCGTC

0.7955	0.0702	0.05362	0.08071
0.04015	0.07161	0.8251	0.06315
0.1067	0.7027	0.1044	0.08617
0.1046	0.06817	0.6691	0.1582
0.09331	0.06203	0.06448	0.7802
0.05316	0.798	0.09133	0.05748

MOTIF CGTATG

0.121	0.6507	0.1309	0.09743
0.04179	0.05399	0.7791	0.1251
0.06289	0.06319	0.058	0.8159
0.6609	0.08388	0.1319	0.1234
0.08092	0.02245	0.08248	0.8141
0.03334	0.06308	0.8224	0.08121

MOTIF TGTAGG

0.07989	0.06799	0.05974	0.7924
0.0586	0.07901	0.765	0.09736
0.1072	0.09937	0.07653	0.7169
0.7789	0.05426	0.0674	0.0994
0.08368	0.02901	0.808	0.07934
0.09533	0.06694	0.7392	0.09856

MOTIF ACTACG

0.8052	0.0673	0.07032	0.05721
0.06841	0.819	0.05052	0.06202
0.08461	0.08522	0.1044	0.7258
0.7711	0.0816	0.06407	0.08326
0.08403	0.7619	0.07179	0.08227
0.1018	0.09415	0.7276	0.07646

MOTIF AATGGG

0.7978	0.06518	0.07059	0.06639
0.8307	0.05696	0.05833	0.05401
0.08544	0.04965	0.04039	0.8245
0.05295	0.05304	0.8074	0.08659
0.0816	0.06526	0.7713	0.08184
0.1223	0.07881	0.7004	0.09854

MOTIF TAAGAC

0.08894	0.1251	0.08105	0.7049
0.8207	0.06413	0.06542	0.04976
0.8191	0.05559	0.04909	0.07618
0.1282	0.07238	0.7275	0.07195
0.8042	0.06252	0.06679	0.0665
0.08923	0.7511	0.07814	0.08155

MOTIF CACGAA

0.09339	0.7874	0.07544	0.0438
0.8457	0.05107	0.04305	0.0602
0.1221	0.7233	0.05542	0.09914
0.1024	0.08797	0.7333	0.07635
0.8234	0.08819	0.06101	0.02742
0.8318	0.05989	0.05828	0.05006

MOTIF GTCAAT

0.06923	0.05411	0.7879	0.08874
0.06812	0.05448	0.04592	0.8315
0.07966	0.7248	0.1146	0.08096
0.8432	0.05578	0.04936	0.05166
0.7683	0.09024	0.06651	0.07493
0.09199	0.07895	0.08829	0.7408

MOTIF TCTAACGCTA

0.08125	0.06746	0.07134	0.78
0.09689	0.7114	0.08392	0.1078
0.1033	0.08769	0.06987	0.7391
0.7887	0.07423	0.06135	0.07572
0.7367	0.08023	0.08372	0.09938
0.1658	0.605	0.09874	0.1305
0.1466	0.119	0.5969	0.1376
0.1068	0.7235	0.07685	0.09283
0.08266	0.07375	0.05376	0.7898
0.7616	0.08191	0.06291	0.09362

MOTIF GAACGCTAAC

0.134	0.0968	0.6803	0.08891
0.7772	0.08646	0.07586	0.06051
0.787	0.06628	0.07543	0.07126
0.1364	0.6479	0.1135	0.1022
0.1256	0.1125	0.6449	0.1169
0.1105	0.7169	0.08472	0.0878
0.1178	0.09933	0.07601	0.7068
0.7963	0.06808	0.07868	0.05698
0.7711	0.07304	0.08151	0.07439
0.1213	0.7009	0.08511	0.09272

MOTIF GGACGTTAAC

0.1072	0.07197	0.7264	0.09449
0.116	0.09484	0.7197	0.06943
0.7801	0.06974	0.06327	0.0869
0.1214	0.6864	0.09055	0.1017
0.1306	0.09957	0.6628	0.107
0.1192	0.09117	0.08636	0.7032
0.08962	0.09703	0.106	0.7073
0.7455	0.07725	0.0973	0.07992
0.7452	0.07348	0.08239	0.09888

0.108	0.7178	0.07545	0.09868

MOTIF GCCCGTTATA

0.1236	0.08598	0.6869	0.1035
0.1112	0.7138	0.07498	0.1
0.121	0.6869	0.07201	0.1201
0.1115	0.7027	0.07386	0.112
0.1254	0.09213	0.626	0.1565
0.09594	0.0814	0.05592	0.7667
0.07424	0.08585	0.08129	0.7586
0.7129	0.0863	0.08848	0.1123
0.09003	0.09219	0.07748	0.7403
0.743	0.08364	0.08347	0.08988

MOTIF TAAGCG

0.05464	0.04102	0.04329	0.8611
0.8597	0.04343	0.03885	0.05799
0.9039	0.03093	0.03355	0.03166
0.05947	0.05216	0.836	0.05233
0.06289	0.7819	0.07946	0.07574
0.06473	0.05498	0.7971	0.08319

MOTIF GATACCGTAC

0.104	0.09698	0.6989	0.1002
0.768	0.07957	0.07786	0.07457
0.1097	0.1214	0.06953	0.6993
0.7719	0.06584	0.08887	0.07335
0.0986	0.7161	0.08327	0.102
0.1227	0.6809	0.07807	0.1183
0.1401	0.09778	0.6486	0.1135
0.09715	0.07282	0.1082	0.7218
0.7322	0.09659	0.0844	0.08683
0.09549	0.7063	0.09213	0.1061

MOTIF ACGTTA

0.8973	0.03401	0.02686	0.04183
0.09028	0.7521	0.06564	0.09201
0.08639	0.0674	0.7661	0.08008
0.04238	0.03954	0.03533	0.8827
0.03931	0.04419	0.04373	0.8728
0.8849	0.0306	0.0394	0.04513

MOTIF CGCAAT

0.07326	0.8282	0.03628	0.06224
0.07246	0.04852	0.8225	0.05649
0.06566	0.8324	0.04895	0.05303
0.839	0.06517	0.04186	0.05399
0.9065	0.02862	0.02588	0.03903
0.04981	0.05055	0.0422	0.8574

MOTIF ATACGG

0.8655	0.04352	0.04396	0.04698
0.08911	0.04365	0.04092	0.8263
0.8842	0.0382	0.02577	0.05181
0.09214	0.751	0.06176	0.09506
0.05181	0.05222	0.8423	0.05366
0.04448	0.03546	0.875	0.04506

MOTIF ACGCTAAC

0.823	0.04997	0.0566	0.07046
0.1249	0.7049	0.07736	0.09287
0.09652	0.09776	0.7064	0.09936
0.08481	0.7791	0.07155	0.06458
0.0855	0.0921	0.05018	0.7722
0.8205	0.06409	0.06494	0.0505
0.7983	0.06343	0.06418	0.07408
0.0972	0.7712	0.06271	0.06889

MOTIF CGCTAT

0.07611	0.7841	0.06308	0.07669
0.06672	0.05325	0.7995	0.08055
0.04682	0.8612	0.04294	0.04907
0.04449	0.05523	0.03584	0.8644
0.8765	0.03977	0.03661	0.04709
0.04977	0.04279	0.04124	0.8662

MOTIF TTACGG

0.04342	0.03787	0.0385	0.8802
0.0441	0.04543	0.04932	0.8612
0.851	0.0412	0.04106	0.06677
0.08111	0.7495	0.08742	0.08197
0.06577	0.0487	0.8221	0.06341
0.04001	0.04153	0.8681	0.05033

MOTIF TATGCG

0.05144	0.0344	0.03638	0.8778
0.864	0.02683	0.04388	0.06526
0.05706	0.04106	0.04501	0.8569
0.05888	0.0732	0.8097	0.0582
0.06558	0.7706	0.07955	0.08424
0.05395	0.05292	0.7732	0.1199

MOTIF GCGATA

0.06289	0.0512	0.8388	0.04708
0.07143	0.7994	0.05311	0.07608
0.06657	0.06541	0.762	0.106
0.8769	0.04291	0.0414	0.03876
0.04846	0.04156	0.04422	0.8658
0.8826	0.04047	0.04011	0.0368

MOTIF TATCGG

0.03966	0.03188	0.03918	0.8893
0.8414	0.03963	0.0405	0.07844
0.04984	0.04108	0.05135	0.8577
0.07198	0.8067	0.05739	0.0639
0.05499	0.05637	0.8303	0.05832
0.04796	0.0415	0.847	0.06354

MOTIF CGGTAT

0.06884	0.7955	0.06172	0.07398
0.05492	0.05165	0.842	0.05142
0.05507	0.05312	0.8343	0.05748
0.05042	0.03292	0.03529	0.8814
0.8308	0.03537	0.05739	0.07649
0.05561	0.04376	0.03946	0.8612

MOTIF GCGTTA

0.05704	0.04657	0.8381	0.05831
0.07218	0.7703	0.07196	0.08554
0.06709	0.06181	0.7912	0.07989
0.03911	0.04093	0.03952	0.8804
0.04605	0.04711	0.05482	0.852
0.8683	0.03847	0.04735	0.04587

MOTIF TATAGCGG

0.0607	0.053	0.06654	0.8198
0.7769	0.06101	0.07733	0.08474
0.07034	0.05921	0.06347	0.807
0.8239	0.05065	0.06278	0.06262
0.07953	0.0603	0.7736	0.08659
0.1167	0.6819	0.08404	0.1173
0.09194	0.06767	0.7394	0.101
0.08383	0.07224	0.7531	0.09081

MOTIF CCGTTA

0.05175	0.8621	0.03303	0.0531
0.06097	0.8349	0.04527	0.05883
0.07076	0.06711	0.7862	0.07589
0.04367	0.04355	0.0356	0.8772
0.04101	0.04833	0.04429	0.8664
0.794	0.0526	0.07677	0.07659

MOTIF ACGTAG

0.8722	0.04844	0.03983	0.03952
0.04529	0.8539	0.05564	0.04516
0.04261	0.04201	0.8362	0.07918
0.05834	0.02925	0.04308	0.8693
0.7925	0.05239	0.09002	0.06511
0.06331	0.07514	0.8124	0.04917

MOTIF CGTTACGT

0.0975	0.7005	0.09466	0.1074
0.09115	0.06896	0.7455	0.09441
0.05386	0.05922	0.05924	0.8277
0.06154	0.08603	0.08325	0.7692
0.7592	0.07751	0.07296	0.0903
0.08237	0.7272	0.07498	0.1155
0.1073	0.08472	0.7104	0.09761
0.05962	0.05644	0.06067	0.8233

MOTIF TAACCG

0.06463	0.04861	0.05242	0.8343
0.8493	0.05296	0.04927	0.04849
0.8606	0.04828	0.04321	0.0479
0.05293	0.8637	0.03751	0.04587
0.06094	0.8312	0.04458	0.06325
0.07875	0.0659	0.771	0.08439

MOTIF AAAGCG

0.8819	0.03758	0.04004	0.04051
0.8706	0.03857	0.04198	0.04883
0.8969	0.03528	0.03287	0.03498
0.07535	0.06799	0.8128	0.04388
0.04636	0.7731	0.09011	0.09041
0.06922	0.04146	0.8015	0.08786

MOTIF TCGTTA

0.04591	0.0378	0.02976	0.8865
0.06868	0.7864	0.0702	0.07468
0.06638	0.05652	0.811	0.06607
0.03709	0.03502	0.03863	0.8893
0.03669	0.04258	0.03272	0.888
0.851	0.04078	0.04558	0.06259

MOTIF ACGTCT

0.8726	0.04846	0.03086	0.04809
0.0511	0.8737	0.03171	0.04352
0.04572	0.05449	0.8566	0.04322
0.04237	0.03473	0.0663	0.8566
0.05114	0.7912	0.07936	0.07828
0.07652	0.05974	0.05399	0.8098

MOTIF AATTACGG

0.8378	0.04343	0.05999	0.05873
0.8297	0.03956	0.05077	0.08001
0.08238	0.05824	0.04986	0.8095
0.06562	0.06096	0.06414	0.8093
0.7969	0.04997	0.05636	0.09673
0.1168	0.6339	0.1083	0.1411
0.1134	0.05746	0.7413	0.08782

0.08175	0.06603	0.7652	0.08703

MOTIF GAATACGG

0.1416	0.06484	0.7193	0.07426
0.8037	0.04021	0.0903	0.06578
0.7557	0.06353	0.08189	0.09891
0.1193	0.06588	0.05968	0.7551
0.8068	0.04829	0.05886	0.08603
0.1141	0.7064	0.08153	0.09795
0.06064	0.07515	0.7873	0.07688
0.08468	0.04582	0.7969	0.07261

MOTIF TCGTATGG

0.08777	0.07549	0.06219	0.7746
0.131	0.6487	0.1171	0.1032
0.08959	0.05674	0.7301	0.1236
0.05594	0.04947	0.05364	0.8409
0.7915	0.03984	0.07334	0.09536
0.08574	0.051	0.05743	0.8058
0.09805	0.08324	0.7218	0.09694
0.05048	0.06433	0.8167	0.06848

MOTIF AAATAGGATT

0.7664	0.07348	0.05798	0.1022
0.7747	0.08239	0.07122	0.07166
0.7678	0.08124	0.06296	0.08799
0.1151	0.06358	0.07919	0.7421
0.7757	0.06152	0.0774	0.08538
0.1075	0.0589	0.6965	0.1372
0.1283	0.09412	0.6847	0.09282
0.7589	0.07108	0.06377	0.1063
0.1139	0.04905	0.08474	0.7523
0.09285	0.06833	0.08618	0.7526

MOTIF GGTTCAAAGT

0.06486	0.05334	0.7494	0.1324
0.07992	0.1019	0.7042	0.114
0.105	0.06982	0.06663	0.7586
0.06776	0.05027	0.08623	0.7957
0.1124	0.7671	0.0632	0.05734
0.8089	0.08104	0.04276	0.06725
0.7965	0.05294	0.04845	0.1021
0.7625	0.06509	0.09084	0.08156
0.1113	0.07262	0.7029	0.1132
0.1001	0.0503	0.1014	0.7482

MOTIF GATCAGGGTT

0.1414	0.07537	0.7155	0.0677
0.7365	0.08496	0.07464	0.1039
0.0638	0.08103	0.09037	0.7648

0.1318	0.7166	0.05707	0.09453
0.8174	0.03407	0.07555	0.07293
0.1208	0.0635	0.6737	0.142
0.2051	0.06446	0.5915	0.1389
0.08621	0.07402	0.7422	0.09753
0.1239	0.05393	0.1114	0.7108
0.07567	0.03945	0.06775	0.8171

MOTIF GAGCCCAGTT

0.1098	0.07572	0.7238	0.09065
0.7765	0.09807	0.07296	0.05249
0.08491	0.09225	0.7445	0.07832
0.08688	0.7779	0.07891	0.05634
0.09436	0.7127	0.09808	0.09487
0.1152	0.6891	0.1129	0.08277
0.8283	0.04472	0.04999	0.07701
0.09882	0.09164	0.7155	0.09406
0.09032	0.06381	0.1223	0.7236
0.06609	0.0401	0.09194	0.8019

MOTIF TAATGGGA

0.1126	0.08984	0.07559	0.722
0.7444	0.07267	0.1065	0.07641
0.7964	0.05074	0.07235	0.08051
0.08052	0.02664	0.07035	0.8225
0.1115	0.04469	0.7471	0.09673
0.09748	0.04579	0.7457	0.111
0.1026	0.0861	0.7387	0.07256
0.7666	0.0827	0.05057	0.1001

MOTIF TGCCGAAC

0.09102	0.08128	0.07752	0.7502
0.08837	0.07805	0.7762	0.05737
0.1081	0.7382	0.09165	0.06204
0.1006	0.7665	0.04411	0.08886
0.1398	0.1048	0.6799	0.07556
0.8318	0.07631	0.04702	0.04485
0.758	0.09339	0.06721	0.08144
0.08059	0.7372	0.0938	0.08841

MOTIF GTATCCCG

0.1192	0.04657	0.7111	0.1232
0.122	0.06272	0.08115	0.7341
0.7651	0.08973	0.05753	0.08768
0.05054	0.08061	0.06497	0.8039
0.07108	0.7705	0.07789	0.08049
0.05958	0.8108	0.03781	0.0918
0.0648	0.7566	0.05714	0.1215
0.1335	0.07762	0.6887	0.1002

MOTIF CTGGGTGA

0.09883	0.6808	0.09742	0.123
0.0927	0.058	0.06823	0.7811
0.09703	0.1001	0.6631	0.1397
0.06484	0.05488	0.7918	0.08848
0.08184	0.07727	0.6974	0.1435
0.1101	0.05965	0.072	0.7582
0.08732	0.08004	0.7678	0.0648
0.7306	0.08941	0.09914	0.0809

MOTIF TCTTGGGT

0.06612	0.07712	0.06598	0.7908
0.09082	0.7223	0.08082	0.1061
0.07299	0.06976	0.06155	0.7957
0.05799	0.04493	0.08231	0.8148
0.05669	0.04962	0.7372	0.1565
0.0776	0.05347	0.7636	0.1053
0.1028	0.09489	0.7061	0.09621
0.07901	0.08389	0.09146	0.7456

MOTIF CAGGATTC

0.1025	0.6927	0.07487	0.1299
0.7875	0.05071	0.091	0.07074
0.1096	0.05765	0.7084	0.1244
0.1198	0.05336	0.7649	0.06192
0.734	0.05872	0.1154	0.09193
0.07883	0.07263	0.06477	0.7838
0.08417	0.07982	0.07479	0.7612
0.0786	0.7043	0.08044	0.1366

MOTIF CGGCTTAG

0.09507	0.6356	0.1825	0.08685
0.1271	0.0495	0.7481	0.07524
0.07692	0.08882	0.7616	0.07267
0.09281	0.7641	0.05376	0.08928
0.07814	0.06037	0.07016	0.7913
0.1353	0.08202	0.09073	0.6919
0.6935	0.07947	0.09946	0.1275
0.06107	0.03936	0.7928	0.1067

MOTIF CACCTTAC

0.1186	0.7145	0.08315	0.08371
0.7621	0.1046	0.03583	0.0974
0.08886	0.7359	0.06387	0.1113
0.1106	0.7533	0.0286	0.1075
0.1132	0.0941	0.05557	0.7371
0.08873	0.08178	0.07294	0.7565
0.6598	0.1212	0.1042	0.1148
0.1088	0.7127	0.06788	0.1106

MOTIF GTAAGGTA

0.1213	0.07894	0.6963	0.1035
0.1418	0.127	0.1037	0.6275
0.764	0.05891	0.08436	0.09274
0.7396	0.03475	0.08503	0.1406
0.1047	0.05906	0.7513	0.08497
0.1177	0.101	0.7017	0.07967
0.1066	0.07966	0.1046	0.7092
0.6152	0.09682	0.1489	0.139

MOTIF TACCCCGA

0.1073	0.09512	0.08519	0.7124
0.7313	0.09816	0.06683	0.1037
0.06606	0.6786	0.1006	0.1547
0.08321	0.7548	0.0782	0.08376
0.07056	0.7768	0.05753	0.09513
0.09815	0.7442	0.04344	0.1142
0.1511	0.09263	0.6513	0.105
0.7311	0.0809	0.0725	0.1155

MOTIF AGAACTCT

0.8002	0.07753	0.04977	0.07245
0.1653	0.1212	0.5888	0.1248
0.7782	0.09087	0.06273	0.06819
0.8152	0.07499	0.02195	0.0879
0.1156	0.758	0.09097	0.03539
0.07719	0.08692	0.04083	0.7951
0.07932	0.7832	0.0495	0.08793
0.1079	0.08674	0.05083	0.7545

MOTIF GTAAGG

0.1303	0.06881	0.7216	0.07937
0.1088	0.08056	0.09067	0.72
0.8239	0.06226	0.04979	0.06402
0.8111	0.04005	0.06801	0.08081
0.08374	0.03388	0.7865	0.09592
0.07238	0.04776	0.8062	0.07365

MOTIF ACCTAC

0.7942	0.05773	0.06181	0.08624
0.1024	0.7707	0.05465	0.07227
0.06614	0.8323	0.03519	0.06639
0.09794	0.07889	0.04084	0.7823
0.7512	0.1013	0.0624	0.0851
0.07196	0.7599	0.08194	0.08621

MOTIF CTCACG

0.08129	0.8104	0.04223	0.06604
0.05702	0.07497	0.04179	0.8262
0.06596	0.7933	0.06317	0.07757

0.7789	0.0802	0.0566	0.08425
0.1556	0.6189	0.08265	0.1429
0.09037	0.1094	0.7212	0.07905

MOTIF CCCAGT

0.0737	0.7902	0.06612	0.06997
0.06213	0.8225	0.05379	0.06159
0.07843	0.7588	0.1018	0.06098
0.8445	0.05241	0.03188	0.07125
0.08628	0.06276	0.7194	0.1315
0.09468	0.09026	0.08846	0.7266

MOTIF TGCGTA

0.08792	0.05015	0.07787	0.7841
0.01767	0.05614	0.8478	0.07839
0.08497	0.6704	0.1278	0.1169
0.1281	0.1475	0.6037	0.1208
0.06907	0.03775	0.08861	0.8046
0.7808	0.05265	0.08272	0.08381

MOTIF ATAGGA

0.7704	0.08334	0.06408	0.08216
0.07705	0.04461	0.07323	0.8051
0.7901	0.04387	0.09189	0.07409
0.06226	0.03341	0.8272	0.07711
0.05833	0.08747	0.7984	0.0558
0.8073	0.06996	0.05774	0.06501

MOTIF ATCCCG

0.8246	0.06885	0.05328	0.05323
0.05545	0.08844	0.07138	0.7847
0.07143	0.7954	0.0637	0.06945
0.06782	0.7783	0.05587	0.09799
0.07398	0.7209	0.06686	0.1383
0.09835	0.08003	0.7188	0.1028

MOTIF ATCGGC

0.7357	0.07231	0.09038	0.1016
0.07443	0.03709	0.06958	0.8189
0.098	0.7142	0.1073	0.08055
0.09308	0.06745	0.7713	0.06816
0.07081	0.05345	0.8258	0.04993
0.06459	0.817	0.05273	0.06568

MOTIF CAGGAT

0.0894	0.6929	0.08246	0.1353
0.8057	0.05394	0.08164	0.05872
0.1139	0.04228	0.7517	0.09209
0.07085	0.05084	0.8253	0.05296
0.806	0.04682	0.06979	0.07734

0.08111	0.04571	0.07111	0.8021

MOTIF ATTGCG

0.8019	0.06195	0.05523	0.08097
0.06987	0.05921	0.05911	0.8118
0.05835	0.05658	0.1035	0.7815
0.06147	0.06868	0.7871	0.08272
0.09089	0.689	0.09769	0.1224
0.1422	0.0948	0.6455	0.1175

MOTIF TTACTG

0.06329	0.08204	0.04468	0.81
0.05595	0.06824	0.0708	0.805
0.6864	0.09382	0.09469	0.1251
0.1122	0.6765	0.07454	0.1368
0.06266	0.04655	0.06429	0.8265
0.08579	0.06491	0.7576	0.09167

MOTIF TTACCC

0.07593	0.06693	0.06248	0.7947
0.06456	0.09862	0.05611	0.7807
0.7398	0.07812	0.08292	0.09913
0.1015	0.708	0.09294	0.09756
0.06451	0.8059	0.06749	0.06212
0.07466	0.7976	0.04478	0.08294

MOTIF CTACAC

0.07132	0.7901	0.06642	0.07214
0.1172	0.08746	0.04845	0.7469
0.7733	0.09887	0.04666	0.08116
0.08456	0.7862	0.07745	0.05183
0.7753	0.07398	0.06945	0.08124
0.1287	0.7463	0.05786	0.06711

MOTIF CGATTC

0.06354	0.7528	0.08769	0.09597
0.08424	0.1108	0.6918	0.1132
0.7995	0.07426	0.06603	0.06018
0.07786	0.0622	0.08299	0.777
0.02976	0.06379	0.05448	0.852
0.08245	0.8066	0.0376	0.07333

MOTIF GAACTC

0.1154	0.1103	0.704	0.07029
0.8139	0.04759	0.08386	0.05461
0.8173	0.08016	0.03674	0.06582
0.07216	0.8537	0.04243	0.03167
0.07399	0.06218	0.03913	0.8247
0.05393	0.7896	0.0649	0.09153

MOTIF CGCATT

0.1167	0.6976	0.07961	0.1061
0.1498	0.1458	0.5673	0.1371
0.08892	0.7879	0.08543	0.03776
0.8065	0.06058	0.04383	0.08906
0.07703	0.05928	0.05995	0.8037
0.06515	0.05736	0.07241	0.8051

MOTIF TTAACC

0.0644	0.074	0.05036	0.8112
0.07396	0.1075	0.09319	0.7254
0.7627	0.08158	0.0886	0.06716
0.7726	0.07713	0.07347	0.07685
0.0693	0.7674	0.05823	0.105
0.06678	0.8325	0.04239	0.05836

MOTIF AGCATACAGT

0.7894	0.04352	0.05512	0.1119
0.1163	0.08516	0.7266	0.07194
0.1043	0.7471	0.06755	0.08109
0.8017	0.06781	0.04661	0.0839
0.1374	0.07153	0.07623	0.7149
0.7677	0.0662	0.07508	0.091
0.08557	0.7811	0.06381	0.06953
0.7809	0.0517	0.08706	0.08029
0.1214	0.04129	0.7019	0.1355
0.1035	0.07003	0.05586	0.7707

MOTIF CTGGGAAAGC

0.1093	0.7137	0.07049	0.1065
0.1013	0.06022	0.08551	0.753
0.0798	0.08117	0.7276	0.1114
0.1192	0.05225	0.694	0.1345
0.0823	0.07074	0.7271	0.1199
0.7863	0.07577	0.08667	0.05121
0.6934	0.125	0.09057	0.09103
0.7914	0.07021	0.07406	0.06436
0.1513	0.1034	0.675	0.07027
0.1028	0.7537	0.07986	0.06373

MOTIF CGGGTAATTA

0.08699	0.7396	0.06232	0.1111
0.105	0.1486	0.6667	0.07971
0.08121	0.04412	0.794	0.08069
0.09346	0.05657	0.7886	0.06133
0.06074	0.0641	0.06856	0.8066
0.7504	0.09666	0.05951	0.09346
0.8358	0.0364	0.04874	0.07903
0.05478	0.04293	0.05292	0.8494
0.05078	0.03707	0.07443	0.8377

0.7199 0.05176 0.1249 0.1035

MOTIF ATCAATGG
0.7765 0.03385 0.06271 0.1269
0.07002 0.06569 0.07795 0.7863
0.08163 0.7634 0.06798 0.08695
0.7757 0.08913 0.07513 0.06009
0.7666 0.06736 0.09318 0.07283
0.08059 0.06445 0.05424 0.8007
0.0738 0.05118 0.7543 0.1207
0.1095 0.112 0.7005 0.07796

MOTIF AACGCTGC
0.8035 0.07187 0.06479 0.05982
0.7843 0.07614 0.06788 0.0717
0.09496 0.6888 0.07847 0.1378
0.09655 0.07128 0.7212 0.111
0.05136 0.793 0.078 0.07768
0.06109 0.08267 0.05945 0.7968
0.05247 0.04452 0.8301 0.07292
0.09829 0.7644 0.06326 0.07402

MOTIF CTGTCCAT
0.07958 0.6863 0.09474 0.1394
0.07005 0.07042 0.05399 0.8055
0.1203 0.1325 0.6035 0.1437
0.08038 0.078 0.05215 0.7895
0.05958 0.7446 0.06601 0.1298
0.1213 0.7437 0.05467 0.08035
0.8361 0.06047 0.03767 0.06573
0.06755 0.09291 0.06262 0.7769

MOTIF GACGCTGT
0.1243 0.06739 0.7329 0.07544
0.7335 0.09274 0.07872 0.09503
0.08621 0.7198 0.09171 0.1023
0.1227 0.08102 0.7264 0.06988
0.08804 0.7638 0.07234 0.07583
0.07271 0.05992 0.05551 0.8119
0.06817 0.06229 0.8185 0.05106
0.1375 0.1035 0.06116 0.6978

MOTIF TCGCTGAA
0.08984 0.08005 0.06258 0.7675
0.08624 0.7191 0.1006 0.09408
0.1263 0.08072 0.6957 0.09729
0.1037 0.7276 0.06743 0.1013
0.06341 0.04322 0.03956 0.8538
0.08975 0.09905 0.757 0.05424
0.7742 0.07372 0.08933 0.06276

0.7816	0.07637	0.05482	0.08721

MOTIF TCTTCAGG

0.05452	0.03644	0.06015	0.8489
0.1109	0.6376	0.1024	0.1492
0.08646	0.06978	0.06375	0.78
0.06447	0.07619	0.08814	0.7712
0.03598	0.8111	0.05414	0.0988
0.7904	0.04508	0.05645	0.108
0.08712	0.04934	0.7191	0.1445
0.08344	0.1185	0.6704	0.1277

MOTIF AATTACCC

0.7893	0.08518	0.05444	0.07105
0.8325	0.05032	0.05229	0.06486
0.09176	0.071	0.0584	0.7788
0.0819	0.09902	0.06148	0.7576
0.7488	0.08451	0.08429	0.08244
0.08988	0.7086	0.06864	0.1329
0.1149	0.7157	0.07196	0.09734
0.1113	0.6931	0.09148	0.1041

MOTIF CCTGGTAG

0.126	0.6877	0.08385	0.1025
0.1328	0.6929	0.07473	0.0995
0.06651	0.04522	0.05961	0.8287
0.06274	0.045	0.7883	0.1039
0.09031	0.08425	0.7648	0.06062
0.08202	0.08254	0.05977	0.7757
0.702	0.08512	0.1198	0.09309
0.08681	0.09328	0.736	0.08395

MOTIF AACAGTTT

0.7399	0.09837	0.05734	0.1044
0.7359	0.07365	0.08826	0.1022
0.09727	0.7215	0.09583	0.08541
0.7275	0.04531	0.06792	0.1593
0.1052	0.08694	0.7167	0.09118
0.06865	0.06575	0.0549	0.8107
0.06563	0.04073	0.1134	0.7803
0.09482	0.08891	0.09681	0.7195

MOTIF AAACTGTA

0.761	0.07854	0.09358	0.06686
0.7893	0.07493	0.05974	0.07601
0.7832	0.07177	0.06047	0.08454
0.1103	0.6901	0.08691	0.1127
0.1203	0.05365	0.05994	0.7661
0.08774	0.08051	0.7403	0.0914
0.1518	0.09614	0.08525	0.6668

0.7011	0.06667	0.1137	0.1186

MOTIF ACGCTG

0.7893	0.05578	0.07587	0.07908
0.08937	0.7212	0.1023	0.08718
0.09486	0.09314	0.725	0.087
0.06125	0.7979	0.07315	0.06774
0.0462	0.05879	0.04032	0.8547
0.05199	0.0393	0.8768	0.03191

MOTIF TACCGC

0.1049	0.1333	0.0644	0.6974
0.7665	0.07599	0.09592	0.06157
0.07595	0.7546	0.06478	0.1047
0.1166	0.7031	0.0398	0.1405
0.09042	0.06013	0.7932	0.05623
0.08327	0.7798	0.0668	0.07011

MOTIF ATCGCT

0.8017	0.06792	0.06715	0.06319
0.07242	0.06078	0.02447	0.8423
0.08869	0.6936	0.08623	0.1315
0.1033	0.06905	0.7163	0.1114
0.03071	0.8469	0.05463	0.06772
0.08478	0.06639	0.04733	0.8015

MOTIF GCATAC

0.0716	0.087	0.7576	0.08376
0.06869	0.8565	0.05129	0.02351
0.8381	0.07102	0.04681	0.0441
0.1025	0.04753	0.07674	0.7732
0.8025	0.1057	0.03113	0.06061
0.06616	0.7407	0.09971	0.0934

MOTIF ATGCGG

0.8102	0.05562	0.06108	0.07306
0.07037	0.04652	0.09402	0.7891
0.05978	0.05315	0.8018	0.08524
0.09918	0.722	0.0825	0.09636
0.1274	0.07051	0.6982	0.1038
0.1029	0.06082	0.7932	0.04301

MOTIF TAACGC

0.07098	0.08363	0.1048	0.7406
0.7838	0.05213	0.09306	0.07102
0.7356	0.09145	0.0676	0.1053
0.1325	0.6682	0.07842	0.1209
0.1035	0.07265	0.7373	0.08651
0.06506	0.7988	0.07563	0.0605

MOTIF GAACGC

0.05645	0.06359	0.8181	0.0619
0.838	0.04039	0.06524	0.0564
0.8147	0.04969	0.06557	0.06999
0.111	0.7247	0.1054	0.05893
0.09034	0.1096	0.7019	0.09816
0.07439	0.7798	0.09306	0.05275

MOTIF GGATGC

0.05178	0.06691	0.7963	0.08498
0.06132	0.03154	0.7995	0.1076
0.7924	0.06861	0.06933	0.06963
0.07336	0.05858	0.07591	0.7922
0.05331	0.0184	0.8519	0.07642
0.1139	0.7038	0.1193	0.06303

MOTIF CTGGTA

0.1115	0.765	0.05557	0.0679
0.06945	0.04406	0.06432	0.8222
0.05983	0.04356	0.8111	0.08548
0.07531	0.07842	0.7887	0.05756
0.07603	0.06619	0.06844	0.7893
0.7464	0.05842	0.1038	0.09139

MOTIF CAGAAT

0.08256	0.7711	0.08835	0.05804
0.8409	0.05794	0.03389	0.06724
0.1188	0.08562	0.7416	0.05395
0.793	0.06636	0.06741	0.07324
0.803	0.0549	0.07154	0.0706
0.09561	0.08596	0.05696	0.7615

MOTIF GTGACG

0.07606	0.08372	0.7766	0.06359
0.08948	0.1008	0.05583	0.7539
0.05526	0.0615	0.8233	0.05992
0.7562	0.07075	0.1021	0.07091
0.1227	0.6489	0.07944	0.149
0.09082	0.07778	0.7641	0.06728

MOTIF GTAGGC

0.04875	0.06115	0.8408	0.04932
0.122	0.09044	0.09069	0.6968
0.7653	0.05071	0.08013	0.1039
0.05742	0.05912	0.7687	0.1148
0.06255	0.07133	0.7937	0.07247
0.1021	0.7802	0.04991	0.06782

MOTIF CCACTG

0.08515	0.768	0.05843	0.08843

0.07997	0.8061	0.04654	0.06738
0.8206	0.0594	0.05637	0.0636
0.08709	0.7108	0.07708	0.125
0.04685	0.07946	0.03798	0.8357
0.07271	0.06502	0.7765	0.08576

MOTIF CAGAGT

0.1136	0.7662	0.04755	0.07263
0.8482	0.04389	0.04699	0.06088
0.1049	0.07819	0.7383	0.07864
0.7357	0.09157	0.08655	0.08615
0.1037	0.06228	0.7541	0.07989
0.06975	0.06622	0.06397	0.8001

MOTIF CTGTGA

0.09221	0.7429	0.07309	0.09183
0.1041	0.04679	0.08118	0.7679
0.06662	0.08141	0.738	0.114
0.07809	0.05209	0.07836	0.7915
0.08646	0.07819	0.7701	0.06528
0.7879	0.08124	0.05489	0.07595

MOTIF CTATCG

0.06732	0.7956	0.05202	0.08502
0.06714	0.119	0.0265	0.7874
0.7721	0.06818	0.09396	0.06571
0.06463	0.0732	0.0557	0.8065
0.03574	0.7517	0.06116	0.1514
0.1658	0.1187	0.6119	0.1036

MOTIF GTTCAG

0.07335	0.08144	0.7356	0.1096
0.1019	0.07749	0.05139	0.7693
0.05334	0.05299	0.04726	0.8464
0.04695	0.805	0.06593	0.0821
0.8091	0.04004	0.0561	0.0948
0.09651	0.05219	0.7225	0.1288

MOTIF GGGTTC

0.08153	0.08157	0.7304	0.1065
0.09789	0.08184	0.7332	0.08708
0.0723	0.05477	0.7603	0.1127
0.04659	0.04073	0.03777	0.8749
0.06567	0.04107	0.07445	0.8188
0.05508	0.7829	0.06542	0.09655

MOTIF AATTGC

0.8075	0.06862	0.05195	0.07193
0.8099	0.05539	0.07014	0.06454
0.06616	0.05524	0.04916	0.8294

0.07072	0.04047	0.07224	0.8166
0.1013	0.05939	0.7581	0.08122
0.09563	0.759	0.06479	0.0806

MOTIF CCCATCCACG

0.05467	0.7747	0.06158	0.109
0.05241	0.7488	0.08036	0.1184
0.04656	0.8639	0.04384	0.04568
0.7408	0.09951	0.06014	0.09959
0.08644	0.0755	0.05155	0.7865
0.05131	0.8314	0.06875	0.04858
0.06784	0.8208	0.06294	0.04844
0.7436	0.09482	0.06961	0.09199
0.08342	0.7392	0.06474	0.1127
0.09789	0.101	0.7135	0.08753

MOTIF GCATCCACGC

0.07987	0.1603	0.6314	0.1284
0.04727	0.7814	0.1088	0.06256
0.7508	0.1044	0.06474	0.08007
0.0687	0.07783	0.1029	0.7506
0.07565	0.7905	0.09006	0.04384
0.06073	0.8548	0.04442	0.04002
0.7708	0.06765	0.06595	0.09562
0.1293	0.7089	0.07739	0.08445
0.07129	0.1333	0.7181	0.07733
0.06229	0.7207	0.08592	0.1311

MOTIF ATCCACGC

0.7793	0.08553	0.06728	0.06792
0.06516	0.05568	0.09783	0.7813
0.0777	0.7598	0.1158	0.04667
0.04839	0.8471	0.05909	0.04539
0.7806	0.08035	0.06135	0.07768
0.1109	0.7136	0.06937	0.1061
0.08895	0.09052	0.7447	0.07581
0.06693	0.7635	0.07945	0.09008

MOTIF CGAATCGC

0.08764	0.8175	0.05552	0.0394
0.08755	0.1036	0.7608	0.04804
0.7408	0.1153	0.09223	0.05165
0.7052	0.07611	0.0931	0.1256
0.0538	0.07315	0.09552	0.7775
0.05832	0.7896	0.08875	0.06332
0.09342	0.07989	0.7707	0.05596
0.08058	0.7842	0.07652	0.05865

MOTIF GCCTCCCT

0.09002	0.1376	0.668	0.1044

0.07024	0.8155	0.06436	0.04987
0.08695	0.733	0.06019	0.1199
0.0751	0.08443	0.1062	0.7342
0.062	0.742	0.09804	0.09797
0.07066	0.7655	0.0738	0.09008
0.1267	0.7015	0.08121	0.09066
0.1033	0.1373	0.05856	0.7009

MOTIF AGCATATG
0.7789	0.05289	0.04405	0.1242
0.08035	0.09999	0.74	0.07962
0.0996	0.7109	0.1148	0.07477
0.8156	0.05823	0.06108	0.06508
0.08077	0.1102	0.1218	0.6872
0.713	0.1049	0.06184	0.1203
0.05527	0.05921	0.05439	0.8311
0.05959	0.06027	0.8045	0.07567

MOTIF CGTCCAAC
0.08417	0.7802	0.05644	0.07916
0.1378	0.099	0.6501	0.1131
0.08332	0.1103	0.07777	0.7286
0.07589	0.7823	0.07202	0.06982
0.07477	0.7796	0.08452	0.06113
0.7721	0.0829	0.07294	0.07203
0.6982	0.1246	0.08997	0.08724
0.09083	0.7598	0.07948	0.06988

MOTIF TTCCCAAA
0.09665	0.071	0.07031	0.762
0.09434	0.1065	0.07851	0.7206
0.06822	0.7177	0.1151	0.09898
0.1078	0.6948	0.06208	0.1353
0.1132	0.7806	0.04995	0.05622
0.8113	0.07845	0.04084	0.06939
0.7895	0.08507	0.05163	0.07381
0.7197	0.1293	0.06742	0.08357

MOTIF TGCCGAAC
0.05364	0.1117	0.06681	0.7679
0.0661	0.0474	0.7996	0.08694
0.06978	0.7076	0.1319	0.09077
0.1267	0.6856	0.06591	0.1217
0.1289	0.1011	0.7199	0.05008
0.7487	0.1267	0.06628	0.0584
0.7618	0.06097	0.08119	0.09603
0.05049	0.8072	0.07249	0.0698

MOTIF ATTCTCCG
0.6935	0.1176	0.1042	0.08477

0.07022	0.09974	0.04632	0.7837
0.06827	0.1405	0.1017	0.6896
0.05381	0.8009	0.07325	0.07204
0.06932	0.07914	0.1261	0.7255
0.089	0.7638	0.06115	0.08606
0.09419	0.746	0.07198	0.08782
0.1882	0.0849	0.6308	0.0961

MOTIF ACTACCAT

0.7094	0.103	0.08663	0.101
0.08262	0.7796	0.0618	0.07602
0.132	0.1477	0.05456	0.6658
0.7356	0.1023	0.06608	0.09601
0.09883	0.7051	0.0865	0.1095
0.07939	0.8088	0.05677	0.05503
0.8205	0.06628	0.05024	0.06298
0.1187	0.09617	0.1099	0.6753

MOTIF GCGAAT

0.05312	0.05717	0.8157	0.07405
0.09249	0.7416	0.1119	0.05394
0.0929	0.08702	0.7778	0.04228
0.7976	0.06934	0.06852	0.0645
0.7754	0.07514	0.06811	0.08136
0.08056	0.06863	0.1048	0.746

MOTIF CCGCAC

0.06104	0.8374	0.05408	0.04743
0.07453	0.7909	0.06221	0.07234
0.07904	0.0744	0.7721	0.07444
0.06054	0.8232	0.07779	0.03844
0.7555	0.08696	0.06627	0.09132
0.06698	0.8517	0.04305	0.03825

MOTIF TACCGC

0.09798	0.1155	0.09296	0.6935
0.6621	0.09985	0.1367	0.1014
0.03771	0.825	0.08653	0.05078
0.07854	0.7507	0.06195	0.1088
0.08417	0.08627	0.7604	0.06914
0.07877	0.8204	0.05469	0.04611

MOTIF ACGCCC

0.7115	0.1017	0.08196	0.1049
0.07457	0.8039	0.04625	0.07533
0.07536	0.07792	0.779	0.06773
0.06423	0.8151	0.05409	0.0666
0.07035	0.7974	0.06509	0.06713
0.0565	0.8301	0.06647	0.04694

MOTIF GCGCTA

0.05401	0.08391	0.7942	0.06787
0.07654	0.7939	0.05567	0.07388
0.08482	0.08814	0.7379	0.08917
0.049	0.8311	0.08391	0.03601
0.09945	0.1218	0.1009	0.6779
0.7517	0.07509	0.1139	0.05937

MOTIF TGCCGC

0.0695	0.1613	0.07755	0.6917
0.05489	0.06473	0.8141	0.0663
0.04478	0.8152	0.0762	0.06376
0.09278	0.7238	0.07956	0.1039
0.07775	0.09162	0.7668	0.06378
0.05352	0.8429	0.05036	0.05324

MOTIF GCGATC

0.05287	0.0756	0.8033	0.06828
0.06419	0.7792	0.07336	0.08321
0.07709	0.09659	0.7731	0.05326
0.7901	0.06733	0.08255	0.05997
0.06957	0.1056	0.06987	0.755
0.04114	0.7528	0.1215	0.08451

MOTIF GCTGTA

0.06224	0.06295	0.8071	0.06768
0.07717	0.7437	0.07454	0.1046
0.1008	0.06007	0.08116	0.758
0.07294	0.08658	0.7966	0.0439
0.1163	0.1416	0.09012	0.652
0.7304	0.0782	0.1144	0.07692

MOTIF AGGACT

0.7422	0.07543	0.08059	0.1018
0.09132	0.07223	0.768	0.06848
0.09642	0.05158	0.8351	0.01686
0.8228	0.03054	0.1037	0.04293
0.0892	0.7339	0.09091	0.08599
0.1141	0.07239	0.1294	0.6841

MOTIF GCGTGC

0.0571	0.07038	0.8202	0.05234
0.05565	0.773	0.07465	0.09672
0.02715	0.1026	0.7939	0.07634
0.06011	0.0273	0.07856	0.834
0.03577	0.0724	0.8451	0.04672
0.03345	0.7885	0.1015	0.0766

MOTIF GTCTAC

0.08786	0.1075	0.6464	0.1582

0.0703	0.05204	0.08561	0.7921
0.02005	0.8657	0.06853	0.04575
0.093	0.1006	0.03178	0.7746
0.7183	0.07779	0.08927	0.1147
0.07809	0.8074	0.05266	0.0619

MOTIF ACTACC

0.8072	0.06384	0.05664	0.07228
0.08377	0.8097	0.05122	0.05527
0.1132	0.1282	0.0698	0.6889
0.7673	0.0752	0.08893	0.0686
0.07585	0.7798	0.06466	0.07967
0.0799	0.8351	0.04071	0.04432

MOTIF ATTGCC

0.7536	0.07623	0.06784	0.1023
0.08059	0.08813	0.09186	0.7394
0.07737	0.1082	0.07556	0.7389
0.05162	0.109	0.7828	0.0566
0.03929	0.8295	0.08704	0.04418
0.09113	0.7949	0.04715	0.06685

MOTIF CGGGCA

0.07587	0.7508	0.1122	0.06112
0.08465	0.05983	0.7787	0.07685
0.07318	0.07166	0.7601	0.09506
0.08676	0.06324	0.7978	0.0522
0.05535	0.8473	0.06304	0.03433
0.7341	0.06192	0.1151	0.08893

MOTIF TAGCGA

0.09113	0.09434	0.0655	0.749
0.7702	0.02615	0.1209	0.08275
0.05002	0.08158	0.7958	0.07265
0.1097	0.7329	0.07747	0.07998
0.08148	0.09394	0.7974	0.02714
0.7726	0.09176	0.08417	0.0515

MOTIF TTTCCGGCCT

0.04295	0.1192	0.1238	0.7141
0.04779	0.07104	0.1104	0.7707
0.03498	0.09769	0.1501	0.7173
0.0449	0.6831	0.1357	0.1363
0.06828	0.7935	0.05663	0.08155
0.07464	0.07373	0.7787	0.07297
0.09911	0.08594	0.6763	0.1387
0.05453	0.801	0.05817	0.08628
0.08818	0.7344	0.09734	0.08012
0.09655	0.0902	0.1456	0.6677

MOTIF ATGGCGCAAA

0.7779	0.06817	0.08461	0.06931
0.09515	0.09643	0.14	0.6684
0.1233	0.08999	0.6977	0.08893
0.08387	0.1032	0.7204	0.0926
0.1115	0.675	0.112	0.1015
0.0937	0.1128	0.7038	0.08964
0.1019	0.742	0.09024	0.06588
0.8244	0.05282	0.07861	0.04419
0.7027	0.09231	0.0659	0.1391
0.7835	0.06895	0.1111	0.03643

MOTIF TTGCGTGCGA

0.0705	0.1047	0.07181	0.753
0.0865	0.03258	0.106	0.7749
0.05879	0.09826	0.7567	0.08629
0.1041	0.6855	0.09257	0.1178
0.06539	0.08233	0.7101	0.1422
0.07192	0.07954	0.1491	0.6995
0.05302	0.1484	0.7392	0.05938
0.1007	0.6734	0.1151	0.1109
0.09315	0.08282	0.7107	0.1133
0.7448	0.1081	0.06891	0.07816

MOTIF TCGGAGCAAA

0.1335	0.1291	0.07759	0.6598
0.1756	0.6657	0.07379	0.0849
0.1538	0.08498	0.7045	0.0567
0.08877	0.06998	0.771	0.07025
0.7577	0.07506	0.1078	0.05938
0.06706	0.09982	0.7575	0.07564
0.08399	0.7665	0.09972	0.04982
0.8098	0.09099	0.05514	0.0441
0.8306	0.07709	0.05525	0.03711
0.7614	0.1186	0.05599	0.06397

MOTIF TTGTACGC

0.07162	0.05102	0.04663	0.8307
0.05295	0.0422	0.05956	0.8453
0.0452	0.05579	0.8295	0.06951
0.06547	0.1098	0.08372	0.741
0.7083	0.08935	0.1007	0.1017
0.08162	0.7173	0.08852	0.1126
0.05986	0.07851	0.7767	0.08494
0.05411	0.7403	0.1188	0.0868

MOTIF TTCCGGCC

0.0546	0.04593	0.1148	0.7847
0.03513	0.1041	0.08422	0.7766
0.06499	0.6827	0.116	0.1363

0.04207	0.8077	0.06069	0.08959
0.06215	0.07119	0.7816	0.08509
0.05313	0.07002	0.7756	0.1012
0.05187	0.8261	0.07202	0.04998
0.05915	0.7684	0.1031	0.0694

MOTIF AACGTCCA

0.8045	0.07598	0.06701	0.05246
0.6951	0.1716	0.06775	0.06555
0.07978	0.6901	0.1457	0.08439
0.05413	0.09089	0.766	0.08898
0.08411	0.09444	0.09298	0.7285
0.07302	0.7964	0.07243	0.05817
0.07169	0.804	0.06016	0.06419
0.7746	0.07601	0.08291	0.06651

MOTIF TGGTCCTG

0.0844	0.06405	0.07509	0.7765
0.05302	0.06219	0.7838	0.101
0.0612	0.08727	0.7348	0.1167
0.0896	0.144	0.07358	0.6929
0.06238	0.7541	0.1147	0.06884
0.07953	0.7796	0.06832	0.07258
0.07491	0.06312	0.1096	0.7524
0.06109	0.08726	0.7715	0.08014

MOTIF CAGGGCCA

0.1082	0.719	0.1052	0.06765
0.7897	0.07379	0.06292	0.07354
0.08311	0.06401	0.7854	0.06746
0.08009	0.07266	0.7545	0.09278
0.1378	0.07738	0.6923	0.09245
0.09227	0.7537	0.09802	0.05602
0.1125	0.7811	0.0595	0.04686
0.7607	0.1027	0.0639	0.07264

MOTIF TTGCGCGC

0.07566	0.08352	0.0593	0.7815
0.07654	0.06778	0.09447	0.7612
0.05901	0.0987	0.7809	0.06142
0.1005	0.7178	0.07419	0.1075
0.1166	0.09828	0.6919	0.09324
0.0611	0.7129	0.1284	0.09759
0.0842	0.08802	0.7255	0.1023
0.04187	0.783	0.0978	0.07737

MOTIF AAGAGCAA

0.7491	0.1075	0.07647	0.06698
0.7505	0.04967	0.1287	0.07121
0.08376	0.07553	0.7757	0.06502

0.7617	0.07407	0.1008	0.0634
0.09325	0.07836	0.7619	0.06645
0.1073	0.7053	0.136	0.05135
0.8162	0.05561	0.06423	0.06391
0.7803	0.08166	0.07991	0.05809

MOTIF ATACGGGA
0.7029	0.09461	0.1175	0.08507
0.1253	0.1102	0.08887	0.6757
0.7405	0.05987	0.09535	0.1042
0.1015	0.6933	0.1265	0.07879
0.07619	0.1264	0.7294	0.068
0.07537	0.08327	0.7894	0.05201
0.0676	0.07141	0.762	0.09895
0.7758	0.05433	0.07329	0.0966

MOTIF TGCCGATT
0.05595	0.09169	0.1453	0.7071
0.05819	0.1031	0.7437	0.09505
0.0699	0.7686	0.09639	0.06515
0.1063	0.6813	0.1023	0.1101
0.07247	0.1455	0.7234	0.05861
0.6874	0.1357	0.08521	0.09171
0.08663	0.07933	0.07408	0.76
0.07412	0.1013	0.1174	0.7072

MOTIF AAGGGCCA
0.6611	0.1598	0.1095	0.06965
0.7875	0.06928	0.07744	0.06582
0.07659	0.07973	0.7782	0.06545
0.08523	0.0548	0.7783	0.08166
0.07662	0.09595	0.7136	0.1138
0.06568	0.7449	0.1143	0.07519
0.1469	0.7187	0.06423	0.07012
0.7426	0.09504	0.07542	0.08689

MOTIF ATCGGGAA
0.6913	0.08533	0.1393	0.08407
0.1136	0.09104	0.1023	0.6931
0.1111	0.7073	0.1063	0.07531
0.07403	0.05437	0.807	0.06458
0.06407	0.05963	0.8225	0.05383
0.08739	0.1345	0.6959	0.08217
0.7653	0.1052	0.08501	0.04453
0.8381	0.05455	0.0677	0.03963

MOTIF CTACGGTA
0.08642	0.714	0.1155	0.08404
0.1351	0.06941	0.1001	0.6954
0.764	0.06844	0.08207	0.08545

0.09202	0.7308	0.1007	0.07648
0.106	0.09247	0.7061	0.09541
0.05693	0.09559	0.8065	0.04102
0.06512	0.109	0.09813	0.7277
0.7433	0.07548	0.07726	0.1039

MOTIF GTTACG

0.06674	0.06639	0.7919	0.07498
0.07409	0.08456	0.07186	0.7695
0.09712	0.08579	0.08625	0.7308
0.7267	0.1065	0.09158	0.07517
0.06569	0.7879	0.06591	0.08046
0.07511	0.05664	0.8028	0.06545

MOTIF ACCGTA

0.8266	0.04883	0.07334	0.05118
0.04614	0.8501	0.06136	0.04235
0.08245	0.7792	0.08466	0.0537
0.0726	0.07547	0.7763	0.07565
0.07038	0.06733	0.1661	0.6962
0.7405	0.1399	0.05678	0.06286

MOTIF GCGCAA

0.04462	0.07285	0.8375	0.04498
0.1379	0.7082	0.08845	0.06551
0.06061	0.06958	0.7873	0.08252
0.04651	0.8475	0.06525	0.04073
0.8437	0.07094	0.04151	0.04389
0.7944	0.05598	0.07458	0.07508

MOTIF AGGGCC

0.8124	0.0608	0.05401	0.07275
0.06755	0.04478	0.8238	0.06387
0.06439	0.03129	0.8319	0.0724
0.06686	0.05614	0.8011	0.0759
0.09719	0.7754	0.0706	0.05678
0.1086	0.7816	0.07064	0.03915

MOTIF CGTATC

0.07501	0.787	0.06414	0.0738
0.0437	0.09134	0.7901	0.07484
0.08114	0.1047	0.09321	0.721
0.6801	0.09777	0.1098	0.1123
0.1241	0.07745	0.09333	0.7051
0.07331	0.7472	0.0927	0.08679

MOTIF CGTACA

0.06456	0.8301	0.0803	0.02502
0.07984	0.06597	0.777	0.07721
0.09566	0.08201	0.2374	0.585

0.7852	0.08981	0.08631	0.03866
0.05972	0.8415	0.05261	0.04615
0.8573	0.05634	0.03201	0.05438

MOTIF CTTACG

0.06512	0.7527	0.109	0.07316
0.08124	0.09121	0.03887	0.7887
0.05234	0.07125	0.113	0.7634
0.6147	0.1247	0.1803	0.08032
0.02586	0.8395	0.08515	0.04945
0.1097	0.07251	0.7437	0.07414

MOTIF ATCGGC

0.6691	0.05702	0.08227	0.1916
0.08338	0.06779	0.0938	0.755
0.05516	0.8115	0.07511	0.05827
0.0597	0.08588	0.787	0.06744
0.04098	0.06966	0.8253	0.06408
0.07411	0.8127	0.0534	0.05977

MOTIF CGGACA

0.07478	0.825	0.05543	0.04477
0.04373	0.05862	0.8503	0.04733
0.0655	0.05917	0.824	0.05131
0.8191	0.08282	0.04604	0.05207
0.0771	0.8359	0.04825	0.03878
0.7357	0.09108	0.09388	0.07936

MOTIF AAGAGC

0.807	0.05282	0.0745	0.06569
0.8099	0.04747	0.076	0.06662
0.05466	0.05775	0.8407	0.04689
0.7637	0.06644	0.1008	0.06902
0.06675	0.05833	0.8176	0.05736
0.1081	0.7406	0.1002	0.05109

MOTIF AACGTC

0.8329	0.07426	0.0261	0.06675
0.675	0.2003	0.05842	0.06622
0.0582	0.7594	0.1164	0.06596
0.05979	0.06779	0.7762	0.09625
0.05698	0.09347	0.08468	0.7649
0.06724	0.8408	0.06913	0.0228

MOTIF TAGTCA

0.1148	0.1248	0.08678	0.6736
0.6787	0.1075	0.1087	0.1052
0.03988	0.07369	0.8379	0.04857
0.08929	0.08925	0.128	0.6935
0.09092	0.7716	0.08493	0.05258

0.6628	0.1631	0.09101	0.08307

MOTIF GCCGTA

0.0851	0.05413	0.8018	0.05896
0.05358	0.8119	0.08236	0.05219
0.05486	0.8357	0.06263	0.0468
0.06931	0.09779	0.771	0.06191
0.1784	0.08034	0.1252	0.6161
0.7832	0.05547	0.08155	0.07975

MOTIF CCTACG

0.04559	0.8297	0.06632	0.05835
0.06753	0.8391	0.0317	0.06169
0.09208	0.08728	0.1095	0.7111
0.6388	0.06573	0.1105	0.185
0.07136	0.8291	0.04698	0.05258
0.07202	0.09141	0.7474	0.08919

MOTIF GTCCCT

0.03647	0.06055	0.7837	0.1193
0.06465	0.07833	0.1432	0.7138
0.07438	0.7612	0.07615	0.08826
0.0526	0.8354	0.06347	0.04856
0.05589	0.8058	0.07138	0.06689
0.06013	0.07395	0.07047	0.7955

MOTIF CGGAAA

0.09323	0.7616	0.07427	0.07091
0.04735	0.1073	0.7954	0.05
0.1018	0.08513	0.7747	0.03835
0.7911	0.07117	0.07926	0.05846
0.7519	0.1579	0.05037	0.03982
0.8329	0.05804	0.06503	0.04403

MOTIF CGGGAA

0.1279	0.7216	0.0889	0.06154
0.08421	0.08317	0.7728	0.0598
0.06229	0.07153	0.8217	0.04444
0.09536	0.09053	0.741	0.07307
0.798	0.06229	0.08412	0.05559
0.7979	0.07795	0.07038	0.05382

MOTIF CGGGCCGCGC

0.04457	0.8056	0.09156	0.05832
0.07139	0.09664	0.7496	0.08236
0.06094	0.09265	0.7614	0.08496
0.05807	0.1563	0.7308	0.05485
0.0515	0.8026	0.08476	0.06117
0.04984	0.8103	0.08584	0.05403
0.08036	0.09369	0.7555	0.0704

0.07754	0.6252	0.2362	0.06103
0.06669	0.1431	0.7393	0.05088
0.06166	0.7931	0.08823	0.05697

MOTIF CTAAGCACGT

0.1013	0.764	0.0483	0.08635
0.08978	0.1259	0.06474	0.7196
0.779	0.09493	0.06373	0.06239
0.7784	0.09657	0.06646	0.0586
0.1768	0.04527	0.7066	0.07138
0.05822	0.7714	0.08377	0.08662
0.7538	0.1132	0.04884	0.08418
0.1023	0.7562	0.06707	0.07443
0.04012	0.08054	0.7984	0.08099
0.1697	0.08987	0.06112	0.6793

MOTIF CTGTGGCCTA

0.08118	0.6967	0.1042	0.118
0.06996	0.07873	0.09234	0.759
0.1067	0.1258	0.691	0.0765
0.08299	0.07772	0.07159	0.7677
0.04949	0.05391	0.8332	0.06341
0.1328	0.06887	0.7039	0.09446
0.08331	0.7041	0.1408	0.07175
0.09595	0.7837	0.0715	0.04881
0.1038	0.1081	0.072	0.7161
0.7031	0.08987	0.1249	0.08216

MOTIF CATACGCGGG

0.08381	0.7263	0.08778	0.1022
0.6849	0.07984	0.1665	0.06881
0.0767	0.1744	0.08138	0.6675
0.7144	0.09824	0.123	0.06429
0.08694	0.7337	0.09583	0.08353
0.0797	0.08042	0.7617	0.07817
0.06446	0.7724	0.05846	0.1047
0.1125	0.07183	0.7392	0.07649
0.12	0.09511	0.682	0.1029
0.05834	0.08775	0.7142	0.1397

MOTIF CATGGACGTG

0.07271	0.7508	0.09546	0.081
0.7131	0.07368	0.111	0.1022
0.1039	0.05805	0.08611	0.752
0.06456	0.08856	0.786	0.06091
0.07218	0.1022	0.7603	0.0653
0.7314	0.05206	0.126	0.09051
0.1201	0.6798	0.1022	0.09788
0.06705	0.08421	0.749	0.09975
0.1111	0.09453	0.1015	0.6929

0.07559	0.08553	0.7715	0.06738

MOTIF CACACGTG

0.093	0.7551	0.07991	0.072
0.7111	0.1254	0.0709	0.09257
0.06021	0.8283	0.05028	0.06125
0.8083	0.09322	0.04191	0.05659
0.07947	0.7695	0.1267	0.02432
0.09868	0.07631	0.7017	0.1233
0.0672	0.101	0.07289	0.7589
0.1182	0.1095	0.7184	0.05392

MOTIF GGCGTCAC

0.05106	0.1536	0.7184	0.07699
0.08458	0.08864	0.7671	0.05969
0.06481	0.7449	0.07334	0.1169
0.04868	0.1382	0.7339	0.07928
0.08089	0.1152	0.1529	0.651
0.05328	0.7761	0.1109	0.05975
0.7221	0.09958	0.1117	0.06661
0.06253	0.7881	0.05738	0.09198

MOTIF CCAAGCGA

0.07316	0.7278	0.1385	0.06048
0.06462	0.8365	0.06563	0.03328
0.7804	0.05516	0.07147	0.09297
0.6655	0.145	0.1086	0.08089
0.09841	0.05644	0.7873	0.0579
0.08846	0.7193	0.09031	0.1019
0.1102	0.1042	0.7319	0.05366
0.6798	0.1317	0.1139	0.07453

MOTIF AGGCCACA

0.7055	0.08687	0.0895	0.1181
0.0665	0.07524	0.7474	0.1109
0.07493	0.08932	0.7532	0.08256
0.09165	0.7627	0.05562	0.09006
0.06821	0.8149	0.06433	0.05259
0.7309	0.08921	0.08865	0.09123
0.08941	0.7235	0.1206	0.06651
0.7539	0.1073	0.09395	0.04482

MOTIF ACGTGACA

0.7069	0.08371	0.1476	0.06182
0.05915	0.7535	0.08881	0.0985
0.08632	0.07325	0.7685	0.07193
0.08113	0.07255	0.1157	0.7307
0.03922	0.09633	0.8102	0.05429
0.6307	0.1169	0.1525	0.09994
0.09091	0.7369	0.1042	0.06799

0.7044	0.11	0.1148	0.07086

MOTIF TGCGGCCA

0.07263	0.09255	0.1555	0.6793
0.06105	0.09664	0.7686	0.07367
0.08394	0.7416	0.05335	0.1212
0.07994	0.0822	0.746	0.09189
0.05956	0.05842	0.7889	0.09308
0.04841	0.7994	0.08746	0.06473
0.1187	0.7498	0.06958	0.06199
0.7429	0.08445	0.08524	0.08745

MOTIF GGCGGACA

0.05279	0.09714	0.7916	0.05844
0.0706	0.1224	0.7474	0.0596
0.06791	0.8204	0.05892	0.0528
0.1078	0.0719	0.7436	0.07667
0.06853	0.145	0.6903	0.0962
0.6256	0.1681	0.1471	0.05918
0.06761	0.7993	0.08562	0.04746
0.6422	0.123	0.1409	0.09392

MOTIF CCCGCGTA

0.0776	0.8322	0.06324	0.02693
0.08773	0.752	0.08957	0.07075
0.06622	0.7843	0.05394	0.09553
0.1234	0.06349	0.7681	0.04497
0.08456	0.778	0.05422	0.08319
0.07612	0.1116	0.7327	0.07958
0.08664	0.1401	0.06431	0.7089
0.6379	0.1733	0.13	0.05879

MOTIF TGCGTA

0.04197	0.06385	0.0595	0.8347
0.01988	0.03997	0.8946	0.0456
0.05661	0.7608	0.1035	0.07907
0.07906	0.07327	0.7696	0.07807
0.05588	0.03093	0.04645	0.8667
0.7301	0.07369	0.137	0.05917

MOTIF CGTACT

0.01875	0.8226	0.09023	0.06838
0.04516	0.04501	0.8409	0.06897
0.09499	0.0598	0.1135	0.7317
0.7293	0.1019	0.1281	0.0407
0.03102	0.8406	0.05945	0.06895
0.06984	0.0564	0.02923	0.8445

MOTIF CGGGCC

0.02891	0.8919	0.03968	0.03948

0.0661	0.09475	0.7701	0.06908
0.05642	0.05067	0.8466	0.04631
0.03377	0.0819	0.8452	0.03909
0.04086	0.8605	0.04641	0.05224
0.04002	0.8406	0.05562	0.06373

MOTIF GGCCTA

0.06351	0.05056	0.8259	0.06002
0.04466	0.04919	0.859	0.04716
0.04693	0.7863	0.1039	0.06284
0.07197	0.8326	0.05372	0.0417
0.09097	0.07928	0.1127	0.7171
0.666	0.1092	0.1442	0.0806

MOTIF CGCGTT

0.04719	0.8435	0.05136	0.05792
0.06129	0.1254	0.7411	0.07225
0.07433	0.7287	0.1144	0.0826
0.04853	0.07271	0.8306	0.04814
0.05712	0.07328	0.07845	0.7911
0.06112	0.08925	0.1258	0.7238

MOTIF CATACG

0.08755	0.8047	0.04043	0.06729
0.8347	0.07707	0.03992	0.04829
0.08609	0.19	0.05961	0.6643
0.7081	0.09939	0.1365	0.05605
0.06814	0.8384	0.06005	0.03342
0.09309	0.07031	0.7812	0.05537

MOTIF TAGTCG

0.1044	0.08645	0.08218	0.727
0.6045	0.1702	0.1182	0.1071
0.05189	0.1001	0.798	0.04992
0.05931	0.116	0.08096	0.7437
0.06979	0.7422	0.1168	0.07124
0.07002	0.08511	0.7822	0.06271

MOTIF CCCGAT

0.04025	0.8437	0.06226	0.0538
0.08103	0.798	0.07097	0.04996
0.09102	0.7449	0.1019	0.06213
0.03637	0.08447	0.8385	0.04064
0.6619	0.1052	0.09399	0.1389
0.07393	0.1128	0.1184	0.6949

MOTIF GTCACG

0.07095	0.06388	0.7833	0.08188
0.1063	0.1098	0.0969	0.6869
0.04503	0.8278	0.1002	0.0269

0.761	0.09074	0.07192	0.0763
0.07042	0.8235	0.0512	0.0549
0.08792	0.07471	0.7863	0.05109

MOTIF CTATCG

0.09019	0.7537	0.07853	0.0776
0.1202	0.1142	0.02721	0.7384
0.6211	0.1189	0.136	0.124
0.08357	0.1165	0.1621	0.6378
0.02013	0.7479	0.1761	0.05588
0.0852	0.06203	0.7958	0.05693

MOTIF AACGAC

0.6653	0.1291	0.124	0.08159
0.7971	0.07587	0.079	0.048
0.04721	0.8138	0.07904	0.05997
0.06255	0.06566	0.8234	0.04834
0.8242	0.06254	0.07526	0.038
0.06807	0.807	0.09016	0.0348

MOTIF AATACG

0.7733	0.09434	0.04965	0.08267
0.7224	0.08378	0.1477	0.04616
0.07679	0.1302	0.1213	0.6717
0.7865	0.06942	0.079	0.0651
0.05682	0.8207	0.05836	0.06415
0.05292	0.06986	0.8357	0.04154

MOTIF CGCTTG

0.08361	0.7752	0.06495	0.07625
0.08825	0.08782	0.7458	0.07808
0.0372	0.8655	0.03898	0.05835
0.08961	0.08534	0.1091	0.7159
0.0638	0.06834	0.09049	0.7774
0.04339	0.05432	0.8582	0.04407

MOTIF CACGGA

0.0319	0.867	0.07084	0.03026
0.7507	0.09527	0.07272	0.08127
0.05447	0.7674	0.07736	0.1007
0.07676	0.04994	0.8265	0.04676
0.07621	0.1173	0.7327	0.07384
0.7699	0.09296	0.06594	0.07117

MOTIF CGTGGG

0.05207	0.8162	0.07675	0.05496
0.09686	0.0596	0.761	0.08251
0.0471	0.1134	0.08212	0.7574
0.04131	0.04093	0.8322	0.08554
0.05148	0.05904	0.7823	0.1071

0.0718	0.09384	0.7717	0.0626

MOTIF TGCGGT

0.04285	0.0935	0.08963	0.774
0.04932	0.1176	0.7824	0.0507
0.07501	0.7035	0.1201	0.1014
0.08814	0.0874	0.758	0.06646
0.04042	0.07521	0.8172	0.06719
0.03814	0.1068	0.1065	0.7486

MOTIF TACCCG

0.1179	0.0855	0.1301	0.6665
0.7539	0.06985	0.09872	0.07749
0.03939	0.8332	0.08174	0.04567
0.07192	0.7947	0.06722	0.06621
0.05394	0.783	0.08935	0.07367
0.09791	0.08785	0.7762	0.03801

MOTIF ATTGCG

0.6425	0.1425	0.1168	0.09819
0.06908	0.1038	0.08983	0.7372
0.06734	0.08272	0.1195	0.7305
0.04532	0.05363	0.8352	0.06589
0.04643	0.8002	0.04888	0.1045
0.0701	0.09166	0.7758	0.06248

MOTIF GCAGTA

0.08067	0.0875	0.7973	0.03456
0.0411	0.8021	0.08365	0.07313
0.7196	0.08372	0.1276	0.06902
0.04277	0.1055	0.8098	0.04194
0.06577	0.1	0.1114	0.7228
0.7497	0.08477	0.102	0.06361

MOTIF GAAACG

0.08047	0.101	0.7591	0.05936
0.7167	0.1194	0.09259	0.0713
0.6781	0.177	0.1008	0.04416
0.7556	0.1018	0.09502	0.04754
0.08171	0.7766	0.068	0.07368
0.06131	0.07948	0.8062	0.05302

MOTIF CGTGTG

0.05118	0.7566	0.09024	0.102
0.06877	0.07524	0.7819	0.07413
0.05554	0.09843	0.06559	0.7804
0.04622	0.03925	0.8648	0.04976
0.04952	0.06181	0.09842	0.7903
0.05017	0.08737	0.802	0.0605

MOTIF ATCACG

0.6794	0.08177	0.1344	0.1045
0.09778	0.09959	0.1423	0.6603
0.04062	0.8489	0.06983	0.04068
0.7878	0.06851	0.07272	0.07097
0.07033	0.7956	0.06925	0.06487
0.1065	0.06772	0.7737	0.05211

MOTIF GGCGTA

0.04543	0.06589	0.7825	0.1062
0.04331	0.0717	0.8129	0.07212
0.06659	0.776	0.08485	0.07253
0.08576	0.1285	0.7375	0.04825
0.109	0.1164	0.1356	0.639
0.7696	0.08834	0.08783	0.05423

MOTIF GTAGTCTGCC

0.09666	0.115	0.6893	0.09902
0.1184	0.08572	0.05836	0.7375
0.6597	0.1254	0.09862	0.1163
0.07028	0.0847	0.7473	0.09773
0.09791	0.09048	0.06732	0.7443
0.09113	0.6885	0.1098	0.1106
0.09139	0.06944	0.0805	0.7587
0.1026	0.07742	0.7128	0.1072
0.0692	0.7843	0.06595	0.0805
0.1185	0.6699	0.04825	0.1633

MOTIF CTACCTTGAA

0.09588	0.6714	0.1273	0.1054
0.1125	0.08812	0.05235	0.747
0.7726	0.07496	0.04695	0.1055
0.1092	0.7242	0.09458	0.07198
0.1068	0.6974	0.05233	0.1434
0.07368	0.05687	0.05526	0.8142
0.0801	0.05233	0.06751	0.8001
0.0989	0.08991	0.6939	0.1173
0.7153	0.09346	0.06907	0.1222
0.7318	0.07986	0.1096	0.07876

MOTIF ACTAGAATCA

0.7875	0.05611	0.07639	0.08002
0.1067	0.6744	0.09696	0.1219
0.07843	0.06384	0.04327	0.8145
0.8142	0.05809	0.04667	0.08104
0.106	0.1149	0.716	0.06307
0.7607	0.08064	0.05732	0.1013
0.7585	0.07449	0.06278	0.1043
0.1002	0.1091	0.102	0.6887
0.1323	0.6968	0.06616	0.1047

0.7793 0.06716 0.06061 0.09294

MOTIF TTGTCAAGCA
0.09556 0.08928 0.09272 0.7224
0.07274 0.06782 0.1062 0.7533
0.1116 0.07945 0.6883 0.1206
0.1034 0.108 0.1107 0.6778
0.1002 0.7049 0.07025 0.1247
0.8185 0.05856 0.02552 0.09743
0.7923 0.04497 0.0626 0.1002
0.1319 0.05696 0.7289 0.08228
0.1053 0.6593 0.09244 0.1429
0.735 0.071 0.08982 0.1042

MOTIF TCAAGGTA
0.1088 0.09411 0.08837 0.7087
0.0741 0.7546 0.08469 0.08665
0.8059 0.05719 0.05561 0.08133
0.8253 0.0549 0.05447 0.06532
0.1082 0.06367 0.705 0.1232
0.09015 0.1041 0.6971 0.1087
0.1085 0.08198 0.07457 0.735
0.7373 0.07344 0.1011 0.08813

MOTIF CGTACTAG
0.1431 0.6176 0.06632 0.173
0.09869 0.1047 0.7152 0.08142
0.09342 0.06963 0.07851 0.7584
0.7784 0.05635 0.09551 0.06969
0.06212 0.7645 0.1059 0.06751
0.07353 0.04841 0.06379 0.8143
0.7969 0.04034 0.05815 0.1046
0.09829 0.1044 0.7513 0.04599

MOTIF TTGCGTGT
0.09119 0.06211 0.07696 0.7697
0.07434 0.03723 0.06532 0.8231
0.0841 0.0874 0.7279 0.1006
0.1004 0.6702 0.08234 0.1471
0.1264 0.09984 0.6405 0.1333
0.07162 0.04577 0.05585 0.8268
0.06572 0.06042 0.7862 0.08771
0.05632 0.08547 0.1014 0.7568

MOTIF CGTATGCT
0.08789 0.6803 0.1173 0.1146
0.166 0.1253 0.5947 0.1139
0.07005 0.058 0.0431 0.8289
0.6908 0.08465 0.1283 0.09622
0.0585 0.05403 0.05717 0.8303

0.04974	0.05657	0.821	0.07268
0.05739	0.7936	0.06324	0.08579
0.1172	0.03556	0.03577	0.8114

MOTIF ACACTGGT

0.8058	0.0476	0.07957	0.06705
0.0946	0.7738	0.07506	0.05651
0.8545	0.04312	0.03871	0.06368
0.0704	0.739	0.08234	0.1082
0.0947	0.05432	0.03654	0.8144
0.132	0.08213	0.7093	0.07649
0.06125	0.1064	0.7499	0.08243
0.1208	0.03915	0.04986	0.7902

MOTIF CGCTTTAG

0.134	0.5801	0.0827	0.2032
0.12	0.07321	0.7187	0.08802
0.05847	0.7984	0.07287	0.07024
0.08429	0.06056	0.06344	0.7917
0.04249	0.08928	0.07292	0.7953
0.07149	0.06246	0.1152	0.7509
0.751	0.06169	0.09131	0.09595
0.07856	0.1105	0.7248	0.08612

MOTIF TGTACTAC

0.08713	0.09126	0.05986	0.7618
0.07813	0.1438	0.6739	0.1042
0.06702	0.06201	0.06579	0.8052
0.7128	0.07579	0.1209	0.09049
0.06689	0.7082	0.1315	0.09343
0.07836	0.06736	0.06418	0.7901
0.7878	0.05386	0.05702	0.1013
0.1655	0.6228	0.1486	0.0631

MOTIF TGCTTAGG

0.07888	0.04113	0.04405	0.8359
0.07993	0.05838	0.7774	0.08428
0.08732	0.733	0.07426	0.1054
0.0965	0.06393	0.05146	0.7881
0.08251	0.04324	0.07214	0.8021
0.6766	0.07014	0.1336	0.1197
0.1468	0.06597	0.6231	0.1641
0.1225	0.08913	0.6822	0.1061

MOTIF TCTACTAC

0.08343	0.1347	0.0728	0.7091
0.0751	0.6949	0.1292	0.1007
0.1164	0.06548	0.06294	0.7552
0.7432	0.07972	0.08227	0.09484
0.09344	0.7072	0.1335	0.06586

0.1032	0.09305	0.05283	0.7509
0.7728	0.08056	0.06674	0.07994
0.1143	0.7191	0.09336	0.07325

MOTIF GGTATGCA

0.1118	0.117	0.6618	0.1094
0.09154	0.08975	0.6881	0.1306
0.102	0.07209	0.08157	0.7443
0.7806	0.06263	0.07351	0.08327
0.1181	0.05593	0.07813	0.7478
0.0441	0.0672	0.8188	0.06991
0.09282	0.7366	0.08819	0.08235
0.7682	0.05	0.0457	0.1361

MOTIF GTAGTAGG

0.06923	0.1062	0.7514	0.07313
0.09318	0.07721	0.1009	0.7288
0.744	0.05514	0.09225	0.1086
0.05625	0.1246	0.7264	0.09277
0.1136	0.07729	0.08774	0.7214
0.8161	0.05146	0.05266	0.07975
0.08892	0.08785	0.7275	0.09572
0.1532	0.07917	0.6763	0.09134

MOTIF TAGTAC

0.09162	0.04444	0.04141	0.8225
0.8328	0.047	0.05025	0.07
0.0644	0.118	0.7652	0.0524
0.06967	0.08757	0.05681	0.786
0.8103	0.0638	0.06808	0.05778
0.06767	0.7466	0.1067	0.07904

MOTIF TTAACG

0.05732	0.04031	0.055	0.8474
0.0912	0.09883	0.04983	0.7602
0.7834	0.08547	0.07811	0.05298
0.806	0.07601	0.07114	0.04687
0.09609	0.7406	0.08791	0.07536
0.1426	0.09652	0.6146	0.1463

MOTIF GGTCTA

0.09621	0.0741	0.7589	0.07081
0.1272	0.07657	0.7095	0.0867
0.08282	0.04664	0.04707	0.8235
0.06793	0.7571	0.08348	0.09154
0.07483	0.08411	0.05503	0.786
0.7767	0.04805	0.05939	0.1159

MOTIF ACGTAA

0.8801	0.03822	0.04062	0.04104

0.1017	0.6944	0.09622	0.1077
0.1287	0.1386	0.6114	0.1213
0.0675	0.09388	0.05761	0.781
0.8216	0.04682	0.06953	0.06206
0.7501	0.1186	0.06853	0.06268

MOTIF GAACGC

0.06418	0.1224	0.751	0.06243
0.7723	0.08945	0.06883	0.06939
0.8202	0.07082	0.06089	0.04804
0.1179	0.7045	0.08306	0.09452
0.152	0.1104	0.6374	0.1002
0.05979	0.7859	0.09027	0.06408

MOTIF GCATAC

0.09617	0.07808	0.7575	0.06824
0.06758	0.8569	0.05464	0.02092
0.8279	0.06367	0.04445	0.06398
0.09363	0.1114	0.08648	0.7085
0.8301	0.05038	0.04059	0.0789
0.08833	0.7347	0.09513	0.08181

MOTIF AGGCAA

0.8548	0.03426	0.03834	0.07257
0.1288	0.07279	0.7029	0.09551
0.0862	0.08287	0.7712	0.05977
0.09218	0.7587	0.07549	0.07362
0.8689	0.03979	0.04375	0.04761
0.8117	0.06838	0.08339	0.03658

MOTIF GCGCTT

0.06066	0.1114	0.7567	0.0713
0.1152	0.6515	0.06595	0.1674
0.1366	0.07615	0.6635	0.1238
0.04326	0.8309	0.06665	0.05923
0.06595	0.06678	0.06587	0.8014
0.04489	0.06299	0.05322	0.8389

MOTIF ATACGC

0.811	0.05657	0.07631	0.0561
0.06962	0.1492	0.06643	0.7147
0.7517	0.09043	0.07656	0.0813
0.1338	0.6464	0.08704	0.1327
0.1418	0.1497	0.6036	0.1048
0.08735	0.77	0.04926	0.09334

MOTIF TTGCGT

0.05345	0.04894	0.07675	0.8209
0.04874	0.03744	0.05047	0.8633
0.06571	0.0487	0.8293	0.05627

0.1279	0.6472	0.08821	0.1366
0.1058	0.1375	0.6191	0.1375
0.05637	0.03683	0.03859	0.8682

MOTIF GAGTCT

0.1038	0.06201	0.7565	0.07768
0.8028	0.04063	0.07302	0.08358
0.09063	0.092	0.7355	0.08185
0.09631	0.05666	0.06986	0.7772
0.06502	0.7905	0.08322	0.0613
0.05655	0.07453	0.06832	0.8006

MOTIF GCTTAA

0.09868	0.06185	0.7528	0.08668
0.0637	0.7543	0.07242	0.1096
0.07443	0.05208	0.03115	0.8423
0.1061	0.08002	0.0452	0.7687
0.718	0.07286	0.1114	0.09774
0.795	0.05747	0.07594	0.07162

MOTIF CCGCTT

0.05646	0.7878	0.09028	0.06549
0.08329	0.6657	0.06398	0.187
0.1112	0.08873	0.6916	0.1084
0.04701	0.8373	0.05555	0.06014
0.04438	0.1012	0.06224	0.7922
0.05246	0.06987	0.04961	0.8281

MOTIF TGTAGG

0.07653	0.03617	0.08164	0.8057
0.05761	0.06339	0.7798	0.09915
0.05869	0.06343	0.09586	0.782
0.8001	0.0418	0.07398	0.0841
0.07431	0.05547	0.7605	0.1097
0.06721	0.07045	0.7457	0.1166

MOTIF ACGCTA

0.8332	0.06081	0.04733	0.05863
0.1573	0.6068	0.0743	0.1617
0.1343	0.1342	0.5922	0.1392
0.05577	0.7812	0.08479	0.07824
0.1191	0.07659	0.05421	0.7501
0.828	0.04634	0.06144	0.06427

MOTIF GGTAGG

0.08335	0.03602	0.7821	0.09852
0.07685	0.07884	0.785	0.05927
0.1054	0.07269	0.08387	0.738
0.7593	0.07698	0.06026	0.1035
0.0755	0.0522	0.7735	0.0988

0.08903	0.05456	0.8031	0.05334

MOTIF GCTTGA

0.08431	0.08721	0.7561	0.07239
0.05894	0.7946	0.07004	0.0764
0.03494	0.04151	0.04448	0.8791
0.07488	0.03272	0.07677	0.8156
0.07548	0.06751	0.7653	0.09167
0.7459	0.08257	0.09877	0.07274

MOTIF TGGCAT

0.04339	0.03367	0.03958	0.8834
0.1088	0.04783	0.7357	0.1077
0.08187	0.1209	0.6884	0.1088
0.0914	0.771	0.0525	0.08505
0.8324	0.0444	0.05323	0.06999
0.07824	0.08761	0.07735	0.7568

MOTIF GATTCTGGAG

0.08364	0.05893	0.7813	0.07617
0.7142	0.1131	0.06919	0.1036
0.09841	0.05456	0.05848	0.7886
0.05184	0.07243	0.1266	0.7492
0.07885	0.7227	0.08843	0.1101
0.08491	0.06877	0.03367	0.8126
0.08285	0.08465	0.7376	0.09491
0.07444	0.09071	0.7611	0.07374
0.7628	0.05988	0.0961	0.08124
0.1152	0.0647	0.7428	0.07725

MOTIF ATTTACTACT

0.7275	0.06642	0.0725	0.1336
0.1023	0.138	0.06697	0.6928
0.09315	0.07608	0.06598	0.7648
0.05793	0.0794	0.0562	0.8065
0.6974	0.08982	0.09174	0.121
0.1173	0.6168	0.08358	0.1823
0.08498	0.0973	0.05858	0.7591
0.7519	0.06902	0.06663	0.1124
0.1156	0.64	0.0957	0.1487
0.06786	0.05362	0.04783	0.8307

MOTIF GGCCAGGGTT

0.0966	0.08288	0.7458	0.07475
0.05228	0.09529	0.7576	0.09481
0.1051	0.7418	0.07529	0.07782
0.1561	0.6763	0.09252	0.07509
0.7692	0.04021	0.09483	0.09577
0.1188	0.07143	0.7268	0.08295
0.07492	0.06461	0.7816	0.07886

0.07233	0.09916	0.7057	0.1228
0.06983	0.0909	0.1006	0.7387
0.05864	0.07968	0.08956	0.7721

MOTIF GTGAGTGA

0.07623	0.07292	0.7482	0.1026
0.09143	0.06446	0.1121	0.732
0.07587	0.05603	0.7884	0.07972
0.7451	0.05077	0.06067	0.1434
0.07824	0.06423	0.7281	0.1295
0.1037	0.05909	0.08581	0.7514
0.06904	0.07379	0.7841	0.07306
0.7845	0.05062	0.07146	0.09339

MOTIF TCCAAACG

0.05841	0.06179	0.06252	0.8173
0.1031	0.71	0.09661	0.09023
0.08715	0.7866	0.06119	0.06508
0.8367	0.05739	0.04487	0.06102
0.8144	0.04721	0.08342	0.05501
0.7657	0.07088	0.07193	0.09153
0.1153	0.6631	0.1047	0.1169
0.1214	0.07765	0.71	0.09094

MOTIF TCACAGCG

0.07526	0.07385	0.08151	0.7694
0.05301	0.7989	0.07754	0.0706
0.7634	0.05674	0.094	0.08589
0.08169	0.789	0.06435	0.06498
0.7994	0.05832	0.05417	0.08811
0.1162	0.1167	0.6983	0.06886
0.08994	0.716	0.06516	0.1289
0.09944	0.07424	0.6791	0.1472

MOTIF AGTAGTAA

0.8423	0.05595	0.04432	0.05745
0.1444	0.1147	0.6322	0.1086
0.08091	0.06402	0.07077	0.7843
0.8091	0.03893	0.07135	0.0806
0.1292	0.09228	0.6746	0.104
0.1102	0.08675	0.08152	0.7215
0.8009	0.0426	0.09121	0.06528
0.7701	0.08146	0.07863	0.06981

MOTIF GTAAAGCT

0.1111	0.1068	0.6638	0.1184
0.1004	0.07796	0.0982	0.7235
0.7238	0.1146	0.08514	0.07646
0.7451	0.09182	0.106	0.05712
0.8045	0.06268	0.07381	0.05901

0.125	0.07447	0.7179	0.08264
0.1151	0.7193	0.0882	0.07741
0.1237	0.08244	0.04881	0.7451

MOTIF TTAAGCCC

0.07056	0.09524	0.07251	0.7617
0.09021	0.1087	0.07583	0.7253
0.7389	0.09486	0.08666	0.07963
0.8077	0.05044	0.06399	0.07782
0.09392	0.09789	0.6816	0.1266
0.08561	0.7046	0.111	0.09876
0.1361	0.6923	0.04096	0.1306
0.09927	0.7137	0.07324	0.1138

MOTIF GTCATTCA

0.1047	0.1109	0.6935	0.0909
0.0795	0.06967	0.07281	0.778
0.1055	0.6886	0.1112	0.09466
0.7467	0.09066	0.0657	0.09695
0.1065	0.1326	0.07972	0.6812
0.0823	0.091	0.07793	0.7488
0.09108	0.7206	0.06612	0.1222
0.7981	0.07726	0.05037	0.07426

MOTIF TTCGCAGC

0.08376	0.0727	0.06038	0.7832
0.07992	0.05642	0.07961	0.784
0.109	0.7022	0.08821	0.1005
0.09636	0.1108	0.6389	0.1539
0.08435	0.7857	0.06394	0.06604
0.7509	0.06961	0.08324	0.09624
0.07787	0.08804	0.7788	0.0553
0.06024	0.7869	0.07888	0.07393

MOTIF TCACTCCC

0.07048	0.09326	0.05669	0.7796
0.06277	0.7923	0.0754	0.06956
0.7557	0.0784	0.06914	0.09673
0.1151	0.752	0.05284	0.08013
0.103	0.07791	0.05704	0.7621
0.08809	0.7027	0.08603	0.1232
0.1673	0.626	0.08404	0.1227
0.1107	0.7151	0.08236	0.0919

MOTIF TGGGCACA

0.142	0.08354	0.0536	0.7209
0.09381	0.1607	0.568	0.1775
0.1344	0.07315	0.7561	0.03638
0.09992	0.1048	0.6239	0.1714
0.1542	0.7091	0.06382	0.07285

0.8002	0.03493	0.05203	0.1128
0.1499	0.6049	0.1185	0.1266
0.6988	0.07258	0.1448	0.0838

MOTIF TTAATACT

0.09135	0.1045	0.06922	0.7349
0.08951	0.05517	0.0581	0.7972
0.7407	0.07199	0.08485	0.1024
0.6439	0.1291	0.09518	0.1318
0.09634	0.06301	0.05557	0.7851
0.7711	0.06725	0.05809	0.1035
0.109	0.6467	0.1026	0.1418
0.07571	0.07346	0.04661	0.8042

MOTIF TTGGCCGA

0.05133	0.08563	0.1049	0.7581
0.04413	0.06678	0.09994	0.7891
0.05977	0.07929	0.6791	0.1818
0.1094	0.05505	0.7623	0.07324
0.1033	0.7261	0.07968	0.09094
0.08871	0.6873	0.0627	0.1613
0.0345	0.06271	0.818	0.08481
0.6967	0.07363	0.106	0.1237

MOTIF TGCTTTAT

0.0917	0.03703	0.06205	0.8092
0.11	0.07321	0.6392	0.1777
0.1629	0.6042	0.1112	0.1217
0.06516	0.08158	0.03532	0.8179
0.07036	0.06717	0.06753	0.7949
0.08526	0.07934	0.07647	0.7589
0.6474	0.08801	0.1307	0.1339
0.1208	0.08781	0.06541	0.726

MOTIF ACGTGA

0.8565	0.05781	0.04669	0.03898
0.1399	0.6579	0.08215	0.1201
0.1068	0.09332	0.6489	0.1509
0.04691	0.03263	0.05309	0.8674
0.06977	0.07608	0.8144	0.03978
0.7743	0.07384	0.07369	0.07821

MOTIF GCGTAT

0.07316	0.08128	0.7663	0.07927
0.1403	0.6683	0.08761	0.1038
0.1031	0.08179	0.6876	0.1275
0.09317	0.05585	0.07882	0.7722
0.6871	0.08906	0.1045	0.1194
0.06517	0.05116	0.05088	0.8328

MOTIF AGTAAC

0.8227	0.05032	0.05699	0.07
0.09415	0.05322	0.7971	0.05556
0.1497	0.07544	0.07231	0.7026
0.7797	0.06347	0.07077	0.08602
0.8432	0.0433	0.06087	0.0526
0.08443	0.7833	0.0714	0.06082

MOTIF TTCCGG

0.07905	0.09317	0.1005	0.7272
0.06874	0.04776	0.07359	0.8099
0.06154	0.7433	0.07769	0.1175
0.1464	0.6644	0.04193	0.1472
0.1037	0.05036	0.7316	0.1143
0.0758	0.06524	0.8017	0.05727

MOTIF CCGATT

0.06933	0.8	0.0585	0.07221
0.08463	0.7623	0.06097	0.09206
0.1562	0.08701	0.6587	0.09808
0.7778	0.05921	0.0733	0.08965
0.06208	0.04488	0.1137	0.7793
0.0558	0.05913	0.05553	0.8295

MOTIF TAAGGC

0.09759	0.1158	0.09353	0.6931
0.7607	0.08948	0.08501	0.06481
0.8258	0.0665	0.04115	0.06658
0.1314	0.06184	0.755	0.0517
0.05848	0.09749	0.7772	0.0668
0.09003	0.7912	0.05578	0.06302

MOTIF TCACAG

0.0747	0.06028	0.07173	0.7933
0.05731	0.8006	0.06702	0.07509
0.8082	0.05986	0.0542	0.07777
0.08536	0.7776	0.06722	0.06982
0.8241	0.0498	0.04626	0.07987
0.1395	0.1117	0.6454	0.1035

MOTIF GGCTTA

0.09409	0.03462	0.7573	0.114
0.06963	0.1075	0.7612	0.06167
0.08779	0.7384	0.0818	0.09199
0.06511	0.06248	0.04985	0.8226
0.07388	0.06769	0.1094	0.749
0.7481	0.08742	0.08744	0.077

MOTIF TTGGAA

0.05582	0.05168	0.06919	0.8233

0.08486	0.03685	0.0711	0.8072
0.1042	0.01545	0.7914	0.0889
0.0921	0.01521	0.8363	0.05636
0.8296	0.05576	0.05988	0.05479
0.8176	0.04718	0.06151	0.07376

MOTIF GCGTTC

0.06044	0.087	0.7718	0.08071
0.09247	0.6989	0.09216	0.1165
0.07533	0.127	0.6808	0.1169
0.06537	0.06759	0.06113	0.8059
0.04731	0.0924	0.04525	0.815
0.07723	0.7373	0.07912	0.1063

MOTIF TCTGGG

0.08316	0.08329	0.05341	0.7801
0.06835	0.7437	0.07204	0.1159
0.06628	0.04222	0.05344	0.8381
0.06716	0.06259	0.7573	0.113
0.08514	0.04202	0.787	0.08579
0.08943	0.08818	0.7569	0.06545

MOTIF TTACCG

0.05871	0.06462	0.06449	0.8122
0.08451	0.07747	0.09611	0.7419
0.7431	0.05536	0.1228	0.07872
0.0722	0.7682	0.05434	0.1053
0.1169	0.7113	0.0641	0.1077
0.1005	0.08009	0.7149	0.1046

MOTIF GTGCGA

0.04572	0.08352	0.8094	0.06139
0.07196	0.04852	0.08529	0.7942
0.03725	0.06876	0.8082	0.08574
0.131	0.6246	0.08522	0.1592
0.1025	0.1144	0.671	0.1121
0.7928	0.05058	0.07214	0.0845

MOTIF ACTCAC

0.7419	0.09342	0.06689	0.09776
0.1086	0.7274	0.07234	0.09171
0.09222	0.0492	0.05794	0.8006
0.05804	0.7945	0.05167	0.09576
0.7993	0.07526	0.04419	0.08126
0.08027	0.7557	0.0922	0.07184

MOTIF CGGAGAATCA

0.09549	0.7157	0.05695	0.1318
0.09304	0.05543	0.7817	0.06982
0.1446	0.08589	0.6902	0.07929

0.808	0.05051	0.07665	0.06489
0.07904	0.0628	0.822	0.03615
0.8476	0.05267	0.05661	0.04311
0.833	0.04741	0.05395	0.06561
0.08501	0.0663	0.1529	0.6958
0.07966	0.7477	0.1078	0.06486
0.8463	0.05017	0.04108	0.06247

MOTIF TTCTACTCAC

0.06266	0.1115	0.04906	0.7768
0.06275	0.09878	0.1149	0.7236
0.06752	0.7599	0.07349	0.0991
0.08813	0.07387	0.0472	0.7908
0.6678	0.1267	0.07674	0.1288
0.07741	0.735	0.08118	0.1064
0.08589	0.08132	0.06218	0.7706
0.106	0.7352	0.05877	0.1001
0.7571	0.08917	0.05289	0.1008
0.1275	0.7006	0.07624	0.09557

MOTIF CCGTAGTG

0.0776	0.7619	0.09438	0.06615
0.07634	0.7481	0.09009	0.08543
0.09087	0.07188	0.7594	0.07787
0.09987	0.09154	0.09959	0.709
0.739	0.07879	0.0939	0.08826
0.08675	0.1079	0.7329	0.07245
0.1332	0.1162	0.03235	0.7182
0.0725	0.08127	0.7744	0.07183

MOTIF TAGTAGGA

0.1122	0.0684	0.1018	0.7176
0.8121	0.04551	0.06111	0.08127
0.05031	0.05955	0.7825	0.1077
0.1272	0.07705	0.09631	0.6995
0.7913	0.04501	0.06936	0.0943
0.09571	0.05041	0.756	0.09784
0.1343	0.09853	0.678	0.08917
0.8069	0.0607	0.07474	0.05771

MOTIF ATTCTCCG

0.6295	0.1635	0.08792	0.1191
0.0519	0.08511	0.06008	0.8029
0.07238	0.07527	0.05569	0.7967
0.03537	0.7921	0.09226	0.0803
0.09962	0.08193	0.05074	0.7677
0.08915	0.7234	0.06747	0.12
0.09505	0.7351	0.06099	0.1088
0.1407	0.1012	0.6253	0.1329

MOTIF GCTTCCCG

0.07885	0.07016	0.7767	0.07428
0.07212	0.7711	0.07493	0.08184
0.06445	0.08375	0.0744	0.7774
0.0793	0.12	0.09163	0.709
0.1038	0.7558	0.09028	0.0501
0.08781	0.7211	0.1058	0.08529
0.09669	0.7379	0.0734	0.092
0.08426	0.1518	0.6453	0.1186

MOTIF ACGTTCTA

0.7939	0.05458	0.04536	0.1061
0.1281	0.6892	0.07313	0.1096
0.1289	0.1057	0.6439	0.1215
0.0721	0.07179	0.06449	0.7916
0.07974	0.0448	0.05648	0.819
0.0393	0.7509	0.09072	0.119
0.07355	0.0725	0.04757	0.8064
0.7995	0.06498	0.05352	0.082

MOTIF TACGCTGC

0.06554	0.0972	0.1103	0.7269
0.6064	0.1637	0.1108	0.1191
0.07268	0.7322	0.06588	0.1292
0.07699	0.08647	0.7297	0.1068
0.09168	0.7198	0.1078	0.08068
0.07031	0.06613	0.06094	0.8026
0.05796	0.06453	0.8002	0.07731
0.0723	0.8167	0.03973	0.07124

MOTIF CTGAAACC

0.1017	0.7117	0.07762	0.109
0.08853	0.05255	0.06095	0.798
0.1563	0.08954	0.6297	0.1244
0.7523	0.1036	0.07462	0.06955
0.7322	0.06764	0.07083	0.1293
0.7586	0.05717	0.07808	0.1062
0.107	0.7173	0.08361	0.09207
0.1092	0.6761	0.06386	0.1509

MOTIF GTAAGA

0.09723	0.1098	0.7149	0.07803
0.09772	0.06169	0.0836	0.757
0.8224	0.04871	0.05716	0.07174
0.8114	0.05002	0.08106	0.05749
0.1025	0.06709	0.7574	0.07297
0.8434	0.03844	0.05634	0.06181

MOTIF TACGGG

0.08801	0.06072	0.06135	0.7899

0.8071	0.05244	0.06558	0.07487
0.1113	0.6989	0.1506	0.03913
0.1193	0.09642	0.6859	0.09839
0.07951	0.05924	0.7969	0.06436
0.05298	0.07929	0.7741	0.09362

MOTIF TACCGG

0.05579	0.1338	0.06478	0.7457
0.8128	0.05019	0.0538	0.0832
0.05231	0.8037	0.06815	0.07587
0.121	0.6926	0.08307	0.1033
0.08337	0.05192	0.7734	0.09134
0.07464	0.08455	0.7455	0.0953

MOTIF CGCAAT

0.1485	0.6095	0.09106	0.151
0.08324	0.06558	0.7202	0.131
0.07851	0.7491	0.07077	0.1016
0.8175	0.08016	0.0375	0.06483
0.7832	0.061	0.07415	0.08169
0.06601	0.06154	0.09758	0.7749

MOTIF CTAAGA

0.09548	0.7378	0.0891	0.07763
0.05572	0.07152	0.05962	0.8131
0.828	0.05035	0.05248	0.06912
0.833	0.05122	0.06878	0.04703
0.1381	0.07062	0.7135	0.07777
0.8355	0.04309	0.05218	0.06925

MOTIF TACTCG

0.07105	0.05412	0.04635	0.8285
0.7882	0.08017	0.06616	0.06547
0.04399	0.8276	0.04659	0.08181
0.05352	0.04825	0.05363	0.8446
0.1176	0.6494	0.09366	0.1393
0.0944	0.1376	0.6373	0.1308

MOTIF TTCGTA

0.05518	0.06375	0.04628	0.8348
0.01967	0.04499	0.05243	0.8829
0.1352	0.6656	0.09215	0.107
0.08731	0.1153	0.6322	0.1652
0.02309	0.04875	0.08239	0.8458
0.803	0.07505	0.04433	0.07764

MOTIF GTAGGA

0.05536	0.07	0.7891	0.08557
0.1375	0.07947	0.09956	0.6835
0.8273	0.03646	0.06189	0.0743

0.09125	0.05192	0.7957	0.06109
0.08314	0.1086	0.7228	0.08547
0.8198	0.04225	0.08369	0.05429

MOTIF GGGGGA

0.1138	0.05923	0.7727	0.05424
0.112	0.0504	0.7695	0.06808
0.12	0.05421	0.7531	0.07272
0.09776	0.04764	0.7965	0.05814
0.07809	0.0783	0.7844	0.05917
0.8047	0.06094	0.08168	0.0527

MOTIF TACAGG

0.06064	0.06233	0.04265	0.8344
0.789	0.05779	0.08262	0.07057
0.09909	0.7398	0.09182	0.06928
0.7604	0.08395	0.07076	0.08488
0.1244	0.04777	0.7315	0.0963
0.07149	0.069	0.7548	0.1047

MOTIF GTATCG

0.05831	0.08723	0.7608	0.09365
0.05922	0.07458	0.04823	0.818
0.7565	0.07487	0.08229	0.08633
0.0687	0.09568	0.09188	0.7437
0.05567	0.7451	0.08573	0.1135
0.1371	0.0805	0.6705	0.1119

MOTIF CGTGAC

0.1193	0.674	0.119	0.08768
0.1323	0.09669	0.6663	0.1047
0.0962	0.05587	0.07507	0.7729
0.07003	0.09545	0.7435	0.09097
0.6824	0.1071	0.1119	0.09866
0.08831	0.7402	0.08579	0.08569

MOTIF CGATAG

0.1518	0.6579	0.1072	0.08314
0.08404	0.08811	0.7894	0.03842
0.7265	0.0874	0.1239	0.06226
0.1019	0.07259	0.08964	0.7358
0.8638	0.02612	0.06007	0.05006
0.09509	0.09403	0.7555	0.0554

MOTIF GAATCA

0.1001	0.07888	0.7785	0.04253
0.8243	0.05423	0.04758	0.07385
0.7896	0.05587	0.05882	0.09573
0.06937	0.06174	0.1264	0.7425
0.08725	0.7605	0.08457	0.06766

0.8509	0.03723	0.03301	0.07884

MOTIF TCTCCG

0.05786	0.05195	0.06598	0.8242
0.04559	0.8076	0.07198	0.07479
0.09086	0.06596	0.05505	0.7881
0.07858	0.7933	0.0548	0.07333
0.06739	0.8037	0.0558	0.07316
0.1194	0.1004	0.6687	0.1114

MOTIF GTATTC

0.109	0.09315	0.6963	0.1015
0.03049	0.07447	0.04345	0.8516
0.7701	0.07441	0.05965	0.0958
0.05201	0.1111	0.04247	0.7944
0.02792	0.05529	0.05563	0.8611
0.07539	0.7388	0.0617	0.1241

MOTIF AGCGGC

0.7866	0.07777	0.07468	0.06092
0.06016	0.06718	0.8088	0.06386
0.09236	0.7218	0.1066	0.07921
0.1824	0.06066	0.6368	0.1202
0.05549	0.03963	0.8413	0.06356
0.08246	0.7912	0.07198	0.05434

MOTIF TGCGGTGGGA

0.05971	0.06416	0.07131	0.8048
0.03713	0.0662	0.8084	0.08824
0.04459	0.695	0.1223	0.138
0.05732	0.07248	0.812	0.05817
0.06838	0.06009	0.7937	0.07787
0.064	0.08554	0.0657	0.7848
0.04314	0.06567	0.7887	0.1025
0.04963	0.05764	0.7451	0.1477
0.07017	0.07379	0.7905	0.06555
0.6693	0.1488	0.08319	0.09864

MOTIF CCGCGCGGTT

0.03271	0.7902	0.09898	0.07806
0.04342	0.773	0.1257	0.05793
0.05916	0.06497	0.8179	0.05798
0.06476	0.7389	0.0572	0.1392
0.1038	0.08966	0.7292	0.07731
0.06038	0.7386	0.1204	0.08054
0.04064	0.1037	0.7747	0.08098
0.0727	0.1224	0.7619	0.04299
0.088	0.1029	0.1168	0.6923
0.0936	0.1881	0.1125	0.6058

MOTIF TCTGTGCGTT

0.09546	0.07484	0.08021	0.7495
0.08753	0.693	0.07789	0.1416
0.03299	0.07093	0.09969	0.7964
0.06485	0.09807	0.753	0.0841
0.07294	0.07228	0.07872	0.7761
0.05662	0.1555	0.6799	0.108
0.08294	0.7033	0.06902	0.1447
0.1069	0.08971	0.6631	0.1402
0.07168	0.06474	0.09965	0.7639
0.05739	0.06655	0.1833	0.6928

MOTIF ACTGTACCTA

0.7736	0.09203	0.0851	0.04931
0.1318	0.6896	0.08532	0.09323
0.08817	0.06454	0.04155	0.8057
0.06462	0.05577	0.8128	0.06682
0.09158	0.09387	0.05769	0.7569
0.7247	0.09466	0.09048	0.09013
0.08961	0.7535	0.06511	0.09176
0.1971	0.6261	0.1181	0.05865
0.1032	0.1119	0.09113	0.6937
0.7253	0.0961	0.1308	0.04781

MOTIF TACTGTAC

0.08479	0.1168	0.07689	0.7215
0.7537	0.07429	0.09507	0.07691
0.1306	0.7469	0.06424	0.05826
0.09444	0.05769	0.04534	0.8025
0.06648	0.05049	0.7997	0.08328
0.04854	0.1077	0.06174	0.7821
0.7389	0.1002	0.07202	0.08891
0.07425	0.7447	0.0843	0.09672

MOTIF ATCCCACC

0.701	0.0964	0.09736	0.1052
0.0778	0.1197	0.1038	0.6987
0.0683	0.7765	0.09601	0.05923
0.1054	0.7662	0.07388	0.05446
0.09359	0.7578	0.1092	0.03939
0.7394	0.07845	0.1008	0.08132
0.08467	0.7677	0.06986	0.07774
0.07829	0.7767	0.09768	0.04729

MOTIF GGACGGAT

0.05744	0.06404	0.8263	0.05224
0.07145	0.1193	0.711	0.09831
0.6786	0.1544	0.06826	0.09882
0.06421	0.7392	0.09126	0.1054
0.1128	0.06637	0.7601	0.06074

0.07217	0.09407	0.7606	0.07314
0.7691	0.06324	0.09178	0.07593
0.08815	0.08678	0.0931	0.732

MOTIF GGCTGTAC

0.07525	0.1187	0.6989	0.1072
0.1029	0.08471	0.7272	0.08512
0.06631	0.7897	0.07869	0.06527
0.05508	0.1429	0.05655	0.7455
0.0335	0.0661	0.8476	0.05282
0.09277	0.1684	0.1114	0.6274
0.7045	0.1162	0.09566	0.08365
0.08482	0.7815	0.05924	0.07445

MOTIF GGCCGCAT

0.04463	0.05899	0.8143	0.0821
0.06599	0.07509	0.7806	0.07835
0.07194	0.8285	0.03688	0.06272
0.09999	0.6982	0.1112	0.0906
0.06943	0.08373	0.7677	0.07909
0.06992	0.7521	0.1145	0.06346
0.7602	0.08477	0.09297	0.06203
0.1184	0.1404	0.09738	0.6438

MOTIF AGCCGGTT

0.686	0.09005	0.1218	0.1021
0.02277	0.06575	0.7734	0.1381
0.06084	0.7438	0.08289	0.1125
0.08706	0.7205	0.09496	0.09745
0.07713	0.05318	0.7531	0.1165
0.07412	0.09087	0.772	0.06298
0.08918	0.04969	0.08305	0.7781
0.03774	0.1061	0.2114	0.6448

MOTIF GGTGCGTG

0.08201	0.08077	0.6781	0.1591
0.08706	0.09424	0.7402	0.07851
0.05858	0.0636	0.07968	0.7981
0.0807	0.1094	0.7298	0.08009
0.07747	0.6959	0.1002	0.1264
0.0702	0.1036	0.714	0.1122
0.06117	0.09302	0.1019	0.7439
0.08588	0.08441	0.7596	0.07011

MOTIF CACAGACC

0.08522	0.7719	0.07536	0.06757
0.7537	0.09819	0.1014	0.04667
0.08143	0.7878	0.09689	0.03392
0.7429	0.07878	0.1044	0.07388
0.08976	0.11	0.6944	0.1058

0.7576	0.0829	0.07936	0.08015
0.08517	0.7003	0.1002	0.1143
0.1241	0.7313	0.06939	0.07523

MOTIF CGCGGAAT

0.06749	0.6894	0.09652	0.1466
0.09884	0.0803	0.7674	0.05348
0.105	0.6915	0.117	0.08642
0.09056	0.08789	0.6989	0.1227
0.07531	0.08336	0.7735	0.0678
0.6796	0.1224	0.1205	0.07762
0.7316	0.108	0.07602	0.08433
0.06865	0.1545	0.06851	0.7084

MOTIF ATGCGTAC

0.7415	0.08876	0.09375	0.07604
0.0529	0.07532	0.06948	0.8023
0.06711	0.1119	0.7715	0.04954
0.06556	0.6985	0.1178	0.1182
0.2103	0.08786	0.6431	0.05872
0.06731	0.05073	0.1129	0.7691
0.6999	0.09799	0.1371	0.06494
0.0658	0.7645	0.08218	0.08752

MOTIF GATCCG

0.06584	0.06198	0.7983	0.0739
0.824	0.06091	0.0763	0.03877
0.1756	0.09138	0.0746	0.6584
0.04297	0.8399	0.07815	0.03897
0.05134	0.8189	0.06169	0.06808
0.09317	0.0513	0.8064	0.04918

MOTIF CCGCAT

0.06853	0.8236	0.05617	0.05171
0.08801	0.7511	0.08591	0.07494
0.09114	0.06965	0.7805	0.0587
0.05793	0.7941	0.09994	0.04803
0.8064	0.08405	0.06137	0.04818
0.09499	0.1102	0.05335	0.7414

MOTIF ACGCAC

0.8125	0.09465	0.05228	0.04057
0.08321	0.7634	0.07606	0.07728
0.1048	0.07295	0.7486	0.07365
0.06229	0.7955	0.07383	0.06835
0.8402	0.07126	0.04095	0.04761
0.05632	0.8268	0.06001	0.05691

MOTIF CTGTAC

0.1209	0.7538	0.06229	0.06293

0.06875	0.06972	0.05192	0.8096
0.03829	0.03665	0.8796	0.04551
0.06428	0.07453	0.07897	0.7822
0.7136	0.1182	0.08147	0.08675
0.05711	0.8261	0.05352	0.06332

MOTIF TCTCTG

0.04355	0.05718	0.07136	0.8279
0.04643	0.7963	0.07963	0.07761
0.08353	0.07094	0.06157	0.784
0.06402	0.7285	0.1181	0.08946
0.05101	0.04553	0.0714	0.8321
0.04146	0.1082	0.8008	0.04958

MOTIF GCGTAC

0.1011	0.09661	0.7251	0.07715
0.05317	0.7647	0.09629	0.0858
0.07213	0.05766	0.8116	0.05856
0.06693	0.06895	0.09873	0.7654
0.7159	0.09671	0.1202	0.06718
0.06522	0.8143	0.06737	0.05306

MOTIF ATTCCG

0.8045	0.07176	0.05635	0.06743
0.09306	0.06846	0.09244	0.746
0.05766	0.09422	0.1142	0.734
0.06071	0.78	0.06747	0.09181
0.07227	0.7579	0.08412	0.08568
0.07991	0.09883	0.7413	0.07991

MOTIF TAACCG

0.08451	0.0606	0.2402	0.6147
0.7621	0.1012	0.08959	0.04714
0.6984	0.1375	0.06725	0.09681
0.04637	0.8515	0.05362	0.0485
0.09062	0.7649	0.05735	0.08717
0.06191	0.06011	0.8414	0.03657

MOTIF GAACCG

0.05489	0.04054	0.8465	0.05806
0.7675	0.1186	0.06709	0.04684
0.7566	0.07697	0.06449	0.1019
0.04853	0.8818	0.03927	0.03043
0.08261	0.8013	0.05632	0.05977
0.05969	0.04392	0.8422	0.05418

MOTIF GACCCG

0.06217	0.06302	0.769	0.1058
0.8034	0.07472	0.07721	0.04465
0.1823	0.6133	0.07664	0.1277

0.05995	0.837	0.06272	0.04033
0.07631	0.7944	0.06231	0.06695
0.07181	0.05855	0.8085	0.06115

MOTIF GGTAAT

0.04979	0.07789	0.7948	0.07756
0.0728	0.07975	0.8094	0.03803
0.09204	0.0818	0.1051	0.721
0.698	0.1057	0.113	0.08326
0.746	0.09964	0.06259	0.09173
0.09638	0.06611	0.0637	0.7738

MOTIF CCGAAC

0.06931	0.8426	0.05036	0.03772
0.07651	0.8235	0.03551	0.06448
0.08967	0.08722	0.7895	0.03362
0.7235	0.09434	0.1145	0.06767
0.7886	0.08224	0.07468	0.05452
0.08358	0.7806	0.08315	0.05268

MOTIF TGGCCG

0.0598	0.03479	0.0666	0.8388
0.04329	0.06553	0.8272	0.064
0.05536	0.06419	0.7862	0.09422
0.06914	0.7698	0.106	0.05504
0.0747	0.73	0.08017	0.1151
0.0319	0.07366	0.8469	0.04757

MOTIF ATACTG

0.7491	0.0771	0.1115	0.06236
0.08386	0.1044	0.06746	0.7442
0.728	0.07095	0.1177	0.08333
0.07272	0.7913	0.07044	0.06552
0.09479	0.05928	0.06673	0.7792
0.06382	0.06331	0.8143	0.05855

MOTIF ATGCCG

0.7434	0.07772	0.1011	0.07776
0.06902	0.1146	0.06339	0.753
0.04353	0.06877	0.8116	0.07607
0.03421	0.8166	0.07538	0.07381
0.07496	0.7367	0.1004	0.0879
0.08247	0.07856	0.791	0.04796

MOTIF GCGAAT

0.09171	0.09321	0.7541	0.06101
0.06156	0.6989	0.1101	0.1295
0.09981	0.1209	0.7107	0.06857
0.7237	0.08871	0.1201	0.06753
0.7804	0.05474	0.1041	0.06069

0.05927	0.08519	0.09695	0.7586

MOTIF CCGGCT

0.05885	0.8125	0.06007	0.06858
0.06521	0.7731	0.0675	0.0942
0.07974	0.06624	0.7743	0.07972
0.05299	0.08065	0.8031	0.06321
0.0955	0.7693	0.05946	0.07571
0.07175	0.1159	0.1284	0.684

MOTIF CCAGGA

0.07093	0.8127	0.07096	0.04543
0.1017	0.7731	0.08372	0.04143
0.7829	0.07498	0.05625	0.08586
0.08187	0.09629	0.7113	0.1106
0.1149	0.1266	0.6995	0.05901
0.7571	0.1042	0.06468	0.074

MOTIF GACCCCGTTC

0.05012	0.03362	0.8464	0.06985
0.8153	0.06249	0.04151	0.08074
0.027	0.876	0.03963	0.05731
0.03731	0.8384	0.08744	0.03688
0.04736	0.8519	0.05113	0.04958
0.02863	0.8871	0.03094	0.05333
0.05081	0.0675	0.8287	0.053
0.05487	0.07128	0.1915	0.6824
0.04075	0.1205	0.0711	0.7676
0.09408	0.8184	0.04512	0.04243

MOTIF TCGTCTGTCC

0.06257	0.07572	0.05456	0.8072
0.05813	0.8018	0.08046	0.05957
0.07008	0.1228	0.7454	0.06166
0.07308	0.1239	0.09228	0.7108
0.04347	0.8297	0.06278	0.064
0.05232	0.1248	0.1225	0.7004
0.07452	0.0984	0.6776	0.1494
0.09399	0.1094	0.06888	0.7277
0.08034	0.7866	0.06733	0.0657
0.1003	0.7338	0.0916	0.07424

MOTIF GAACCCGGTC

0.0583	0.06527	0.7702	0.1063
0.7048	0.0633	0.158	0.07397
0.4979	0.331	0.08126	0.08983
0.03704	0.8125	0.08725	0.06325
0.05675	0.7864	0.07948	0.07733
0.0642	0.8133	0.03705	0.08549
0.06657	0.1203	0.723	0.09017

0.08215	0.05226	0.7944	0.07117
0.07581	0.09451	0.08647	0.7432
0.04153	0.8407	0.0609	0.05685

MOTIF GTAAGTTGGC

0.1153	0.08166	0.7543	0.04875
0.08748	0.05835	0.1288	0.7254
0.7324	0.1431	0.0948	0.02967
0.7613	0.08355	0.08277	0.07238
0.06462	0.1481	0.655	0.1323
0.09774	0.04534	0.1194	0.7375
0.04198	0.0996	0.05481	0.8036
0.05247	0.05802	0.8402	0.04935
0.06686	0.06545	0.8176	0.05011
0.04918	0.7597	0.07122	0.1199

MOTIF GGCCCCGGTC

0.08436	0.07725	0.75	0.08835
0.2116	0.07444	0.628	0.08599
0.1057	0.7544	0.05901	0.08084
0.06733	0.7847	0.08784	0.06008
0.06701	0.7361	0.07397	0.1229
0.06471	0.8201	0.05323	0.06196
0.07824	0.09692	0.7493	0.07556
0.05924	0.0815	0.7986	0.06068
0.09633	0.1028	0.1181	0.6828
0.05892	0.8299	0.05318	0.05799

MOTIF CCCCGGTC

0.06774	0.8344	0.03569	0.0622
0.06187	0.7931	0.09342	0.05156
0.06127	0.7279	0.09868	0.1121
0.0769	0.7494	0.1023	0.07144
0.06799	0.0934	0.7562	0.08242
0.05331	0.09298	0.7687	0.08502
0.08218	0.1501	0.0892	0.6785
0.04982	0.8308	0.06474	0.05465

MOTIF ACGTTGGC

0.75	0.08958	0.06645	0.09393
0.1265	0.6989	0.1037	0.07097
0.05747	0.1151	0.7706	0.05678
0.07967	0.06249	0.1213	0.7366
0.03539	0.1385	0.0841	0.7421
0.05215	0.04994	0.8576	0.04028
0.06385	0.09622	0.7451	0.09482
0.0428	0.799	0.09138	0.0668

MOTIF TGGGACGC

0.1307	0.1133	0.1518	0.6042

0.09009	0.1011	0.7424	0.06637
0.06645	0.08731	0.7532	0.09306
0.08885	0.1487	0.7236	0.03886
0.7182	0.08381	0.1047	0.09331
0.1475	0.6565	0.1387	0.05732
0.09881	0.07552	0.7237	0.1019
0.115	0.7054	0.1182	0.06142

MOTIF AAGTCGGT

0.7591	0.09582	0.07815	0.06696
0.5438	0.1684	0.1531	0.1347
0.0826	0.102	0.7754	0.04001
0.07557	0.08937	0.09422	0.7408
0.03114	0.7667	0.06624	0.1359
0.06211	0.0662	0.8238	0.04786
0.07362	0.08095	0.7716	0.07381
0.09794	0.1358	0.0747	0.6916

MOTIF ATTAACGC

0.7576	0.07697	0.09084	0.07457
0.0584	0.07571	0.08021	0.7857
0.06027	0.103	0.06204	0.7747
0.7113	0.1017	0.1332	0.05372
0.7875	0.05526	0.1025	0.05466
0.03814	0.8291	0.05859	0.07416
0.08567	0.1036	0.7462	0.06453
0.09742	0.7763	0.05282	0.07345

MOTIF GACGCGCT

0.06199	0.09604	0.791	0.05096
0.7254	0.1264	0.05728	0.09093
0.05969	0.7698	0.1223	0.04824
0.09575	0.07132	0.7858	0.04715
0.09803	0.7588	0.08882	0.05438
0.06194	0.1364	0.7225	0.07911
0.09889	0.7594	0.09952	0.04224
0.09672	0.0937	0.1361	0.6735

MOTIF AGGTCTAA

0.7589	0.07692	0.08662	0.07756
0.1005	0.0751	0.7639	0.06048
0.06219	0.07405	0.8045	0.05929
0.08665	0.1055	0.1351	0.6728
0.06923	0.7811	0.06359	0.08603
0.06614	0.06609	0.1162	0.7516
0.7122	0.1294	0.07112	0.08729
0.763	0.07407	0.08973	0.07318

MOTIF AACTCGGT

0.665	0.1132	0.1337	0.0882

0.6779	0.1096	0.1076	0.1049
0.09297	0.7271	0.1363	0.04363
0.08379	0.1109	0.07624	0.7291
0.04159	0.7604	0.07798	0.1201
0.0717	0.09867	0.7693	0.06037
0.05416	0.07201	0.8234	0.05041
0.05886	0.1475	0.1223	0.6714

MOTIF ATTAGCGC

0.7011	0.1253	0.1092	0.06444
0.1092	0.09312	0.07372	0.724
0.05072	0.155	0.08828	0.706
0.6926	0.1167	0.127	0.0637
0.1592	0.06383	0.7105	0.06638
0.05789	0.7912	0.08808	0.06282
0.06117	0.1293	0.719	0.09058
0.05721	0.7981	0.07025	0.07442

MOTIF ACCCCG

0.7082	0.1127	0.07352	0.1055
0.049	0.8299	0.06009	0.06097
0.06707	0.7901	0.07954	0.06326
0.04758	0.8114	0.08301	0.05805
0.05045	0.842	0.05006	0.05745
0.04945	0.08106	0.8086	0.06085

MOTIF TCCCGA

0.08216	0.1018	0.1658	0.6502
0.06788	0.828	0.06273	0.04134
0.04857	0.8831	0.01994	0.04842
0.03971	0.8748	0.01749	0.06802
0.06316	0.07945	0.793	0.06439
0.7918	0.07555	0.06588	0.06676

MOTIF GTAACG

0.04628	0.06911	0.7841	0.1005
0.07119	0.0966	0.08045	0.7518
0.7363	0.1024	0.09594	0.0653
0.7639	0.09279	0.06451	0.07878
0.06548	0.7958	0.08486	0.05386
0.06296	0.06586	0.8114	0.0598

MOTIF TTAACG

0.05279	0.0814	0.1244	0.7414
0.07047	0.1138	0.06959	0.7462
0.7512	0.1106	0.08066	0.0575
0.8078	0.08671	0.06186	0.04359
0.04146	0.8362	0.0624	0.05998
0.07332	0.1003	0.7749	0.05142

MOTIF TAGCGC

0.07821	0.1283	0.1221	0.6714
0.6368	0.1239	0.153	0.08628
0.08039	0.07225	0.798	0.04937
0.06312	0.8093	0.08664	0.04095
0.05814	0.09968	0.7846	0.05759
0.04057	0.847	0.06288	0.04959

MOTIF TCAGCG

0.07792	0.08626	0.09115	0.7447
0.03549	0.8297	0.0834	0.05146
0.7113	0.0923	0.1305	0.06586
0.0917	0.08419	0.7526	0.07149
0.07111	0.7507	0.1208	0.05739
0.04789	0.1242	0.7483	0.07959

MOTIF GCGAAT

0.07179	0.0793	0.8006	0.0483
0.07378	0.7432	0.1018	0.08117
0.04999	0.09138	0.7835	0.07515
0.7212	0.1131	0.09628	0.06941
0.7416	0.07279	0.0888	0.0968
0.06622	0.1234	0.1593	0.6511

MOTIF GTACGG

0.06792	0.06214	0.7889	0.081
0.1226	0.1084	0.1717	0.5973
0.6944	0.1607	0.06883	0.07601
0.1034	0.7065	0.1088	0.08126
0.05183	0.1198	0.754	0.0743
0.07467	0.08234	0.7807	0.06229

MOTIF GAGACC

0.06534	0.05427	0.8277	0.05268
0.8036	0.06621	0.0677	0.06252
0.05024	0.07846	0.8219	0.04944
0.6494	0.1058	0.1578	0.08704
0.05795	0.7982	0.1082	0.03559
0.05499	0.8396	0.06093	0.04453

MOTIF CGGTCA

0.06756	0.8107	0.0768	0.04494
0.05954	0.08237	0.7636	0.09444
0.05279	0.07334	0.8261	0.04779
0.1064	0.1273	0.07992	0.6864
0.05652	0.8361	0.07314	0.03422
0.7315	0.08563	0.09953	0.08338

MOTIF GTACCG

0.05584	0.09113	0.7843	0.06872

0.08215	0.1206	0.1012	0.696
0.7442	0.1198	0.0649	0.07116
0.1011	0.7597	0.06278	0.07647
0.03983	0.8316	0.06955	0.05901
0.08356	0.1132	0.7113	0.09187

MOTIF GCGTCA

0.06033	0.07585	0.794	0.06981
0.04406	0.8155	0.07018	0.07022
0.0696	0.09195	0.7576	0.08089
0.07771	0.1133	0.06732	0.7417
0.03795	0.8179	0.08363	0.06051
0.7176	0.08923	0.1377	0.05545

MOTIF CCGACT

0.05732	0.8352	0.04204	0.06543
0.04784	0.8084	0.09284	0.05096
0.06192	0.05269	0.8336	0.05178
0.7386	0.1221	0.05709	0.08226
0.04103	0.7819	0.12	0.05708
0.09126	0.1608	0.1024	0.6455

MOTIF ACGCTT

0.7184	0.09996	0.1037	0.07798
0.03569	0.786	0.118	0.0603
0.05651	0.1783	0.7001	0.06512
0.06359	0.8143	0.06653	0.05562
0.0494	0.1142	0.03173	0.8047
0.05474	0.09422	0.09501	0.756

MOTIF TTACCC

0.06629	0.08905	0.09788	0.7468
0.07767	0.06478	0.07495	0.7826
0.66	0.1473	0.08647	0.1062
0.06834	0.7754	0.09849	0.05781
0.04129	0.8695	0.0458	0.04339
0.08692	0.7886	0.06353	0.06093

MOTIF CGGGGA

0.09894	0.7538	0.08626	0.06097
0.05205	0.05811	0.8439	0.04591
0.09849	0.05646	0.7836	0.06142
0.06453	0.08492	0.8024	0.04811
0.07392	0.05483	0.8195	0.05173
0.7045	0.07261	0.1081	0.1148

MOTIF CTGAGT

0.06427	0.8251	0.05836	0.05227
0.05915	0.1529	0.105	0.6829
0.08588	0.09087	0.7622	0.06106

0.7436	0.07249	0.08598	0.09791
0.02319	0.07122	0.8569	0.04873
0.09705	0.09775	0.1258	0.6794

MOTIF ACTCGG

0.6346	0.1503	0.1381	0.07702
0.05731	0.8024	0.0982	0.04215
0.05516	0.06712	0.06445	0.8133
0.03777	0.8098	0.05773	0.09476
0.07592	0.08321	0.7921	0.04877
0.0535	0.05216	0.8481	0.04624

MOTIF AACCGTGAGC

0.7432	0.09079	0.09866	0.06736
0.7472	0.08191	0.0992	0.07167
0.08939	0.7541	0.08586	0.07065
0.1189	0.6935	0.09318	0.09445
0.1221	0.08439	0.6726	0.121
0.1072	0.07116	0.06793	0.7537
0.08296	0.0935	0.7259	0.09764
0.7352	0.09017	0.09476	0.07991
0.1005	0.08384	0.7351	0.08051
0.09466	0.7386	0.07482	0.09191

MOTIF TACGTACAGC

0.08724	0.1114	0.08277	0.7185
0.7229	0.07298	0.09495	0.1091
0.09808	0.6823	0.09774	0.1219
0.1406	0.09221	0.6717	0.09548
0.08442	0.07963	0.0811	0.7549
0.7215	0.0802	0.1104	0.08795
0.08771	0.7343	0.08746	0.09053
0.7994	0.05339	0.06981	0.07744
0.1146	0.08097	0.7103	0.0941
0.0974	0.7105	0.0891	0.103

MOTIF GCGTTA

0.04821	0.05046	0.8493	0.05207
0.04886	0.8141	0.0654	0.0716
0.07527	0.05376	0.7876	0.08335
0.0343	0.03976	0.04125	0.8847
0.05158	0.04942	0.06106	0.8379
0.8448	0.04988	0.05304	0.05225

MOTIF TACGTC

0.03389	0.05005	0.01333	0.9027
0.8436	0.0585	0.04362	0.05423
0.03849	0.8643	0.04147	0.05576
0.0765	0.06047	0.8028	0.06026
0.04913	0.04338	0.05261	0.8549

0.02043	0.8612	0.0668	0.05157

MOTIF TATTACGC

0.06663	0.06156	0.04607	0.8257
0.7902	0.05512	0.05868	0.09602
0.05109	0.05873	0.05755	0.8326
0.07518	0.06812	0.06329	0.7934
0.809	0.05629	0.06704	0.0677
0.09729	0.7001	0.08224	0.1204
0.1258	0.07771	0.6967	0.09974
0.08185	0.7605	0.07241	0.08527

MOTIF CCGTTA

0.04339	0.8729	0.03674	0.04701
0.0553	0.8309	0.05161	0.06221
0.0657	0.05223	0.8103	0.07172
0.03761	0.04977	0.04629	0.8663
0.04094	0.05017	0.06314	0.8457
0.792	0.05633	0.07778	0.07391

MOTIF GCGTTTAC

0.08346	0.08291	0.7381	0.09556
0.08984	0.7052	0.09969	0.1052
0.1099	0.08752	0.687	0.1156
0.05783	0.05104	0.07403	0.8171
0.06025	0.05879	0.08555	0.7954
0.05856	0.06685	0.06326	0.8113
0.7803	0.07011	0.07327	0.07627
0.1076	0.7257	0.08246	0.08415

MOTIF ATAGCG

0.8701	0.04175	0.04278	0.04538
0.0499	0.05623	0.0465	0.8474
0.8668	0.03956	0.05764	0.03599
0.04385	0.04661	0.8694	0.04019
0.08096	0.7916	0.05219	0.07529
0.07698	0.055	0.7983	0.06974

MOTIF AATGCG

0.8708	0.05088	0.03576	0.04258
0.8456	0.05437	0.05158	0.04844
0.06786	0.05563	0.04997	0.8265
0.05513	0.04646	0.8637	0.03468
0.03599	0.8208	0.06503	0.0782
0.08458	0.04985	0.7842	0.08139

MOTIF TAAGCG

0.06304	0.05185	0.05183	0.8333
0.8437	0.04541	0.05378	0.05714
0.8729	0.04589	0.04343	0.03783

0.07382	0.06188	0.8187	0.04561
0.05124	0.8297	0.06551	0.05359
0.06417	0.04484	0.8213	0.06967

MOTIF CGATAC

0.06011	0.8197	0.04616	0.07405
0.06885	0.08548	0.767	0.07868
0.8434	0.05107	0.05582	0.0497
0.0589	0.04618	0.03393	0.861
0.8649	0.04574	0.04514	0.04425
0.04299	0.873	0.03371	0.05027

MOTIF CCGTGA

0.04914	0.8651	0.0531	0.03261
0.05508	0.8273	0.0634	0.0542
0.0548	0.0374	0.8597	0.04814
0.04118	0.04233	0.03774	0.8787
0.03941	0.04844	0.8688	0.04333
0.7994	0.09211	0.0558	0.05268

MOTIF CGGTTA

0.06003	0.8233	0.05595	0.0607
0.05469	0.0448	0.8402	0.06027
0.03646	0.03485	0.8696	0.05909
0.04658	0.06033	0.04573	0.8474
0.04525	0.04807	0.06232	0.8444
0.8014	0.06987	0.05248	0.07629

MOTIF CGCAAT

0.05944	0.8177	0.03798	0.08487
0.0812	0.03511	0.8449	0.03883
0.04893	0.8398	0.04935	0.06192
0.8125	0.07446	0.05086	0.06217
0.8448	0.05023	0.05266	0.0523
0.05148	0.05391	0.05412	0.8405

MOTIF TAACCGTG

0.1003	0.08369	0.09077	0.7253
0.77	0.08477	0.08091	0.06433
0.7937	0.06647	0.06681	0.07301
0.08509	0.7889	0.0584	0.06756
0.09689	0.745	0.06361	0.09454
0.1091	0.07065	0.7235	0.09676
0.07047	0.04793	0.04887	0.8327
0.0765	0.06513	0.7756	0.08277

MOTIF CTGTACGA

0.08859	0.7683	0.07318	0.06997
0.0634	0.0508	0.05333	0.8325
0.07503	0.07097	0.7863	0.06766

0.06739	0.09225	0.06626	0.7741
0.7833	0.08444	0.05161	0.08069
0.07452	0.7563	0.08046	0.08874
0.07734	0.09655	0.7254	0.1007
0.7777	0.06947	0.07053	0.08235

MOTIF TTAGCG

0.05255	0.04663	0.04795	0.8529
0.03981	0.04091	0.04838	0.8709
0.883	0.03461	0.03871	0.0437
0.05183	0.05168	0.8575	0.03896
0.08542	0.7663	0.08257	0.06568
0.06915	0.05795	0.796	0.07686

MOTIF TAGTACGG

0.069	0.07501	0.07538	0.7806
0.7694	0.05466	0.09379	0.08215
0.06508	0.06581	0.7909	0.07823
0.08542	0.08371	0.05824	0.7726
0.7777	0.0733	0.06812	0.0809
0.08979	0.7083	0.08094	0.1209
0.1146	0.06315	0.7424	0.07984
0.06589	0.05998	0.7932	0.08089

MOTIF TACGGTAT

0.07969	0.07037	0.05215	0.7978
0.7969	0.07723	0.05504	0.07081
0.1016	0.6761	0.09613	0.1262
0.1139	0.06583	0.6976	0.1226
0.08025	0.07511	0.7552	0.08943
0.04981	0.05764	0.05431	0.8382
0.7592	0.05198	0.08976	0.09907
0.06092	0.06269	0.05904	0.8173

MOTIF GAACCGTG

0.08962	0.07468	0.7698	0.06587
0.7634	0.08265	0.08747	0.06646
0.7865	0.05454	0.08494	0.07403
0.07145	0.812	0.06313	0.05343
0.09994	0.7453	0.07245	0.08236
0.09468	0.07388	0.751	0.08041
0.08441	0.0546	0.05631	0.8047
0.06662	0.06743	0.7865	0.07942

MOTIF CTATACGC

0.08012	0.7466	0.0708	0.1025
0.0576	0.06803	0.04708	0.8273
0.7106	0.0982	0.1019	0.08928
0.1037	0.08525	0.06633	0.7447
0.8187	0.06509	0.05858	0.05762

0.08992	0.7347	0.07266	0.1027
0.1009	0.08822	0.6933	0.1176
0.1089	0.7287	0.06279	0.09959

MOTIF GGGTAGGACC

0.08004	0.06297	0.7858	0.07115
0.07931	0.08919	0.7499	0.08163
0.07853	0.07237	0.7641	0.08495
0.1102	0.1217	0.09674	0.6715
0.7502	0.09679	0.06462	0.08836
0.07037	0.06467	0.7969	0.06809
0.0649	0.08869	0.7596	0.08677
0.6275	0.1166	0.1427	0.1132
0.05896	0.7259	0.1322	0.0829
0.04919	0.8436	0.0639	0.04334

MOTIF GCATTGTACG

0.0497	0.06636	0.7763	0.1077
0.1129	0.7113	0.09768	0.07816
0.7366	0.09056	0.08424	0.08859
0.04615	0.06469	0.07832	0.8108
0.08374	0.07226	0.07826	0.7657
0.0834	0.06493	0.7716	0.08011
0.07596	0.07161	0.08761	0.7648
0.7369	0.08001	0.1033	0.07977
0.0963	0.7272	0.1032	0.07332
0.06937	0.0917	0.7313	0.1076

MOTIF GCCACCGAGG

0.08506	0.07955	0.7201	0.1153
0.09879	0.6688	0.1202	0.1122
0.08409	0.7851	0.07066	0.06016
0.7005	0.06668	0.1162	0.1166
0.07933	0.6942	0.1055	0.1209
0.07928	0.7068	0.1038	0.1101
0.1438	0.08258	0.6984	0.07516
0.7586	0.09693	0.06836	0.07614
0.1161	0.06998	0.7225	0.09136
0.08961	0.1923	0.6457	0.07239

MOTIF GCGATTAGAG

0.06545	0.07077	0.7906	0.07313
0.1027	0.7205	0.1188	0.05795
0.05658	0.08491	0.7926	0.06595
0.7583	0.1131	0.0538	0.07472
0.09309	0.05785	0.06568	0.7834
0.05306	0.05543	0.1354	0.7561
0.7795	0.05599	0.09083	0.07367
0.0467	0.09247	0.8188	0.04201
0.8071	0.0529	0.06423	0.07575

0.1128	0.04982	0.7763	0.06104

MOTIF GGTCAGTCAC

0.08199	0.05274	0.7548	0.1105
0.04649	0.09984	0.7826	0.07108
0.1002	0.07531	0.0861	0.7384
0.1221	0.7145	0.1095	0.05385
0.7717	0.09539	0.07539	0.05756
0.07058	0.07789	0.7788	0.07269
0.06867	0.09018	0.1149	0.7262
0.09445	0.7405	0.08575	0.07931
0.7075	0.1054	0.08173	0.1054
0.0865	0.6972	0.1515	0.06487

MOTIF GGTAAGTCAC

0.09149	0.06008	0.7633	0.08509
0.06803	0.1127	0.7632	0.0561
0.1236	0.09255	0.08792	0.6959
0.6645	0.1728	0.1091	0.05367
0.7895	0.073	0.06533	0.07213
0.05115	0.0788	0.799	0.07107
0.1128	0.09051	0.1065	0.6902
0.1063	0.7395	0.05996	0.09421
0.7441	0.09334	0.07676	0.08584
0.06153	0.6427	0.1902	0.1055

MOTIF AGGACGTA

0.7536	0.09797	0.07826	0.07016
0.07276	0.06597	0.7874	0.07391
0.07962	0.08061	0.7686	0.07118
0.8152	0.06146	0.06149	0.06189
0.1302	0.7108	0.08077	0.07826
0.08986	0.09486	0.706	0.1092
0.1391	0.07548	0.07876	0.7066
0.721	0.07914	0.1152	0.08464

MOTIF CTTGCGAC

0.05369	0.8379	0.06605	0.04237
0.1098	0.07672	0.06993	0.7435
0.06113	0.04602	0.07259	0.8203
0.06632	0.0782	0.7543	0.1011
0.1304	0.6766	0.08294	0.11
0.07879	0.08516	0.722	0.114
0.7204	0.08609	0.08802	0.1055
0.08251	0.797	0.05742	0.06304

MOTIF CATAACGA

0.09159	0.7395	0.08589	0.08304
0.7622	0.09228	0.05667	0.08888
0.1111	0.09737	0.08609	0.7055

0.7681	0.0753	0.09838	0.05827
0.7906	0.07689	0.05896	0.07351
0.1126	0.6281	0.128	0.1313
0.08479	0.09558	0.7602	0.05949
0.8219	0.0534	0.05847	0.06622

MOTIF GCCCAATG

0.1057	0.09283	0.6993	0.1021
0.07112	0.7761	0.06861	0.0842
0.1094	0.7536	0.05453	0.08242
0.09382	0.7595	0.06365	0.08301
0.7833	0.09613	0.06883	0.05177
0.7461	0.0763	0.09123	0.08641
0.1004	0.05835	0.1063	0.735
0.07015	0.08579	0.7546	0.08951

MOTIF CAGTACCC

0.08752	0.7481	0.05686	0.1075
0.7871	0.08539	0.06311	0.06442
0.0826	0.1215	0.7544	0.04152
0.09369	0.1178	0.09205	0.6965
0.6965	0.08007	0.1018	0.1217
0.06287	0.7591	0.09765	0.08039
0.1371	0.7176	0.06448	0.0808
0.1394	0.7088	0.05924	0.09246

MOTIF TCCCTTCT

0.05504	0.0811	0.07184	0.792
0.07156	0.7483	0.07817	0.1019
0.0911	0.6869	0.08345	0.1386
0.08607	0.7522	0.04991	0.1118
0.08945	0.07296	0.06655	0.771
0.06389	0.06721	0.09314	0.7758
0.0764	0.7734	0.06376	0.0865
0.08345	0.07008	0.09427	0.7522

MOTIF CTCGGTAT

0.09567	0.707	0.09072	0.1066
0.08197	0.07732	0.06929	0.7714
0.07217	0.7407	0.07283	0.1143
0.1183	0.1084	0.6817	0.0916
0.1237	0.05459	0.6891	0.1326
0.09799	0.1158	0.05989	0.7264
0.6888	0.08261	0.1388	0.0898
0.06606	0.1081	0.09312	0.7327

MOTIF TTGTACGC

0.06175	0.08429	0.06527	0.7887
0.07652	0.04667	0.1116	0.7652
0.06838	0.1012	0.7583	0.07213

0.08372	0.0718	0.07259	0.7719
0.7663	0.05848	0.09869	0.07651
0.1485	0.6535	0.1367	0.06126
0.05208	0.0945	0.7587	0.09473
0.09408	0.7093	0.07346	0.1232

MOTIF ACGTTCAA

0.7654	0.07476	0.07897	0.08087
0.07709	0.7295	0.09738	0.09603
0.09854	0.1224	0.701	0.07811
0.07656	0.09746	0.06654	0.7594
0.1071	0.1208	0.09158	0.6805
0.07105	0.7877	0.07524	0.06597
0.7568	0.08798	0.06005	0.09515
0.7499	0.08877	0.08958	0.07178

MOTIF GCCCACTT

0.09844	0.09526	0.6931	0.1132
0.06981	0.7883	0.04892	0.09297
0.05746	0.7801	0.07765	0.08479
0.05981	0.7333	0.1029	0.104
0.6933	0.1228	0.0792	0.1047
0.1166	0.7367	0.07659	0.07007
0.1232	0.1005	0.0991	0.6772
0.1082	0.1005	0.09738	0.6939

MOTIF GCGATTAG

0.1228	0.0671	0.698	0.1121
0.1341	0.6464	0.133	0.08656
0.07384	0.115	0.7482	0.06295
0.6642	0.1626	0.08344	0.08983
0.07864	0.07084	0.09147	0.7591
0.07677	0.06697	0.1568	0.6995
0.7494	0.07603	0.1041	0.07045
0.0729	0.07717	0.8063	0.04366

MOTIF GGGATATT

0.1087	0.08745	0.6705	0.1333
0.08072	0.05703	0.7773	0.08499
0.08058	0.0975	0.7728	0.04909
0.7141	0.1082	0.1018	0.07586
0.09181	0.1058	0.0859	0.7165
0.6713	0.1097	0.1121	0.1069
0.0875	0.07689	0.1101	0.7255
0.09888	0.07706	0.1547	0.6693

MOTIF TATCCC

0.0699	0.1115	0.0534	0.7652
0.7231	0.1117	0.0575	0.1077
0.03775	0.06836	0.0906	0.8033

0.0549	0.8212	0.05432	0.0696
0.07194	0.8297	0.03737	0.06098
0.06172	0.7513	0.08601	0.1009

MOTIF GGACGT

0.05107	0.06818	0.8189	0.06186
0.07499	0.07297	0.7856	0.06644
0.7996	0.07568	0.06118	0.06349
0.07575	0.7395	0.09959	0.08517
0.07585	0.1072	0.7316	0.08541
0.08918	0.05714	0.06931	0.7844

MOTIF AAGCCC

0.809	0.07587	0.04114	0.07396
0.8025	0.05209	0.07724	0.06815
0.06498	0.05013	0.8241	0.06084
0.0475	0.808	0.07263	0.0719
0.07349	0.7952	0.05975	0.0716
0.09748	0.7824	0.04218	0.0779

MOTIF AGTACC

0.8305	0.08184	0.02851	0.05917
0.06621	0.108	0.7729	0.05288
0.07482	0.1275	0.1039	0.6938
0.7842	0.04956	0.07431	0.09197
0.06778	0.7783	0.09316	0.06073
0.07718	0.8348	0.06582	0.02215

MOTIF TAGCGT

0.05498	0.07783	0.08158	0.7856
0.7626	0.06925	0.09121	0.07691
0.07076	0.0654	0.7681	0.09571
0.1195	0.6585	0.121	0.101
0.1346	0.06824	0.7271	0.07006
0.05051	0.06276	0.05188	0.8348

MOTIF GTTACG

0.05661	0.07688	0.7975	0.06906
0.115	0.0807	0.1004	0.7039
0.06292	0.07256	0.07817	0.7863
0.7319	0.1102	0.09784	0.06006
0.1091	0.6788	0.09502	0.1171
0.06868	0.05148	0.7866	0.09324

MOTIF TGAGCG

0.07304	0.05367	0.06168	0.8116
0.07587	0.06293	0.7861	0.07515
0.7606	0.06506	0.09464	0.07968
0.07081	0.08168	0.7987	0.04877
0.09509	0.7008	0.1115	0.09258

0.1021	0.06795	0.7471	0.08292

MOTIF GGTACG

0.06852	0.02822	0.8201	0.08319
0.1013	0.06256	0.7664	0.06971
0.09587	0.06984	0.1014	0.7329
0.7292	0.1067	0.09085	0.07323
0.1058	0.7497	0.08535	0.05913
0.06431	0.03077	0.7738	0.1311

MOTIF CCGACT

0.08319	0.7916	0.05973	0.06546
0.08696	0.7653	0.05217	0.09553
0.06502	0.1143	0.734	0.08659
0.7903	0.07214	0.06457	0.07301
0.07684	0.7651	0.09357	0.06453
0.08733	0.09713	0.06089	0.7547

MOTIF CTCGTA

0.0702	0.7924	0.06378	0.07362
0.06245	0.06073	0.05219	0.8246
0.0732	0.7464	0.08703	0.09335
0.08454	0.06916	0.745	0.1013
0.07364	0.06617	0.1052	0.755
0.7448	0.08535	0.07216	0.09768

MOTIF CGTAGG

0.0975	0.682	0.08148	0.139
0.0654	0.03095	0.8171	0.08658
0.1646	0.09445	0.1151	0.6258
0.7682	0.06248	0.1064	0.06286
0.0778	0.03382	0.8507	0.03768
0.06168	0.06678	0.8199	0.05159

MOTIF CGTAAG

0.1329	0.648	0.1124	0.1067
0.09641	0.09167	0.7853	0.02662
0.1315	0.09324	0.06635	0.7089
0.741	0.09507	0.1104	0.05348
0.8245	0.03525	0.1024	0.03784
0.05902	0.07663	0.815	0.04935

MOTIF CTTACC

0.05644	0.8555	0.04304	0.04502
0.0567	0.07374	0.07673	0.7928
0.04966	0.1181	0.09595	0.7363
0.6597	0.1294	0.1043	0.1066
0.08025	0.7656	0.06513	0.08899
0.0843	0.7511	0.06128	0.1033

MOTIF GGCCTA

0.07731	0.08652	0.7492	0.08694
0.04972	0.06843	0.7992	0.08267
0.06353	0.7865	0.1002	0.0498
0.05841	0.7961	0.06641	0.0791
0.09722	0.1096	0.06985	0.7233
0.7525	0.07413	0.07721	0.09617

MOTIF CCCAAT

0.05793	0.7976	0.05876	0.08572
0.05905	0.8066	0.04258	0.09179
0.08024	0.7725	0.06155	0.08567
0.8073	0.08461	0.04932	0.05878
0.7704	0.08888	0.05697	0.08377
0.08354	0.0702	0.06735	0.7789

MOTIF TACGCA

0.06439	0.1059	0.08768	0.742
0.8027	0.08848	0.04419	0.06468
0.1375	0.6445	0.1165	0.1015
0.03944	0.09025	0.7813	0.08904
0.08983	0.812	0.06539	0.0328
0.7776	0.08773	0.05375	0.08087

MOTIF TCCTGA

0.0577	0.07941	0.05339	0.8095
0.06714	0.7356	0.09553	0.1017
0.02224	0.8386	0.05519	0.084
0.06151	0.06408	0.03009	0.8443
0.07385	0.1299	0.6692	0.1271
0.7609	0.08361	0.08822	0.06731

MOTIF GGCATT

0.06772	0.07356	0.7622	0.09651
0.0863	0.06657	0.752	0.0951
0.07706	0.7937	0.0737	0.05558
0.7492	0.08783	0.0555	0.1075
0.06401	0.07109	0.0712	0.7937
0.05389	0.06057	0.1163	0.7692

MOTIF AACGTG

0.7855	0.0262	0.1139	0.07433
0.7265	0.09655	0.1084	0.06859
0.06761	0.7104	0.1359	0.08613
0.08063	0.1146	0.7131	0.09173
0.1078	0.0537	0.07189	0.7666
0.05809	0.0686	0.8571	0.01626

MOTIF CCCACT

0.06521	0.7897	0.06069	0.0844

0.05485	0.8212	0.05782	0.06617
0.05458	0.7769	0.09536	0.07313
0.7418	0.1042	0.06027	0.09369
0.1259	0.7258	0.05862	0.08966
0.09866	0.08681	0.08263	0.7319

MOTIF CGTATC

0.07924	0.8054	0.05798	0.05734
0.09499	0.09928	0.6958	0.11
0.08209	0.07617	0.07573	0.766
0.7509	0.06835	0.1013	0.0795
0.08166	0.09348	0.07403	0.7508
0.0793	0.8029	0.03402	0.08374

MOTIF TGTACG

0.07618	0.03343	0.09544	0.795
0.07773	0.06514	0.7988	0.05836
0.08481	0.1192	0.09354	0.7025
0.738	0.05751	0.09938	0.1051
0.1069	0.6783	0.1505	0.06433
0.02425	0.07164	0.8103	0.09377

MOTIF CGCTAGCTTA

0.1219	0.7394	0.07912	0.05959
0.09634	0.08448	0.7414	0.07775
0.04206	0.8519	0.04346	0.06254
0.03633	0.04898	0.09184	0.8229
0.8128	0.06769	0.05739	0.06208
0.06606	0.08029	0.8102	0.0435
0.05392	0.8146	0.0438	0.08768
0.09844	0.04551	0.09719	0.7589
0.06744	0.08131	0.05544	0.7958
0.8038	0.065	0.06541	0.06578

MOTIF ACTATTAAAC

0.7816	0.08944	0.04337	0.08561
0.1156	0.6969	0.07128	0.1163
0.121	0.05234	0.04693	0.7797
0.8147	0.0738	0.03625	0.07522
0.1346	0.07146	0.04753	0.7464
0.1142	0.1079	0.07942	0.6985
0.8005	0.07543	0.05283	0.0712
0.7343	0.08161	0.07301	0.111
0.7206	0.05164	0.1007	0.1271
0.147	0.6295	0.06703	0.1565

MOTIF CTACAGCT

0.08349	0.783	0.0507	0.08281
0.121	0.05817	0.05489	0.7659
0.7548	0.1103	0.05707	0.07785

0.1298	0.6672	0.1114	0.09163
0.7535	0.07445	0.0633	0.1087
0.1453	0.09055	0.6757	0.08842
0.1072	0.7356	0.08137	0.07584
0.09273	0.07818	0.06908	0.76

MOTIF TGCGGGTC

0.1024	0.06364	0.05609	0.7779
0.06876	0.05	0.7591	0.1222
0.0746	0.7105	0.05983	0.1551
0.07574	0.04257	0.7826	0.0991
0.07195	0.03534	0.7866	0.1061
0.07054	0.06587	0.8119	0.05173
0.06468	0.05753	0.04541	0.8324
0.0865	0.7397	0.08832	0.08551

MOTIF CTAGCTTA

0.07462	0.7627	0.06855	0.09415
0.05415	0.0552	0.1071	0.7836
0.7909	0.04936	0.06691	0.09284
0.1094	0.1132	0.6968	0.08056
0.09279	0.7353	0.0703	0.1016
0.1005	0.08949	0.07726	0.7328
0.07998	0.07503	0.05645	0.7885
0.746	0.09834	0.0621	0.09351

MOTIF CTACGATG

0.128	0.7328	0.06309	0.07618
0.1056	0.07352	0.08338	0.7375
0.8258	0.05162	0.05283	0.06975
0.1068	0.6669	0.1281	0.09825
0.08758	0.09262	0.6728	0.147
0.8206	0.06142	0.0737	0.04432
0.08116	0.06533	0.07392	0.7796
0.09599	0.08935	0.7057	0.109

MOTIF GCAACTAT

0.1526	0.1372	0.5943	0.116
0.1105	0.7013	0.05089	0.1373
0.7945	0.06656	0.03801	0.101
0.7721	0.0884	0.05476	0.08473
0.1005	0.7781	0.06208	0.05932
0.07932	0.05578	0.0751	0.7898
0.7699	0.08604	0.05342	0.09066
0.09089	0.0628	0.08858	0.7577

MOTIF CCTCTAAA

0.08856	0.7074	0.06975	0.1343
0.08781	0.7712	0.05495	0.08603
0.07296	0.05902	0.04425	0.8238

0.05685 0.8023 0.04884 0.09205
0.08598 0.06653 0.04189 0.8056
0.81 0.08114 0.04655 0.06232
0.7741 0.08242 0.05042 0.09302
0.8486 0.05106 0.02937 0.07096

MOTIF ATCGTACC
0.7762 0.07033 0.08388 0.06961
0.04924 0.06564 0.07027 0.8148
0.1162 0.7019 0.07848 0.1034
0.08419 0.1292 0.6949 0.09171
0.08961 0.05837 0.0736 0.7784
0.7607 0.06928 0.08211 0.08791
0.09603 0.6039 0.1056 0.1944
0.1284 0.7108 0.07379 0.08697

MOTIF GTAACTAT
0.1211 0.1261 0.6439 0.1089
0.09581 0.1201 0.0435 0.7406
0.774 0.08855 0.06189 0.0756
0.8008 0.04232 0.05816 0.09867
0.1274 0.7319 0.06226 0.07844
0.06949 0.04958 0.08021 0.8007
0.8081 0.06236 0.05473 0.07477
0.06634 0.02938 0.09397 0.8103

MOTIF CCAGCGTG
0.07881 0.7548 0.1042 0.06214
0.08914 0.6245 0.1348 0.1515
0.8159 0.0479 0.07779 0.05837
0.1094 0.08775 0.7121 0.09077
0.07152 0.7978 0.07999 0.05071
0.1562 0.0744 0.7027 0.06665
0.07685 0.08053 0.04366 0.799
0.1109 0.06334 0.7402 0.08553

MOTIF GGACGACA
0.1239 0.07153 0.7578 0.04684
0.08852 0.1197 0.707 0.08474
0.783 0.05899 0.07599 0.08198
0.0773 0.7095 0.1059 0.1074
0.111 0.04801 0.7612 0.07987
0.8394 0.06207 0.06277 0.03579
0.09971 0.696 0.129 0.07527
0.7954 0.05895 0.06178 0.08389

MOTIF ATTTCG
0.7991 0.0624 0.07273 0.06581
0.07168 0.05079 0.06988 0.8076
0.04643 0.07897 0.05229 0.8223

0.05868	0.04581	0.05114	0.8444
0.1539	0.6448	0.0649	0.1363
0.07591	0.05405	0.7885	0.08157

MOTIF ATCGTA

0.8037	0.05031	0.07071	0.07526
0.04183	0.05269	0.06847	0.837
0.1267	0.6855	0.0675	0.1202
0.09496	0.0869	0.7238	0.09435
0.07491	0.04508	0.03899	0.841
0.7974	0.05359	0.05181	0.09724

MOTIF TACAGC

0.0553	0.06622	0.06712	0.8114
0.7632	0.08226	0.0674	0.0871
0.127	0.6888	0.08835	0.09587
0.8504	0.04651	0.05017	0.05287
0.1011	0.07555	0.7618	0.06154
0.07308	0.7488	0.06149	0.1166

MOTIF GCAACT

0.1288	0.1079	0.6962	0.06709
0.08925	0.7847	0.03832	0.08775
0.8462	0.04207	0.03772	0.07402
0.8341	0.05842	0.05624	0.05123
0.06329	0.7626	0.1073	0.06682
0.08105	0.05241	0.05576	0.8108

MOTIF GCGGGT

0.05712	0.0558	0.7214	0.1657
0.08782	0.7197	0.04954	0.1429
0.07861	0.06873	0.7308	0.1219
0.08112	0.05124	0.7774	0.09023
0.06697	0.04279	0.8164	0.07381
0.06594	0.06427	0.04183	0.828

MOTIF CTTACT

0.06379	0.7982	0.05831	0.07969
0.07148	0.07138	0.03372	0.8234
0.101	0.08036	0.04995	0.7687
0.7393	0.07084	0.07866	0.1112
0.08281	0.7569	0.06274	0.09752
0.05967	0.04454	0.0281	0.8677

MOTIF CGCTAG

0.1626	0.6367	0.08986	0.1108
0.08378	0.06948	0.7604	0.08637
0.04315	0.8276	0.06812	0.06117
0.06073	0.03835	0.04531	0.8556
0.7914	0.08899	0.06327	0.05631

0.06831	0.07043	0.7884	0.07288

MOTIF CCTACA

0.1004	0.7439	0.08054	0.07514
0.07567	0.8041	0.04273	0.07746
0.09895	0.0846	0.04846	0.768
0.7983	0.08226	0.06353	0.05595
0.09597	0.7532	0.06339	0.08741
0.786	0.0684	0.05333	0.09229

MOTIF GCATAC

0.1401	0.07	0.7247	0.06516
0.08692	0.8077	0.07513	0.03024
0.7401	0.1064	0.0578	0.09567
0.08704	0.09338	0.04822	0.7714
0.8219	0.07627	0.04229	0.05954
0.1083	0.7024	0.09967	0.08959

MOTIF CGTATC

0.05516	0.797	0.05772	0.09013
0.1128	0.09992	0.6929	0.09434
0.09092	0.04346	0.06167	0.804
0.7876	0.06696	0.08161	0.06386
0.05909	0.05469	0.07925	0.807
0.08393	0.7836	0.05729	0.07517

MOTIF ACGACA

0.8457	0.04507	0.05406	0.05521
0.0762	0.7629	0.07006	0.09085
0.09688	0.05863	0.7519	0.09264
0.8429	0.05036	0.05453	0.0522
0.09259	0.767	0.08163	0.05878
0.7856	0.07839	0.06535	0.07064

MOTIF CGTAAC

0.1038	0.7325	0.06734	0.09636
0.06749	0.0887	0.7367	0.1071
0.1183	0.1059	0.03817	0.7376
0.693	0.1235	0.09984	0.08369
0.7161	0.06301	0.08999	0.131
0.06321	0.7994	0.04561	0.09182

MOTIF CGTAGA

0.1823	0.6422	0.09079	0.08475
0.08873	0.07323	0.7474	0.09068
0.05174	0.05031	0.08611	0.8118
0.7916	0.08176	0.07708	0.04953
0.05252	0.0467	0.8141	0.08663
0.7813	0.07865	0.06158	0.07851

MOTIF AGACCC

0.7613	0.08448	0.062	0.09218
0.1249	0.1134	0.6562	0.1055
0.8412	0.05646	0.05274	0.04961
0.1194	0.7052	0.09332	0.0821
0.09094	0.7587	0.07255	0.07784
0.08754	0.7949	0.05341	0.06414

MOTIF CTTAGG

0.1201	0.6874	0.0702	0.1223
0.06657	0.02608	0.07453	0.8328
0.1046	0.0458	0.05954	0.7901
0.7807	0.05171	0.08225	0.08537
0.03142	0.0451	0.8284	0.09508
0.08683	0.1008	0.7141	0.09823

MOTIF TAAACG

0.07491	0.07704	0.05618	0.7919
0.8268	0.04277	0.06006	0.07033
0.802	0.05662	0.06972	0.07167
0.8265	0.03993	0.05416	0.07942
0.1242	0.7087	0.08688	0.08022
0.1272	0.1029	0.6403	0.1296

MOTIF GTGACG

0.06071	0.05171	0.8235	0.06412
0.07499	0.04871	0.05401	0.8223
0.07709	0.09117	0.7675	0.06423
0.7556	0.05927	0.06959	0.1156
0.1013	0.6846	0.1119	0.1022
0.1121	0.08691	0.7024	0.09852

MOTIF TCTGAAGACG

0.05885	0.08685	0.1025	0.7518
0.06767	0.8121	0.05145	0.06877
0.06507	0.07788	0.09136	0.7657
0.08743	0.06291	0.802	0.04766
0.7764	0.08722	0.06953	0.06685
0.7506	0.09727	0.0765	0.07559
0.05862	0.04678	0.8399	0.0547
0.8215	0.06541	0.0639	0.04918
0.1201	0.7349	0.06443	0.08063
0.07555	0.07028	0.7839	0.07024

MOTIF AGGGAGTCCA

0.7621	0.1038	0.06558	0.06853
0.1078	0.07067	0.7369	0.0846
0.08893	0.1213	0.7233	0.06649
0.07702	0.06276	0.7931	0.06713
0.7995	0.04413	0.1008	0.05562

0.07877	0.135	0.7063	0.08001
0.06819	0.06661	0.1004	0.7648
0.09973	0.7552	0.0605	0.08454
0.09295	0.7519	0.1003	0.05491
0.7583	0.08579	0.1007	0.05514

MOTIF AGGCGACTGG

0.7642	0.06818	0.0857	0.08196
0.08921	0.04238	0.8147	0.05371
0.08035	0.05174	0.7916	0.07627
0.08274	0.7805	0.07756	0.05917
0.04857	0.1241	0.7592	0.06811
0.7305	0.08761	0.08902	0.09283
0.05912	0.7922	0.08252	0.06614
0.08272	0.05845	0.1048	0.754
0.05505	0.09448	0.7736	0.07685
0.07092	0.06401	0.8134	0.05169

MOTIF TGTGCCCGAA

0.06415	0.09905	0.09737	0.7394
0.07391	0.09045	0.7299	0.1058
0.1215	0.118	0.1244	0.636
0.06975	0.08659	0.7632	0.08047
0.06951	0.7136	0.07578	0.1411
0.09129	0.7952	0.05281	0.0607
0.09917	0.7067	0.121	0.07314
0.09781	0.09301	0.7303	0.0789
0.7564	0.08269	0.08511	0.07585
0.7072	0.1113	0.1038	0.07776

MOTIF GCGGGATT

0.0489	0.09486	0.7569	0.09933
0.1645	0.6579	0.07258	0.1051
0.09841	0.09214	0.7077	0.1017
0.06696	0.0392	0.805	0.08882
0.07851	0.05451	0.8061	0.06084
0.7674	0.07855	0.09562	0.0584
0.08563	0.05711	0.102	0.7552
0.07331	0.09274	0.08946	0.7445

MOTIF TCGTTAGG

0.04326	0.03982	0.08407	0.8328
0.09735	0.6715	0.1112	0.12
0.0528	0.096	0.7324	0.1188
0.08785	0.0762	0.06837	0.7676
0.06246	0.12	0.05273	0.7648
0.686	0.09705	0.1243	0.0926
0.06802	0.0427	0.8381	0.05115
0.05633	0.08047	0.8154	0.04783

MOTIF CCTATGGC

0.04636	0.8418	0.04566	0.06614
0.07845	0.7478	0.06386	0.1099
0.09971	0.1014	0.06961	0.7293
0.7006	0.1228	0.06573	0.1108
0.1472	0.07749	0.1267	0.6487
0.07939	0.09233	0.7702	0.05812
0.05942	0.1301	0.6926	0.1179
0.07638	0.8175	0.05766	0.0485

MOTIF CCAGTCGC

0.0777	0.7705	0.06275	0.08909
0.08308	0.7283	0.1039	0.08467
0.7134	0.1058	0.06729	0.1135
0.08265	0.07884	0.7874	0.05108
0.1375	0.07171	0.1442	0.6467
0.08486	0.7481	0.1178	0.04924
0.07061	0.112	0.742	0.07538
0.09878	0.7539	0.05067	0.0967

MOTIF CTTAAGGC

0.1032	0.7846	0.04958	0.06265
0.08279	0.1465	0.07929	0.6914
0.09949	0.06618	0.09622	0.7381
0.6764	0.1359	0.08961	0.09812
0.6678	0.06889	0.1575	0.1058
0.05405	0.04105	0.8443	0.06059
0.08995	0.0783	0.7609	0.07089
0.08775	0.7608	0.09173	0.05968

MOTIF CCCGAAAA

0.0719	0.7729	0.06151	0.09368
0.09362	0.7973	0.05304	0.05608
0.1031	0.7073	0.1104	0.07923
0.1377	0.1099	0.6647	0.08778
0.7579	0.08112	0.09503	0.06596
0.7854	0.09152	0.08214	0.04093
0.7478	0.07022	0.1379	0.04404
0.7798	0.08216	0.07278	0.06527

MOTIF GCGTCTTG

0.03733	0.09099	0.7444	0.1273
0.05296	0.7723	0.06974	0.105
0.09875	0.07993	0.7015	0.1198
0.09561	0.0684	0.07098	0.765
0.05231	0.8029	0.07709	0.06772
0.09066	0.08533	0.1054	0.7186
0.105	0.1391	0.1048	0.6512
0.04664	0.122	0.7602	0.07113

MOTIF CCGTCTAG

0.06425	0.6781	0.149	0.1086
0.05857	0.7829	0.09707	0.0615
0.1264	0.1238	0.6773	0.07245
0.08311	0.1033	0.07556	0.738
0.04443	0.811	0.08815	0.05645
0.08335	0.09463	0.09222	0.7298
0.6615	0.1517	0.08451	0.1023
0.06552	0.1188	0.7559	0.0598

MOTIF GCCCCT

0.08259	0.08035	0.7706	0.06648
0.04994	0.82	0.06147	0.0686
0.05021	0.8284	0.04652	0.07481
0.05971	0.8189	0.04871	0.07266
0.0791	0.7931	0.04522	0.08257
0.05708	0.07597	0.1048	0.7622

MOTIF GGGCTT

0.0439	0.05539	0.821	0.07971
0.04993	0.04177	0.8621	0.04622
0.0611	0.1103	0.7904	0.03812
0.1159	0.7461	0.0767	0.06126
0.06196	0.09263	0.05789	0.7875
0.07887	0.105	0.05261	0.7635

MOTIF GCGTCT

0.04304	0.1118	0.735	0.1101
0.07764	0.7725	0.07302	0.07688
0.08093	0.09416	0.7515	0.07343
0.09861	0.06022	0.06928	0.7719
0.05391	0.8308	0.0736	0.04166
0.05516	0.04867	0.09253	0.8036

MOTIF CCAATC

0.06382	0.8013	0.06865	0.06628
0.06951	0.8254	0.04701	0.0581
0.7845	0.1043	0.05185	0.05937
0.7611	0.07371	0.08078	0.08441
0.08052	0.07838	0.0652	0.7759
0.06608	0.8286	0.06302	0.04232

MOTIF GCGACT

0.06854	0.1367	0.7127	0.08207
0.06542	0.7987	0.07994	0.05595
0.06925	0.09116	0.7721	0.06748
0.7319	0.06684	0.07688	0.1244
0.04788	0.8319	0.06572	0.05447
0.08396	0.06772	0.1017	0.7466

MOTIF CCTACC

0.08385	0.7949	0.04961	0.07166
0.06748	0.838	0.05014	0.04435
0.1045	0.1062	0.1399	0.6494
0.6804	0.1356	0.09895	0.08505
0.1114	0.7526	0.05315	0.08281
0.06066	0.7881	0.07347	0.07778

MOTIF TCCGTT

0.05433	0.07726	0.06123	0.8072
0.03197	0.7778	0.05522	0.135
0.07399	0.7748	0.0821	0.06909
0.08373	0.08804	0.7032	0.125
0.0842	0.1005	0.0445	0.7708
0.07406	0.08841	0.09173	0.7458

MOTIF AATCCG

0.7695	0.0704	0.09252	0.06761
0.7138	0.1392	0.105	0.04197
0.05332	0.0602	0.06619	0.8203
0.05382	0.8597	0.04123	0.04527
0.05937	0.8251	0.04251	0.073
0.1571	0.1448	0.6289	0.06914

MOTIF CTTAGG

0.06615	0.7089	0.1063	0.1187
0.08016	0.03769	0.06902	0.8131
0.06228	0.08591	0.08785	0.764
0.6681	0.1149	0.09449	0.1225
0.03043	0.05443	0.8601	0.05504
0.05564	0.07463	0.8236	0.04609

MOTIF GCTCAG

0.07885	0.08717	0.7555	0.07853
0.06816	0.7924	0.06985	0.06957
0.05014	0.07973	0.06761	0.8025
0.06642	0.7223	0.1118	0.09947
0.686	0.1113	0.1325	0.07023
0.08404	0.04054	0.8165	0.05891

MOTIF GGGTCC

0.05074	0.04929	0.8221	0.07786
0.1287	0.04207	0.766	0.06328
0.03806	0.07534	0.8258	0.06079
0.07845	0.03201	0.1197	0.7699
0.05295	0.7775	0.1091	0.0604
0.06048	0.7598	0.1087	0.07103

MOTIF GGAGTC

0.04206	0.1183	0.7807	0.05888

0.05277	0.03978	0.8667	0.04071
0.82	0.06372	0.07095	0.04537
0.06425	0.1145	0.7676	0.05361
0.06709	0.07212	0.09367	0.7671
0.1235	0.6861	0.07501	0.1154

MOTIF CCTAAC

0.05687	0.838	0.0518	0.05335
0.04786	0.8646	0.05046	0.0371
0.1149	0.1168	0.101	0.6673
0.7792	0.05645	0.0864	0.07791
0.7691	0.09219	0.05124	0.08743
0.1311	0.692	0.1045	0.07239

MOTIF TACAGC

0.07981	0.08648	0.06874	0.765
0.6883	0.09659	0.1166	0.09853
0.06101	0.7893	0.07899	0.0707
0.7598	0.05859	0.06552	0.1161
0.0702	0.08009	0.7897	0.06003
0.06278	0.7909	0.08767	0.05863

MOTIF CTGGGT

0.07671	0.7269	0.06623	0.1302
0.05248	0.091	0.09386	0.7627
0.04549	0.04611	0.8499	0.05854
0.07183	0.05569	0.7869	0.08555
0.07635	0.06909	0.7998	0.0548
0.06263	0.07025	0.07562	0.7915

MOTIF GGACGT

0.06028	0.1219	0.7534	0.06445
0.05814	0.06575	0.8156	0.06046
0.7149	0.0785	0.09021	0.1164
0.0697	0.7517	0.1146	0.06405
0.07277	0.07419	0.7525	0.1005
0.1196	0.06155	0.1275	0.6914

MOTIF TCAACGACGA

0.1266	0.05735	0.1341	0.682
0.05839	0.8227	0.06554	0.05335
0.7087	0.1223	0.1118	0.05724
0.7967	0.0515	0.09189	0.05995
0.09117	0.6977	0.06857	0.1426
0.09333	0.05768	0.7902	0.05883
0.8603	0.02861	0.07097	0.04014
0.09061	0.7708	0.08067	0.05791
0.06717	0.09957	0.7711	0.0622
0.7929	0.07448	0.08191	0.05071

MOTIF TCGCTTCCCC

0.104	0.1082	0.04799	0.7398
0.08063	0.7473	0.07272	0.0993
0.06188	0.1124	0.6632	0.1625
0.09551	0.7499	0.07677	0.07786
0.0749	0.1406	0.07919	0.7053
0.02781	0.09302	0.1199	0.7593
0.07196	0.735	0.1283	0.06468
0.1003	0.7253	0.06159	0.1128
0.1288	0.6782	0.08199	0.1109
0.06219	0.7348	0.07574	0.1272

MOTIF TAGCTCGGCC

0.07357	0.1069	0.08784	0.7317
0.6904	0.08791	0.1335	0.08814
0.08992	0.04883	0.7989	0.06239
0.08555	0.8071	0.04692	0.06046
0.05432	0.1302	0.06669	0.7488
0.07153	0.725	0.106	0.09755
0.07428	0.07586	0.7728	0.07702
0.09922	0.07108	0.7424	0.08727
0.07017	0.7754	0.08	0.07439
0.07456	0.7682	0.07162	0.08563

MOTIF GTAGACGCTC

0.06752	0.1302	0.7327	0.06954
0.1032	0.07991	0.1176	0.6992
0.6584	0.1037	0.1331	0.1047
0.06677	0.05828	0.7669	0.1081
0.6958	0.1177	0.06181	0.1247
0.1675	0.5443	0.09843	0.1898
0.05557	0.06713	0.8046	0.07267
0.1217	0.7307	0.06646	0.08109
0.05684	0.05045	0.07625	0.8165
0.06287	0.7581	0.09133	0.08767

MOTIF AGCTCCTT

0.7214	0.06597	0.09435	0.1183
0.07806	0.1046	0.7056	0.1118
0.05814	0.7902	0.07091	0.08075
0.09669	0.06652	0.0694	0.7674
0.07194	0.7415	0.09985	0.08667
0.08617	0.7754	0.05535	0.08311
0.08008	0.05884	0.04692	0.8142
0.06337	0.1388	0.08385	0.714

MOTIF TTGCCGGA

0.0477	0.07218	0.1085	0.7716
0.1028	0.07612	0.1101	0.711
0.1292	0.04499	0.7688	0.05701

0.09067	0.7206	0.1004	0.08842
0.08796	0.7322	0.0665	0.1133
0.07695	0.09141	0.7593	0.07233
0.09717	0.07363	0.7244	0.1048
0.7751	0.07537	0.08616	0.06341

MOTIF CGGCTCCA

0.08068	0.7567	0.08502	0.07763
0.1143	0.06613	0.7054	0.1142
0.07603	0.124	0.7176	0.08235
0.04634	0.8047	0.05313	0.09581
0.06779	0.07968	0.04388	0.8086
0.07367	0.7552	0.08995	0.08116
0.102	0.7252	0.08285	0.08994
0.7042	0.09435	0.06589	0.1355

MOTIF GATCAGGA

0.06631	0.05331	0.8337	0.04665
0.7948	0.09017	0.06941	0.0456
0.06856	0.08991	0.122	0.7196
0.09481	0.6572	0.1809	0.06713
0.8054	0.06252	0.04499	0.08714
0.1172	0.09694	0.6739	0.1119
0.06371	0.0781	0.8071	0.05113
0.7548	0.05582	0.104	0.08537

MOTIF CGTCGTTG

0.08872	0.7303	0.09214	0.08887
0.07195	0.1268	0.7391	0.06215
0.03965	0.08158	0.0515	0.8273
0.06078	0.7503	0.07421	0.1147
0.1106	0.1065	0.6971	0.08578
0.07437	0.08387	0.05737	0.7844
0.0712	0.1667	0.1021	0.66
0.04153	0.08213	0.8027	0.07364

MOTIF ACAATGCG

0.719	0.1058	0.1114	0.06383
0.1038	0.7392	0.1036	0.05337
0.7919	0.06474	0.1017	0.04166
0.7997	0.04709	0.06905	0.08416
0.05079	0.07623	0.1231	0.7499
0.09972	0.07435	0.762	0.06393
0.1424	0.72	0.0688	0.0688
0.1125	0.09917	0.7317	0.05659

MOTIF AGTGGCTC

0.7243	0.07536	0.1013	0.09911
0.08563	0.1155	0.6752	0.1237
0.08794	0.1533	0.06471	0.694

0.0984	0.05918	0.7173	0.1251
0.06877	0.06687	0.8207	0.04366
0.06262	0.7655	0.08183	0.09
0.08289	0.07669	0.0716	0.7688
0.06837	0.7426	0.08339	0.1056

MOTIF CGCATTCT

0.07447	0.6832	0.08797	0.1544
0.08508	0.09356	0.6887	0.1327
0.0493	0.7955	0.04615	0.109
0.7174	0.1256	0.07754	0.07946
0.0769	0.0551	0.0742	0.7938
0.04621	0.1024	0.08537	0.766
0.06345	0.6865	0.1299	0.1201
0.0711	0.07497	0.07289	0.781

MOTIF ACTGGCCG

0.713	0.113	0.1047	0.06927
0.07439	0.7441	0.1269	0.05464
0.0798	0.1069	0.07616	0.7371
0.08093	0.06649	0.7466	0.106
0.07744	0.07783	0.7243	0.1204
0.05649	0.7916	0.07682	0.07507
0.0712	0.7428	0.06493	0.1211
0.0984	0.09842	0.7429	0.06025

MOTIF GATCTGCT

0.0727	0.155	0.7059	0.06646
0.7542	0.08752	0.06289	0.09537
0.09897	0.05517	0.1247	0.7211
0.07162	0.7116	0.1018	0.115
0.1215	0.05213	0.07395	0.7524
0.08356	0.1002	0.6957	0.1205
0.07559	0.7535	0.08908	0.08183
0.07158	0.06447	0.07802	0.7859

MOTIF GAGCGCAT

0.05824	0.06714	0.8038	0.07078
0.7207	0.1281	0.07748	0.07371
0.07783	0.1065	0.7302	0.0855
0.1501	0.6951	0.0702	0.08462
0.1756	0.1023	0.6095	0.1126
0.06508	0.7544	0.0851	0.09546
0.7987	0.08135	0.07304	0.04687
0.07886	0.06139	0.08386	0.7759

MOTIF TAGTCCTC

0.06863	0.06244	0.09623	0.7727
0.6431	0.1682	0.08664	0.102
0.08101	0.1486	0.6926	0.07782

0.07831	0.07468	0.04565	0.8014
0.05778	0.756	0.08918	0.09704
0.07282	0.7356	0.112	0.07953
0.1007	0.06193	0.09299	0.7444
0.04602	0.7448	0.07184	0.1374

MOTIF TTAACCGG

0.12	0.1142	0.08426	0.6816
0.07068	0.08103	0.1064	0.7419
0.7015	0.1302	0.07724	0.09103
0.7643	0.07683	0.08087	0.07801
0.08705	0.7411	0.106	0.0659
0.07137	0.7444	0.09608	0.08811
0.06854	0.06542	0.7824	0.08362
0.08251	0.08235	0.699	0.1361

MOTIF CGGTTA

0.0792	0.7528	0.06748	0.1005
0.05121	0.05576	0.8223	0.07071
0.05	0.07112	0.7972	0.08171
0.06922	0.071	0.04678	0.813
0.08589	0.08319	0.1087	0.7222
0.7789	0.09046	0.06447	0.06621

MOTIF AATGCG

0.7658	0.06959	0.1198	0.04479
0.8287	0.04845	0.06356	0.05934
0.04988	0.08659	0.07537	0.7882
0.05154	0.05801	0.8418	0.04866
0.1269	0.702	0.08354	0.08761
0.1034	0.07365	0.7396	0.08339

MOTIF AACGCG

0.7571	0.06842	0.108	0.06651
0.7879	0.08222	0.06138	0.0685
0.05836	0.7223	0.09008	0.1292
0.09657	0.058	0.7905	0.05497
0.1189	0.7791	0.05653	0.04547
0.08198	0.07263	0.7739	0.0715

MOTIF CGTTAA

0.06538	0.7773	0.08128	0.07608
0.1106	0.09099	0.694	0.1044
0.08824	0.06244	0.0881	0.7612
0.06143	0.0893	0.06546	0.7838
0.7086	0.09018	0.1236	0.07764
0.8465	0.04567	0.05946	0.04835

MOTIF GCTCCT

0.07916	0.1188	0.7132	0.08876

0.06873	0.8328	0.0481	0.05036
0.06685	0.04759	0.04703	0.8385
0.05796	0.7535	0.1048	0.08372
0.08052	0.7891	0.05963	0.07077
0.08356	0.06222	0.05632	0.7979

MOTIF ACTGGC

0.7754	0.08562	0.08034	0.05867
0.0936	0.7383	0.1073	0.06072
0.05464	0.0729	0.08144	0.791
0.05364	0.04557	0.8012	0.09956
0.07262	0.07941	0.7505	0.09746
0.05553	0.8411	0.05677	0.0466

MOTIF AGGACT

0.7486	0.0996	0.07371	0.07809
0.06532	0.06935	0.8093	0.05606
0.09054	0.09398	0.8019	0.01358
0.8461	0.02109	0.06	0.07277
0.08444	0.7317	0.1121	0.0718
0.07256	0.05964	0.07808	0.7897

MOTIF CGTTCA

0.1239	0.698	0.09336	0.08479
0.07078	0.07938	0.7775	0.07235
0.07	0.0563	0.07853	0.7952
0.04373	0.1211	0.0998	0.7354
0.07874	0.7368	0.1138	0.07067
0.8316	0.05219	0.03566	0.08054

MOTIF TTACCG

0.03774	0.07574	0.05246	0.8341
0.1483	0.1182	0.0898	0.6437
0.7483	0.06108	0.1063	0.08426
0.09808	0.7323	0.07002	0.09961
0.05407	0.8023	0.0565	0.08713
0.08906	0.09414	0.7434	0.07339

MOTIF GATCAC

0.07262	0.08833	0.8008	0.03821
0.7516	0.09857	0.07725	0.07261
0.05822	0.06696	0.07672	0.7981
0.04782	0.8314	0.0776	0.04315
0.8019	0.04586	0.06831	0.08389
0.1093	0.7065	0.1003	0.08393

MOTIF TTGCGA

0.08604	0.09805	0.08141	0.7345
0.04903	0.03275	0.09987	0.8184
0.05031	0.0613	0.8196	0.06876

0.1257	0.6293	0.06054	0.1845
0.0364	0.106	0.7368	0.1208
0.7485	0.07846	0.07377	0.09924

MOTIF CGGCTC

0.06945	0.7689	0.05116	0.1105
0.08576	0.07295	0.733	0.1083
0.07232	0.08969	0.7609	0.0771
0.06274	0.8296	0.0388	0.06882
0.06058	0.06543	0.04648	0.8275
0.05491	0.8239	0.07207	0.04916

MOTIF CATCGC

0.03202	0.8412	0.07792	0.0489
0.7243	0.09738	0.1023	0.07601
0.1151	0.0762	0.05078	0.7579
0.05248	0.7198	0.09481	0.133
0.09963	0.07537	0.7017	0.1233
0.07016	0.7951	0.04973	0.08499

MOTIF TAGATC

0.06856	0.09688	0.08429	0.7503
0.7223	0.08001	0.07935	0.1183
0.06414	0.06386	0.8183	0.05374
0.772	0.09432	0.05651	0.07718
0.06161	0.05126	0.06793	0.8192
0.05466	0.7939	0.09857	0.05284

MOTIF CGGAAT

0.1267	0.6666	0.09423	0.1125
0.114	0.07018	0.7093	0.1065
0.0674	0.09499	0.7805	0.05714
0.7891	0.08143	0.0844	0.04506
0.6691	0.1501	0.09465	0.08611
0.1018	0.05414	0.0775	0.7666

MOTIF TCAGGA

0.08339	0.09535	0.06985	0.7514
0.1098	0.6924	0.1357	0.06208
0.8639	0.02172	0.03235	0.08203
0.106	0.09512	0.7588	0.04012
0.08477	0.09465	0.7693	0.05132
0.7637	0.06064	0.07991	0.09573

MOTIF AGTGCG

0.7592	0.07433	0.08099	0.08548
0.1214	0.05351	0.7585	0.06666
0.08083	0.06043	0.08036	0.7784
0.03938	0.1102	0.797	0.05343
0.1312	0.6643	0.1162	0.08824

0.09681	0.08653	0.7173	0.09939

MOTIF TGGACG

0.07448	0.04085	0.06337	0.8213
0.05378	0.07879	0.7975	0.06996
0.07643	0.08754	0.7923	0.04377
0.7713	0.09923	0.06035	0.06914
0.1333	0.6859	0.1064	0.07438
0.06896	0.0788	0.7808	0.07143

MOTIF GATCGC

0.03309	0.115	0.7993	0.0526
0.7307	0.108	0.09391	0.06746
0.06563	0.07415	0.07202	0.7882
0.06125	0.7753	0.1001	0.06335
0.1521	0.05703	0.6866	0.1043
0.09624	0.7426	0.05935	0.1018

MOTIF GACGCT

0.07192	0.0698	0.7927	0.0656
0.7571	0.1104	0.06096	0.07147
0.1226	0.6357	0.1087	0.1329
0.07794	0.06588	0.7877	0.06846
0.1291	0.7407	0.0825	0.0477
0.07741	0.05822	0.1064	0.7579

MOTIF CGTTGA

0.1021	0.7733	0.06001	0.06455
0.08751	0.0917	0.7413	0.0795
0.05953	0.06091	0.07864	0.8009
0.03999	0.07391	0.08714	0.799
0.06512	0.06762	0.8071	0.0602
0.7284	0.08941	0.09974	0.08248

MOTIF ACCTCTGCGT

0.7876	0.08536	0.08165	0.04538
0.08381	0.8133	0.0517	0.05118
0.05715	0.8337	0.07225	0.03686
0.06017	0.09744	0.05917	0.7832
0.02902	0.895	0.03652	0.03943
0.06917	0.07097	0.06502	0.7948
0.04805	0.05444	0.8274	0.07014
0.04343	0.8575	0.05668	0.04241
0.0511	0.09625	0.7936	0.05909
0.03674	0.1001	0.06242	0.8007

MOTIF ATCGCCGTCT

0.615	0.1262	0.1381	0.1207
0.04842	0.1109	0.0924	0.7483
0.05428	0.7892	0.08188	0.07466

0.07945	0.09127	0.7436	0.08565
0.07786	0.7362	0.1161	0.06984
0.06697	0.8197	0.06253	0.05078
0.09008	0.06337	0.77	0.07657
0.05923	0.08273	0.1171	0.741
0.03631	0.8104	0.08804	0.06529
0.07924	0.146	0.08095	0.6938

MOTIF GGCCGTAC

0.04878	0.07492	0.796	0.08029
0.0743	0.0593	0.8378	0.02865
0.04835	0.8435	0.05268	0.0555
0.06397	0.8327	0.05136	0.05198
0.07572	0.07594	0.801	0.04735
0.08718	0.1409	0.1694	0.6026
0.68	0.07245	0.1597	0.08788
0.04838	0.784	0.1009	0.06672

MOTIF GTCTTAGT

0.08335	0.06854	0.7904	0.05773
0.08162	0.055	0.1094	0.7539
0.04123	0.8497	0.07015	0.03897
0.07993	0.07314	0.112	0.7349
0.04738	0.1034	0.1322	0.717
0.69	0.1417	0.08473	0.08351
0.06082	0.07143	0.8201	0.04768
0.09512	0.1415	0.142	0.6213

MOTIF AGACCGCG

0.7053	0.1035	0.1029	0.08832
0.03823	0.09033	0.8365	0.03499
0.7009	0.1061	0.09378	0.09923
0.03331	0.826	0.09227	0.04846
0.07847	0.7028	0.1511	0.06762
0.04695	0.1119	0.7814	0.05978
0.09108	0.7305	0.1088	0.06958
0.04389	0.1123	0.8015	0.04232

MOTIF CACCGGCG

0.04819	0.796	0.1163	0.03954
0.6862	0.1406	0.06487	0.1083
0.09273	0.7088	0.1147	0.08384
0.07794	0.7733	0.09585	0.05288
0.07514	0.1039	0.7153	0.1056
0.09534	0.1468	0.6842	0.07364
0.04216	0.8609	0.078	0.01897
0.1042	0.1719	0.6023	0.1215

MOTIF CAGCGTGC

0.09903	0.7524	0.08344	0.06516

0.6644	0.111	0.09197	0.1326
0.04743	0.1182	0.7567	0.07767
0.06238	0.7499	0.1042	0.0836
0.07174	0.09922	0.7559	0.07316
0.08809	0.1031	0.1212	0.6876
0.07177	0.1004	0.7935	0.03436
0.06756	0.7711	0.06529	0.09605

MOTIF ATCGTCCC

0.6464	0.1331	0.1325	0.08805
0.1142	0.1366	0.09958	0.6496
0.04138	0.8228	0.07326	0.06258
0.04399	0.07885	0.8129	0.06426
0.09644	0.08451	0.1431	0.676
0.03864	0.8179	0.09094	0.05254
0.03717	0.7659	0.1281	0.06883
0.07798	0.6733	0.1757	0.07295

MOTIF GGCCGGAC

0.08417	0.1091	0.7381	0.06865
0.04743	0.1118	0.8228	0.01798
0.06917	0.6981	0.1226	0.1101
0.05977	0.8032	0.09032	0.04675
0.06848	0.08494	0.7857	0.06086
0.07485	0.1122	0.7187	0.0943
0.7326	0.033	0.1097	0.1248
0.04693	0.7547	0.1164	0.08194

MOTIF AGACGG

0.7839	0.06536	0.0919	0.05883
0.04667	0.07467	0.8535	0.02518
0.7812	0.1067	0.07319	0.03896
0.05771	0.7808	0.08962	0.07187
0.06043	0.06376	0.8118	0.064
0.04977	0.05877	0.8363	0.05513

MOTIF TCCGCA

0.06407	0.08383	0.0981	0.754
0.06037	0.8402	0.02373	0.07568
0.06877	0.8122	0.05486	0.06419
0.06251	0.08468	0.7873	0.06553
0.04117	0.894	0.02573	0.0391
0.6123	0.1667	0.1317	0.08923

MOTIF ACGGCC

0.6361	0.1642	0.1076	0.09216
0.05787	0.8167	0.06528	0.0602
0.04999	0.08082	0.8123	0.05693
0.04428	0.06573	0.8275	0.06246
0.04889	0.8158	0.05155	0.08375

0.08022 0.7613 0.106 0.05247

MOTIF ACCGCG
0.6576 0.1054 0.1107 0.1263
0.04294 0.8412 0.07632 0.03953
0.07511 0.7063 0.1489 0.06964
0.07663 0.06423 0.7828 0.07636
0.06487 0.8146 0.05194 0.06862
0.06176 0.1102 0.7707 0.05735

MOTIF CGATAA
0.09969 0.7593 0.07076 0.07025
0.04278 0.07442 0.8311 0.05167
0.7614 0.08419 0.0735 0.08086
0.1373 0.1218 0.145 0.5959
0.6855 0.07864 0.1711 0.06476
0.7186 0.09607 0.09623 0.0891

MOTIF CCCCGA
0.05984 0.7447 0.1583 0.03717
0.06658 0.782 0.1006 0.05081
0.06578 0.8336 0.06005 0.0406
0.0592 0.7905 0.09311 0.05723
0.08036 0.1214 0.7367 0.06161
0.7559 0.09082 0.08278 0.07047

MOTIF GTGCGA
0.06743 0.07667 0.7981 0.05778
0.06934 0.06663 0.1023 0.7617
0.05701 0.1234 0.7745 0.04505
0.06168 0.776 0.07817 0.08411
0.03855 0.07143 0.8002 0.08984
0.6699 0.06927 0.1636 0.0972

MOTIF ACGTCC
0.639 0.1393 0.1009 0.1208
0.03921 0.8365 0.07036 0.05394
0.04687 0.08497 0.8153 0.05287
0.0618 0.09517 0.148 0.695
0.09133 0.7955 0.07513 0.03808
0.06468 0.7491 0.1023 0.08397

MOTIF ACGCAG
0.7401 0.08182 0.1112 0.06686
0.05815 0.7493 0.1365 0.0561
0.06687 0.04948 0.8057 0.07792
0.07301 0.7964 0.07557 0.05507
0.7603 0.07964 0.07359 0.08647
0.03942 0.08856 0.8146 0.05743

MOTIF TAAGAC

0.09343	0.09522	0.1234	0.688
0.7656	0.09196	0.08446	0.05798
0.7906	0.06036	0.09474	0.05426
0.03853	0.06568	0.8556	0.04024
0.7983	0.0849	0.06112	0.0557
0.04263	0.8631	0.04188	0.05239

MOTIF ACCGGC

0.7261	0.1105	0.0436	0.1198
0.06351	0.7983	0.06918	0.06898
0.06343	0.8214	0.05541	0.05973
0.06923	0.07877	0.7913	0.0607
0.06386	0.0936	0.7282	0.1143
0.0417	0.9018	0.04523	0.01126

MOTIF GGAGTG

0.07248	0.07097	0.8017	0.05487
0.06565	0.0902	0.7943	0.04982
0.698	0.1097	0.09633	0.09593
0.03431	0.07876	0.8369	0.05008
0.1019	0.1109	0.1178	0.6694
0.04684	0.05956	0.8106	0.08297

MOTIF GCGTAA

0.0684	0.09061	0.7806	0.06041
0.06473	0.7827	0.09132	0.06128
0.06182	0.1248	0.7315	0.08181
0.1392	0.0698	0.1152	0.6758
0.7257	0.09898	0.1006	0.07473
0.7214	0.1021	0.09399	0.08251

MOTIF GGATCG

0.05918	0.04525	0.8473	0.04826
0.03405	0.08554	0.8343	0.04609
0.5754	0.1678	0.1971	0.05965
0.06546	0.1496	0.1272	0.6577
0.07599	0.7865	0.0691	0.06841
0.06049	0.03705	0.8553	0.04719

MOTIF CTCAGA

0.05521	0.8146	0.08358	0.04666
0.06047	0.07265	0.1076	0.7593
0.09618	0.7422	0.1168	0.0448
0.7602	0.06632	0.09058	0.08289
0.04745	0.0665	0.8416	0.04445
0.7384	0.1124	0.07848	0.07066

MOTIF GCCGGA

0.03543	0.07593	0.8735	0.01518

0.06242	0.7722	0.1268	0.03854
0.05591	0.8221	0.06475	0.05722
0.05423	0.127	0.7447	0.07406
0.06134	0.07329	0.8097	0.05568
0.7631	0.04069	0.09659	0.09962

MOTIF ATTAATCTAG

0.7871	0.03471	0.07051	0.1077
0.1069	0.07598	0.06546	0.7516
0.07195	0.08706	0.06287	0.7781
0.7973	0.07752	0.04061	0.08461
0.797	0.04265	0.07432	0.08599
0.1082	0.05198	0.04347	0.7964
0.09799	0.593	0.1287	0.1803
0.07276	0.06281	0.04685	0.8176
0.7885	0.05751	0.06138	0.09259
0.1291	0.08057	0.6734	0.117

MOTIF TATTACCAGG

0.07629	0.08062	0.0463	0.7968
0.7526	0.08744	0.07588	0.08414
0.08285	0.0379	0.03641	0.8428
0.06674	0.04153	0.05631	0.8354
0.8749	0.03901	0.03281	0.05325
0.07395	0.7898	0.06978	0.06646
0.07067	0.8206	0.04784	0.06091
0.8592	0.02892	0.05554	0.05633
0.07557	0.04254	0.7817	0.1002
0.1151	0.05618	0.7455	0.08321

MOTIF TTACCAGG

0.08695	0.0616	0.05716	0.7943
0.06482	0.05084	0.08962	0.7947
0.8521	0.05088	0.03061	0.06638
0.07963	0.7467	0.05953	0.1141
0.1015	0.7672	0.06654	0.06475
0.8348	0.03938	0.05823	0.06761
0.1076	0.05989	0.7214	0.1111
0.09699	0.07672	0.7223	0.104

MOTIF GGCTCTAT

0.1008	0.04554	0.6689	0.1848
0.0747	0.1161	0.6296	0.1796
0.086	0.6883	0.09404	0.1316
0.09614	0.08805	0.03107	0.7847
0.1412	0.6027	0.1095	0.1465
0.05755	0.1111	0.06097	0.7704
0.79	0.06086	0.0753	0.07386
0.07127	0.09233	0.06656	0.7698

MOTIF GGTAACAT

0.09812	0.05292	0.6805	0.1685
0.1582	0.06826	0.6613	0.1122
0.08982	0.1008	0.07007	0.7393
0.8082	0.07806	0.04044	0.0733
0.7661	0.06315	0.07044	0.1004
0.1307	0.643	0.07344	0.1529
0.8032	0.06798	0.05005	0.07878
0.0885	0.05494	0.0603	0.7963

MOTIF GTCCTTAT

0.08558	0.1005	0.7024	0.1115
0.07918	0.07244	0.07352	0.7749
0.08142	0.7416	0.06499	0.112
0.08827	0.7163	0.0581	0.1373
0.1557	0.05323	0.06333	0.7278
0.0703	0.06402	0.08972	0.7759
0.766	0.05711	0.08747	0.08945
0.08931	0.05197	0.03864	0.8201

MOTIF TTTCGGAT

0.06741	0.04547	0.08761	0.7995
0.06394	0.05898	0.05303	0.824
0.09366	0.03784	0.05493	0.8136
0.1481	0.6238	0.07737	0.1508
0.1597	0.06289	0.6644	0.1129
0.09132	0.06591	0.6912	0.1515
0.7946	0.05571	0.06602	0.08365
0.04444	0.04513	0.03283	0.8776

MOTIF ATATCTTC

0.7597	0.05962	0.0518	0.1289
0.08878	0.07899	0.05154	0.7807
0.7985	0.06232	0.06968	0.06949
0.07568	0.04617	0.04461	0.8335
0.1117	0.5646	0.1232	0.2004
0.1578	0.07443	0.08148	0.6863
0.09971	0.06165	0.06517	0.7735
0.1408	0.6646	0.0944	0.1001

MOTIF GTACGAAC

0.1525	0.06341	0.7218	0.06224
0.06694	0.09803	0.05808	0.777
0.8319	0.07885	0.05721	0.03202
0.07976	0.7223	0.08954	0.1084
0.1033	0.1199	0.6555	0.1213
0.7989	0.08302	0.08625	0.03185
0.7484	0.07619	0.06526	0.1102
0.1222	0.7417	0.05997	0.07607

MOTIF TAACGT

0.03678	0.06875	0.05499	0.8395
0.8416	0.04526	0.04153	0.07161
0.8254	0.04788	0.03039	0.09636
0.1039	0.649	0.08443	0.1627
0.1861	0.09914	0.6205	0.09431
0.08677	0.04466	0.03257	0.836

MOTIF ATTAAC

0.8773	0.03946	0.01996	0.06323
0.1005	0.04642	0.04288	0.8102
0.09996	0.04979	0.06273	0.7875
0.8088	0.05918	0.04748	0.08451
0.8003	0.07246	0.05849	0.0688
0.1576	0.7466	0.05853	0.03726

MOTIF TATCGA

0.07606	0.0496	0.05057	0.8238
0.863	0.02626	0.06046	0.05027
0.07808	0.04599	0.02941	0.8465
0.1248	0.6616	0.1203	0.09333
0.08684	0.08635	0.7349	0.09195
0.8296	0.05209	0.03782	0.08051

MOTIF TGCGTA

0.06436	0.05378	0.05151	0.8303
0.046	0.1079	0.7303	0.1158
0.09555	0.5551	0.1595	0.1899
0.1288	0.06548	0.6959	0.1098
0.05059	0.01856	0.08122	0.8496
0.8425	0.06252	0.05263	0.04237

MOTIF TACCAG

0.06746	0.06072	0.07566	0.7962
0.8209	0.06874	0.05296	0.05739
0.07551	0.7582	0.0768	0.08944
0.1033	0.7727	0.06324	0.06072
0.858	0.03905	0.04174	0.06126
0.1175	0.06007	0.7138	0.1087

MOTIF TATGGC

0.06929	0.05754	0.04471	0.8285
0.8255	0.045	0.05281	0.0767
0.06042	0.02563	0.04942	0.8645
0.08074	0.07246	0.7439	0.1029
0.1183	0.06779	0.7178	0.09613
0.1585	0.6421	0.07712	0.1223

MOTIF CGTGTA

0.1135	0.6614	0.06062	0.1644

0.1393	0.07648	0.6451	0.1391
0.05179	0.04487	0.05454	0.8488
0.1069	0.08103	0.7196	0.09246
0.06166	0.04946	0.0599	0.829
0.7974	0.07925	0.06297	0.06038

MOTIF CATACG

0.1013	0.7379	0.05447	0.1064
0.8494	0.05471	0.02837	0.0675
0.06024	0.0719	0.04687	0.821
0.8288	0.06929	0.04866	0.05325
0.155	0.6686	0.07849	0.09787
0.1304	0.13	0.611	0.1286

MOTIF TCCGTA

0.06714	0.04382	0.03065	0.8584
0.03834	0.7898	0.08343	0.08844
0.08593	0.709	0.07346	0.1316
0.2021	0.05691	0.6111	0.1298
0.1086	0.06463	0.02947	0.7973
0.8207	0.05865	0.05756	0.06306

MOTIF GGTAAC

0.07257	0.06312	0.7252	0.1392
0.1063	0.0702	0.7855	0.03802
0.1073	0.06126	0.04738	0.7841
0.8052	0.07784	0.04274	0.07419
0.8395	0.03078	0.04491	0.08482
0.1321	0.6642	0.0808	0.1228

MOTIF CCTAGT

0.04009	0.7959	0.05498	0.109
0.0716	0.7906	0.0388	0.09896
0.1068	0.06914	0.03851	0.7855
0.6697	0.06984	0.1104	0.15
0.08956	0.06411	0.7479	0.09842
0.09531	0.05759	0.02031	0.8268

MOTIF GCTGTA

0.141	0.06616	0.6653	0.1275
0.1211	0.6707	0.05413	0.1541
0.08294	0.02937	0.03062	0.8571
0.09102	0.0967	0.6985	0.1138
0.07577	0.05628	0.04671	0.8212
0.8419	0.0519	0.0487	0.05753

MOTIF ATTTCG

0.8429	0.03617	0.04898	0.07199
0.05357	0.03599	0.04514	0.8653
0.1098	0.03942	0.06707	0.7837

0.05382	0.04832	0.04759	0.8503
0.1521	0.5961	0.1093	0.1425
0.1639	0.09424	0.6561	0.08575

MOTIF ACTGTA

0.7663	0.05239	0.08118	0.1001
0.1434	0.6961	0.07389	0.0866
0.07749	0.04572	0.04723	0.8296
0.11	0.06724	0.7206	0.1022
0.08098	0.03914	0.04644	0.8334
0.7753	0.07029	0.08689	0.06756

MOTIF GTTACA

0.1348	0.05596	0.7162	0.09307
0.1085	0.05675	0.04263	0.7921
0.108	0.05482	0.0484	0.7888
0.8003	0.06705	0.05811	0.07458
0.07805	0.7376	0.07906	0.1053
0.8216	0.07071	0.04987	0.05779

MOTIF CGATTA

0.1868	0.6322	0.08406	0.09702
0.1017	0.1298	0.6467	0.1217
0.8047	0.0585	0.05335	0.08341
0.1101	0.05685	0.04417	0.7888
0.1012	0.04693	0.04939	0.8025
0.8408	0.057	0.03615	0.06605

MOTIF AGTAGA

0.7632	0.04711	0.07864	0.111
0.1379	0.06059	0.6786	0.1229
0.1097	0.05842	0.09042	0.7415
0.8238	0.04609	0.06349	0.06661
0.08618	0.05723	0.7754	0.08118
0.8291	0.06647	0.04546	0.05902

MOTIF TCTGTA

0.09147	0.0454	0.07218	0.791
0.1162	0.667	0.09162	0.1252
0.05798	0.05111	0.04548	0.8454
0.1165	0.07914	0.7256	0.07878
0.06756	0.0456	0.05103	0.8358
0.8114	0.04531	0.05429	0.08898

MOTIF TATACG

0.06953	0.04786	0.04152	0.8411
0.8324	0.04596	0.06407	0.05762
0.06497	0.08239	0.05643	0.7962
0.755	0.1124	0.0486	0.084
0.1025	0.6392	0.07735	0.1809

0.1037	0.07309	0.7428	0.08034

MOTIF TTCGTA

0.06389	0.07229	0.06516	0.7987
0.03432	0.07699	0.07394	0.8148
0.1378	0.5639	0.1241	0.1742
0.1287	0.07669	0.7038	0.09084
0.02221	0.03735	0.08858	0.8519
0.8001	0.0732	0.05073	0.07592

MOTIF CGGATA

0.1011	0.7254	0.04253	0.131
0.1527	0.05195	0.692	0.1034
0.1038	0.06034	0.7422	0.09366
0.8676	0.03268	0.04589	0.05382
0.06836	0.05687	0.05108	0.8237
0.7983	0.06607	0.05112	0.08455

MOTIF TCCGCA

0.04772	0.04768	0.04158	0.863
0.1261	0.7964	0.02044	0.05714
0.1107	0.7158	0.06875	0.1047
0.2109	0.0978	0.5867	0.1046
0.1611	0.6701	0.03972	0.1291
0.8026	0.05421	0.05402	0.08914

MOTIF ACACGCCGGC

0.6635	0.1376	0.1377	0.06117
0.08251	0.7192	0.1105	0.08785
0.7188	0.09063	0.07802	0.1126
0.08173	0.7749	0.07963	0.06371
0.1003	0.06213	0.7599	0.07759
0.05646	0.7672	0.1126	0.06374
0.0917	0.781	0.06886	0.05848
0.07744	0.1226	0.7348	0.06521
0.07966	0.0834	0.736	0.1009
0.09221	0.7719	0.05266	0.08324

MOTIF GCTCACGGCT

0.07802	0.07473	0.7627	0.08453
0.08397	0.7852	0.05318	0.07769
0.09604	0.07109	0.1306	0.7022
0.09505	0.6713	0.1298	0.1039
0.6845	0.07994	0.1179	0.1177
0.1025	0.6656	0.1507	0.08114
0.08745	0.1116	0.7054	0.0955
0.05662	0.09811	0.7056	0.1396
0.096	0.7377	0.09215	0.07412
0.1169	0.1114	0.07522	0.6965

MOTIF ACTTACGG

0.8286	0.03748	0.09629	0.03761
0.07941	0.7638	0.07211	0.08464
0.05549	0.06014	0.08165	0.8027
0.04015	0.07295	0.09928	0.7876
0.7471	0.06797	0.09891	0.08601
0.04432	0.8236	0.04824	0.08383
0.06348	0.07624	0.7956	0.06464
0.05697	0.1869	0.69	0.06618

MOTIF GCCGTAAT

0.07821	0.06856	0.8046	0.04863
0.03646	0.7769	0.08686	0.09978
0.0983	0.7523	0.05982	0.08957
0.09214	0.07543	0.7461	0.08632
0.1073	0.1492	0.1131	0.6304
0.7847	0.06915	0.08487	0.06129
0.8057	0.07901	0.04783	0.06751
0.06172	0.08278	0.1172	0.7383

MOTIF TCCACAGT

0.0805	0.06734	0.08976	0.7624
0.08227	0.7448	0.09816	0.07478
0.09697	0.778	0.05095	0.07412
0.6881	0.1026	0.09324	0.1161
0.07954	0.8054	0.07074	0.04431
0.7353	0.08417	0.07668	0.1038
0.09311	0.1497	0.6695	0.08773
0.166	0.1353	0.09316	0.6055

MOTIF TCATTCGT

0.0986	0.08448	0.0966	0.7203
0.06817	0.7609	0.1018	0.06915
0.7805	0.1081	0.06337	0.04803
0.06215	0.07044	0.08213	0.7853
0.04861	0.09597	0.0812	0.7742
0.07139	0.7336	0.09907	0.09597
0.09688	0.1066	0.6845	0.1121
0.09236	0.07665	0.07451	0.7565

MOTIF AGCTTCCC

0.7804	0.0469	0.09237	0.08035
0.09148	0.0854	0.7143	0.1088
0.07188	0.8018	0.05132	0.07501
0.07034	0.09211	0.07793	0.7596
0.05333	0.07558	0.1511	0.72
0.06192	0.7608	0.1005	0.07686
0.1186	0.6058	0.1731	0.1025
0.1287	0.6714	0.08569	0.1142

MOTIF GGCAATCC

0.08566	0.09474	0.6233	0.1963
0.07424	0.1111	0.746	0.06862
0.08648	0.763	0.08105	0.06946
0.7216	0.09757	0.1092	0.07162
0.7728	0.09692	0.08232	0.04796
0.1018	0.09222	0.08205	0.7239
0.07857	0.7886	0.06712	0.06567
0.1075	0.7461	0.06906	0.07728

MOTIF ACCTGGGA

0.7187	0.09704	0.09775	0.08647
0.1097	0.6442	0.154	0.09209
0.09697	0.7514	0.06123	0.09044
0.1052	0.0668	0.07544	0.7526
0.0327	0.07895	0.801	0.08731
0.1074	0.09261	0.7243	0.07575
0.1255	0.07524	0.7209	0.07835
0.7388	0.1048	0.08874	0.06765

MOTIF TGCAATCC

0.06935	0.07787	0.1065	0.7463
0.07387	0.1413	0.697	0.08789
0.1281	0.7241	0.08973	0.05806
0.7352	0.08777	0.1067	0.07035
0.7833	0.08245	0.07616	0.05804
0.09212	0.08379	0.1235	0.7006
0.06616	0.7772	0.08063	0.07603
0.06055	0.7597	0.06371	0.116

MOTIF CCATCGCA

0.1237	0.7291	0.08055	0.06656
0.08032	0.8023	0.06985	0.0475
0.7489	0.06909	0.07722	0.1048
0.07941	0.09502	0.1147	0.7108
0.107	0.6842	0.1087	0.1001
0.1197	0.1269	0.6387	0.1147
0.07527	0.7639	0.09838	0.06249
0.7449	0.1189	0.05972	0.07649

MOTIF TACGTTCT

0.04318	0.148	0.1333	0.6755
0.7251	0.08853	0.1005	0.08587
0.09933	0.6961	0.1049	0.09966
0.106	0.1065	0.7256	0.0619
0.07946	0.08583	0.09056	0.7441
0.08271	0.09102	0.07448	0.7518
0.06699	0.7588	0.1017	0.07255
0.07253	0.07646	0.06396	0.7871

MOTIF TATTCGGA

0.1187	0.1053	0.1353	0.6407
0.6677	0.137	0.11	0.08525
0.08753	0.08462	0.04455	0.7833
0.05813	0.09829	0.09076	0.7528
0.07674	0.6859	0.09774	0.1396
0.08006	0.08243	0.7183	0.1192
0.0827	0.06977	0.7833	0.06425
0.7821	0.07877	0.06664	0.07253

MOTIF ACGTTC

0.7902	0.08667	0.06503	0.05811
0.1007	0.7091	0.1195	0.07071
0.08273	0.1014	0.7528	0.06304
0.09119	0.05364	0.1003	0.7549
0.08091	0.07403	0.07838	0.7667
0.04889	0.8356	0.06309	0.05244

MOTIF CTGGGA

0.09114	0.7988	0.05123	0.05886
0.071	0.09518	0.05767	0.7761
0.04194	0.06418	0.804	0.08992
0.08855	0.05677	0.7968	0.05785
0.1005	0.06284	0.7637	0.07289
0.7559	0.09294	0.08493	0.06628

MOTIF GCGTAG

0.07221	0.04879	0.8207	0.05828
0.07293	0.7906	0.07177	0.06475
0.08296	0.04429	0.8079	0.06481
0.107	0.1069	0.1106	0.6755
0.7433	0.1004	0.08923	0.06709
0.1463	0.05417	0.7448	0.05465

MOTIF CGGATT

0.08991	0.7268	0.07743	0.1059
0.08116	0.08281	0.7275	0.1085
0.08534	0.04543	0.8232	0.04608
0.7839	0.0703	0.09051	0.05532
0.09036	0.05567	0.08752	0.7664
0.03955	0.09546	0.1081	0.7569

MOTIF TAACGC

0.07632	0.08849	0.08223	0.753
0.6362	0.07309	0.09848	0.1922
0.7925	0.07306	0.05707	0.07736
0.08366	0.7263	0.07431	0.1157
0.07428	0.1188	0.7372	0.0697
0.06335	0.8001	0.07588	0.06071

MOTIF GGTAAG

0.06361	0.08263	0.7761	0.07765
0.08564	0.07664	0.7811	0.05661
0.09848	0.1192	0.1335	0.6488
0.7269	0.09157	0.09233	0.08922
0.7562	0.06558	0.07479	0.1034
0.0544	0.03391	0.8263	0.08542

MOTIF CATTCG

0.07545	0.783	0.07658	0.06499
0.8053	0.1055	0.04289	0.04632
0.05203	0.06975	0.05867	0.8196
0.05269	0.09998	0.06751	0.7798
0.07109	0.767	0.07306	0.08889
0.06136	0.07742	0.7409	0.1203

MOTIF AAGCGA

0.7537	0.1178	0.08057	0.04791
0.8022	0.05059	0.08807	0.05912
0.07401	0.08818	0.7622	0.07561
0.1002	0.7339	0.07814	0.08775
0.124	0.1056	0.6768	0.09361
0.7737	0.08732	0.06597	0.07304

MOTIF CAGGGG

0.08543	0.7998	0.07282	0.04193
0.8053	0.0465	0.05823	0.09
0.0797	0.06327	0.7947	0.0623
0.09782	0.06538	0.759	0.07784
0.08188	0.09239	0.7725	0.0532
0.07276	0.09903	0.7542	0.07406

MOTIF GTGCGG

0.05389	0.05961	0.8044	0.08212
0.0817	0.05901	0.1233	0.736
0.06483	0.06939	0.784	0.08179
0.08438	0.727	0.06923	0.1194
0.07773	0.07812	0.7616	0.0826
0.06489	0.05649	0.7881	0.09052

MOTIF ATCGGC

0.7851	0.05023	0.0582	0.1065
0.06792	0.06984	0.0816	0.7806
0.04692	0.7823	0.083	0.08774
0.1451	0.08712	0.6755	0.09236
0.09558	0.08397	0.7569	0.06354
0.06286	0.8422	0.05274	0.0422

MOTIF TTCGGA

0.06477	0.08321	0.05193	0.8001

0.0476	0.03002	0.08927	0.8331
0.05894	0.7576	0.07068	0.1128
0.1028	0.09292	0.6976	0.1067
0.02238	0.04716	0.8699	0.06052
0.7644	0.1132	0.04607	0.07635

MOTIF CCGTAA

0.05982	0.6956	0.1716	0.07301
0.08925	0.7613	0.0721	0.07733
0.1152	0.08869	0.7215	0.07454
0.07785	0.09724	0.08309	0.7418
0.7431	0.09325	0.09919	0.06444
0.812	0.0788	0.05386	0.05535

MOTIF ACGCTA

0.8243	0.0729	0.05326	0.04955
0.1012	0.743	0.0605	0.09533
0.09228	0.07821	0.768	0.06151
0.03706	0.829	0.07984	0.05406
0.06956	0.1129	0.04529	0.7723
0.7469	0.06304	0.1176	0.07246

MOTIF ACGCCC

0.7644	0.1176	0.05599	0.06203
0.0878	0.7985	0.05121	0.06249
0.1055	0.06186	0.766	0.06668
0.06579	0.7762	0.09264	0.06539
0.08475	0.8105	0.05459	0.05017
0.08085	0.7228	0.122	0.07438

MOTIF CTGAGG

0.07092	0.7537	0.06991	0.1054
0.09029	0.04271	0.07729	0.7897
0.03942	0.09797	0.8318	0.03083
0.7653	0.04881	0.08208	0.1038
0.07969	0.07788	0.7579	0.08449
0.08349	0.0883	0.7531	0.07507

MOTIF GTGGGT

0.08463	0.07614	0.7574	0.0818
0.08142	0.08147	0.06593	0.7712
0.05567	0.05679	0.8128	0.07473
0.07687	0.07978	0.7217	0.1217
0.1059	0.08989	0.7188	0.08535
0.05744	0.07335	0.0716	0.7976

MOTIF CGCCTCTCGT

0.05928	0.7706	0.09052	0.07961
0.08951	0.08576	0.7514	0.07336
0.05219	0.8304	0.07642	0.041

0.03859	0.7784	0.06086	0.1221
0.06591	0.1014	0.1962	0.6365
0.09538	0.7344	0.1003	0.06996
0.04643	0.08827	0.07997	0.7853
0.0646	0.7677	0.07748	0.09025
0.06775	0.09065	0.7539	0.0877
0.06382	0.1317	0.08362	0.7208

MOTIF ATGGGGTAGA

0.7723	0.08129	0.085	0.0614
0.09072	0.07367	0.0843	0.7513
0.06875	0.04745	0.8015	0.08232
0.05532	0.1118	0.7647	0.06819
0.05994	0.059	0.8361	0.04495
0.06318	0.04984	0.7955	0.09153
0.1255	0.1244	0.09612	0.654
0.7013	0.06526	0.1575	0.07584
0.05857	0.09254	0.7868	0.06205
0.7352	0.09155	0.06516	0.108

MOTIF TGGTCGCCGT

0.08337	0.1002	0.1081	0.7083
0.0421	0.158	0.7287	0.07111
0.06061	0.1053	0.7777	0.05642
0.06669	0.09916	0.0698	0.7644
0.05103	0.7848	0.07957	0.08457
0.06584	0.112	0.7687	0.05347
0.02785	0.7918	0.08865	0.09172
0.04726	0.7951	0.07302	0.0846
0.1146	0.1063	0.7213	0.05785
0.0976	0.1747	0.1368	0.5908

MOTIF CGGCGACC

0.04394	0.8138	0.06041	0.08185
0.07019	0.07038	0.8134	0.04602
0.07972	0.07883	0.8107	0.03075
0.05145	0.815	0.07342	0.06012
0.06057	0.08697	0.8044	0.04805
0.6671	0.07921	0.1723	0.08134
0.04591	0.7912	0.09916	0.06372
0.06555	0.7105	0.173	0.05089

MOTIF GGGCGTCT

0.05011	0.1304	0.7468	0.07274
0.0527	0.09893	0.8057	0.0427
0.02482	0.06246	0.8434	0.06932
0.05372	0.8047	0.1001	0.04151
0.06754	0.06657	0.7977	0.06818
0.08028	0.08852	0.162	0.6692
0.0542	0.7416	0.09907	0.1051

0.09006	0.07615	0.1965	0.6373

MOTIF CCGAATCT
0.07584	0.702	0.138	0.08414
0.09413	0.7514	0.06706	0.08744
0.0904	0.1179	0.7081	0.08361
0.6345	0.1484	0.1299	0.08717
0.608	0.1612	0.1158	0.1151
0.09166	0.08971	0.1484	0.6703
0.06564	0.7989	0.08454	0.05094
0.05699	0.2047	0.1391	0.5991

MOTIF AAGCGAAC
0.7014	0.1155	0.1204	0.06263
0.8039	0.09469	0.05635	0.04506
0.05666	0.08511	0.8008	0.0574
0.0782	0.7791	0.07959	0.06308
0.1093	0.112	0.7136	0.06512
0.7637	0.09254	0.07047	0.07328
0.6833	0.123	0.1201	0.07354
0.05759	0.7813	0.1056	0.05545

MOTIF TCGATCCT
0.05731	0.1511	0.1501	0.6415
0.04458	0.8318	0.0644	0.05918
0.08292	0.09609	0.7327	0.08826
0.6852	0.1952	0.051	0.06866
0.04685	0.08174	0.07798	0.7934
0.05203	0.7677	0.1146	0.06572
0.07634	0.8154	0.04943	0.05885
0.0898	0.07948	0.1072	0.7235

MOTIF CCACAGCC
0.06405	0.7915	0.07981	0.0646
0.0688	0.7975	0.08189	0.05182
0.566	0.1564	0.1517	0.1258
0.09797	0.7549	0.07511	0.07199
0.5312	0.2398	0.1136	0.1153
0.07619	0.09668	0.7557	0.0714
0.04381	0.8117	0.08879	0.05567
0.07452	0.7648	0.06066	0.09999

MOTIF CAGCGTCG
0.0443	0.8087	0.1012	0.04574
0.5874	0.1177	0.2375	0.05745
0.02624	0.06354	0.8147	0.09557
0.04457	0.789	0.09474	0.0717
0.09295	0.09918	0.7554	0.05251
0.1187	0.08612	0.2155	0.5797
0.03588	0.8548	0.05674	0.0526

0.06119 0.07613 0.7961 0.06654

MOTIF CGCTTACC
0.07944 0.7611 0.06858 0.0909
0.05179 0.1329 0.7335 0.08176
0.07234 0.784 0.09394 0.04977
0.08408 0.1041 0.07677 0.735
0.08012 0.1432 0.08755 0.6891
0.5798 0.192 0.1144 0.1138
0.03788 0.8101 0.07171 0.0803
0.09138 0.7801 0.05909 0.06938

MOTIF CCGATCCT
0.0565 0.6766 0.1485 0.1184
0.07462 0.7946 0.05837 0.07241
0.1155 0.1117 0.6724 0.1004
0.7282 0.1547 0.05798 0.05918
0.0403 0.113 0.09868 0.748
0.05693 0.7826 0.09085 0.06961
0.06416 0.7521 0.07357 0.1101
0.1031 0.1167 0.06487 0.7153

MOTIF TCCTTAGG
0.08498 0.1212 0.1407 0.6531
0.03807 0.7804 0.1147 0.06687
0.06769 0.8377 0.0569 0.03775
0.07229 0.1235 0.07545 0.7288
0.08899 0.1438 0.1081 0.6591
0.6783 0.1037 0.1327 0.0853
0.04834 0.09764 0.7857 0.0683
0.09641 0.09043 0.7341 0.07906

MOTIF AGGTCTCC
0.6699 0.1059 0.09462 0.1296
0.09263 0.1003 0.7411 0.06593
0.137 0.08587 0.6986 0.07858
0.05206 0.1652 0.07292 0.7099
0.0427 0.8104 0.07697 0.06993
0.06954 0.1166 0.1894 0.6245
0.04055 0.7737 0.09773 0.08798
0.05334 0.8164 0.08096 0.04928

MOTIF ATACAGGA
0.7406 0.08229 0.0829 0.09423
0.09009 0.1212 0.1436 0.6451
0.6163 0.1463 0.184 0.05345
0.06448 0.6916 0.1786 0.06529
0.7742 0.08931 0.07545 0.06104
0.06832 0.08556 0.7069 0.1392
0.05631 0.1264 0.776 0.04135

0.6251	0.1665	0.1293	0.07908

MOTIF TAACGC

0.1451	0.1494	0.1106	0.5949
0.7116	0.1184	0.122	0.04796
0.7136	0.1091	0.1044	0.07287
0.08004	0.6863	0.1693	0.06438
0.06827	0.09886	0.79	0.04285
0.07139	0.8049	0.06299	0.06069

MOTIF GACGCC

0.07821	0.07967	0.8086	0.03354
0.6342	0.2145	0.02799	0.1234
0.05549	0.8032	0.09337	0.04797
0.04487	0.0518	0.8706	0.03273
0.07753	0.8536	0.06153	0.007311
0.04225	0.8336	0.0701	0.05402

MOTIF GCGACC

0.06141	0.06616	0.8209	0.05149
0.05959	0.8227	0.04612	0.07159
0.07844	0.1126	0.77	0.039
0.7291	0.1016	0.1017	0.06761
0.06395	0.7847	0.08222	0.06911
0.06657	0.7521	0.1455	0.03578

MOTIF CCAGCG

0.05476	0.8136	0.08922	0.04238
0.06015	0.8062	0.0872	0.04649
0.7326	0.09418	0.1094	0.06386
0.04168	0.08419	0.8156	0.05849
0.02949	0.8651	0.0416	0.0638
0.07956	0.0918	0.7634	0.06528

MOTIF ATTCGG

0.7129	0.1364	0.08048	0.07018
0.08863	0.1032	0.1235	0.6847
0.0792	0.0943	0.1208	0.7057
0.06715	0.6982	0.1454	0.08925
0.07094	0.0548	0.8082	0.06603
0.07541	0.08085	0.7846	0.05914

MOTIF GCAGAT

0.05518	0.08429	0.8129	0.04767
0.06198	0.7755	0.1144	0.04816
0.7503	0.101	0.07374	0.07488
0.06026	0.07757	0.8085	0.05364
0.7607	0.1271	0.05393	0.05822
0.07562	0.07477	0.1103	0.7393

MOTIF AGGGCG

0.6945	0.1269	0.0895	0.08913
0.06463	0.1186	0.7336	0.08321
0.05702	0.06579	0.8359	0.04134
0.0654	0.07891	0.8001	0.05561
0.09071	0.7625	0.07726	0.06956
0.07526	0.05791	0.829	0.0378

MOTIF GCCTGT

0.04678	0.0591	0.834	0.06014
0.03157	0.8219	0.1137	0.03283
0.05521	0.7643	0.07198	0.1085
0.08005	0.07527	0.07499	0.7697
0.07321	0.1128	0.7321	0.08192
0.09233	0.09278	0.08474	0.7302

MOTIF GCGAGT

0.07819	0.0646	0.7798	0.07738
0.05346	0.8068	0.07363	0.06612
0.05383	0.09079	0.7997	0.05566
0.7669	0.07536	0.0871	0.07069
0.04651	0.05825	0.8097	0.08559
0.0685	0.1287	0.1836	0.6192

MOTIF GATCGG

0.0464	0.06955	0.8227	0.06133
0.7842	0.09129	0.08641	0.03814
0.05257	0.05832	0.1222	0.7669
0.06221	0.7072	0.1448	0.0858
0.0702	0.0656	0.7832	0.08099
0.09392	0.08367	0.7621	0.06028

MOTIF TCGCTT

0.04201	0.08565	0.09487	0.7775
0.03919	0.8214	0.08311	0.05633
0.05277	0.09393	0.7827	0.07059
0.08651	0.7851	0.07277	0.05566
0.05825	0.07026	0.08769	0.7838
0.04137	0.1188	0.1145	0.7253

MOTIF TCGATG

0.06955	0.06637	0.09054	0.7735
0.05319	0.7702	0.1003	0.07639
0.04356	0.1153	0.8057	0.03543
0.7651	0.1013	0.07105	0.06259
0.06927	0.07904	0.09814	0.7536
0.04951	0.09492	0.7826	0.073

MOTIF TAAGCC

0.1013	0.1457	0.1129	0.6401

0.6384	0.1956	0.1037	0.06232
0.7414	0.07442	0.111	0.07312
0.06014	0.07396	0.7919	0.07401
0.04678	0.7988	0.1238	0.03064
0.08882	0.7706	0.07887	0.06173

MOTIF GCCTTA

0.05515	0.1478	0.6875	0.1095
0.02837	0.8275	0.09963	0.04447
0.04525	0.8139	0.08626	0.05461
0.07422	0.07566	0.05957	0.7905
0.08997	0.1006	0.1209	0.6885
0.6804	0.1085	0.1038	0.1073

MOTIF AAACGC

0.7319	0.1065	0.08258	0.07902
0.7222	0.09069	0.1333	0.05388
0.6992	0.07647	0.127	0.09735
0.07947	0.7328	0.117	0.07074
0.06173	0.1199	0.7902	0.02822
0.08118	0.8044	0.09058	0.02381

MOTIF GATCAG

0.06679	0.05924	0.8229	0.05106
0.785	0.09929	0.07494	0.04081
0.06236	0.09379	0.1672	0.6767
0.04139	0.7696	0.1153	0.07367
0.659	0.08561	0.1505	0.1048
0.08074	0.0885	0.7402	0.09052

MOTIF CCCCAT

0.1048	0.7706	0.05788	0.06668
0.06513	0.7589	0.1013	0.07465
0.07237	0.809	0.05973	0.05888
0.06142	0.8258	0.05858	0.05416
0.7445	0.08677	0.08772	0.08105
0.08161	0.1106	0.07596	0.7318

MOTIF GTCTCC

0.09544	0.09545	0.7309	0.07816
0.04471	0.1203	0.05398	0.781
0.05395	0.7889	0.07116	0.086
0.0743	0.1	0.1751	0.6506
0.03765	0.8321	0.06453	0.06569
0.03567	0.8404	0.0646	0.05929

MOTIF GCGGTG

0.06215	0.04176	0.8397	0.0564
0.06545	0.7652	0.07951	0.08982
0.07502	0.07228	0.7823	0.07037

0.06578	0.09246	0.7919	0.04986
0.0993	0.2304	0.1026	0.5676
0.03947	0.05659	0.8649	0.03903

MOTIF AGGATT

0.7349	0.0689	0.07371	0.1224
0.02692	0.06972	0.8166	0.08675
0.04127	0.08047	0.8498	0.02847
0.6802	0.1444	0.1281	0.04728
0.06911	0.04493	0.08642	0.7995
0.0808	0.1562	0.08827	0.6747

MOTIF ACAAGCTGAT

0.7774	0.04611	0.08238	0.0941
0.07699	0.8156	0.06394	0.04345
0.7983	0.09877	0.04937	0.05358
0.8356	0.04515	0.03974	0.07954
0.1163	0.06065	0.7415	0.08149
0.1077	0.6558	0.09046	0.146
0.09008	0.04414	0.03683	0.829
0.09314	0.1171	0.7196	0.07024
0.8185	0.06932	0.04498	0.06724
0.05776	0.05123	0.07089	0.8201

MOTIF GTCTCCAGGG

0.04782	0.03439	0.8442	0.07358
0.06702	0.07144	0.05134	0.8102
0.05818	0.8108	0.06052	0.07054
0.08208	0.04327	0.07612	0.7985
0.03502	0.8289	0.05206	0.08397
0.05863	0.7921	0.041	0.1083
0.8314	0.04967	0.04401	0.07493
0.07537	0.07414	0.7378	0.1127
0.04739	0.08638	0.7768	0.08939
0.0466	0.04502	0.8411	0.06723

MOTIF CCTCTGGCAG

0.0784	0.7671	0.07085	0.08366
0.1087	0.7441	0.07226	0.07488
0.09322	0.1021	0.09438	0.7103
0.1216	0.6971	0.06272	0.1187
0.06978	0.05936	0.07049	0.8004
0.06712	0.07924	0.7395	0.1141
0.061	0.08928	0.747	0.1027
0.06427	0.8079	0.07696	0.05089
0.802	0.07191	0.06819	0.0579
0.1811	0.06216	0.6928	0.06394

MOTIF GACTAAAACC

0.1047	0.05861	0.7622	0.07453

0.8012	0.06293	0.07714	0.05877
0.09488	0.7778	0.06611	0.0612
0.08311	0.07972	0.06158	0.7756
0.7994	0.0375	0.1054	0.05771
0.844	0.06299	0.04148	0.05158
0.7471	0.1115	0.09141	0.04999
0.784	0.08886	0.05076	0.07636
0.1484	0.6902	0.05479	0.1067
0.1113	0.7413	0.05406	0.09328

MOTIF ATTACCCG

0.7399	0.04675	0.08785	0.1255
0.06779	0.05831	0.04589	0.828
0.07942	0.07988	0.1111	0.7296
0.7431	0.08263	0.1221	0.0521
0.07503	0.7766	0.08152	0.06683
0.1077	0.7485	0.05785	0.08596
0.1613	0.6534	0.06314	0.1222
0.1131	0.09613	0.6589	0.1319

MOTIF GATCGCCA

0.09288	0.1045	0.7042	0.0984
0.8098	0.06325	0.05459	0.07241
0.04139	0.0607	0.06625	0.8317
0.09859	0.6926	0.1084	0.1004
0.1299	0.0484	0.7295	0.0922
0.06125	0.7853	0.07287	0.0806
0.08246	0.7768	0.0667	0.07408
0.7659	0.07929	0.06659	0.08819

MOTIF GTTACCAG

0.1036	0.05503	0.7211	0.1203
0.09931	0.04935	0.07626	0.7751
0.08852	0.1147	0.08753	0.7092
0.7618	0.0511	0.1007	0.0864
0.09104	0.7189	0.07254	0.1175
0.1245	0.7666	0.03911	0.06974
0.7954	0.05627	0.07241	0.07597
0.1274	0.07526	0.6726	0.1248

MOTIF AGGGTGAC

0.7886	0.07735	0.06525	0.06882
0.1143	0.06474	0.6432	0.1777
0.09377	0.05525	0.7637	0.08726
0.07298	0.07309	0.7303	0.1236
0.06747	0.0611	0.07399	0.7974
0.08449	0.06454	0.7612	0.08981
0.7014	0.07191	0.1184	0.1082
0.06557	0.7291	0.1142	0.09107

MOTIF TGACCAAA

0.08127	0.09202	0.08239	0.7443
0.1074	0.07358	0.7365	0.08251
0.7866	0.05694	0.07889	0.07756
0.1319	0.684	0.0773	0.1068
0.111	0.7349	0.04622	0.1078
0.7867	0.06825	0.05746	0.08763
0.7867	0.04386	0.08839	0.08106
0.7834	0.0752	0.08081	0.06062

MOTIF CTCCTGAT

0.06685	0.759	0.07774	0.0964
0.1096	0.05857	0.05066	0.7812
0.08348	0.6732	0.1074	0.1359
0.1013	0.7406	0.05903	0.09905
0.09812	0.04273	0.03673	0.8224
0.06821	0.08786	0.7547	0.08926
0.7175	0.1221	0.06284	0.09757
0.0591	0.05972	0.05992	0.8213

MOTIF ATAAGTAG

0.8131	0.06458	0.07105	0.05129
0.09895	0.1666	0.08062	0.6538
0.7328	0.0977	0.09146	0.07807
0.8338	0.05341	0.04971	0.06308
0.08113	0.09899	0.7184	0.1015
0.07671	0.06743	0.1067	0.7491
0.7439	0.06453	0.08891	0.1027
0.1178	0.09239	0.716	0.07376

MOTIF CTCAGCTG

0.1399	0.6713	0.1217	0.06708
0.07162	0.1044	0.09392	0.73
0.04029	0.8143	0.09159	0.05384
0.7431	0.08731	0.04415	0.1254
0.0617	0.114	0.7242	0.1001
0.1159	0.7066	0.09809	0.07949
0.08907	0.05595	0.02925	0.8257
0.08192	0.1341	0.6976	0.08638

MOTIF TTCAGTCT

0.08916	0.09696	0.1028	0.7111
0.06697	0.08487	0.09684	0.7513
0.0908	0.6348	0.12	0.1544
0.7788	0.07472	0.04697	0.0995
0.08103	0.06533	0.7554	0.09823
0.0758	0.0656	0.07164	0.787
0.04866	0.7483	0.0705	0.1325
0.1374	0.08349	0.08133	0.6978

MOTIF AGTCTC

0.7788	0.07201	0.06097	0.08825
0.08455	0.05793	0.756	0.1015
0.09028	0.0664	0.05611	0.7872
0.0603	0.8021	0.05627	0.08132
0.08612	0.06758	0.04362	0.8027
0.06097	0.7518	0.09035	0.09687

MOTIF ATCGCC

0.7226	0.09842	0.07258	0.1064
0.04259	0.06515	0.05137	0.8409
0.04529	0.7356	0.1133	0.1058
0.1192	0.05062	0.7291	0.1012
0.05313	0.8263	0.04863	0.07195
0.07906	0.8127	0.04868	0.05958

MOTIF TACCAG

0.08276	0.07175	0.1018	0.7437
0.7653	0.053	0.1112	0.07048
0.07936	0.7647	0.07534	0.08059
0.1225	0.7769	0.04093	0.05966
0.8447	0.06143	0.03431	0.05954
0.09131	0.05849	0.7525	0.09774

MOTIF TGACCT

0.03234	0.04565	0.04444	0.8776
0.07097	0.09522	0.7795	0.05434
0.7996	0.05554	0.06725	0.07761
0.08443	0.7439	0.07547	0.09623
0.101	0.7245	0.03725	0.1372
0.08899	0.0531	0.06011	0.7978

MOTIF GTAACC

0.0922	0.08626	0.753	0.06849
0.09162	0.09098	0.04323	0.7742
0.7028	0.1032	0.1152	0.07884
0.8351	0.07717	0.05336	0.03436
0.0682	0.8018	0.04305	0.08695
0.09409	0.7476	0.05538	0.1029

MOTIF ACTTGT

0.773	0.06382	0.05783	0.1053
0.07669	0.7884	0.051	0.08389
0.02958	0.05534	0.04436	0.8707
0.01318	0.05392	0.04861	0.8843
0.09876	0.05584	0.7692	0.07622
0.07362	0.08423	0.09052	0.7516

MOTIF TCAGCA

0.05505	0.09626	0.08145	0.7672

0.0862	0.7536	0.07047	0.08971
0.8614	0.02645	0.03709	0.07502
0.09288	0.08715	0.7192	0.1007
0.08179	0.7846	0.0764	0.05722
0.7817	0.05758	0.04273	0.118

MOTIF AGGGTA

0.7793	0.06789	0.08139	0.07139
0.0761	0.04419	0.7693	0.1104
0.1018	0.03659	0.7729	0.0887
0.09499	0.05448	0.7599	0.0906
0.1137	0.06693	0.08968	0.7296
0.7497	0.04799	0.1199	0.0824

MOTIF ACAGAT

0.7744	0.07929	0.05485	0.09142
0.0933	0.782	0.08482	0.03988
0.8395	0.05025	0.02788	0.08243
0.08361	0.1007	0.7687	0.04697
0.8141	0.07809	0.04758	0.06023
0.07308	0.08385	0.07889	0.7642

MOTIF CTGGCA

0.07875	0.7261	0.06035	0.1348
0.07124	0.04605	0.05346	0.8292
0.05828	0.04533	0.7873	0.1091
0.1037	0.07719	0.7014	0.1177
0.06632	0.7661	0.05168	0.1159
0.8062	0.0608	0.08244	0.05051

MOTIF CTGGAG

0.07718	0.7623	0.0925	0.068
0.1181	0.06802	0.03686	0.777
0.1131	0.01769	0.7819	0.0873
0.08052	0.02374	0.8485	0.04721
0.7526	0.09443	0.07971	0.07325
0.07497	0.08008	0.7399	0.1051

MOTIF GAGCGG

0.06637	0.06519	0.7898	0.07864
0.8362	0.04487	0.07856	0.04035
0.06477	0.06431	0.8068	0.06415
0.1003	0.7346	0.08293	0.08218
0.1324	0.09131	0.6158	0.1605
0.1007	0.05087	0.7863	0.06211

MOTIF TGATCG

0.0769	0.03154	0.03816	0.8534
0.06686	0.08248	0.767	0.08367
0.7414	0.07935	0.0998	0.07946

0.0532	0.07816	0.05441	0.8142
0.0771	0.6754	0.1467	0.1007
0.05617	0.09227	0.7199	0.1317

MOTIF TTAAGC

0.07385	0.09315	0.04588	0.7871
0.06615	0.08841	0.1128	0.7326
0.755	0.08774	0.07169	0.08552
0.7887	0.05666	0.07497	0.07963
0.06059	0.07205	0.7287	0.1386
0.06653	0.7208	0.1263	0.08639

MOTIF ATTCGC

0.7541	0.08377	0.1037	0.05841
0.08224	0.08015	0.06877	0.7688
0.07627	0.05447	0.06758	0.8017
0.1236	0.6652	0.09592	0.1153
0.1261	0.07928	0.6396	0.1551
0.07236	0.7597	0.09359	0.07439

MOTIF TACCGC

0.08427	0.1182	0.07419	0.7233
0.6409	0.08243	0.1707	0.106
0.08292	0.7126	0.08375	0.1207
0.1071	0.7052	0.09266	0.09507
0.1271	0.1513	0.6106	0.111
0.06251	0.7756	0.07317	0.08869

MOTIF TGACAG

0.08497	0.06684	0.0676	0.7806
0.07376	0.08419	0.8007	0.04139
0.7292	0.0982	0.0728	0.0998
0.06816	0.8107	0.06462	0.05651
0.8297	0.05675	0.0445	0.06907
0.125	0.08246	0.6947	0.09778

MOTIF TTATCG

0.05464	0.03751	0.06465	0.8432
0.07629	0.09474	0.09135	0.7376
0.7956	0.08792	0.05497	0.06146
0.04926	0.05958	0.07966	0.8115
0.05751	0.694	0.1542	0.09428
0.1139	0.1079	0.6331	0.1451

MOTIF GGAAGT

0.0787	0.04953	0.7874	0.08434
0.07273	0.1137	0.7305	0.08313
0.7566	0.09428	0.08186	0.06725
0.7857	0.06092	0.07389	0.07953
0.0748	0.105	0.7433	0.07695

0.13	0.07358	0.1066	0.6898

MOTIF AGTGTCCGTG

0.7855	0.1029	0.04573	0.0659
0.09124	0.06076	0.799	0.04904
0.1035	0.07849	0.07259	0.7454
0.03398	0.178	0.7358	0.0522
0.06759	0.07215	0.08881	0.7715
0.04957	0.7465	0.09609	0.1079
0.06625	0.7929	0.06969	0.07117
0.06624	0.06637	0.768	0.09935
0.07502	0.07185	0.05309	0.8
0.05578	0.0951	0.8149	0.03418

MOTIF AGTTGTCACG

0.7904	0.04026	0.08651	0.08287
0.05851	0.08981	0.7803	0.07134
0.05959	0.06032	0.07661	0.8035
0.04719	0.0483	0.06007	0.8444
0.05042	0.07671	0.8086	0.06427
0.03884	0.03645	0.08953	0.8352
0.04338	0.8063	0.1092	0.04112
0.8003	0.0634	0.04586	0.09043
0.1161	0.7008	0.0627	0.1204
0.0751	0.05681	0.7505	0.1176

MOTIF CGGTAGCAAG

0.08821	0.7064	0.09791	0.1075
0.08284	0.06968	0.7952	0.0523
0.1167	0.1032	0.6752	0.1049
0.1448	0.1128	0.07235	0.67
0.7634	0.04786	0.0907	0.09807
0.09441	0.04428	0.814	0.04734
0.09446	0.7882	0.06453	0.05285
0.7669	0.06652	0.0731	0.09352
0.7656	0.05941	0.06355	0.1114
0.08491	0.06306	0.7054	0.1466

MOTIF CAATCTCCGT

0.08206	0.7771	0.08634	0.05454
0.68	0.1542	0.06403	0.1018
0.7498	0.07628	0.1038	0.0701
0.08665	0.1602	0.0769	0.6763
0.04322	0.776	0.09533	0.08543
0.05622	0.06946	0.06152	0.8128
0.05468	0.8016	0.06535	0.07837
0.06488	0.8125	0.04745	0.07517
0.124	0.06129	0.7416	0.0731
0.05932	0.1265	0.04449	0.7696

MOTIF GGCCTTAA

0.06437	0.05745	0.8114	0.06675
0.07542	0.06172	0.8147	0.04818
0.06357	0.7733	0.1053	0.05779
0.06779	0.7945	0.07114	0.06654
0.1002	0.09126	0.09222	0.7163
0.05662	0.1699	0.1094	0.6641
0.66	0.09669	0.1441	0.09928
0.5543	0.08029	0.274	0.09139

MOTIF GCCCGCAC

0.04908	0.0908	0.7863	0.07386
0.06393	0.7961	0.09835	0.04164
0.07675	0.7976	0.07267	0.05298
0.09388	0.6612	0.1526	0.09231
0.08812	0.0577	0.7804	0.07381
0.09811	0.7606	0.06792	0.07341
0.6004	0.2015	0.1129	0.08514
0.05085	0.7612	0.09509	0.09286

MOTIF GTTGCGAT

0.06674	0.1091	0.7483	0.07587
0.07242	0.09937	0.06908	0.7591
0.06291	0.048	0.08816	0.8009
0.04833	0.1092	0.8007	0.04175
0.08627	0.661	0.07945	0.1733
0.09459	0.09283	0.7459	0.06668
0.757	0.07976	0.09928	0.06396
0.06572	0.04182	0.08723	0.8052

MOTIF GAACTCAG

0.1012	0.08463	0.7426	0.07157
0.6821	0.105	0.1357	0.07717
0.7482	0.1144	0.06787	0.0695
0.07313	0.7339	0.1115	0.08144
0.1231	0.06483	0.09958	0.7125
0.0478	0.7943	0.0861	0.07179
0.7235	0.1036	0.08888	0.08406
0.1212	0.04578	0.7676	0.06534

MOTIF TCCGTGTC

0.06812	0.1014	0.08181	0.7487
0.06372	0.7753	0.1051	0.05584
0.06646	0.8095	0.06656	0.05746
0.1285	0.08388	0.6929	0.09467
0.08857	0.09695	0.07499	0.7395
0.07137	0.1129	0.7336	0.08217
0.08515	0.1348	0.1059	0.6742
0.06232	0.7862	0.0809	0.07057

MOTIF GGCCTGAC

0.1079	0.1515	0.6627	0.07787
0.04546	0.08015	0.7986	0.07584
0.08267	0.7729	0.08089	0.06357
0.1051	0.7736	0.07187	0.0495
0.07577	0.1793	0.08844	0.6565
0.06533	0.126	0.7288	0.07984
0.7284	0.09374	0.1026	0.07528
0.06502	0.6989	0.1533	0.08277

MOTIF GCATTAGC

0.04956	0.08409	0.7958	0.07053
0.08997	0.788	0.05689	0.06514
0.6651	0.2061	0.07575	0.05304
0.06131	0.08813	0.09633	0.7542
0.06305	0.1727	0.1328	0.6315
0.7036	0.1256	0.1011	0.06965
0.059	0.05353	0.8121	0.07536
0.08248	0.7185	0.09715	0.1019

MOTIF GCCGATTG

0.08908	0.05748	0.7824	0.07107
0.08998	0.7507	0.09947	0.05981
0.0965	0.7168	0.07586	0.1109
0.09408	0.09078	0.7336	0.08152
0.6802	0.1527	0.07814	0.08899
0.112	0.1324	0.09802	0.6576
0.0716	0.07162	0.1721	0.6847
0.04963	0.07703	0.7881	0.08528

MOTIF AGCTGTGA

0.7228	0.06314	0.11	0.1041
0.05543	0.06787	0.8291	0.04759
0.07319	0.6674	0.09301	0.1664
0.09279	0.07169	0.06033	0.7752
0.07977	0.07992	0.784	0.05627
0.1058	0.07727	0.1	0.7169
0.04176	0.1122	0.788	0.05802
0.6305	0.106	0.1697	0.09379

MOTIF GCGTTA

0.0643	0.07517	0.8203	0.0402
0.05011	0.7954	0.07449	0.08
0.1091	0.1621	0.6553	0.07349
0.09616	0.05927	0.08655	0.758
0.07996	0.1003	0.1199	0.6998
0.7864	0.06611	0.08108	0.06636

MOTIF CGGCAC

0.06505	0.8443	0.03995	0.05067

0.07659	0.07973	0.7832	0.06049
0.05009	0.05451	0.8529	0.04246
0.05619	0.8298	0.04806	0.06595
0.7256	0.1245	0.07719	0.07265
0.08136	0.773	0.08965	0.05603

MOTIF ACCGAT

0.7221	0.07651	0.1147	0.08677
0.05862	0.8705	0.05183	0.01903
0.07142	0.7778	0.06998	0.08078
0.07764	0.1096	0.7531	0.05965
0.8063	0.08279	0.03708	0.0738
0.1062	0.1483	0.08412	0.6613

MOTIF TATCGC

0.09821	0.1246	0.05948	0.7177
0.7246	0.129	0.07996	0.06644
0.1119	0.09083	0.06171	0.7356
0.04151	0.738	0.1011	0.1194
0.09635	0.07626	0.7524	0.07495
0.06305	0.793	0.07588	0.06811

MOTIF CACGGA

0.05307	0.8372	0.05434	0.05542
0.7625	0.0565	0.08125	0.09971
0.08719	0.7312	0.07157	0.11
0.06017	0.05384	0.8236	0.06237
0.07122	0.07045	0.8064	0.05195
0.7224	0.1422	0.06958	0.06579

MOTIF GTACCG

0.09469	0.06384	0.779	0.06249
0.07795	0.09819	0.1015	0.7223
0.6375	0.07496	0.2033	0.08418
0.04423	0.8404	0.0477	0.06763
0.04218	0.7625	0.09757	0.09778
0.06903	0.0716	0.7816	0.07772

MOTIF TAAGGC

0.07609	0.1107	0.07301	0.7402
0.615	0.1425	0.1594	0.08308
0.7812	0.06922	0.07853	0.07103
0.06135	0.09196	0.7585	0.08817
0.04276	0.04707	0.8596	0.05052
0.06037	0.8491	0.05433	0.03616

MOTIF CAGGGC

0.04682	0.8427	0.05802	0.05246
0.7283	0.06685	0.09468	0.1102
0.06959	0.1187	0.7344	0.07726

0.0689	0.07581	0.7731	0.08214
0.07452	0.08195	0.7919	0.05165
0.05743	0.8346	0.05979	0.04822

MOTIF ATACCG

0.7181	0.07712	0.1306	0.07423
0.07989	0.1239	0.1126	0.6836
0.7429	0.07354	0.1104	0.07316
0.06245	0.807	0.0637	0.06685
0.08484	0.7588	0.09717	0.0592
0.07057	0.07309	0.7759	0.08048

MOTIF GGGGAC

0.08398	0.06614	0.7457	0.1042
0.08313	0.07853	0.7803	0.05803
0.1144	0.05917	0.7632	0.06323
0.05731	0.06327	0.8176	0.06178
0.7327	0.1036	0.08441	0.07934
0.06242	0.7714	0.09762	0.0686

MOTIF CCTGAC

0.07634	0.7851	0.08784	0.05073
0.0847	0.7785	0.07488	0.06195
0.05647	0.1104	0.05284	0.7803
0.05526	0.09075	0.8111	0.04285
0.7366	0.1103	0.06923	0.08385
0.06007	0.7401	0.1182	0.08167

MOTIF CACAGC

0.05851	0.8067	0.09083	0.04398
0.7785	0.07679	0.08569	0.05902
0.06932	0.8038	0.07808	0.04881
0.7081	0.06373	0.1216	0.1065
0.1007	0.0756	0.7686	0.05513
0.05447	0.8148	0.07743	0.05328

MOTIF CGCTTG

0.09381	0.7091	0.08007	0.117
0.06047	0.08115	0.7708	0.08753
0.05579	0.8088	0.07912	0.05629
0.1184	0.1155	0.09769	0.6683
0.06387	0.07928	0.09172	0.7651
0.04162	0.07631	0.8169	0.06517

MOTIF CGGAAC

0.07566	0.7726	0.06186	0.08985
0.08938	0.06967	0.7559	0.085
0.06285	0.07731	0.8191	0.04075
0.6522	0.1841	0.07025	0.09348
0.7198	0.06928	0.1333	0.07753

0.05785	0.7614	0.1232	0.05755

MOTIF AGTGCG

0.7429	0.07522	0.07996	0.1019
0.07954	0.07421	0.7705	0.07575
0.06172	0.06683	0.08409	0.7874
0.0623	0.09888	0.7389	0.09991
0.08863	0.7475	0.07903	0.08486
0.07601	0.09184	0.7588	0.07332

MOTIF GTCCTG

0.06417	0.09697	0.7512	0.08762
0.06011	0.1644	0.0974	0.6781
0.07812	0.7516	0.09684	0.07345
0.08245	0.7555	0.1085	0.0536
0.06409	0.06489	0.05317	0.8178
0.05358	0.07591	0.8021	0.06839

MOTIF GACCGTATCG

0.08574	0.09047	0.7453	0.07848
0.7705	0.0839	0.07116	0.07442
0.06666	0.7957	0.06555	0.07205
0.0945	0.7259	0.07996	0.09962
0.1047	0.07916	0.7161	0.1
0.08851	0.1065	0.1193	0.6857
0.6404	0.1184	0.1345	0.1067
0.0763	0.1012	0.07894	0.7436
0.08853	0.7344	0.0925	0.08454
0.09626	0.09855	0.6991	0.1061

MOTIF TGTAACGGCA

0.08456	0.05943	0.07761	0.7784
0.0868	0.09291	0.7442	0.07608
0.09975	0.1305	0.09207	0.6777
0.7551	0.07979	0.09713	0.06797
0.7655	0.07971	0.07865	0.0761
0.1228	0.6479	0.1086	0.1208
0.1231	0.07776	0.6834	0.1157
0.08315	0.08317	0.7398	0.09385
0.1179	0.7016	0.08342	0.09716
0.7629	0.07143	0.07339	0.09228

MOTIF TAGCGTTAGA

0.0901	0.07037	0.08334	0.7562
0.7724	0.05492	0.08119	0.09145
0.107	0.08664	0.7085	0.09784
0.142	0.5873	0.1264	0.1443
0.1263	0.09752	0.6286	0.1476
0.07998	0.06876	0.08385	0.7674
0.07094	0.06349	0.07201	0.7936

0.7358	0.06357	0.09096	0.1097
0.09567	0.07616	0.7439	0.08432
0.7734	0.07418	0.07858	0.07388

MOTIF GACGTTAC

0.08025	0.07884	0.7752	0.06573
0.8126	0.06442	0.0545	0.06846
0.1118	0.705	0.08222	0.101
0.08226	0.08379	0.7503	0.08365
0.06279	0.0721	0.09243	0.7727
0.06015	0.1034	0.0908	0.7457
0.7263	0.08947	0.1036	0.08067
0.07175	0.7806	0.07184	0.07582

MOTIF CGTTAT

0.05717	0.8295	0.04497	0.06841
0.05518	0.06439	0.8115	0.06889
0.04508	0.03305	0.04223	0.8796
0.0325	0.0464	0.03931	0.8818
0.8392	0.04314	0.06504	0.05261
0.04208	0.04667	0.04951	0.8617

MOTIF GACGTT

0.01392	0.0561	0.8898	0.04016
0.849	0.05448	0.03725	0.05924
0.07532	0.7857	0.06668	0.07231
0.0492	0.06014	0.8368	0.05385
0.04527	0.04257	0.08235	0.8298
0.03439	0.01703	0.05858	0.89

MOTIF CGGTAG

0.06006	0.8369	0.0457	0.0573
0.05861	0.04439	0.8345	0.06254
0.03142	0.03886	0.8851	0.04466
0.0596	0.0476	0.05059	0.8422
0.8113	0.06656	0.06844	0.05368
0.05255	0.04393	0.8673	0.03621

MOTIF ACCGTA

0.9083	0.03286	0.02934	0.0295
0.03705	0.9038	0.02658	0.03257
0.07742	0.809	0.04396	0.06964
0.07347	0.06581	0.7951	0.06558
0.05282	0.04171	0.08777	0.8177
0.8182	0.07674	0.05016	0.05486

MOTIF AACGTG

0.881	0.01665	0.05744	0.04494
0.8611	0.05156	0.04395	0.04335
0.05833	0.8151	0.07539	0.05116

0.08051	0.04733	0.7921	0.08009
0.0451	0.02539	0.05338	0.8761
0.03875	0.04146	0.9062	0.01354

MOTIF CGCTTA

0.074	0.8387	0.03999	0.04732
0.08541	0.1176	0.7502	0.04672
0.04426	0.807	0.07759	0.07114
0.03379	0.05356	0.05068	0.862
0.06745	0.04902	0.06498	0.8186
0.8088	0.0705	0.06916	0.05156

MOTIF CAATCG

0.05735	0.8192	0.07352	0.04998
0.8302	0.08035	0.02095	0.06847
0.8295	0.05005	0.08194	0.03846
0.1295	0.106	0.07103	0.6934
0.06435	0.8764	0.05139	0.007896
0.03769	0.04024	0.8759	0.0462

MOTIF ATTGCG

0.7466	0.0797	0.1035	0.07018
0.03682	0.08009	0.03886	0.8442
0.0735	0.04736	0.09047	0.7887
0.07363	0.05841	0.8098	0.05818
0.03401	0.8243	0.05293	0.08878
0.09142	0.0609	0.7985	0.04913

MOTIF TTATCG

0.05394	0.0425	0.04659	0.857
0.03329	0.04754	0.03819	0.881
0.8489	0.03768	0.06616	0.04728
0.05013	0.07004	0.04632	0.8335
0.08805	0.8232	0.05177	0.037
0.04476	0.04319	0.8558	0.05623

MOTIF ACCGTATC

0.8325	0.05551	0.04574	0.06628
0.062	0.8326	0.04234	0.06308
0.09554	0.7563	0.05559	0.09253
0.1047	0.08095	0.7089	0.1055
0.06681	0.07055	0.07576	0.7869
0.736	0.09262	0.08264	0.08871
0.06532	0.07572	0.0741	0.7849
0.07115	0.8014	0.05647	0.07099

MOTIF CTAACGTG

0.08218	0.7821	0.06837	0.06736
0.09146	0.08309	0.07165	0.7538
0.7847	0.06908	0.08899	0.05724

0.8043	0.06643	0.05927	0.07005
0.1149	0.7202	0.08351	0.08135
0.1169	0.06942	0.7004	0.1133
0.05971	0.0446	0.05494	0.8408
0.06011	0.06154	0.7934	0.08499

MOTIF AGCGTT

0.8642	0.03572	0.05219	0.04785
0.02044	0.08892	0.8284	0.06227
0.051	0.7929	0.08896	0.06713
0.04385	0.06459	0.8225	0.06907
0.02397	0.008239	0.05056	0.9172
0.03867	0.06027	0.06418	0.8369

MOTIF CTATCG

0.05689	0.836	0.04828	0.05881
0.03188	0.05783	0.01504	0.8952
0.8164	0.0572	0.07492	0.05147
0.06143	0.06732	0.05289	0.8184
0.02066	0.8829	0.04914	0.0473
0.06348	0.06226	0.8083	0.06592

MOTIF TACGGA

0.0879	0.06339	0.08294	0.7658
0.9035	0.01602	0.03687	0.04364
0.069	0.8182	0.05899	0.05384
0.08901	0.07229	0.7847	0.05402
0.03359	0.02873	0.9203	0.01736
0.8391	0.04364	0.04897	0.06832

MOTIF TATACCGC

0.08032	0.1024	0.08554	0.7317
0.6343	0.1397	0.101	0.125
0.07678	0.09472	0.0727	0.7558
0.8682	0.054	0.04618	0.03158
0.1047	0.7694	0.07036	0.05549
0.08424	0.7376	0.08338	0.09482
0.1027	0.05267	0.7548	0.08983
0.08927	0.6862	0.09547	0.129

MOTIF CGTCTG

0.05529	0.8523	0.04324	0.04919
0.02895	0.04008	0.889	0.042
0.05611	0.04132	0.03959	0.863
0.03173	0.8337	0.04974	0.08486
0.07876	0.08036	0.0983	0.7426
0.04182	0.07712	0.8253	0.05572

MOTIF TGCGAA

0.06163	0.06466	0.0674	0.8063

0.05686	0.0702	0.8517	0.02121
0.05003	0.8094	0.06904	0.07158
0.11	0.09299	0.6616	0.1354
0.8799	0.01313	0.06101	0.04594
0.84	0.06614	0.03923	0.05466

MOTIF GACTAC

0.0582	0.07109	0.8156	0.05509
0.8701	0.04172	0.05412	0.03403
0.02539	0.9045	0.03342	0.03669
0.0618	0.05988	0.1026	0.7757
0.8474	0.04097	0.05843	0.05317
0.06252	0.8371	0.04221	0.05821

MOTIF GATACG

0.03116	0.03173	0.899	0.03813
0.8515	0.05613	0.04929	0.04307
0.08265	0.07838	0.07455	0.7644
0.8691	0.05042	0.04042	0.04005
0.05988	0.8265	0.05415	0.05948
0.06483	0.03425	0.8546	0.04637

MOTIF TACAGG

0.05431	0.04219	0.05624	0.8473
0.8704	0.04301	0.05096	0.03558
0.06212	0.8233	0.06301	0.05156
0.8883	0.0427	0.03438	0.03465
0.05913	0.03698	0.8332	0.07068
0.05686	0.04521	0.8253	0.07265

MOTIF TGAACG

0.0546	0.05919	0.07194	0.8143
0.0539	0.06476	0.827	0.05431
0.8077	0.06501	0.0693	0.058
0.8445	0.08092	0.03697	0.03759
0.05896	0.8384	0.06829	0.03433
0.04148	0.04028	0.8761	0.04217

MOTIF ACAACGTG

0.7335	0.1016	0.09669	0.06823
0.07995	0.7419	0.07822	0.09995
0.7731	0.08206	0.09101	0.05387
0.8086	0.06957	0.05594	0.06592
0.06052	0.7905	0.08585	0.06312
0.08882	0.06747	0.7746	0.06914
0.06521	0.04355	0.06627	0.825
0.06898	0.09781	0.7365	0.09673

MOTIF ATAACGGC

0.7208	0.1077	0.09433	0.07712

0.09016 0.1268 0.07612 0.7069
0.7763 0.06742 0.1006 0.05569
0.7849 0.07992 0.06371 0.07144
0.08562 0.7646 0.0817 0.06804
0.08677 0.06667 0.7853 0.06122
0.0961 0.06082 0.7302 0.1129
0.09706 0.7339 0.07362 0.09548

MOTIF GTAACGTG
0.09554 0.08755 0.7393 0.07757
0.08522 0.09013 0.09749 0.7272
0.7533 0.07973 0.09539 0.0716
0.7811 0.07403 0.07128 0.07359
0.09226 0.7285 0.09132 0.08793
0.1008 0.06332 0.7455 0.09044
0.06359 0.03941 0.0682 0.8288
0.067 0.07242 0.7788 0.08183

MOTIF AGGTATCATA
0.8217 0.03909 0.07511 0.06411
0.07005 0.0532 0.7664 0.1104
0.08444 0.07483 0.7657 0.07501
0.09572 0.0673 0.03788 0.7991
0.8059 0.04214 0.07144 0.08053
0.07149 0.1183 0.05186 0.7584
0.07296 0.7763 0.07636 0.07438
0.7951 0.08227 0.05731 0.06536
0.04835 0.05096 0.0549 0.8458
0.7986 0.07178 0.05136 0.07827

MOTIF CCTGGTAGTT
0.07931 0.8039 0.04692 0.0699
0.07733 0.7924 0.05337 0.0769
0.0459 0.03603 0.03215 0.8859
0.06215 0.05765 0.7787 0.1015
0.05226 0.04626 0.8303 0.07118
0.03982 0.04505 0.03621 0.8789
0.8484 0.03136 0.05229 0.06794
0.04263 0.05154 0.8418 0.06408
0.07303 0.05012 0.02945 0.8474
0.04245 0.06324 0.04737 0.8469

MOTIF AAGTCAATGT
0.7715 0.07907 0.05871 0.09076
0.7536 0.05677 0.06508 0.1245
0.1275 0.1027 0.6635 0.1064
0.09508 0.09023 0.07396 0.7407
0.1016 0.6956 0.07636 0.1264
0.77 0.0859 0.0526 0.09148
0.7637 0.05237 0.09037 0.0936

0.09517	0.08286	0.06737	0.7546
0.0726	0.1019	0.7336	0.09195
0.08413	0.06815	0.07544	0.7723

MOTIF GTGGGTTA

0.1105	0.0893	0.6717	0.1286
0.0427	0.06854	0.04482	0.8439
0.06315	0.0541	0.7737	0.109
0.1159	0.07299	0.7194	0.09169
0.09738	0.06888	0.6849	0.1489
0.06139	0.04652	0.06759	0.8245
0.08253	0.09002	0.09753	0.7299
0.7101	0.06884	0.1312	0.08984

MOTIF CTTATGGT

0.1109	0.6705	0.0708	0.1478
0.1327	0.06747	0.05332	0.7465
0.05677	0.06149	0.07872	0.803
0.7115	0.1113	0.081	0.09622
0.06768	0.04749	0.0541	0.8307
0.07604	0.07459	0.7571	0.09223
0.1146	0.06144	0.6942	0.1298
0.1117	0.06169	0.03324	0.7933

MOTIF ATACGTGG

0.7529	0.0508	0.1162	0.08012
0.06921	0.06578	0.05555	0.8095
0.803	0.06949	0.06005	0.06744
0.1337	0.5918	0.1098	0.1647
0.1111	0.08757	0.7426	0.05864
0.05891	0.06519	0.06207	0.8138
0.08574	0.04993	0.7555	0.1088
0.07952	0.05257	0.8124	0.0555

MOTIF CTACCAGG

0.08114	0.68	0.1602	0.07865
0.1275	0.07714	0.05738	0.7379
0.7812	0.05203	0.07284	0.09388
0.07916	0.7542	0.07343	0.09322
0.09414	0.7191	0.07934	0.1074
0.8249	0.03821	0.07462	0.0623
0.09895	0.06123	0.7135	0.1264
0.1104	0.08205	0.6973	0.1102

MOTIF CCGATAAC

0.114	0.7444	0.03982	0.1018
0.1101	0.6659	0.08861	0.1354
0.1181	0.05847	0.7035	0.1199
0.7355	0.08279	0.105	0.0768
0.06839	0.09333	0.07802	0.7603

0.7701	0.0553	0.09613	0.07849
0.743	0.1028	0.06473	0.08945
0.0964	0.6939	0.08255	0.1271

MOTIF GTAAGTGG

0.1746	0.06303	0.6531	0.1092
0.05958	0.04636	0.0737	0.8204
0.6738	0.1082	0.1209	0.0972
0.6537	0.1453	0.08271	0.1183
0.1196	0.07333	0.7354	0.07167
0.05687	0.07168	0.0459	0.8256
0.05888	0.0624	0.7428	0.1359
0.09914	0.05326	0.7436	0.104

MOTIF AGTCAATG

0.7608	0.05266	0.0672	0.1193
0.09951	0.09365	0.7015	0.1053
0.09271	0.08401	0.1063	0.717
0.07699	0.7276	0.09467	0.1008
0.7859	0.0832	0.05281	0.07812
0.7424	0.07797	0.09753	0.0821
0.08262	0.0696	0.08479	0.763
0.08653	0.1307	0.6928	0.08993

MOTIF CCCAGTCA

0.0807	0.7156	0.07815	0.1256
0.1227	0.694	0.07648	0.1068
0.09682	0.7025	0.08692	0.1137
0.7864	0.05503	0.04553	0.1131
0.09767	0.08431	0.6792	0.1388
0.07154	0.06987	0.07858	0.78
0.07429	0.7395	0.07507	0.1112
0.8234	0.05223	0.05083	0.07359

MOTIF ACCGAT

0.8027	0.03979	0.05194	0.1056
0.07149	0.8453	0.05081	0.03242
0.08658	0.676	0.07287	0.1646
0.06989	0.09445	0.7269	0.1087
0.8192	0.06738	0.04125	0.07221
0.06293	0.0704	0.06828	0.7984

MOTIF TAAGCT

0.07112	0.07025	0.04909	0.8095
0.8027	0.0627	0.06622	0.06841
0.834	0.04126	0.04319	0.08155
0.09356	0.06302	0.7437	0.0997
0.1014	0.721	0.07214	0.1054
0.0631	0.04767	0.05504	0.8342

MOTIF AACGCA

0.8174	0.06477	0.0445	0.07333
0.8241	0.06388	0.02839	0.08361
0.09189	0.7256	0.08006	0.1024
0.1217	0.1531	0.5674	0.1578
0.08242	0.788	0.06542	0.06412
0.8758	0.05147	0.03562	0.03713

MOTIF GCGTAT

0.09241	0.0684	0.7416	0.09761
0.09179	0.6721	0.115	0.1211
0.14	0.08492	0.6461	0.129
0.06495	0.05065	0.06411	0.8203
0.8145	0.04704	0.06832	0.07011
0.05276	0.07491	0.06428	0.8081

MOTIF ACTCAC

0.8482	0.03534	0.05466	0.06184
0.06921	0.7546	0.1028	0.0734
0.09111	0.1106	0.07198	0.7263
0.07608	0.7684	0.08098	0.07459
0.8805	0.04227	0.03574	0.0415
0.09416	0.7442	0.05833	0.1033

MOTIF TTACGA

0.07862	0.07718	0.06879	0.7754
0.04233	0.04997	0.0795	0.8282
0.7691	0.06245	0.05435	0.1141
0.1311	0.6582	0.08704	0.1237
0.08045	0.07338	0.7318	0.1144
0.8137	0.04337	0.04749	0.09545

MOTIF GTGGGT

0.07016	0.07776	0.7629	0.08922
0.06542	0.04991	0.04648	0.8382
0.05629	0.0508	0.8037	0.08923
0.1433	0.0801	0.673	0.1036
0.09151	0.07487	0.7198	0.1138
0.05097	0.03806	0.04935	0.8616

MOTIF TATCAG

0.09922	0.06967	0.08047	0.7506
0.7984	0.04917	0.07391	0.07847
0.0659	0.1047	0.0649	0.7645
0.08095	0.7511	0.07113	0.09682
0.8024	0.05258	0.0811	0.06394
0.1045	0.05403	0.7041	0.1374

MOTIF TAGGGA

0.08664	0.07464	0.0472	0.7915

0.8431	0.03922	0.04429	0.07342
0.08457	0.06461	0.7382	0.1126
0.1309	0.05522	0.7229	0.09096
0.1299	0.0719	0.7424	0.05577
0.8188	0.04783	0.06946	0.06386

MOTIF CGGTAC

0.1296	0.5936	0.07201	0.2048
0.06674	0.0785	0.8028	0.05199
0.1023	0.06432	0.777	0.05643
0.07983	0.06312	0.07314	0.7839
0.7443	0.0628	0.08374	0.1091
0.07661	0.7565	0.07823	0.08866

MOTIF CCCAGT

0.08346	0.76	0.0624	0.09419
0.08124	0.747	0.08147	0.09033
0.09402	0.7479	0.07663	0.08149
0.7811	0.05216	0.06371	0.103
0.08657	0.0888	0.716	0.1086
0.09994	0.07945	0.04525	0.7754

MOTIF AGTCAC

0.8259	0.04213	0.06122	0.07073
0.07589	0.1427	0.7006	0.08077
0.06525	0.07482	0.07994	0.78
0.07412	0.7833	0.07008	0.07252
0.8569	0.05134	0.0409	0.05089
0.1368	0.668	0.08406	0.1111

MOTIF GGAATG

0.0892	0.06178	0.6839	0.1651
0.07267	0.113	0.6983	0.1159
0.7498	0.1017	0.06529	0.08318
0.7926	0.06767	0.06097	0.07874
0.04464	0.05603	0.05009	0.8492
0.06366	0.06376	0.7814	0.09119

MOTIF CGCAAC

0.1028	0.6403	0.09624	0.1607
0.1031	0.07527	0.702	0.1197
0.0876	0.7778	0.06358	0.07099
0.7905	0.09606	0.0547	0.05874
0.7905	0.09064	0.05305	0.06579
0.05834	0.8029	0.05378	0.085

MOTIF TGTATC

0.05293	0.06479	0.06641	0.8159
0.07989	0.05624	0.7451	0.1187
0.06559	0.08656	0.07306	0.7748

0.791	0.05626	0.06295	0.08982
0.05777	0.07142	0.0627	0.8081
0.05995	0.7372	0.1209	0.08194

MOTIF TTATCAGAAT

0.09772	0.04153	0.06053	0.8002
0.07514	0.0772	0.08709	0.7606
0.7912	0.07214	0.05651	0.08015
0.0899	0.0525	0.09296	0.7646
0.0937	0.7436	0.08609	0.0766
0.8278	0.0413	0.04454	0.08637
0.1243	0.08228	0.6903	0.1031
0.7919	0.07537	0.05763	0.07508
0.7517	0.05988	0.06759	0.1209
0.106	0.0841	0.0664	0.7435

MOTIF AACCTTCAGC

0.723	0.1143	0.08038	0.08235
0.811	0.05849	0.05994	0.07059
0.1335	0.6956	0.07248	0.09837
0.1508	0.6716	0.06341	0.1141
0.08021	0.07862	0.05505	0.7861
0.07578	0.06689	0.06906	0.7883
0.06339	0.7789	0.06821	0.08948
0.772	0.06045	0.04892	0.1186
0.08862	0.1296	0.652	0.1298
0.09182	0.7846	0.03187	0.0917

MOTIF AGCAGAAT

0.8049	0.05038	0.06307	0.08162
0.1195	0.09099	0.6596	0.1299
0.1414	0.6685	0.1068	0.08329
0.846	0.0222	0.0497	0.0821
0.1318	0.06275	0.742	0.06336
0.8254	0.07172	0.04079	0.0621
0.7773	0.0552	0.08548	0.08205
0.1307	0.1108	0.08492	0.6736

MOTIF CCTTCCCA

0.08457	0.7138	0.05278	0.1488
0.06998	0.6986	0.04274	0.1887
0.1531	0.0567	0.03801	0.7522
0.07059	0.0928	0.07394	0.7627
0.1079	0.6592	0.119	0.1139
0.1177	0.6896	0.04447	0.1483
0.09565	0.6871	0.09296	0.1243
0.6558	0.1412	0.04821	0.1548

MOTIF ACTCATCT

0.7443	0.08445	0.04352	0.1277

0.09869	0.684	0.0716	0.1457
0.1223	0.08046	0.04237	0.7548
0.09711	0.7324	0.08311	0.08737
0.7492	0.07575	0.05365	0.1214
0.0898	0.06587	0.06948	0.7748
0.1012	0.7789	0.04413	0.07571
0.1168	0.06494	0.07896	0.7393

MOTIF ATGCATAG

0.8053	0.04358	0.063	0.0881
0.1325	0.0533	0.09399	0.7202
0.09127	0.09029	0.7057	0.1128
0.1286	0.6872	0.1008	0.08349
0.8449	0.0431	0.03891	0.07304
0.1796	0.07119	0.08692	0.6623
0.7413	0.06846	0.107	0.08322
0.1058	0.0819	0.6963	0.116

MOTIF CTTGTCAT

0.07892	0.6763	0.09172	0.1531
0.07953	0.06275	0.0477	0.81
0.08171	0.0562	0.06434	0.7978
0.09341	0.11	0.6432	0.1534
0.07635	0.09824	0.07116	0.7543
0.08769	0.7053	0.114	0.09302
0.6585	0.1109	0.0738	0.1568
0.08847	0.0752	0.06849	0.7678

MOTIF ACTCAAAG

0.8266	0.04166	0.06136	0.07038
0.1694	0.5995	0.1169	0.1141
0.1465	0.07429	0.0898	0.6894
0.1249	0.7494	0.06897	0.05666
0.8093	0.08638	0.04294	0.06139
0.7806	0.06716	0.05113	0.1011
0.804	0.06146	0.0715	0.06309
0.1577	0.1307	0.616	0.09557

MOTIF CTGTTTCT

0.1007	0.659	0.08901	0.1513
0.08055	0.0594	0.06685	0.7932
0.1039	0.1625	0.5508	0.1828
0.07391	0.06335	0.06425	0.7985
0.08606	0.1047	0.07668	0.7325
0.05044	0.08649	0.09441	0.7687
0.07738	0.6886	0.09319	0.1409
0.06044	0.05942	0.05079	0.8294

MOTIF CTGTCGTT

0.08064	0.6921	0.08196	0.1453

0.07524	0.09283	0.06832	0.7636
0.09664	0.0892	0.696	0.1182
0.07284	0.09316	0.0719	0.7621
0.09006	0.5541	0.161	0.1949
0.07975	0.08287	0.7017	0.1357
0.04647	0.06821	0.04845	0.8369
0.04172	0.09943	0.07607	0.7828

MOTIF ACCATCTC

0.7293	0.0763	0.062	0.1324
0.1353	0.7001	0.07394	0.09066
0.09975	0.7507	0.05061	0.09897
0.7262	0.09144	0.07322	0.1091
0.06077	0.08045	0.05194	0.8068
0.09279	0.6861	0.1126	0.1085
0.1127	0.07407	0.05294	0.7603
0.09192	0.6883	0.089	0.1308

MOTIF CATCCG

0.05083	0.8022	0.09047	0.0565
0.8226	0.06566	0.03791	0.07385
0.09299	0.04368	0.03543	0.8279
0.03599	0.8272	0.06418	0.07261
0.1686	0.6446	0.05155	0.1352
0.1442	0.08858	0.6505	0.1167

MOTIF AACGCC

0.8319	0.05585	0.04869	0.06355
0.8079	0.05569	0.05647	0.07991
0.1143	0.6782	0.1328	0.07469
0.1884	0.07466	0.6006	0.1364
0.1495	0.7351	0.06492	0.05048
0.09518	0.778	0.06677	0.06003

MOTIF TAGACG

0.1016	0.05913	0.09832	0.741
0.7689	0.07503	0.06692	0.08914
0.06599	0.06483	0.8042	0.06501
0.8079	0.04819	0.04481	0.09907
0.1474	0.5816	0.1214	0.1496
0.1598	0.04788	0.7132	0.0791

MOTIF CCGTCA

0.06038	0.7773	0.0655	0.09683
0.121	0.7173	0.04128	0.1204
0.1531	0.1135	0.5491	0.1843
0.07131	0.09216	0.05903	0.7775
0.06653	0.82	0.0619	0.05157
0.7555	0.05949	0.07492	0.1101

MOTIF GGTAAG

0.0719	0.05306	0.7743	0.1007
0.1349	0.05616	0.7445	0.06445
0.07507	0.09744	0.1159	0.7116
0.7861	0.05633	0.07578	0.08181
0.8413	0.02767	0.05846	0.07259
0.1225	0.05372	0.7464	0.07742

MOTIF TATCAG

0.09951	0.1093	0.07241	0.7187
0.7796	0.08065	0.05848	0.08123
0.08771	0.05924	0.06793	0.7851
0.07975	0.7459	0.07503	0.09936
0.8417	0.03761	0.04601	0.07471
0.1034	0.07661	0.7465	0.07353

MOTIF CTTATC

0.1022	0.7535	0.06255	0.08167
0.07782	0.04279	0.03987	0.8395
0.04973	0.09022	0.06536	0.7947
0.6607	0.1046	0.1016	0.1331
0.05119	0.07975	0.04639	0.8227
0.07554	0.7938	0.04362	0.08706

MOTIF CAGAGT

0.1093	0.7026	0.09321	0.09488
0.8579	0.03182	0.04481	0.0655
0.0971	0.07207	0.7436	0.08719
0.8249	0.03889	0.0627	0.07348
0.1376	0.04476	0.7377	0.07998
0.1165	0.06964	0.09055	0.7233

MOTIF CAGGAA

0.1336	0.7006	0.1004	0.06534
0.8661	0.04237	0.03485	0.05669
0.1963	0.0398	0.6571	0.1069
0.1267	0.1086	0.7072	0.05749
0.8463	0.05641	0.05074	0.04651
0.8045	0.07443	0.06159	0.05946

MOTIF CATAGA

0.08217	0.7672	0.07073	0.07993
0.8665	0.04605	0.03556	0.0519
0.1142	0.06966	0.08696	0.7292
0.7451	0.08842	0.07323	0.09326
0.07156	0.08359	0.7647	0.08014
0.8342	0.06302	0.03276	0.06999

MOTIF GGGCAA

0.1227	0.05339	0.736	0.08793

0.1049	0.04697	0.7518	0.09638
0.1131	0.08027	0.729	0.07768
0.0943	0.7198	0.102	0.084
0.8682	0.03539	0.04557	0.05088
0.8214	0.04098	0.0689	0.0687

MOTIF GTCAAT

0.1326	0.09035	0.695	0.08205
0.09958	0.04218	0.09275	0.7655
0.08673	0.7674	0.07984	0.06605
0.8138	0.04549	0.03804	0.1027
0.8134	0.0573	0.06079	0.06852
0.135	0.0658	0.05116	0.748

MOTIF TCTGCG

0.06595	0.0533	0.05388	0.8269
0.07468	0.7449	0.08106	0.09933
0.07381	0.04451	0.03357	0.8481
0.05988	0.08773	0.7311	0.1213
0.1014	0.7215	0.04525	0.1318
0.1342	0.1277	0.523	0.2152

MOTIF TCTGTT

0.05529	0.05015	0.05083	0.8437
0.06123	0.72	0.09805	0.1207
0.05807	0.0411	0.03817	0.8627
0.05963	0.1034	0.7051	0.1319
0.09844	0.08991	0.06107	0.7506
0.03618	0.04994	0.05895	0.8549

MOTIF TTGCGA

0.07925	0.06043	0.04896	0.8114
0.05506	0.01972	0.07184	0.8534
0.05698	0.06091	0.7674	0.1147
0.1615	0.6113	0.07387	0.1534
0.07393	0.1095	0.6859	0.1306
0.7816	0.05937	0.05974	0.09933

MOTIF CACTCA

0.1418	0.6962	0.06415	0.09788
0.7935	0.07575	0.02878	0.102
0.08935	0.7348	0.07599	0.09984
0.1297	0.07367	0.06091	0.7357
0.0692	0.8053	0.06341	0.0621
0.7912	0.0567	0.04447	0.1076

MOTIF TAAGGC

0.1325	0.1081	0.09774	0.6616
0.8196	0.05326	0.07823	0.04888
0.8189	0.03313	0.06432	0.08368

0.1657	0.0541	0.7031	0.07709
0.09575	0.06296	0.7937	0.0476
0.1056	0.6803	0.09051	0.1235

MOTIF AAAGCG

0.8255	0.06295	0.06991	0.04168
0.8246	0.06739	0.05398	0.05405
0.8229	0.04675	0.08364	0.04672
0.1058	0.07388	0.7526	0.06781
0.1703	0.6143	0.1168	0.09861
0.2396	0.0783	0.5673	0.1148

MOTIF CAGCGA

0.08474	0.7829	0.0774	0.055
0.8556	0.03806	0.0475	0.05887
0.1514	0.06632	0.6408	0.1415
0.1434	0.7278	0.06114	0.06766
0.1523	0.106	0.6604	0.0813
0.8484	0.05887	0.04012	0.05259

MOTIF CTTCCC

0.06644	0.7782	0.0612	0.09416
0.08172	0.06279	0.03098	0.8245
0.06892	0.05374	0.06616	0.8112
0.1074	0.7081	0.1034	0.08119
0.07796	0.7216	0.04627	0.1542
0.1013	0.6788	0.05571	0.1642

MOTIF AACGAC

0.8065	0.06917	0.07977	0.04452
0.8249	0.04611	0.07596	0.05302
0.1061	0.7377	0.07872	0.07749
0.1659	0.1376	0.5791	0.1175
0.8245	0.08323	0.05235	0.03995
0.1127	0.7479	0.07155	0.0679

MOTIF TGTAAG

0.0682	0.04709	0.09713	0.7876
0.09368	0.06159	0.751	0.09378
0.09223	0.1355	0.0891	0.6832
0.7581	0.06427	0.08321	0.09446
0.7836	0.05133	0.06288	0.1022
0.07665	0.05745	0.796	0.06985

MOTIF CTGGCATCCG

0.04698	0.834	0.06288	0.05616
0.09302	0.05432	0.04418	0.8085
0.04877	0.07503	0.7903	0.08588
0.1017	0.05345	0.7905	0.05434
0.04749	0.755	0.1053	0.09219

0.7718	0.09268	0.06628	0.06922
0.03571	0.0334	0.1119	0.819
0.04296	0.8581	0.05812	0.04086
0.04447	0.8238	0.06113	0.07065
0.05317	0.07241	0.8161	0.05827

MOTIF TCGAAGACGA

0.1465	0.1107	0.08702	0.6558
0.1803	0.6448	0.1067	0.0681
0.09343	0.07403	0.76	0.07258
0.8054	0.04798	0.08973	0.0569
0.7369	0.1441	0.05966	0.05929
0.05632	0.09358	0.7246	0.1255
0.8246	0.06869	0.04391	0.06282
0.1022	0.752	0.07427	0.07154
0.1125	0.06275	0.7362	0.08857
0.7688	0.06763	0.1207	0.04291

MOTIF CACGACTCTA

0.0907	0.7623	0.09543	0.05162
0.7725	0.07406	0.05898	0.09448
0.1071	0.7361	0.04974	0.1071
0.09418	0.07145	0.7577	0.07669
0.7525	0.0825	0.05034	0.1147
0.07136	0.7614	0.09457	0.07272
0.07271	0.0791	0.0912	0.757
0.09961	0.7957	0.0618	0.04292
0.08149	0.1358	0.1148	0.6679
0.7148	0.1179	0.06393	0.1034

MOTIF TCGTTACT

0.06971	0.06296	0.1049	0.7625
0.08394	0.7803	0.05604	0.07976
0.05978	0.09303	0.7484	0.09881
0.0721	0.06819	0.08894	0.7708
0.06181	0.06632	0.0846	0.7873
0.6902	0.08176	0.1049	0.1232
0.06373	0.7183	0.08619	0.1318
0.07604	0.07047	0.06154	0.792

MOTIF GCTACACC

0.1075	0.1037	0.7061	0.08266
0.08482	0.7091	0.1265	0.07963
0.1227	0.1051	0.114	0.6583
0.7122	0.1406	0.05351	0.09362
0.07603	0.7826	0.1012	0.04019
0.6806	0.09721	0.1155	0.1067
0.1016	0.7273	0.08369	0.08745
0.07545	0.797	0.0465	0.08107

MOTIF GCCGCTTG

0.07081	0.08976	0.7846	0.05479
0.08217	0.7059	0.09611	0.1158
0.0631	0.7761	0.08143	0.0794
0.07104	0.05076	0.801	0.07723
0.04819	0.7609	0.1123	0.07855
0.1253	0.1405	0.0922	0.642
0.09727	0.1262	0.0906	0.6859
0.0434	0.09297	0.8119	0.05168

MOTIF CTAGCGGG

0.05513	0.8329	0.05875	0.0532
0.1212	0.08067	0.1145	0.6837
0.6359	0.1158	0.09699	0.1514
0.05804	0.09586	0.7442	0.1019
0.07326	0.7155	0.1193	0.09194
0.05741	0.09638	0.8017	0.04455
0.08639	0.068	0.7495	0.0961
0.0912	0.146	0.6615	0.1013

MOTIF ACCCCAAG

0.7369	0.1165	0.07622	0.07036
0.09606	0.6623	0.1465	0.09513
0.1311	0.6853	0.1004	0.08318
0.1112	0.7387	0.09665	0.05344
0.09631	0.7615	0.07159	0.07059
0.7263	0.07133	0.1235	0.07882
0.7636	0.105	0.06605	0.0653
0.1106	0.09909	0.7366	0.05376

MOTIF CACGACTC

0.08969	0.7523	0.1055	0.05247
0.721	0.1204	0.07721	0.08137
0.08766	0.7537	0.08247	0.07614
0.1295	0.07237	0.718	0.08014
0.7225	0.1012	0.08433	0.09196
0.06668	0.7494	0.1033	0.08065
0.0862	0.113	0.06582	0.735
0.1022	0.7629	0.07887	0.05607

MOTIF ATCGACGT

0.6962	0.07193	0.104	0.1278
0.1133	0.102	0.08559	0.6991
0.06674	0.7582	0.09473	0.08032
0.08929	0.1367	0.7037	0.07037
0.6268	0.09331	0.07183	0.208
0.1244	0.7074	0.08851	0.07969
0.07198	0.07432	0.7802	0.07351
0.1585	0.09687	0.07152	0.6731

MOTIF GAGGCGTT

0.05177	0.04421	0.8361	0.06795
0.7062	0.1367	0.05874	0.09838
0.09179	0.06638	0.7736	0.06824
0.06684	0.07113	0.7478	0.1142
0.111	0.6436	0.1084	0.1369
0.09948	0.1388	0.7103	0.05139
0.06067	0.07479	0.09692	0.7676
0.1031	0.06904	0.1377	0.6902

MOTIF ATGGTATC

0.6949	0.09597	0.0976	0.1115
0.08577	0.04131	0.08086	0.7921
0.08459	0.1202	0.7105	0.08478
0.08277	0.0504	0.8029	0.06394
0.09669	0.07433	0.06912	0.7599
0.6683	0.1092	0.1109	0.1116
0.08479	0.06288	0.1068	0.7455
0.1058	0.6718	0.1361	0.08618

MOTIF CATTCGTG

0.05759	0.7454	0.1054	0.09158
0.7299	0.1166	0.08894	0.0646
0.0829	0.0569	0.1221	0.7381
0.06612	0.1511	0.05433	0.7285
0.05178	0.7008	0.121	0.1264
0.08021	0.08928	0.7486	0.08196
0.09199	0.05835	0.0511	0.7986
0.0902	0.1015	0.7083	0.09996

MOTIF AATAACGC

0.7568	0.07525	0.08663	0.08133
0.7249	0.05869	0.1496	0.06685
0.06239	0.103	0.0703	0.7643
0.7816	0.07416	0.1032	0.04098
0.8018	0.08321	0.06728	0.04771
0.09156	0.6891	0.1557	0.06356
0.1363	0.1001	0.6725	0.09101
0.195	0.6738	0.07125	0.05986

MOTIF TAACGC

0.06088	0.09279	0.0646	0.7817
0.7716	0.06417	0.1128	0.05137
0.8247	0.06334	0.06778	0.0442
0.05734	0.7524	0.117	0.07331
0.09344	0.1115	0.7371	0.05801
0.126	0.7106	0.08403	0.07943

MOTIF AGTCGT

0.6892	0.1463	0.09879	0.06568

0.08361 0.05626 0.7654 0.09473
0.04202 0.06645 0.08238 0.8091
0.0497 0.8012 0.05557 0.09352
0.06612 0.05695 0.7981 0.07885
0.06024 0.07879 0.05856 0.8024

MOTIF TCGGAT
0.1032 0.08697 0.05731 0.7525
0.04623 0.7963 0.07683 0.08061
0.06107 0.06545 0.8138 0.05972
0.1168 0.0457 0.7532 0.08429
0.7972 0.07438 0.06812 0.06031
0.03709 0.1005 0.09082 0.7716

MOTIF TATCGT
0.08936 0.09392 0.0786 0.7381
0.7973 0.05956 0.07995 0.0632
0.0674 0.05537 0.08462 0.7926
0.06521 0.6753 0.1669 0.09261
0.1091 0.06272 0.7597 0.06846
0.0732 0.03708 0.06789 0.8218

MOTIF TCCGTC
0.06187 0.09109 0.05224 0.7948
0.0432 0.8101 0.05377 0.09293
0.07301 0.7974 0.0681 0.06153
0.07746 0.09753 0.7547 0.07034
0.06985 0.123 0.06003 0.7472
0.05014 0.8314 0.06702 0.05144

MOTIF ACGCTA
0.768 0.08225 0.0721 0.07767
0.08219 0.7531 0.08994 0.07477
0.08831 0.114 0.7144 0.08329
0.1134 0.7369 0.06495 0.08474
0.06603 0.1097 0.07667 0.7476
0.8022 0.04966 0.0894 0.05869

MOTIF TATTCG
0.05216 0.06714 0.08957 0.7911
0.8131 0.08027 0.06267 0.04394
0.05083 0.05558 0.07997 0.8136
0.04 0.1686 0.05134 0.74
0.0749 0.781 0.07366 0.07046
0.0729 0.06169 0.7755 0.08994

MOTIF TAGCGA
0.05753 0.07379 0.1008 0.7679
0.8465 0.03235 0.05658 0.06457
0.1349 0.08327 0.6972 0.08459

0.1243	0.7007	0.09642	0.07852
0.08553	0.1204	0.7684	0.02563
0.748	0.06645	0.07823	0.1073

MOTIF ACCCGT

0.693	0.1693	0.04992	0.08777
0.07426	0.6592	0.1005	0.166
0.0962	0.7744	0.02867	0.1007
0.06809	0.8693	0.02591	0.03675
0.09784	0.07988	0.7169	0.1054
0.1051	0.06801	0.08136	0.7455

MOTIF GGGATC

0.06586	0.08054	0.7824	0.07116
0.03273	0.05442	0.8417	0.07118
0.08212	0.07711	0.7754	0.06533
0.6927	0.1715	0.07316	0.06266
0.05344	0.05558	0.08467	0.8063
0.06364	0.7312	0.1324	0.07276

MOTIF AGAGTC

0.7197	0.1441	0.07485	0.06132
0.05338	0.06861	0.8113	0.06669
0.8058	0.06086	0.06744	0.06592
0.08607	0.08431	0.7523	0.07728
0.1122	0.1058	0.08992	0.692
0.05538	0.8288	0.06611	0.04966

MOTIF ACACCT

0.8355	0.06318	0.03854	0.0628
0.07271	0.8145	0.05199	0.06079
0.7688	0.05484	0.08548	0.09088
0.0916	0.7999	0.05711	0.05135
0.1012	0.7797	0.05548	0.06362
0.09948	0.05813	0.09083	0.7516

MOTIF GCGTAC

0.08493	0.09703	0.7505	0.06753
0.1039	0.6684	0.1272	0.1005
0.09456	0.07842	0.7463	0.08069
0.06058	0.0563	0.1185	0.7646
0.7253	0.1168	0.08023	0.07772
0.06462	0.7402	0.1196	0.07557

MOTIF ACGCCT

0.7487	0.1131	0.08068	0.05747
0.06667	0.8136	0.04499	0.07472
0.1756	0.0656	0.6463	0.1126
0.1296	0.7509	0.07355	0.04595
0.05129	0.8642	0.02459	0.05993

0.09555	0.05942	0.07616	0.7689

MOTIF CCACGA

0.07874	0.7987	0.06882	0.05376
0.07979	0.7047	0.1589	0.05664
0.7501	0.1058	0.06583	0.07819
0.1004	0.7758	0.05754	0.06624
0.08075	0.0836	0.7675	0.06812
0.7763	0.08326	0.08426	0.05619

MOTIF TAGCCA

0.08893	0.1097	0.13	0.6713
0.8568	0.06677	0.0219	0.05457
0.05964	0.07949	0.7605	0.1004
0.09683	0.7091	0.08389	0.1102
0.09482	0.7866	0.09224	0.02634
0.7239	0.09402	0.09809	0.08398

MOTIF ATACGA

0.849	0.06322	0.04625	0.04151
0.09915	0.07507	0.1282	0.6975
0.6971	0.1338	0.04955	0.1196
0.05759	0.7826	0.07344	0.08633
0.08801	0.1167	0.7324	0.06296
0.8346	0.05193	0.05939	0.05413

MOTIF GCCTTA

0.06022	0.06998	0.7748	0.095
0.06097	0.763	0.08864	0.08737
0.07327	0.747	0.1219	0.05778
0.07414	0.08298	0.06932	0.7736
0.07392	0.1151	0.1018	0.7091
0.7123	0.1236	0.08713	0.07696

MOTIF GGGTAG

0.05637	0.05387	0.7777	0.1121
0.08651	0.08668	0.7323	0.09446
0.08712	0.103	0.7492	0.06065
0.07581	0.05478	0.1289	0.7405
0.6956	0.1216	0.07906	0.1038
0.05458	0.09025	0.7839	0.07127

MOTIF GATTCG

0.05716	0.07285	0.7532	0.1168
0.8211	0.06596	0.05752	0.05545
0.0692	0.1511	0.1101	0.6696
0.07358	0.07881	0.0614	0.7862
0.07878	0.7773	0.08335	0.06059
0.07095	0.08046	0.7555	0.09307

MOTIF CTGACT

0.08335	0.7144	0.09759	0.1047
0.08532	0.07677	0.1276	0.7103
0.07645	0.05576	0.8067	0.06105
0.7397	0.07556	0.08504	0.09975
0.05185	0.8218	0.05339	0.07292
0.09311	0.06323	0.09205	0.7516

MOTIF GCCCAGGCGT

0.07682	0.07581	0.7862	0.06115
0.1286	0.7297	0.0727	0.06902
0.05114	0.8044	0.07793	0.06654
0.1079	0.6737	0.1453	0.07308
0.7404	0.07788	0.06402	0.1177
0.04331	0.09221	0.815	0.04945
0.05601	0.06962	0.7965	0.07789
0.0988	0.7694	0.07336	0.05845
0.07816	0.1072	0.7508	0.06383
0.06724	0.06549	0.1254	0.7419

MOTIF TCCCGCTCAT

0.08374	0.05823	0.05821	0.7998
0.06117	0.7816	0.1098	0.04738
0.1027	0.661	0.1673	0.06905
0.08092	0.7195	0.09333	0.1063
0.0763	0.07483	0.7785	0.0704
0.05395	0.7846	0.1141	0.04737
0.04663	0.07117	0.06586	0.8163
0.0557	0.8325	0.07377	0.03805
0.7247	0.1346	0.0722	0.06842
0.04708	0.08855	0.1043	0.76

MOTIF AGGCGTGAGA

0.6515	0.1622	0.09959	0.08675
0.07183	0.1112	0.7385	0.07848
0.1356	0.04848	0.7682	0.04769
0.07506	0.7608	0.08152	0.08258
0.08931	0.07412	0.7508	0.08575
0.08523	0.07224	0.1006	0.7419
0.03524	0.1483	0.7385	0.07798
0.6686	0.1341	0.1413	0.05601
0.05019	0.1002	0.7219	0.1277
0.7546	0.08624	0.1049	0.05427

MOTIF GGCGTTAG

0.03551	0.04977	0.8737	0.04099
0.07995	0.04622	0.8316	0.04228
0.1001	0.7477	0.06986	0.0823
0.0444	0.1051	0.7944	0.05613
0.04746	0.1249	0.07288	0.7548

0.04088	0.1673	0.173	0.6188
0.6465	0.1492	0.1353	0.0689
0.04794	0.05492	0.8316	0.0655

MOTIF CCAAGGGC

0.08935	0.7652	0.08488	0.06054
0.06435	0.8136	0.06919	0.05286
0.6285	0.1267	0.148	0.09676
0.7544	0.06699	0.1067	0.07188
0.06534	0.07115	0.8168	0.0467
0.08945	0.08813	0.7802	0.04221
0.159	0.132	0.6015	0.1074
0.06615	0.7916	0.06908	0.07317

MOTIF CCCAGCGC

0.05385	0.7741	0.09395	0.07813
0.04656	0.7998	0.0751	0.07857
0.06669	0.6833	0.1524	0.09764
0.6917	0.1347	0.08763	0.08597
0.05615	0.1127	0.7612	0.07002
0.06884	0.7725	0.1213	0.03728
0.05796	0.1141	0.7427	0.08522
0.05234	0.8423	0.0573	0.04807

MOTIF AGACTCGC

0.6632	0.1378	0.1177	0.08122
0.09811	0.07558	0.7757	0.05058
0.6541	0.172	0.09967	0.07429
0.04742	0.8201	0.08383	0.04861
0.08713	0.1236	0.1147	0.6746
0.06983	0.6919	0.1568	0.08156
0.08701	0.06115	0.7602	0.09169
0.05306	0.7602	0.1151	0.07158

MOTIF CTGAACGG

0.07729	0.8026	0.07503	0.04504
0.07552	0.06902	0.08822	0.7672
0.03924	0.1375	0.7656	0.05774
0.6288	0.1257	0.1829	0.0626
0.6508	0.1107	0.1784	0.06005
0.09657	0.7658	0.08658	0.05105
0.04418	0.1021	0.8025	0.0512
0.122	0.09153	0.7366	0.04995

MOTIF GCCAGTCT

0.0698	0.1387	0.7276	0.06386
0.04895	0.7814	0.07699	0.09269
0.0847	0.7309	0.1096	0.07482
0.6005	0.1565	0.1205	0.1225
0.09148	0.09	0.7484	0.07016

0.09803	0.1312	0.1126	0.6581
0.06374	0.8183	0.06125	0.05671
0.09636	0.08519	0.08993	0.7285

MOTIF ATCGGTCA

0.6043	0.1799	0.1323	0.08349
0.09666	0.115	0.1037	0.6847
0.05499	0.7253	0.1295	0.09018
0.08496	0.1015	0.7314	0.0821
0.05216	0.1175	0.7651	0.06533
0.085	0.1019	0.0679	0.7452
0.06502	0.8358	0.06061	0.0386
0.7217	0.1196	0.1089	0.04989

MOTIF CTCACGCC

0.08937	0.7547	0.09912	0.0568
0.06123	0.1135	0.1415	0.6838
0.1042	0.7299	0.1321	0.03381
0.6895	0.09546	0.1375	0.07752
0.08953	0.7442	0.09947	0.06679
0.0855	0.08286	0.7357	0.09596
0.04595	0.8352	0.04836	0.07053
0.04844	0.8026	0.06374	0.08518

MOTIF TTTCCGGC

0.05368	0.1091	0.06624	0.7709
0.03461	0.1238	0.147	0.6946
0.05637	0.1223	0.1024	0.7189
0.04476	0.7874	0.1083	0.05947
0.08618	0.7704	0.04463	0.09877
0.07345	0.1221	0.6663	0.1381
0.05028	0.07466	0.8146	0.06042
0.05699	0.7835	0.0851	0.07444

MOTIF CCCAGAAG

0.07058	0.7712	0.0853	0.07294
0.04864	0.8095	0.08907	0.05283
0.1287	0.713	0.08113	0.07721
0.6589	0.1221	0.1295	0.08953
0.06912	0.1344	0.7561	0.04033
0.6996	0.08435	0.1535	0.0625
0.7364	0.09356	0.07747	0.0926
0.07348	0.08162	0.7949	0.05002

MOTIF CCGGGA

0.05384	0.7894	0.1079	0.04889
0.08571	0.7525	0.1028	0.05902
0.07454	0.06038	0.7744	0.09064
0.0713	0.07452	0.8109	0.04326
0.08624	0.0643	0.8158	0.0337

0.7905	0.04742	0.1081	0.05397

MOTIF AGGCGT

0.6367	0.171	0.0951	0.09718
0.03162	0.02234	0.8915	0.05456
0.05731	0.05321	0.819	0.07048
0.04627	0.8356	0.0584	0.05968
0.06203	0.02942	0.8635	0.04508
0.06302	0.09242	0.1111	0.7334

MOTIF TGAGCG

0.07788	0.08952	0.142	0.6906
0.03306	0.08275	0.8524	0.03178
0.8005	0.05846	0.09739	0.04366
0.04537	0.1048	0.8198	0.03006
0.08158	0.7845	0.07805	0.05586
0.07136	0.07996	0.7683	0.08037

MOTIF CCACGG

0.03287	0.8411	0.08105	0.04494
0.07109	0.7771	0.1023	0.04952
0.7277	0.1089	0.03336	0.1301
0.1212	0.7339	0.121	0.02385
0.0301	0.07895	0.8521	0.0388
0.1023	0.09621	0.7207	0.08075

MOTIF GCGGTC

0.06034	0.07683	0.7551	0.1078
0.06718	0.7428	0.1087	0.08128
0.05973	0.1093	0.7473	0.08373
0.05282	0.1239	0.7795	0.04377
0.06116	0.1205	0.05008	0.7683
0.04922	0.7971	0.09412	0.05957

MOTIF ATTGCG

0.6439	0.1919	0.1063	0.05795
0.07636	0.1089	0.06837	0.7463
0.06388	0.1057	0.1732	0.6572
0.03548	0.06881	0.8459	0.04985
0.05753	0.7747	0.1278	0.03993
0.06349	0.1158	0.7539	0.06685

MOTIF GCGAGT

0.04513	0.0936	0.794	0.06728
0.07116	0.7828	0.06972	0.07635
0.06774	0.06882	0.8246	0.03879
0.754	0.07177	0.08979	0.08445
0.04838	0.1149	0.7764	0.06031
0.08182	0.133	0.1513	0.6339

MOTIF TCCCGC

0.06166	0.07941	0.05003	0.8089
0.04476	0.8191	0.08713	0.04896
0.08123	0.7599	0.1001	0.05873
0.05681	0.7767	0.07325	0.09322
0.07128	0.07617	0.7434	0.1091
0.04956	0.7713	0.1218	0.05733

MOTIF TCAGCG

0.06432	0.09389	0.08104	0.7608
0.03743	0.8149	0.1102	0.03743
0.6879	0.08151	0.1412	0.08942
0.05024	0.06052	0.8092	0.08007
0.06137	0.7836	0.09935	0.05565
0.08769	0.1078	0.714	0.0906

MOTIF CTTGCG

0.06127	0.8498	0.05706	0.03183
0.08682	0.09326	0.07737	0.7426
0.05588	0.06401	0.0834	0.7967
0.03365	0.09961	0.8196	0.04712
0.06151	0.7354	0.1148	0.08834
0.07557	0.09485	0.7537	0.07592

MOTIF CATCGC

0.05214	0.8598	0.06305	0.02498
0.6511	0.115	0.1216	0.1123
0.0466	0.1018	0.09865	0.753
0.04259	0.7899	0.1133	0.05416
0.07669	0.0911	0.7615	0.07066
0.05772	0.7869	0.08127	0.07414

MOTIF GCGTCT

0.06464	0.09828	0.7703	0.06676
0.05701	0.7963	0.0765	0.07015
0.03319	0.07946	0.8226	0.06476
0.08342	0.09816	0.1196	0.6988
0.0318	0.8481	0.06986	0.05022
0.1011	0.1292	0.1707	0.599

MOTIF TAAGGC

0.08773	0.127	0.1291	0.6562
0.6724	0.1292	0.1566	0.04176
0.7334	0.07707	0.1346	0.05498
0.04953	0.07619	0.82	0.05426
0.04904	0.06122	0.8217	0.06805
0.05409	0.8146	0.0791	0.0522

MOTIF CCAGGT

0.04173	0.8414	0.06499	0.05183

0.08131	0.746	0.0982	0.07448
0.7933	0.06034	0.06308	0.08333
0.07769	0.08727	0.7711	0.06396
0.04864	0.08889	0.8197	0.04274
0.08371	0.08457	0.123	0.7088

MOTIF CCAGTA

0.06102	0.7858	0.08027	0.07295
0.09982	0.7371	0.08566	0.07737
0.7722	0.07482	0.08272	0.0703
0.07669	0.07513	0.7903	0.05786
0.1133	0.1312	0.1598	0.5957
0.695	0.1603	0.06117	0.08359

MOTIF TGGCGT

0.1135	0.1658	0.0935	0.6272
0.04492	0.05137	0.857	0.04671
0.0523	0.07131	0.8343	0.04209
0.04832	0.7726	0.1074	0.07172
0.07803	0.05829	0.8155	0.04816
0.08185	0.1156	0.06429	0.7383

MOTIF GGGAAC

0.06123	0.06185	0.8133	0.06362
0.05614	0.08628	0.8157	0.04191
0.07861	0.08434	0.7918	0.04521
0.7288	0.06729	0.1466	0.05724
0.6877	0.1403	0.1149	0.05707
0.07857	0.7564	0.09188	0.07315

MOTIF GGTCCC

0.0721	0.1283	0.7152	0.08444
0.04112	0.0962	0.7826	0.08011
0.06399	0.05527	0.02884	0.8519
0.03057	0.8187	0.09133	0.05941
0.07154	0.777	0.05684	0.09461
0.05847	0.8227	0.04214	0.07672

MOTIF GACCGA

0.04452	0.06261	0.8341	0.05875
0.6594	0.1588	0.08341	0.09842
0.05143	0.7955	0.1189	0.03422
0.07049	0.7843	0.06719	0.07805
0.05462	0.1168	0.7865	0.04211
0.762	0.08988	0.08389	0.0642

MOTIF ATTACC

0.6996	0.1092	0.1063	0.08485
0.07675	0.09353	0.08249	0.7472
0.05781	0.1203	0.216	0.6059

0.6106	0.143	0.1386	0.1078
0.03239	0.8327	0.09512	0.03975
0.03923	0.8328	0.05447	0.07353

MOTIF TCGCAG

0.04613	0.07925	0.1207	0.7539
0.04974	0.7662	0.08503	0.09907
0.0477	0.08523	0.7681	0.09894
0.03696	0.8364	0.08533	0.0413
0.6787	0.1479	0.09955	0.07377
0.05099	0.05926	0.8307	0.05903

MOTIF TCGGTA

0.08381	0.08498	0.09891	0.7323
0.07051	0.7004	0.1071	0.122
0.0844	0.05023	0.7948	0.07055
0.03604	0.05695	0.8682	0.03882
0.08214	0.1211	0.13	0.6668
0.5999	0.2217	0.1052	0.07321

MOTIF CGTAACCGTA

0.08637	0.7346	0.07952	0.09954
0.1261	0.09632	0.6865	0.09108
0.1123	0.07368	0.08816	0.7259
0.7288	0.1163	0.08199	0.07287
0.712	0.09386	0.0809	0.1132
0.08596	0.7586	0.07004	0.08545
0.09359	0.7226	0.07622	0.1076
0.1201	0.111	0.6686	0.1003
0.1094	0.07446	0.08183	0.7343
0.7245	0.0866	0.09254	0.09638

MOTIF TACGCATACG

0.08297	0.1155	0.09787	0.7037
0.7358	0.09932	0.08283	0.08209
0.08556	0.7235	0.07643	0.1145
0.1195	0.08975	0.6974	0.09337
0.08904	0.7114	0.09945	0.1002
0.7789	0.08222	0.0748	0.0641
0.09085	0.09229	0.07528	0.7416
0.7411	0.08471	0.09831	0.07588
0.09663	0.6779	0.08809	0.1374
0.1297	0.08565	0.7024	0.08223

MOTIF TCCGCATACG

0.08444	0.09877	0.09584	0.721
0.1035	0.7259	0.08629	0.08435
0.08962	0.7367	0.07888	0.09478
0.1043	0.09081	0.7059	0.09904
0.07816	0.7394	0.09652	0.08595

0.7405	0.1133	0.08449	0.06172
0.09998	0.1055	0.08183	0.7127
0.6582	0.1457	0.1022	0.09388
0.08824	0.7193	0.08141	0.111
0.09037	0.08945	0.7385	0.08164

MOTIF CGTATCCGTA

0.07832	0.7437	0.07125	0.1067
0.122	0.0914	0.6972	0.08941
0.08722	0.07825	0.08908	0.7455
0.743	0.09112	0.08217	0.08368
0.08645	0.08629	0.07429	0.753
0.09919	0.7207	0.09225	0.08787
0.08956	0.7478	0.06072	0.102
0.1116	0.07609	0.7216	0.09071
0.09698	0.08309	0.08782	0.7321
0.702	0.1026	0.08928	0.1061

MOTIF CGTAACGT

0.08394	0.7345	0.0902	0.09132
0.0994	0.09284	0.7301	0.07771
0.08236	0.07357	0.08325	0.7608
0.7312	0.1075	0.1011	0.06021
0.7645	0.09195	0.06789	0.07561
0.07125	0.7858	0.06746	0.0755
0.09744	0.08701	0.73	0.08559
0.0633	0.06317	0.08025	0.7933

MOTIF CGTAAC

0.06271	0.823	0.0499	0.06442
0.06473	0.05438	0.8306	0.0503
0.06158	0.04891	0.0424	0.8471
0.8207	0.07145	0.06266	0.04521
0.8596	0.04932	0.04452	0.04659
0.03819	0.8908	0.03273	0.03827

MOTIF CGCATA

0.07161	0.7986	0.04781	0.08193
0.08612	0.07322	0.7942	0.04642
0.04066	0.8579	0.05893	0.04247
0.895	0.03813	0.03549	0.03133
0.03339	0.04641	0.04049	0.8797
0.8366	0.06019	0.06263	0.04056

MOTIF ATTCGC

0.8618	0.05777	0.04183	0.03862
0.0381	0.04052	0.05528	0.8661
0.05509	0.08228	0.0807	0.7819
0.0355	0.8818	0.04102	0.0417
0.04477	0.04278	0.8748	0.03769

0.0497	0.7788	0.08594	0.08557

MOTIF GACGTA

0.05459	0.05779	0.8646	0.023
0.88	0.04194	0.03686	0.04116
0.04448	0.8512	0.05196	0.05238
0.04625	0.03989	0.8811	0.03277
0.0767	0.06932	0.0757	0.7783
0.8722	0.01463	0.06857	0.04458

MOTIF CGTCTA

0.03379	0.8972	0.03094	0.03809
0.04469	0.06179	0.8664	0.02715
0.03497	0.06073	0.04866	0.8556
0.06938	0.8186	0.05572	0.0563
0.0535	0.05877	0.07835	0.8094
0.7142	0.1193	0.08061	0.08587

MOTIF CGATAG

0.04912	0.8401	0.06494	0.04585
0.03717	0.06302	0.8866	0.01316
0.8185	0.04651	0.06813	0.06681
0.04556	0.05955	0.06495	0.8299
0.8641	0.02537	0.0745	0.03606
0.0561	0.06291	0.8111	0.0699

MOTIF TAAGCG

0.04415	0.05387	0.06801	0.834
0.8725	0.04988	0.03669	0.04092
0.8237	0.05177	0.05643	0.06813
0.0524	0.06836	0.8383	0.04098
0.039	0.8074	0.08696	0.06666
0.0671	0.03869	0.8241	0.07008

MOTIF TACTGG

0.05101	0.04554	0.03822	0.8652
0.8678	0.03266	0.05621	0.04334
0.06714	0.8312	0.05105	0.05065
0.06171	0.05529	0.06113	0.8219
0.03086	0.04128	0.8857	0.0422
0.06392	0.06027	0.7725	0.1033

MOTIF CCCGTA

0.05389	0.8605	0.03754	0.04809
0.05068	0.8716	0.03569	0.04199
0.047	0.8586	0.04479	0.04962
0.07338	0.06256	0.8064	0.05769
0.06755	0.05187	0.03429	0.8463
0.8142	0.06311	0.05597	0.06671

MOTIF AGTAACGG

0.8083	0.05952	0.06525	0.06691
0.06161	0.06498	0.8196	0.05377
0.1078	0.09713	0.09947	0.6956
0.7728	0.07408	0.08731	0.06577
0.8035	0.0666	0.07158	0.05837
0.09295	0.7234	0.08569	0.098
0.08715	0.06395	0.7638	0.08507
0.06817	0.04663	0.8153	0.06992

MOTIF CGTATG

0.06577	0.7794	0.07226	0.08254
0.03064	0.04547	0.8754	0.04845
0.03949	0.06472	0.05782	0.838
0.821	0.05189	0.06751	0.05964
0.03481	0.02017	0.04276	0.9023
0.04355	0.0465	0.8652	0.0448

MOTIF CGTACT

0.01173	0.882	0.05634	0.04993
0.07979	0.04933	0.8186	0.05227
0.04409	0.03944	0.08331	0.8332
0.8416	0.05439	0.05886	0.04514
0.04705	0.8509	0.04651	0.05553
0.05524	0.06309	0.02323	0.8584

MOTIF CGCATACG

0.07101	0.7776	0.07253	0.07888
0.1029	0.08344	0.7364	0.07728
0.06959	0.8387	0.07465	0.01709
0.783	0.08524	0.07974	0.05203
0.08566	0.09916	0.09772	0.7175
0.8152	0.09641	0.03017	0.05817
0.06517	0.7663	0.06923	0.09933
0.0962	0.07978	0.7602	0.06383

MOTIF TGAACG

0.05657	0.08651	0.0883	0.7686
0.05752	0.05104	0.8355	0.0559
0.8078	0.06451	0.0577	0.06998
0.8358	0.0767	0.039	0.04852
0.03003	0.8493	0.07541	0.04523
0.0321	0.05004	0.8823	0.03559

MOTIF ACCGTA

0.8918	0.04002	0.03057	0.03762
0.038	0.8934	0.03329	0.03535
0.06678	0.8271	0.04399	0.06209
0.07421	0.06418	0.8028	0.05884
0.06749	0.04596	0.0939	0.7926

0.8372 0.06114 0.05384 0.04779

MOTIF ATTGCG
0.7715 0.05609 0.1017 0.07066
0.08457 0.06578 0.06403 0.7856
0.05832 0.04828 0.06956 0.8238
0.07875 0.07144 0.774 0.07582
0.03091 0.83 0.07222 0.06685
0.03974 0.03425 0.874 0.052

MOTIF GACGCT
0.04109 0.0639 0.8445 0.05051
0.8772 0.05402 0.02364 0.04512
0.05027 0.8375 0.0428 0.06947
0.05796 0.0535 0.8549 0.03367
0.05396 0.8057 0.09283 0.04755
0.07312 0.06126 0.07872 0.7869

MOTIF TACGGA
0.05841 0.05846 0.0664 0.8167
0.875 0.01509 0.05793 0.05201
0.06017 0.7982 0.06122 0.08039
0.05866 0.05745 0.8329 0.05103
0.04583 0.03905 0.8945 0.02066
0.8613 0.03663 0.0488 0.05327

MOTIF GGTACCGA
0.05928 0.07181 0.7896 0.07936
0.06914 0.06829 0.7922 0.07035
0.08071 0.09681 0.07391 0.7486
0.7917 0.06013 0.08424 0.0639
0.07012 0.7963 0.06536 0.06822
0.08378 0.7466 0.1025 0.06717
0.07114 0.07967 0.7782 0.071
0.7742 0.06765 0.08468 0.07345

MOTIF AACGTA
0.8814 0.05134 0.04719 0.0201
0.8667 0.05337 0.0392 0.04075
0.04885 0.8265 0.06979 0.05486
0.08206 0.04427 0.8112 0.06247
0.0618 0.04351 0.0577 0.837
0.8893 0.03811 0.05698 0.01557

MOTIF GGAACG
0.03916 0.05686 0.8695 0.03446
0.07153 0.06565 0.8145 0.04829
0.826 0.06562 0.05508 0.05326
0.8072 0.07036 0.07078 0.0517
0.03202 0.8657 0.05731 0.045

0.03867	0.04009	0.8893	0.03196

MOTIF ACGTTACC

0.7827	0.08346	0.05782	0.07599
0.09382	0.7503	0.0692	0.08671
0.08625	0.09397	0.7296	0.09016
0.06929	0.0623	0.08397	0.7844
0.07684	0.08864	0.08131	0.7532
0.7583	0.06126	0.08759	0.09282
0.05801	0.7807	0.0695	0.09182
0.08647	0.7665	0.0671	0.07991

MOTIF CCGATACG

0.07712	0.7828	0.06438	0.07571
0.08662	0.7418	0.08756	0.08405
0.07817	0.09411	0.7718	0.05596
0.7372	0.08506	0.1005	0.07719
0.1072	0.1066	0.08904	0.6971
0.728	0.09112	0.1063	0.07463
0.05937	0.8177	0.0475	0.07541
0.08011	0.08122	0.7617	0.07698

MOTIF GCGAATGT

0.1016	0.1033	0.7462	0.04899
0.08605	0.7579	0.07294	0.08312
0.107	0.0814	0.7044	0.1072
0.7092	0.1048	0.08346	0.1025
0.7994	0.09912	0.06142	0.04009
0.05679	0.08637	0.1264	0.7304
0.05243	0.08798	0.8198	0.03983
0.04845	0.08222	0.09831	0.771

MOTIF CGCCGCAATA

0.1012	0.7289	0.08504	0.08482
0.1227	0.07834	0.6824	0.1165
0.0868	0.7466	0.08149	0.08516
0.09716	0.6992	0.08566	0.1179
0.1164	0.08304	0.7124	0.08816
0.09855	0.7696	0.07215	0.05967
0.7585	0.111	0.07053	0.0599
0.7557	0.08231	0.09374	0.06822
0.1117	0.1536	0.1354	0.5993
0.7102	0.1029	0.09743	0.08948

MOTIF GCCGGTAAGA

0.08457	0.0718	0.7541	0.08954
0.09009	0.7484	0.08127	0.08027
0.1167	0.6925	0.09453	0.09628
0.1085	0.08414	0.7063	0.101
0.1014	0.08604	0.7198	0.0928

0.1221 0.1363 0.1361 0.6054
0.721 0.08586 0.1277 0.06547
0.7531 0.08202 0.0915 0.07333
0.09825 0.09458 0.7374 0.06981
0.7709 0.08687 0.08751 0.05476

MOTIF ATTGCG
0.8254 0.05693 0.06078 0.05688
0.04164 0.06002 0.03733 0.861
0.03401 0.03374 0.06975 0.8625
0.03928 0.04347 0.872 0.04521
0.06611 0.8322 0.04288 0.05884
0.08232 0.05856 0.7935 0.06566

MOTIF TCCGTAAG
0.07402 0.06722 0.06927 0.7895
0.06195 0.7901 0.07501 0.07292
0.08964 0.7315 0.07811 0.1007
0.09069 0.09261 0.7122 0.1045
0.07561 0.1032 0.09534 0.7259
0.7514 0.08417 0.08763 0.07683
0.8002 0.06947 0.0609 0.06944
0.08364 0.07065 0.7712 0.07455

MOTIF AACGCT
0.8297 0.06824 0.05547 0.04662
0.9073 0.03932 0.01446 0.03894
0.08961 0.7556 0.07696 0.07786
0.06413 0.07821 0.7959 0.06175
0.04617 0.8835 0.05847 0.01188
0.05002 0.0441 0.03686 0.869

MOTIF TACGCT
0.04211 0.06784 0.05528 0.8348
0.8085 0.07243 0.05497 0.06406
0.06333 0.7969 0.05911 0.08068
0.06247 0.05438 0.8166 0.06655
0.03463 0.892 0.03939 0.03395
0.03195 0.04805 0.03688 0.8831

MOTIF TGAACG
0.04298 0.04713 0.04828 0.8616
0.05268 0.06132 0.8439 0.04206
0.8427 0.06772 0.0484 0.04122
0.8376 0.06089 0.06326 0.03821
0.06634 0.7907 0.08296 0.06004
0.03974 0.04776 0.8516 0.06094

MOTIF CGTAAG
0.07366 0.783 0.0681 0.07523

0.06928	0.07048	0.8424	0.01786
0.05253	0.06665	0.08323	0.7976
0.8404	0.05724	0.05862	0.04372
0.8991	0.01653	0.04192	0.04244
0.0566	0.04513	0.8551	0.04319

MOTIF CGCTTA

0.06654	0.7864	0.05992	0.08716
0.05794	0.06745	0.8122	0.06239
0.03947	0.8657	0.04566	0.04912
0.04379	0.05346	0.03847	0.8643
0.04986	0.05716	0.06743	0.8256
0.8258	0.06262	0.06336	0.04823

MOTIF TTCGCA

0.04574	0.0459	0.04285	0.8655
0.03368	0.05472	0.0169	0.8947
0.07818	0.7548	0.07215	0.09484
0.05301	0.05177	0.8154	0.07987
0.01172	0.8945	0.05052	0.04322
0.8196	0.07156	0.04449	0.06439

MOTIF GCGGTAAT

0.08537	0.06274	0.7658	0.08609
0.09112	0.7242	0.08147	0.1032
0.1072	0.06686	0.7146	0.1114
0.07705	0.08331	0.7766	0.06304
0.08973	0.09347	0.08115	0.7357
0.74	0.08265	0.1118	0.06557
0.8032	0.05913	0.06746	0.07021
0.08268	0.06861	0.06638	0.7823

MOTIF TACTGG

0.04356	0.06038	0.03892	0.8571
0.7856	0.04254	0.1003	0.07156
0.07156	0.8225	0.05784	0.04808
0.05079	0.04774	0.0405	0.861
0.03221	0.03918	0.892	0.03656
0.06544	0.08582	0.7682	0.0805

MOTIF ACTGCGTT

0.7364	0.08035	0.1001	0.08311
0.04769	0.8217	0.05582	0.07477
0.06042	0.04496	0.07474	0.8199
0.05806	0.08754	0.7626	0.09182
0.08847	0.754	0.05338	0.1042
0.1006	0.08482	0.7056	0.109
0.07237	0.07146	0.06309	0.7931
0.07059	0.07628	0.09318	0.7599

MOTIF CGTCTA

0.04927	0.8722	0.03255	0.04597
0.03612	0.08159	0.8308	0.05144
0.04497	0.07649	0.07238	0.8062
0.04543	0.8325	0.05084	0.07122
0.06911	0.06387	0.08893	0.7781
0.7819	0.08865	0.05511	0.07433

MOTIF GTTACGGC

0.0747	0.07393	0.756	0.0954
0.0541	0.1031	0.07419	0.7686
0.06975	0.08421	0.1004	0.7457
0.674	0.08453	0.1301	0.1113
0.08596	0.7349	0.08966	0.08944
0.09973	0.07226	0.7307	0.09735
0.05996	0.07572	0.8018	0.06248
0.07797	0.7815	0.06194	0.07856

MOTIF CGCATA

0.09117	0.7608	0.06111	0.08695
0.04837	0.05078	0.8381	0.0627
0.03083	0.8984	0.03747	0.03331
0.8591	0.0565	0.04104	0.04334
0.06365	0.06199	0.068	0.8064
0.8316	0.05888	0.0621	0.04739

MOTIF TACCGC

0.05492	0.08798	0.0564	0.8007
0.782	0.06606	0.08764	0.06429
0.03503	0.8829	0.0446	0.0375
0.06141	0.8078	0.0471	0.08371
0.06236	0.06083	0.8215	0.05527
0.05036	0.8533	0.04554	0.05082

MOTIF AATCGC

0.8235	0.08217	0.04807	0.04625
0.8526	0.04309	0.05148	0.05286
0.07135	0.06672	0.07171	0.7902
0.05218	0.8152	0.07007	0.0625
0.06166	0.04761	0.8356	0.05515
0.03294	0.8764	0.04332	0.04736

MOTIF CTTACCGG

0.06512	0.7902	0.06433	0.08036
0.07247	0.05628	0.08453	0.7867
0.06822	0.1362	0.08946	0.7061
0.6794	0.1052	0.1078	0.1076
0.07643	0.7991	0.06464	0.05981
0.08605	0.7539	0.06798	0.09208
0.08129	0.07281	0.7607	0.08521

0.08205	0.06695	0.7724	0.07857

MOTIF TTCCGC

0.04293	0.0951	0.054	0.808
0.03247	0.04801	0.06361	0.8559
0.03916	0.865	0.05076	0.04504
0.06949	0.7943	0.06151	0.07468
0.05173	0.06238	0.809	0.07686
0.04074	0.8662	0.05074	0.04233

MOTIF AACGCTGC

0.7494	0.09998	0.08634	0.06428
0.7589	0.08542	0.08034	0.07532
0.09669	0.7081	0.09123	0.1039
0.0915	0.07741	0.7397	0.09144
0.07222	0.7962	0.06761	0.06398
0.08066	0.0831	0.05993	0.7763
0.04648	0.05837	0.8288	0.06635
0.08806	0.8058	0.05664	0.04951

MOTIF CTTACC

0.05385	0.8442	0.04209	0.05985
0.04444	0.03907	0.03958	0.8769
0.05845	0.07565	0.04707	0.8188
0.7963	0.07207	0.07247	0.05915
0.05064	0.8481	0.05535	0.0459
0.0592	0.8486	0.03436	0.05787

MOTIF GCGATA

0.04142	0.04231	0.8882	0.0281
0.04793	0.8528	0.04662	0.05266
0.1044	0.07201	0.7738	0.04971
0.8567	0.05147	0.05134	0.04044
0.0734	0.07871	0.07439	0.7735
0.8178	0.04648	0.0787	0.05696

MOTIF GGAACG

0.04649	0.04884	0.8476	0.05705
0.06727	0.08109	0.8121	0.03956
0.8451	0.0609	0.04503	0.04898
0.7974	0.0598	0.1078	0.03498
0.07736	0.7797	0.08939	0.05353
0.04942	0.04354	0.8724	0.03462

MOTIF CGTATG

0.07301	0.7768	0.06936	0.08085
0.01597	0.05183	0.8471	0.08506
0.04368	0.08033	0.05496	0.821
0.8235	0.05095	0.05586	0.06968
0.05011	0.02055	0.05109	0.8782

0.04107	0.03356	0.8853	0.04003

MOTIF CGCTGA

0.07492	0.7803	0.06591	0.07882
0.05162	0.05091	0.8437	0.05376
0.05456	0.8429	0.05925	0.04333
0.04837	0.06839	0.05808	0.8252
0.03828	0.04293	0.8875	0.03127
0.8234	0.07999	0.05209	0.04454

MOTIF ATTACGCT

0.7115	0.09943	0.08183	0.1072
0.05966	0.08082	0.05649	0.803
0.0616	0.06807	0.08188	0.7885
0.7528	0.09901	0.07542	0.07273
0.0917	0.7461	0.06779	0.09437
0.07665	0.08819	0.7597	0.0755
0.06068	0.7937	0.07859	0.06703
0.06933	0.06799	0.04799	0.8147

MOTIF CGCTCA

0.08576	0.7897	0.06066	0.06388
0.06232	0.05533	0.8047	0.07767
0.02818	0.8847	0.04645	0.04066
0.05011	0.0452	0.05201	0.8527
0.04138	0.8686	0.05606	0.03396
0.8177	0.07741	0.05364	0.0512

MOTIF TTTGCG

0.04467	0.04985	0.05307	0.8524
0.03686	0.05346	0.04862	0.8611
0.04238	0.03904	0.06293	0.8556
0.04397	0.0542	0.85	0.05185
0.06104	0.8272	0.05855	0.05322
0.07981	0.06326	0.7782	0.07871

MOTIF AACGTC

0.8783	0.05452	0.01919	0.04799
0.7597	0.08938	0.08561	0.06532
0.04472	0.8675	0.04503	0.04277
0.05662	0.07445	0.7953	0.07367
0.05578	0.06573	0.09169	0.7868
0.05319	0.8757	0.05301	0.01808

MOTIF TTTGCGGT

0.06011	0.08747	0.07426	0.7782
0.05872	0.05666	0.08426	0.8004
0.05185	0.05737	0.06025	0.8305
0.06553	0.09356	0.7706	0.07032
0.07085	0.7826	0.07311	0.07349

0.1099	0.07748	0.7254	0.08729
0.05558	0.09771	0.7653	0.08144
0.08324	0.08455	0.06371	0.7685

MOTIF CTTACGCT

0.07303	0.7611	0.07155	0.09435
0.06207	0.07301	0.06125	0.8037
0.06067	0.1047	0.07424	0.7604
0.6814	0.1293	0.0972	0.09209
0.1005	0.691	0.1027	0.1058
0.08299	0.09264	0.7218	0.1026
0.05948	0.801	0.07185	0.06769
0.06827	0.07748	0.05034	0.8039

MOTIF TCCGCAAT

0.07141	0.05978	0.08292	0.7859
0.06108	0.7897	0.06735	0.08185
0.09952	0.7136	0.08006	0.1068
0.08619	0.08197	0.7174	0.1145
0.07254	0.7988	0.06012	0.06854
0.7997	0.08825	0.04956	0.06254
0.7918	0.05997	0.07512	0.07309
0.0953	0.08386	0.09552	0.7253

MOTIF GATGCGATGC

0.1027	0.1084	0.7029	0.08599
0.7441	0.08528	0.08117	0.08945
0.08486	0.06394	0.1069	0.7443
0.06775	0.08209	0.782	0.06818
0.11	0.6733	0.09265	0.1241
0.1084	0.1056	0.6902	0.09578
0.7297	0.08083	0.09072	0.09872
0.0883	0.07402	0.07902	0.7587
0.06357	0.07312	0.7913	0.07205
0.1072	0.7079	0.1036	0.08133

MOTIF CCTACGTACA

0.104	0.7346	0.09069	0.07074
0.1371	0.7215	0.06193	0.07949
0.1118	0.1009	0.07992	0.7074
0.7244	0.09334	0.07394	0.1084
0.1041	0.6737	0.0898	0.1324
0.1562	0.1051	0.6296	0.1091
0.08919	0.0972	0.07137	0.7422
0.712	0.09662	0.1159	0.07552
0.09127	0.7589	0.06387	0.086
0.7895	0.07309	0.05724	0.08017

MOTIF TATGCG

0.0536	0.05984	0.06263	0.8239

0.8629	0.03927	0.05259	0.04519
0.03202	0.03711	0.04724	0.8836
0.03757	0.03504	0.888	0.03937
0.05792	0.7887	0.05064	0.1028
0.09479	0.05403	0.7785	0.07272

MOTIF ATCGCT

0.8231	0.06251	0.06013	0.05424
0.03716	0.03683	0.01433	0.9117
0.06505	0.7973	0.0702	0.06747
0.05419	0.05698	0.8103	0.07856
0.01912	0.8896	0.04612	0.04515
0.0519	0.0513	0.03887	0.8579

MOTIF CTTACG

0.04836	0.8614	0.04073	0.04947
0.05365	0.04952	0.01905	0.8778
0.04216	0.04999	0.05432	0.8535
0.805	0.07316	0.06336	0.05845
0.01544	0.8344	0.06164	0.08848
0.06528	0.0562	0.8173	0.06125

MOTIF CGTTAC

0.05211	0.831	0.05263	0.06424
0.07901	0.06119	0.7897	0.07009
0.03989	0.05128	0.03953	0.8693
0.04016	0.06808	0.05382	0.8379
0.7688	0.05937	0.08932	0.08246
0.03528	0.8837	0.04095	0.04004

MOTIF AATGCG

0.832	0.05948	0.06201	0.04652
0.8945	0.03428	0.03667	0.0346
0.04363	0.04114	0.05485	0.8604
0.04492	0.04253	0.8599	0.05266
0.06316	0.8134	0.05676	0.06665
0.1039	0.05153	0.7772	0.0674

MOTIF CGAAACGC

0.1015	0.7385	0.08431	0.07571
0.08286	0.0891	0.7553	0.07279
0.7982	0.09186	0.06037	0.04956
0.8038	0.07745	0.06945	0.04926
0.7757	0.06611	0.08907	0.06908
0.08956	0.7477	0.0788	0.08393
0.09415	0.07786	0.7535	0.07451
0.09692	0.7539	0.07989	0.06932

MOTIF GAATGCGA

0.08995	0.06939	0.7726	0.0681

0.7767	0.08653	0.07897	0.05781
0.8071	0.05636	0.07545	0.06107
0.0791	0.06371	0.1101	0.7471
0.06959	0.08478	0.7707	0.07495
0.1021	0.7359	0.08325	0.07875
0.1114	0.07571	0.747	0.06583
0.8275	0.05712	0.05466	0.0607

MOTIF ACATACGC

0.8249	0.06624	0.04696	0.06192
0.07838	0.7988	0.05167	0.07118
0.8355	0.05479	0.06361	0.04615
0.07266	0.09399	0.06099	0.7724
0.7944	0.06709	0.08522	0.05328
0.09447	0.7149	0.07824	0.1124
0.1179	0.07441	0.7185	0.08922
0.08491	0.7899	0.05265	0.07252

MOTIF TAAGCG

0.05892	0.06953	0.07094	0.8006
0.8586	0.04293	0.05197	0.04648
0.8298	0.04765	0.06131	0.06128
0.05015	0.03949	0.8651	0.04523
0.05215	0.8095	0.06915	0.06923
0.07865	0.05991	0.7976	0.06383

MOTIF AACGCT

0.8628	0.05643	0.04059	0.04022
0.8661	0.04546	0.01808	0.0704
0.08006	0.7332	0.0931	0.09368
0.0461	0.06461	0.8427	0.04663
0.05477	0.8844	0.04524	0.0156
0.06231	0.0622	0.05231	0.8232

MOTIF CCGTAATA

0.1175	0.7139	0.06846	0.1001
0.08919	0.7673	0.06063	0.08288
0.09674	0.08563	0.7143	0.1033
0.09463	0.06051	0.06254	0.7823
0.7912	0.07687	0.05701	0.07494
0.7987	0.06923	0.06597	0.06607
0.07995	0.08117	0.0839	0.755
0.8073	0.06393	0.07053	0.05824

MOTIF GAGCGATG

0.07755	0.05912	0.793	0.07034
0.7828	0.06163	0.07722	0.0784
0.06121	0.06153	0.8127	0.06459
0.116	0.729	0.07939	0.07567
0.08304	0.09512	0.7512	0.07063

0.8041	0.05571	0.08033	0.05985
0.08238	0.08022	0.1135	0.7239
0.04975	0.04698	0.8319	0.07137

MOTIF CGTACT

0.0219	0.866	0.04429	0.06779
0.07739	0.05908	0.8056	0.05796
0.06158	0.04556	0.057	0.8359
0.8091	0.05621	0.07585	0.05889
0.04588	0.8452	0.05373	0.05518
0.05405	0.0481	0.01913	0.8787

MOTIF CGTATG

0.06651	0.7659	0.0706	0.09702
0.03149	0.06087	0.8342	0.07342
0.04523	0.0648	0.06088	0.8291
0.8244	0.04513	0.07145	0.059
0.03695	0.01706	0.04019	0.9058
0.03917	0.0369	0.8764	0.04753

MOTIF ATTACG

0.8704	0.04168	0.04037	0.04754
0.05614	0.05555	0.049	0.8393
0.03996	0.05114	0.06076	0.8481
0.8533	0.04713	0.04382	0.05573
0.05904	0.8037	0.05851	0.07875
0.07561	0.04546	0.8139	0.06498

MOTIF AACGTC

0.8495	0.08068	0.02235	0.04747
0.8512	0.04798	0.04225	0.05856
0.051	0.8543	0.03925	0.05542
0.07468	0.06099	0.8181	0.04619
0.06822	0.06507	0.07346	0.7932
0.0531	0.8711	0.06071	0.01506

MOTIF CTCGTA

0.04407	0.8604	0.04416	0.05133
0.06584	0.04233	0.0434	0.8484
0.0657	0.8319	0.03844	0.06392
0.06746	0.06798	0.8189	0.04563
0.04807	0.05641	0.0609	0.8346
0.8345	0.07069	0.04326	0.0516

MOTIF ATTCGC

0.8139	0.07949	0.05372	0.05287
0.05919	0.04819	0.04467	0.8479
0.04515	0.04225	0.07558	0.837
0.04832	0.845	0.0469	0.05981
0.04775	0.0525	0.8385	0.06129

0.06714	0.8222	0.05267	0.05795

MOTIF AGAACG

0.8427	0.05175	0.06384	0.04169
0.05903	0.05538	0.8425	0.04311
0.8084	0.07704	0.06108	0.05351
0.8593	0.03395	0.06322	0.04354
0.07164	0.7769	0.06683	0.08459
0.03813	0.0361	0.8961	0.02964

MOTIF ACGTAACG

0.7928	0.07774	0.06342	0.06601
0.09855	0.7332	0.07117	0.09712
0.08497	0.06638	0.7835	0.06518
0.1096	0.09849	0.07925	0.7127
0.7292	0.09681	0.1038	0.07016
0.7947	0.05933	0.08065	0.06535
0.09499	0.7304	0.07775	0.09691
0.109	0.09603	0.7168	0.07815

MOTIF ACGTACTA

0.8237	0.05455	0.0571	0.06469
0.07811	0.7477	0.06485	0.1093
0.105	0.07645	0.7308	0.08766
0.0689	0.06122	0.05146	0.8184
0.8028	0.07031	0.07252	0.05439
0.09149	0.7252	0.09217	0.09111
0.08974	0.07488	0.06382	0.7716
0.7969	0.07991	0.05654	0.06667

MOTIF CGCTAT

0.09299	0.7457	0.07115	0.09013
0.0495	0.06708	0.8318	0.05164
0.04263	0.8719	0.04027	0.04521
0.04916	0.09039	0.05631	0.8041
0.8305	0.06608	0.03887	0.06455
0.04918	0.05793	0.04349	0.8494

MOTIF ACTACG

0.8481	0.04523	0.06926	0.03743
0.05538	0.8521	0.0457	0.04682
0.05178	0.04195	0.1058	0.8004
0.8442	0.05827	0.04313	0.05441
0.0474	0.8477	0.03935	0.06556
0.06215	0.04252	0.8185	0.07688

MOTIF ACCGTA

0.8499	0.05206	0.04853	0.04952
0.05407	0.8478	0.04515	0.05297
0.05315	0.8667	0.04084	0.03927

0.07419	0.06442	0.8	0.06143
0.07335	0.059	0.0708	0.7969
0.8607	0.05453	0.03934	0.04539

MOTIF TAGTACGG

0.06253	0.07654	0.09787	0.7631
0.7766	0.06697	0.08252	0.07393
0.07528	0.07425	0.738	0.1125
0.0713	0.08068	0.07771	0.7703
0.8141	0.05209	0.0627	0.07107
0.09974	0.7217	0.0884	0.09019
0.08602	0.05754	0.7901	0.06635
0.09512	0.07952	0.7013	0.124

MOTIF GACGTA

0.04787	0.06839	0.8629	0.02085
0.8744	0.04142	0.04006	0.04417
0.0653	0.8081	0.05864	0.06795
0.07194	0.04464	0.8216	0.06184
0.08007	0.05871	0.06723	0.794
0.8607	0.02141	0.07347	0.04438

MOTIF CGGAAT

0.07558	0.7892	0.0602	0.07503
0.04793	0.06442	0.848	0.03964
0.06433	0.05041	0.8309	0.05436
0.8678	0.05459	0.03731	0.04028
0.8055	0.06322	0.06823	0.06306
0.04471	0.053	0.07777	0.8245

MOTIF ATTGCG

0.8195	0.0611	0.06014	0.05922
0.07069	0.04463	0.04733	0.8373
0.04355	0.05186	0.08273	0.8219
0.0493	0.04859	0.8442	0.05789
0.04313	0.8764	0.0414	0.03903
0.09798	0.06256	0.7534	0.08602

MOTIF TCGCATAG

0.07381	0.05997	0.07423	0.792
0.08624	0.7333	0.07992	0.1006
0.09555	0.07591	0.7577	0.07084
0.06122	0.8155	0.06494	0.05834
0.7955	0.07713	0.06534	0.06207
0.07153	0.05701	0.06796	0.8035
0.7373	0.08998	0.08545	0.08732
0.07994	0.07812	0.7431	0.09882

MOTIF ACCGCTATGC

0.6787	0.1051	0.1066	0.1095

0.08317	0.6947	0.1209	0.1013
0.1076	0.6966	0.08411	0.1117
0.1295	0.1039	0.6441	0.1224
0.1026	0.7385	0.0922	0.06666
0.09763	0.09632	0.08316	0.7229
0.6656	0.1105	0.1081	0.1158
0.0706	0.09181	0.07631	0.7613
0.07231	0.09675	0.7636	0.06731
0.08181	0.7379	0.09202	0.08824

MOTIF ACGCCGCTAT

0.6924	0.1255	0.0829	0.09926
0.104	0.6921	0.08388	0.12
0.09874	0.08512	0.7049	0.1113
0.08861	0.7499	0.0743	0.0872
0.1062	0.7302	0.06997	0.09365
0.08917	0.0791	0.6982	0.1335
0.09088	0.7735	0.05919	0.07643
0.0823	0.1165	0.1007	0.7005
0.6725	0.1327	0.1135	0.08133
0.07539	0.1232	0.09278	0.7086

MOTIF CGCGATAG

0.09205	0.7673	0.07394	0.06671
0.0739	0.06751	0.784	0.07457
0.0798	0.7706	0.07767	0.07197
0.05981	0.08131	0.7805	0.07838
0.7869	0.07493	0.08421	0.05395
0.06542	0.08617	0.1167	0.7317
0.7569	0.08711	0.09306	0.06294
0.05585	0.04937	0.8363	0.0585

MOTIF ATCGCCGCAT

0.6923	0.1068	0.1017	0.09922
0.07559	0.0827	0.09558	0.7461
0.09272	0.7347	0.08307	0.08948
0.1189	0.0708	0.7063	0.104
0.09476	0.7521	0.07548	0.07768
0.09922	0.6999	0.08698	0.1139
0.1001	0.0828	0.7273	0.08986
0.07492	0.7686	0.07909	0.07743
0.6937	0.1246	0.1067	0.07492
0.0994	0.08834	0.0988	0.7135

MOTIF CGCTAT

0.07057	0.7982	0.05298	0.07828
0.0703	0.05809	0.7826	0.08897
0.0363	0.8861	0.03898	0.03863
0.04253	0.0766	0.04966	0.8312
0.8481	0.0582	0.04763	0.04608

0.03847 0.0392 0.04487 0.8775

MOTIF GCGGTATC
0.06516 0.07364 0.7842 0.07703
0.0822 0.7536 0.07063 0.0936
0.07282 0.07013 0.776 0.08102
0.07244 0.1092 0.7481 0.0702
0.06641 0.1123 0.09369 0.7276
0.7415 0.07702 0.106 0.07556
0.06467 0.07141 0.1104 0.7535
0.06129 0.7846 0.08126 0.07289

MOTIF CGTTAT
0.05122 0.8179 0.07124 0.0596
0.06057 0.0689 0.8188 0.05178
0.05323 0.07939 0.05654 0.8108
0.03629 0.05505 0.05892 0.8497
0.8565 0.04337 0.04816 0.05192
0.02947 0.04538 0.04596 0.8792

MOTIF GCGGTTAG
0.07339 0.07552 0.7603 0.09081
0.09206 0.736 0.0804 0.09153
0.08327 0.0704 0.769 0.07737
0.07122 0.05956 0.7977 0.07153
0.08002 0.1117 0.07026 0.738
0.04775 0.08377 0.09966 0.7688
0.7323 0.06773 0.1069 0.09312
0.06081 0.08234 0.802 0.05489

MOTIF GTAACCGA
0.07334 0.08994 0.7714 0.06536
0.08996 0.08727 0.07965 0.7431
0.805 0.07613 0.05972 0.05912
0.7527 0.08801 0.07774 0.08157
0.06639 0.8115 0.05628 0.06585
0.06131 0.7966 0.07416 0.06796
0.07538 0.07421 0.7873 0.0631
0.7891 0.07886 0.0644 0.0676

MOTIF AATGCG
0.8314 0.0652 0.06343 0.03994
0.8766 0.03652 0.04232 0.04452
0.04778 0.0389 0.05048 0.8628
0.03498 0.05703 0.854 0.05395
0.06218 0.7933 0.07299 0.07151
0.1014 0.05285 0.7783 0.06753

MOTIF TGCGAA
0.06471 0.04936 0.07265 0.8133

0.02389	0.04823	0.9115	0.01638
0.03755	0.8684	0.04825	0.04581
0.09791	0.05991	0.7676	0.07454
0.8785	0.01932	0.0582	0.044
0.7925	0.06282	0.0837	0.06103

MOTIF TAACCG

0.05948	0.0568	0.05489	0.8288
0.8474	0.06685	0.04656	0.03914
0.7988	0.07402	0.06358	0.06356
0.04974	0.873	0.03194	0.04534
0.05291	0.8491	0.04963	0.04836
0.07247	0.06464	0.8054	0.05752

MOTIF CGTCTA

0.03477	0.8901	0.04292	0.03218
0.09881	0.1233	0.727	0.05085
0.0449	0.06455	0.09258	0.798
0.05349	0.8113	0.06419	0.07098
0.03758	0.05546	0.07541	0.8315
0.8114	0.08533	0.04584	0.05742

MOTIF ATCGCT

0.7994	0.08638	0.06094	0.05324
0.02928	0.05328	0.01467	0.9028
0.07925	0.7806	0.06358	0.07655
0.04523	0.05777	0.8289	0.06806
0.01629	0.8944	0.04383	0.04552
0.05031	0.05577	0.05511	0.8388

MOTIF CGATAC

0.04655	0.8327	0.06525	0.0555
0.05786	0.07701	0.8114	0.05374
0.8624	0.05407	0.0425	0.04104
0.04827	0.0636	0.05828	0.8298
0.8066	0.05838	0.07944	0.05558
0.04175	0.8604	0.04603	0.05181

MOTIF ATACCG

0.871	0.04591	0.04247	0.04063
0.06216	0.06835	0.05192	0.8176
0.8148	0.07237	0.06747	0.04538
0.05179	0.8266	0.07275	0.0489
0.05285	0.8371	0.057	0.05306
0.08085	0.08034	0.7749	0.06391

MOTIF CGTTCA

0.04664	0.8805	0.0457	0.02715
0.1401	0.06713	0.7074	0.08542
0.06102	0.0756	0.06813	0.7953

0.02669	0.0565	0.08035	0.8365
0.03651	0.8442	0.04781	0.0715
0.8373	0.05247	0.05708	0.05315

MOTIF TATGCG

0.06262	0.07658	0.07457	0.7862
0.8429	0.05838	0.05144	0.04732
0.02974	0.03538	0.04353	0.8914
0.03497	0.06112	0.8534	0.0505
0.0431	0.8311	0.06887	0.05692
0.09942	0.05035	0.7526	0.09759

MOTIF ACCCGATG

0.7831	0.06674	0.07654	0.07367
0.06242	0.7484	0.0872	0.102
0.08587	0.7648	0.0848	0.06456
0.09206	0.7692	0.06475	0.07403
0.09053	0.07665	0.7595	0.07329
0.7963	0.06636	0.05728	0.0801
0.07283	0.05601	0.06418	0.807
0.1037	0.08356	0.7312	0.08158

MOTIF ATCGGG

0.8315	0.06156	0.04503	0.06188
0.04809	0.03765	0.0633	0.851
0.06005	0.8198	0.05857	0.06159
0.0505	0.05613	0.8245	0.06885
0.04934	0.05302	0.843	0.05468
0.05052	0.04934	0.8601	0.04

MOTIF CATTCG

0.05623	0.8512	0.04791	0.04464
0.8694	0.05187	0.03287	0.04588
0.04289	0.05026	0.047	0.8599
0.0338	0.04912	0.06865	0.8484
0.04748	0.8009	0.06264	0.08895
0.07943	0.06632	0.8004	0.0539

MOTIF TGACCC

0.04867	0.08246	0.05266	0.8162
0.09869	0.06718	0.7625	0.07167
0.7926	0.0813	0.05721	0.06886
0.04624	0.8184	0.06914	0.06618
0.08262	0.799	0.07447	0.04393
0.04349	0.8433	0.07679	0.03646

MOTIF TAAACG

0.05819	0.06896	0.06451	0.8083
0.8717	0.04409	0.04968	0.03454
0.8445	0.05978	0.04665	0.0491

0.8239	0.07873	0.03972	0.05763
0.04945	0.7955	0.06459	0.09042
0.0457	0.05028	0.8575	0.0465

MOTIF CGGGAT

0.05988	0.8211	0.0567	0.06233
0.056	0.06269	0.8127	0.06859
0.06083	0.05831	0.8358	0.04509
0.03974	0.05841	0.8571	0.04477
0.8151	0.06786	0.04775	0.06927
0.04391	0.05018	0.05466	0.8512

MOTIF CGTAGA

0.04716	0.8371	0.0624	0.05333
0.1311	0.06414	0.7557	0.04907
0.05923	0.09759	0.1004	0.7428
0.7829	0.08552	0.07783	0.05374
0.02543	0.03005	0.8824	0.06209
0.7932	0.09952	0.04901	0.05831

MOTIF TTGCAGTAGG

0.08855	0.08411	0.1031	0.7242
0.09885	0.05514	0.08864	0.7574
0.06747	0.07204	0.7766	0.08385
0.09829	0.6925	0.09549	0.1137
0.7482	0.07959	0.05947	0.1127
0.08263	0.09054	0.7241	0.1027
0.1074	0.1211	0.1184	0.6531
0.7079	0.08704	0.07804	0.127
0.09417	0.0569	0.7508	0.09809
0.1392	0.127	0.6204	0.1133

MOTIF TCTAACGCTA

0.08914	0.0726	0.09639	0.7419
0.09114	0.7416	0.07518	0.09206
0.1244	0.09536	0.05622	0.724
0.7767	0.07605	0.07539	0.07185
0.6926	0.1106	0.0943	0.1026
0.1468	0.6314	0.08802	0.1338
0.1305	0.1013	0.6333	0.1349
0.09214	0.7399	0.07961	0.08833
0.1322	0.0802	0.04977	0.7378
0.7672	0.07516	0.06683	0.09083

MOTIF GCTAACGCTA

0.1015	0.08111	0.7008	0.1165
0.1059	0.7566	0.05803	0.07947
0.1208	0.0975	0.05374	0.728
0.7426	0.09213	0.09361	0.07166
0.681	0.1085	0.122	0.08848

0.1503	0.6384	0.08862	0.1227
0.1296	0.1074	0.6469	0.116
0.08779	0.731	0.07557	0.1056
0.1071	0.08713	0.06233	0.7435
0.7643	0.07262	0.08087	0.0822

MOTIF GCGTGATC

0.07657	0.07003	0.7626	0.09081
0.07068	0.772	0.07038	0.08693
0.1093	0.06038	0.7239	0.1065
0.07214	0.05621	0.05708	0.8146
0.04118	0.1093	0.7901	0.05941
0.7488	0.08288	0.09359	0.07474
0.07425	0.06155	0.06851	0.7957
0.05046	0.7701	0.1162	0.06324

MOTIF TTGACGGC

0.09308	0.07072	0.07206	0.7641
0.05824	0.1129	0.07354	0.7553
0.04348	0.05622	0.8478	0.0525
0.7737	0.07268	0.07435	0.07923
0.09105	0.7191	0.08559	0.1043
0.07951	0.04702	0.7916	0.08184
0.09727	0.07073	0.7412	0.09079
0.06195	0.7946	0.06551	0.07789

MOTIF ATAGCG

0.8426	0.05523	0.05261	0.04959
0.04598	0.05973	0.05251	0.8418
0.8211	0.04685	0.08886	0.04324
0.04639	0.03279	0.8812	0.03963
0.07613	0.7772	0.06338	0.08328
0.0785	0.04873	0.8129	0.05986

MOTIF GGCCTACA

0.07807	0.07802	0.7676	0.07635
0.07025	0.0704	0.7767	0.08259
0.08307	0.7862	0.05772	0.07304
0.05677	0.8118	0.07332	0.05807
0.09768	0.05785	0.08377	0.7607
0.7125	0.108	0.08897	0.09055
0.07075	0.8136	0.06186	0.05383
0.7838	0.04808	0.08013	0.08801

MOTIF TAAGCGCT

0.07216	0.1514	0.08609	0.6904
0.6792	0.09693	0.09817	0.1257
0.7861	0.0642	0.07545	0.07426
0.1037	0.06893	0.7422	0.08516
0.08247	0.6853	0.1168	0.1154

0.1097	0.09683	0.7189	0.07451
0.08999	0.7373	0.07527	0.09746
0.07109	0.05117	0.0645	0.8132

MOTIF TACAGC

0.05402	0.05377	0.05967	0.8325
0.7622	0.069	0.1044	0.06441
0.06391	0.8415	0.05208	0.04249
0.8717	0.02799	0.03914	0.0612
0.06872	0.0583	0.8233	0.04966
0.03901	0.8646	0.04289	0.05351

MOTIF AAACGC

0.8497	0.06019	0.04891	0.04116
0.8671	0.05362	0.04803	0.03125
0.8571	0.03973	0.05298	0.05023
0.06339	0.7701	0.08595	0.08061
0.07323	0.05137	0.8081	0.06734
0.06136	0.8364	0.05544	0.04676

MOTIF CTACTGCA

0.06391	0.8015	0.06735	0.06726
0.09954	0.08164	0.07528	0.7435
0.6698	0.1015	0.1255	0.1032
0.07369	0.7674	0.08389	0.075
0.09109	0.04465	0.05532	0.809
0.09386	0.08881	0.7414	0.07592
0.07433	0.7876	0.06538	0.07269
0.7821	0.0713	0.04721	0.09943

MOTIF CAGTCGTA

0.0761	0.7564	0.08045	0.08702
0.8172	0.06262	0.05274	0.06743
0.06897	0.07205	0.8018	0.05714
0.06274	0.07574	0.0599	0.8016
0.105	0.7015	0.07343	0.12
0.06385	0.06895	0.7815	0.08571
0.09544	0.08033	0.09018	0.7341
0.762	0.1042	0.06693	0.06693

MOTIF ACGCAA

0.8839	0.04341	0.04103	0.03167
0.08839	0.7464	0.07077	0.09442
0.07088	0.04598	0.8141	0.06899
0.05594	0.8515	0.04749	0.04508
0.8426	0.06251	0.04374	0.05117
0.8695	0.05228	0.04688	0.03138

MOTIF GCGTTA

0.04194	0.04471	0.8632	0.05014

0.07288	0.7839	0.0641	0.0791
0.08428	0.05713	0.7675	0.09104
0.04144	0.05601	0.03992	0.8626
0.04246	0.0796	0.08198	0.796
0.8204	0.05227	0.06571	0.06166

MOTIF GCCTAC

0.05144	0.05113	0.8376	0.05978
0.05561	0.8449	0.04973	0.04975
0.03396	0.8845	0.04168	0.03985
0.08051	0.05386	0.0667	0.7989
0.7706	0.07289	0.07224	0.08432
0.05225	0.8522	0.05165	0.04392

MOTIF TTCGAC

0.05948	0.07035	0.02194	0.8482
0.04548	0.06364	0.03779	0.8531
0.04872	0.8313	0.06057	0.05943
0.08052	0.05798	0.7937	0.06779
0.8127	0.05398	0.0519	0.08141
0.01185	0.9073	0.03712	0.0437

MOTIF GCGATT

0.06551	0.0571	0.8034	0.07402
0.05791	0.8282	0.03725	0.07665
0.07797	0.07248	0.7868	0.0627
0.8293	0.0599	0.04457	0.06623
0.04001	0.06509	0.05103	0.8439
0.04429	0.06054	0.07751	0.8177

MOTIF TGACGC

0.04018	0.06934	0.05666	0.8338
0.03417	0.06116	0.8713	0.03341
0.8132	0.05872	0.06936	0.05873
0.07934	0.789	0.05148	0.08016
0.04536	0.03201	0.873	0.04958
0.07941	0.7988	0.06067	0.06116

MOTIF TGCCGTAA

0.09349	0.06751	0.06299	0.776
0.06825	0.04742	0.8264	0.05789
0.06523	0.7754	0.07563	0.08375
0.1421	0.6615	0.05316	0.1432
0.08631	0.07813	0.7352	0.1004
0.08443	0.09675	0.05541	0.7634
0.7268	0.1097	0.0925	0.07094
0.7257	0.08347	0.1172	0.07367

MOTIF CTATCC

0.03094	0.883	0.0333	0.05272

0.07152	0.08885	0.07147	0.7682
0.777	0.08535	0.066	0.07163
0.08182	0.05644	0.0804	0.7813
0.05089	0.818	0.05767	0.07341
0.06825	0.8253	0.04565	0.0608

MOTIF ATCGGC

0.756	0.08604	0.08499	0.07293
0.06398	0.05007	0.07553	0.8104
0.05568	0.8501	0.04589	0.04832
0.06599	0.0383	0.8282	0.06746
0.08006	0.05749	0.7932	0.06928
0.04257	0.8924	0.03134	0.03366

MOTIF ACGCTT

0.8403	0.04423	0.05893	0.05655
0.02563	0.8037	0.07294	0.09771
0.0806	0.0649	0.7746	0.07987
0.0615	0.8367	0.05245	0.04932
0.03921	0.0659	0.01609	0.8788
0.06275	0.05556	0.06678	0.8149

MOTIF AGACGT

0.8039	0.0712	0.0534	0.0715
0.04618	0.04298	0.8736	0.03728
0.8212	0.05799	0.05747	0.06337
0.07283	0.7968	0.06292	0.06743
0.03708	0.03421	0.8479	0.08078
0.08763	0.0471	0.07519	0.7901

MOTIF AAGCGC

0.8016	0.07061	0.06786	0.05989
0.8288	0.0623	0.06638	0.04255
0.06598	0.04393	0.8335	0.05654
0.05737	0.8028	0.06887	0.07096
0.07786	0.05095	0.8077	0.06351
0.05842	0.845	0.04689	0.0497

MOTIF CCAGTA

0.07442	0.7804	0.08146	0.06374
0.06802	0.8174	0.05257	0.06204
0.8755	0.03682	0.03155	0.05617
0.04656	0.08073	0.8115	0.06125
0.04828	0.1125	0.04797	0.7912
0.8357	0.04736	0.06095	0.05601

MOTIF GCTTAC

0.0592	0.04867	0.832	0.06012
0.0471	0.8596	0.03722	0.05611
0.04167	0.0706	0.05037	0.8374

0.05236	0.074	0.07644	0.7972
0.7445	0.07114	0.09555	0.0888
0.06589	0.8372	0.05654	0.04035

MOTIF GTCTAC

0.06686	0.08099	0.7737	0.07842
0.05773	0.06174	0.05266	0.8279
0.01021	0.8847	0.05897	0.04613
0.08188	0.07221	0.02575	0.8202
0.8023	0.04955	0.05651	0.09161
0.05725	0.864	0.0451	0.03366
